# Distinct and relatively mild clinical characteristics of SARS-CoV-2 BA.5 infections against BA.2

**DOI:** 10.1038/s41392-023-01443-2

**Published:** 2023-04-26

**Authors:** Liping Guo, Xue Liu, Yuchen Gu, Jinyan Jiang, Ziyue Yang, Qiuying Lv, Deyin Guo, Yang Yang, Hongzhou Lu, Jing Yuan

**Affiliations:** 1 Shenzhen Key Laboratory of Pathogen and Immunity, National Clinical Research Center for Infectious Disease, State Key Discipline of Infectious Disease, Shenzhen Third People’s Hospital, Second Hospital Affiliated to Southern University of Science and Technology, Shenzhen, China; 2 Centre for Infection and Immunity Study (CIIS), School of Medicine (Shenzhen), Shenzhen Campus of Sun Yat-sen University, Sun Yat-sen University, Shenzhen, China; 3 Shenzhen Center for Disease Control and Prevention, Shenzhen, China

**Keywords:** Vaccines, Infectious diseases

**Dear Editor**,

The global pandemic of severe acute respiratory syndrome coronavirus 2 (SARS-CoV-2) Omicron variant has resulted in its continuous evolution and the emergence of numerous subvariants of Omicron (https://gisaid.org/). Currently, possibly owing to the increased evasion of neutralizing antibodies elicited by previous infection and vaccination and the higher transmissibility, the BA.5 variant has replaced BA.2 variant and dominated the pandemic.^1^ Notably, experimental data from hamsters infection model suggested that BA.4/5 is more pathogenic than BA.2.^2^ However, the comparison of clinical characteristics between human BA.2 and BA.5 infections needs further investigation. In this study, we make a comprehensive comparison of clinical features, viral and antibody kinetics between BA.2 and BA.5 infections.

A total of 1349 laboratory-confirmed SARS-CoV-2 BA.2 (BA.2 group, *N* = 751) and BA.5 variants (BA.5 group, *N* = 598) infected participants were enrolled according to the inclusion and exclusion criteria shown in Supplementary Fig. 1. The vast majority of patients were between the ages of 15 and 64, with larger proportion of patients younger than 15 year-old in BA.5 group (12%, 70/598 vs. 18%, 136/751, *p* = 0.0013) (Fig. 1a). Briefly, the type of vaccines involved in this study were mainly inactivated vaccines (84.07% and 89.62% for the BA.2 and BA.5 patients, respectively; Supplementary Table 1). More than 80% of the BA.5 group had received at least two doses of the SARS-CoV-2 vaccines, and 62.04% of them received a booster dose, both higher than that of BA.2 group (Fig. 1b). Compared with the BA.2 group, the BA.5 group had a higher proportion of asymptomatic infections (31% vs. 18%), but a lower proportion of moderate disease (5% vs. 17%) (Fig. 1c and Supplementary Table 2). The basic information of BA.2 and BA.5 infected patients was provided in Supplementary Table 3. The top 5 frequently reported symptoms for the symptomatic BA.2 infections were cough (289 [47%]), fever (191 [31%]), sore throat (147 [24%]), hawking (100 [16%]) and fatigue (63 [10%]). While for the symptomatic BA.5 infections, fever was the most obvious symptoms (183 [45%]), followed by sore throat (95 [23%]), and then cough (88 [21%]) (Fig. 1d and Supplementary Table 4). Of note, chest tightness was observed only in the BA.2 group, while chilly or shiver was observed only in the BA.5 group. Odds ratios (OR) were used to assess differences in the prevalence of various symptoms of BA.5 infections against BA.2 infections. Of the 31 symptoms assessed, 6 were significantly less prevalent (false discovery rate *p* < 0.05) in BA.5 group than the BA.2 group (OR for cough: 0.31, 95% CI 0.23–0.42 [*p* < 0.001]; OR for hawking: 0.12, 95% CI 0.05–0.23 [*p* < 0.001]; OR for dry throat: 0.24, 95% CI 0.09–0.54 [*p* < 0.001]; OR for throat itching: 0.25, 95% CI 0.09–0.57 [*p* < 0.001]; OR for runny nose: 0.46, 95% CI 0.24–0.85 [*p* = 0.01]; OR for diarrhea: 0.40, 95% CI 0.13–1.03 [*p* = 0.04]). However, fever, unusual muscle pains and pharyngeal discomfort were more likely to be present in the BA.5 group (fever OR 1.80, 95% CI 1.38–2.35 [*p* < 0.001]; unusual muscle pains OR 2.56, 1.42–4.72 [*p* < 0.001], pharyngeal discomfort OR 3.83, 1.09–16.82 [*p* = 0.02]). The computed tomography (CT) manifestations of 597 symptomatic patients from BA.2 and 332 from BA.5 group showed that the proportions of Ground Glass Nodules and Ground Glass Stove (22% vs. 10% with OR 2.63 and *p* < 0.001), Infection focus (7% vs. 1% with OR 6.04 and *p* < 0.001) and Double lung involved (41% vs. 29% with OR 1.76 and *p* < 0.001) were higher in the BA.2 group, while the Fibrous lesions (9% vs. 4% with OR 2.37 and *p* < 0.001) and Multilobar lesions (8% vs. 1% with OR 7.03 and *p* < 0.001) were more frequent in BA.5 group. Additionally, ~42% of the BA.5 infections and 32% of BA.2 infections showed normal CT throughout the disease period with a significant advantage in the BA.5 group (OR 1.53, 1.15–2.04 [*p* = 0.002]) (Fig. 1e and Supplementary Table 4). Further analysis grouped on vaccination doses showed that the differences in symptoms and CT imaging between BA.2 and BA.5 infections mainly existed in the vaccinated participants, especially the booster vaccinated (Supplementary Figs. 2 and 3).

**Fig. 1:**
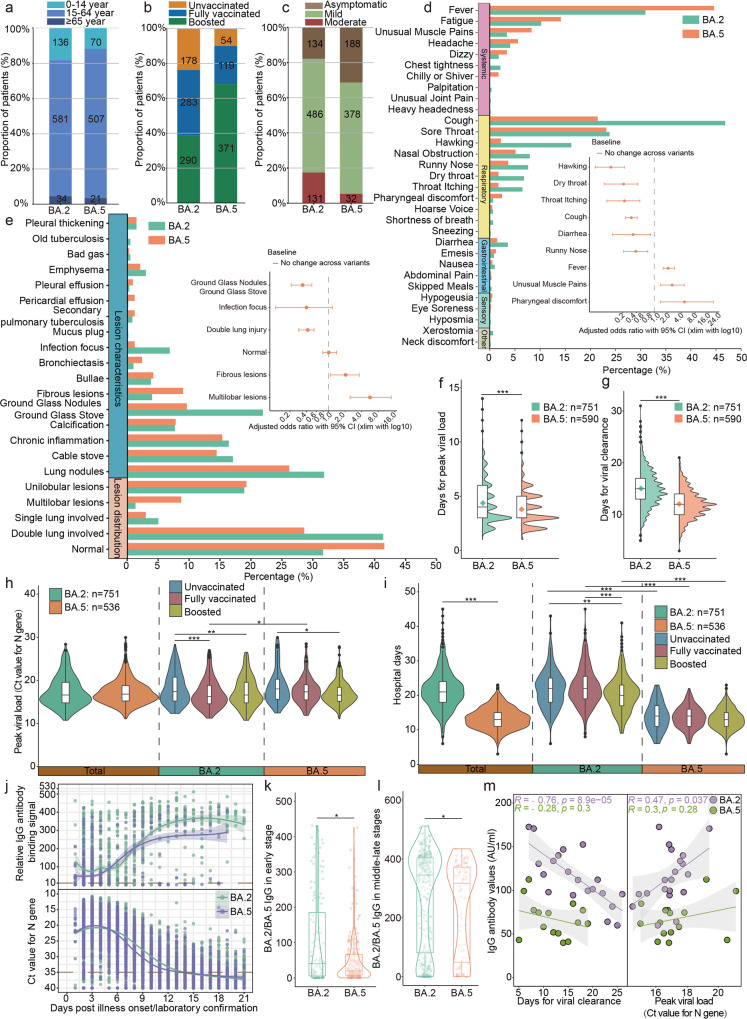
Comparative clinical characteristics against SARS-CoV-2 Omicron BA.2 and BA.5 subvariants. Proportion of Omicron subvariants BA.2 and BA.5 patients grouped by age (**a**), vaccination (**b**), and disease severity (**c**). The numbers in the bar represent the total number of patients in each category. **d** Prevalence of symptoms reported by symptomatic BA.2 and BA.5 infections. Error bars (95% CI) for symptoms with significant differences (*p* < 0.05) are marked. **e** Comparative computed tomography (CT) imaging features and the related proportion in symptomatic BA.2 and BA.5 infections. Data are odds ratios comparing BA.2 and BA.5 prevalence. Error bars indicate 95% CI. Comparison of days for peak viral load (**f**) and days for viral clearance (Viral clearance was defined as Ct values ≥35 for two consecutive qRT-PCR tests within 3 days.) (**g**) between BA.2 and BA.5 acute infections. Comparison of peak viral load (**h**), and days of hospital stay (**i**) for BA.2 and BA.5 infections with different status of vaccination (54 patients with unknown vaccination information were excluded from the BA.5 group). **j** Antibody and viral dynamics of Omicron subvariant BA.2 and BA.5 during hospitalization. **k**, **l** IgG antibody values in the early (within 2 days of infection) and middle-late stages (after 5 days of infection) of infection with the BA.2 and BA.5 variants. **m** Correlation between IgG antibody level at admission and the days of viral clearance and peak viral load using person correlation coefficients. Statistical significance was determined using *t-*test for continuous variables and Wilcoxon rank-sum test for non-normal data, and *p* values <0.05 were considered statistically significant. *p < 0.05, **p < 0.01, ***p < 0.001

Participants in the acute infection stage (defined as the lowest Ct value was below 30 during hospitalization) were analyzed for viral dynamics (Supplementary Table 5). The days to peak viral load was much shorter in BA.5 group (mean: 3.78; 95% CI: 3.65, 3.91) than that of the BA.2 group (mean: 4.35; 95% CI: 4.21, 4.51) (Fig. 1f), suggesting a faster replication and transmissibility for BA.5 variant. Moreover, faster viral clearance (mean: 12.05, 95% CI: 11.83, 12.28) was also found in BA.5 group than BA.2 group (mean: 15.05, 95% CI: 14.79, 15.31) (Fig. 1g), which were independent of vaccination status (Supplementary Fig. 4). Although there was no difference in peak viral load between BA.5 and BA.2 groups, while unexpectedly, the peak viral load in the unvaccinated participants was lower than the vaccinated participants both in BA.2 and BA.5 groups (Fig. 1h). Shorter hospital stay was also found in BA.5 group (mean: 13.32, 95% CI: 13.07, 13.56) than BA.2 group (mean: 20.20, 95% CI: 19.27, 21.13, respectively) with no significant difference in length of hospital stay among participants with different vaccination status (Fig. 1i and Supplementary Table 3). Further analyses using participants with inactivated vaccine (Supplementary Fig. 5), participants with booster vaccinations and the interval of last vaccination and illness onset below 6 months (Supplementary Fig. 6), as well as local infection cases (Supplementary Fig. 7) showed similar results with Fig. 1f–i.

We also compared the antibody dynamics of BA.2 and BA.5 infection (Supplementary Table 6). The results showed that antibody increased slower and also to a lower level following BA.5 infection, despite of higher rates of vaccination, whereas the viral replication and clearance was faster and shorter when compared with BA.2 (Fig. 1j–l and Supplementary Fig. 8). Similar results were found in the analyses in participants who were boosted within 6 months (Supplementary Fig. 9), indicating the potentially lower immunogenicity and rapid transmission of BA.5 variant. Pearson correlation analyses showed a negative correlation between the IgG antibody levels and the days of viral clearance (*R* = −0.76, *p* < 0.001), while a positive correlation between the IgG antibody levels and the peak viral load (*R* = 0.47, *p* = 0.037) in BA.2 group, while not in BA.5 group (Fig. 1m).

Unlike the results obtained from hamster,^2^ our data indicated that BA.5 infections was relatively milder than BA.2 infections within different status of vaccination, as shown by the lower proportion of moderate disease (5% vs. 17%) and the higher rate of normal CT manifestations (OR 1.53, 1.15–2.04 [*p* = 0.002]). Meanwhile, the reported symptoms of BA.5 infection differed from those of the BA.2 infections, with fever, unusual muscle pains, and pharyngeal discomfort more prevalent in BA.5 infections regardless of vaccination status. Besides, our results also indicated that BA.5 possessed a transmission advantage against BA.2,^3^ as manifested by the faster replication of BA.5 in humans (Fig. 1f), which might contribute to the surge of BA.5 variant. Interestingly, different with BA.2 infections, BA.5 infections induced slower and lower levels of antibody production, and no significant correlation was found between either viral clearance or peak viral load. This might associate a lower immunogenicity but a higher immune evasion of BA.5 variant.^4^ However, a limitation of this study in analyzing the infectious phase is that neutralizing antibodies were not detected, although there is a high correlation between antibody binding and neutralizing capacity,^5^ and the faster and higher IgG responses could also provide higher protection efficiency. In conclusion, our findings undertook a detailed comparison of clinical characteristics, viral and antibody response against BA.2 and BA.5 infections. The results showed that BA.5 is more transmissible with milder symptoms when compared with BA.2, which will be informative in formulating efficient control and treatment strategies for the emerging Omicron subvariants.

## Supplementary information

Supplementary Materials

## Data Availability

The data supporting the findings of this study are available from the corresponding authors upon reasonable request.
