# Supplementary material for: Distinct and relatively mild clinical characteristics of SARS-CoV-2 BA.5 infections against BA.2
Source: Signal Transduct Target Ther. 2023 Apr 26;8:171. doi: 10.1038/s41392-023-01443-2 (PMC10132431; doi:10.1038/s41392-023-01443-2)
Supplement: Supplementary file 1 — Supplementary Materials [file 41392_2023_1443_MOESM1_ESM.doc]

Supplementary Materials for

Distinct and relatively mild clinical characteristics of SARS-CoV-2 BA.5 infections against BA.2

Liping Guo^†^, Xue Liu^†^, Yuchen Gu, Jinyan Jiang, Ziyue Yang, Qiuying Lv, Deyin Guo, Yang Yang^*^, Hongzhou Lu^*^, Jing Yuan^*^

Correspondence to: Jing Yuan, Email: 13500054798@139.com; Hongzhou Lu, Email: luhongzhou@szsy.sustech.edu.cn; Yang Yang, yyszth2018@163.com;

†Contributed equally.

*Contributed equally.

**This PDF file includes:**

Materials and Methods

Figures S1 to S9

Tables S1 to S6

# Methods and Materials

**Patient information and sample collection**

All participants enrolled in this study were hospitalized patients in the Shenzhen Third People's Hospital. General information (including vaccination information), clinical symptoms, laboratory test results (such as CT imaging, Viral Ct values, and IgG Antibody values) of the participants were collected at the earliest time-point after admission. Disease severity classification was evaluated according to China National Health Commission Guidelines for the Diagnosis and Treatment Protocol for Novel Coronavirus Pneumonia (Trial Version 9) of China, that is (1) asymptomatic, defined as no COVID-19 symptoms throughout follow-up; (2) mildly symptomatic, defined as reporting only mild COVID-19 symptoms and no pneumonia was found in imaging during follow-up; and (3) moderately, defined as mild clinical symptoms combined with imaging findings of COVID-19 related pneumonia, such as ground-glass nodules, occurred during the follow-up period. The CT findings of each patient were checked to determine whether the lung lesions were caused by the underlying disease. In this study, pulmonary lesions related to complications caused by COVID-19, such as Fibrous lesions and Lung nodules, were also included in the analysis, but they were not used as the basis for the diagnosis of moderate infections. Each Ct values (N gene) of qRT-PCR and IgG antibody value during hospitalization were collected for future analyses. For mildly and asymptomatic infections, Days post illness onset (d.a.o) was defined as days post laboratory confirmation. Considering the viral loads are different along with the post-exposure/infection time, we enrolled participants who were in the acute infection stage (defined as the lowest Ct value was below 30 during hospitalization) in both groups for viral replication analysis. The study protocol was approved by the Ethics Committees of Shenzhen Third People’s Hospital (2020-010), and written informed consent was obtained.

**Viral Laboratory Confirmation and Antibody Measurement**

Viral RNA was sampled by nasal swab and detected by quantitative real-time PCR (qRT-PCR) targeting ORF1ab and N genes using a novel coronavirus detection kit (BioGerm, Shanghai, China) approved by the China Food and Drug Administration (CFDA). The type of virus variation was determined by whole genome sequencing and epidemiological data. Peak viral load refers to the lowest Ct value of the N gene. Viral clearance was defined as Ct values ≥ 35 for two consecutive qRT-PCR tests (the sampling time interval was at least 24 hours). Discharge criteria were as follows: (1) Body temperature returned to normal for more than 3 days; (2) and respiratory symptoms improved significantly; (3) Pulmonary imaging revealed marked improvement in the acute exudative lesions; (4) Ct values ≥ 35 for two consecutive qRT-PCR tests (the sampling time interval was at least 24 hours). The 2019-nCoV IgG antibody detection kit (Medical & Biological Laboratories Co., Ltd.) based on the chemiluminescence method was used to detect IgG antibodies against the S1 and N proteins of SARS-CoV-2 in serum samples. Antibody values ≥ 10 AU/mL are considered positive.

**Statistical analysis**

In descriptive analyses, data were presented as medians (interquartile range, IQR) for continuous parameters and as frequencies (percentages) for categorical variables. T-test and Wilcoxon rank-sum test were used to compare the continuous variables for normal and non-normal data, respectively. The chi-square test and Fisher exact test were used to compare count data. The 95% CI for CT symptoms and clinical symptoms were calculated as odds ratios using Fisher's exact test. All statistical tests were calculated using GraphPad Prism and R software. A p-value less than 0.05 was considered statistically significant. IgG levels and days for viral clearance were calculated using linear fitting regression as well as Pearson correlation coefficients. What’s more, the kinetics of IgG levels during hospitalization were calculated using LOESS (locally estimated scatter smoothing) curve fitting polynomial regression, version R 4.04.

# 2 Supplementary Figures

**Figure S1.** Flow chart of patient recruitment and disposition in this study


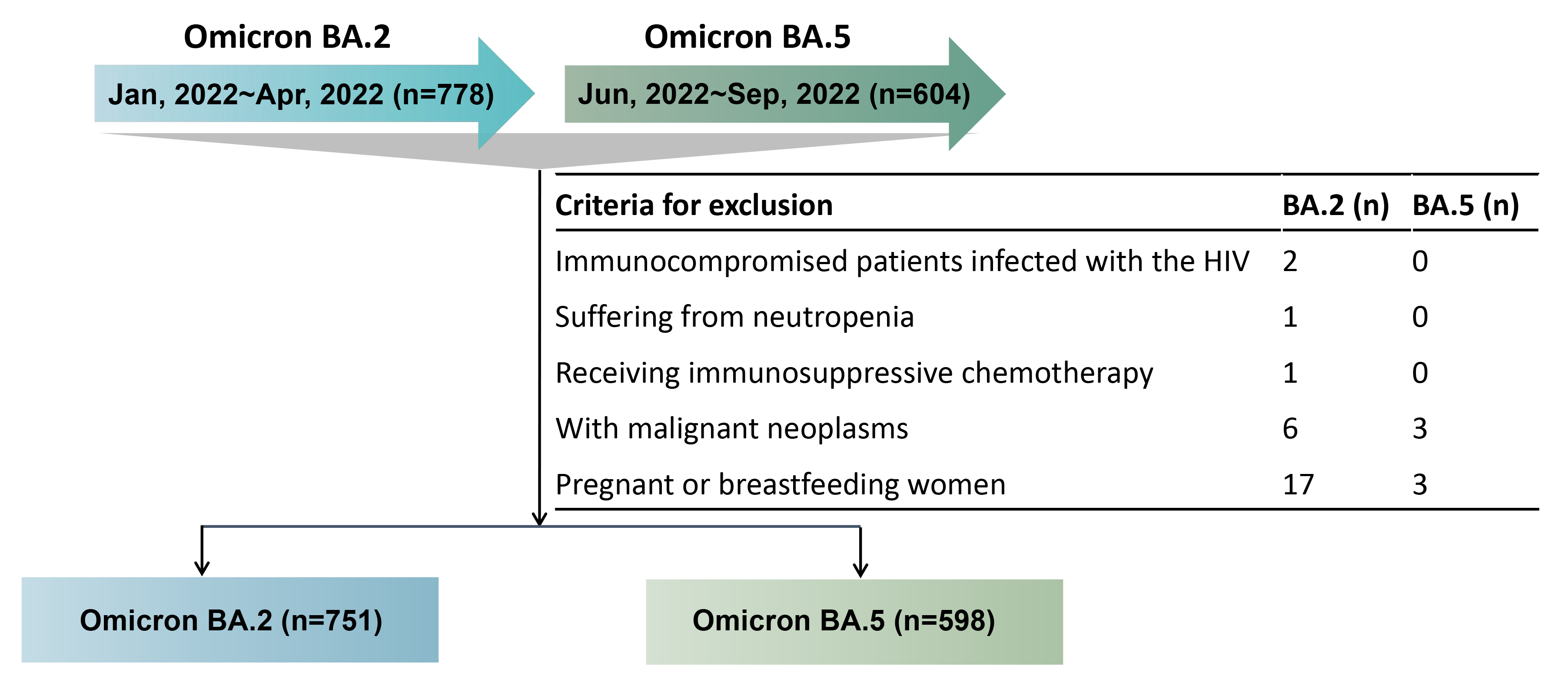


**Figure S2.** Calculation of correlations among the symptoms and CT features for BA.2 (a) and BA.5 (b). Associated symptoms or CT features are circled. The thickness of the lines indicates the strength of the correlation, with red or green lines indicating positive and negative correlations, respectively.


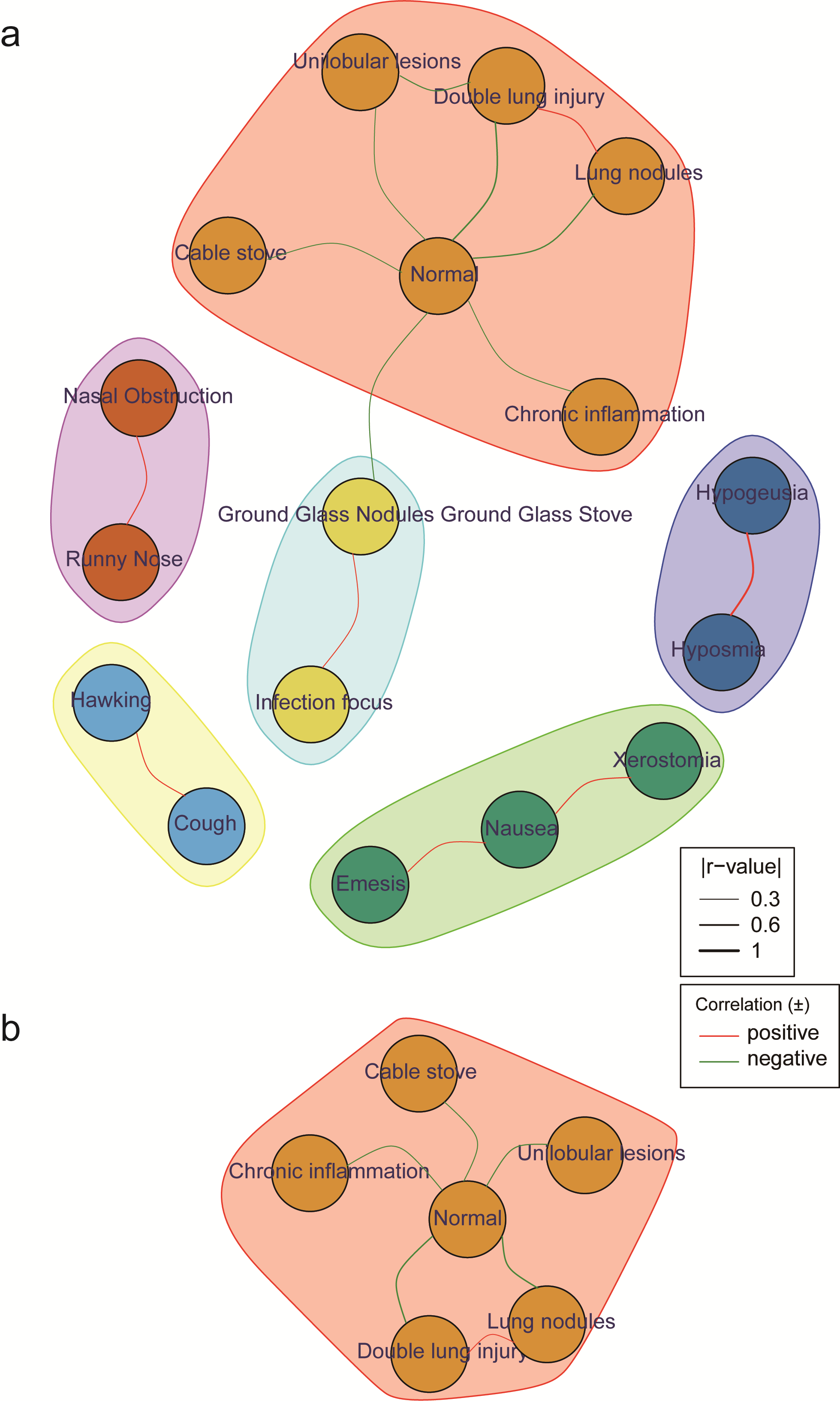


**Figure S3.** Comparison of disease severity, symptoms and CT imaging of BA.2 and BA.5 infections in Unvaccinated (Figure S3a, Figure S3d, Figure S3e), Fully Vaccinated (Figure S3b, Figure S3f, Figure S3g) and Boosted populations (Figure S3c, Figure S3h, Figure S3i) respectively.

**
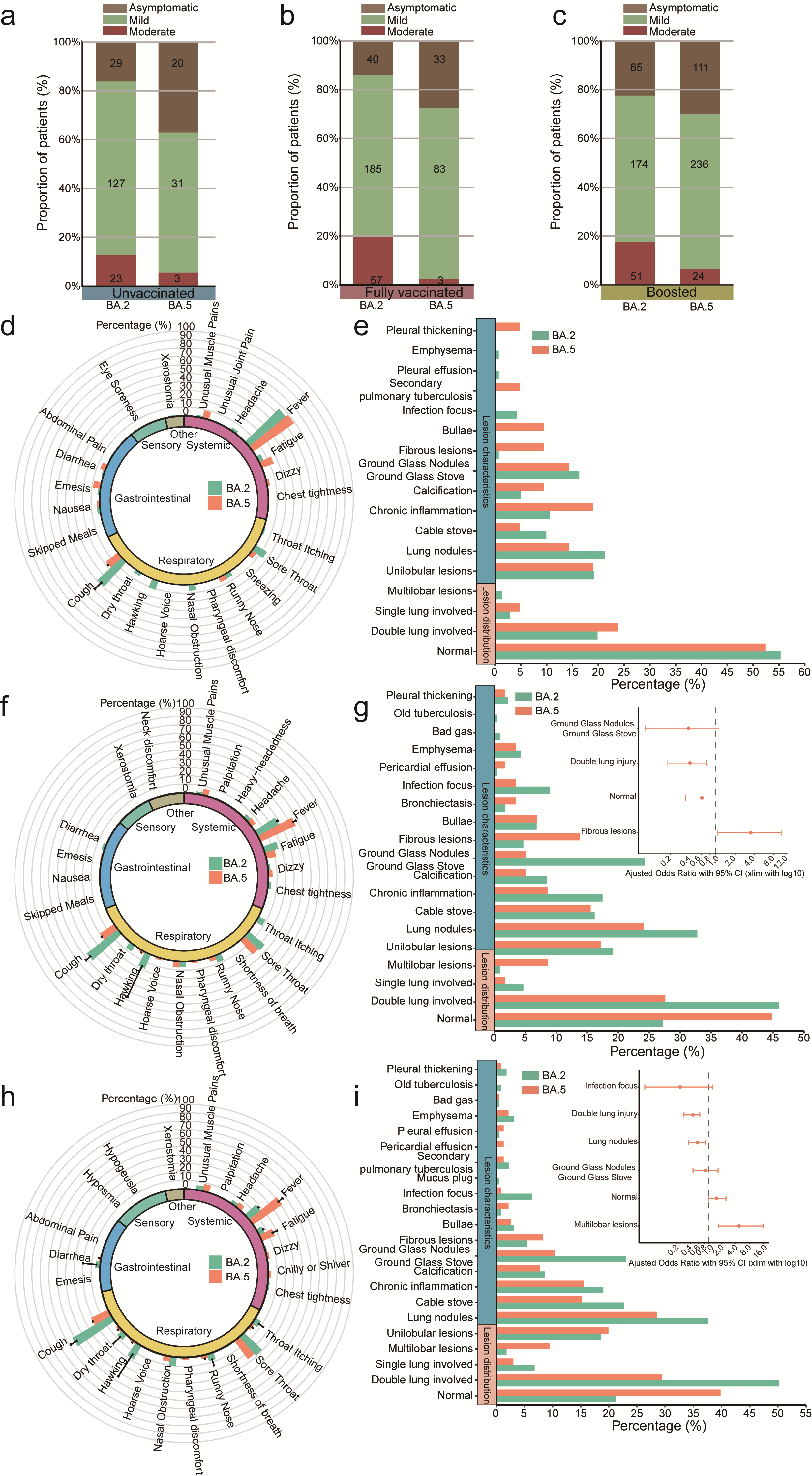
**

**Figure S4.** The days for peak viral load (a) and viral clearance (b) in BA.2 and BA.5 groups. *p < 0.05, **p < 0.01, ***p < 0.001. The line and bar represent the median and interquartile range (IQR) for the corresponding index.


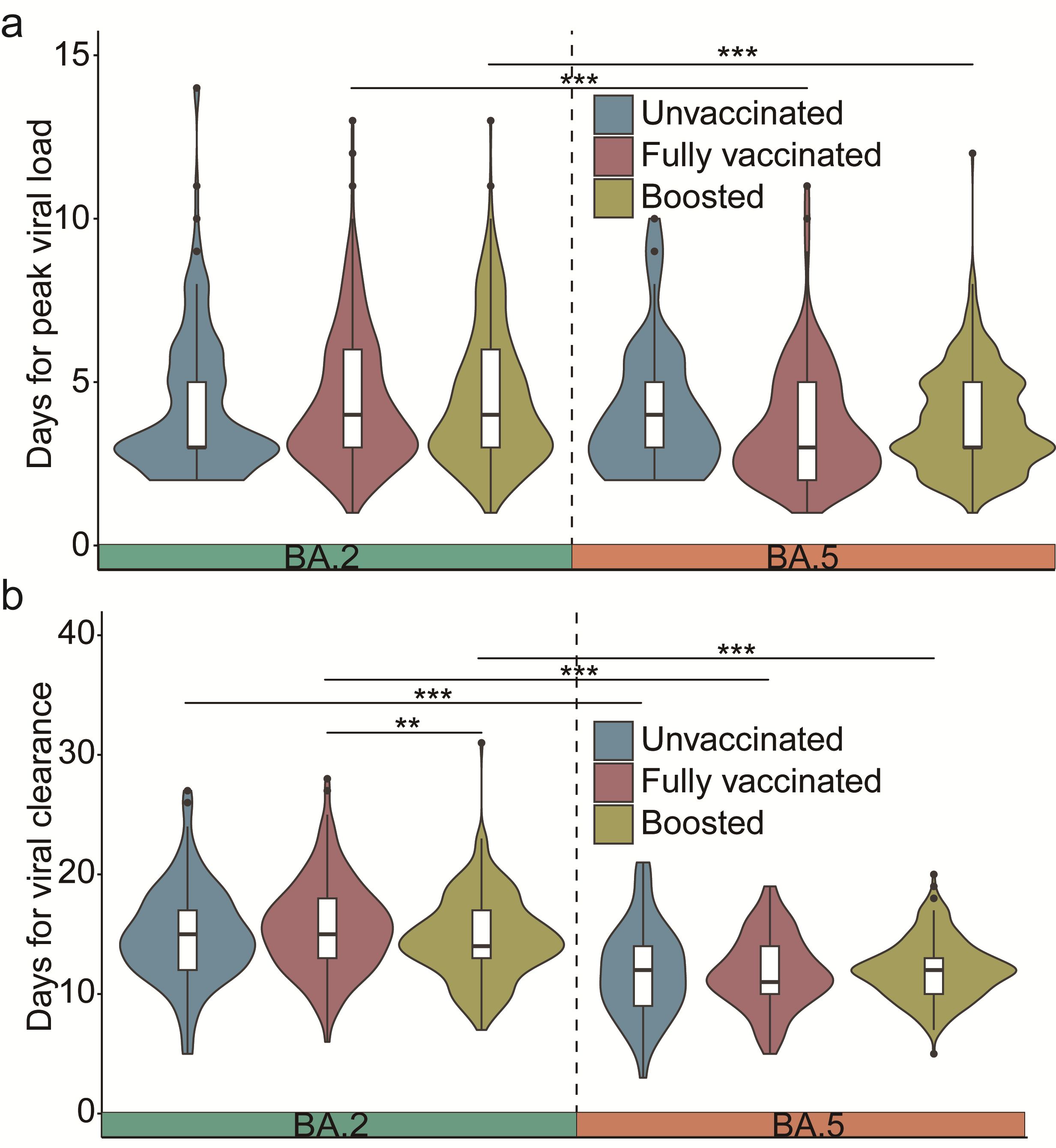


**Figure S5.** Comparison of peak viral load (a) and hospital days (b) between BA.2 and BA.5 infections with inactivated vaccine. *p < 0.05, **p < 0.01, ***p < 0.001. The line and bar represent the median and interquartile range (IQR) for the corresponding index.


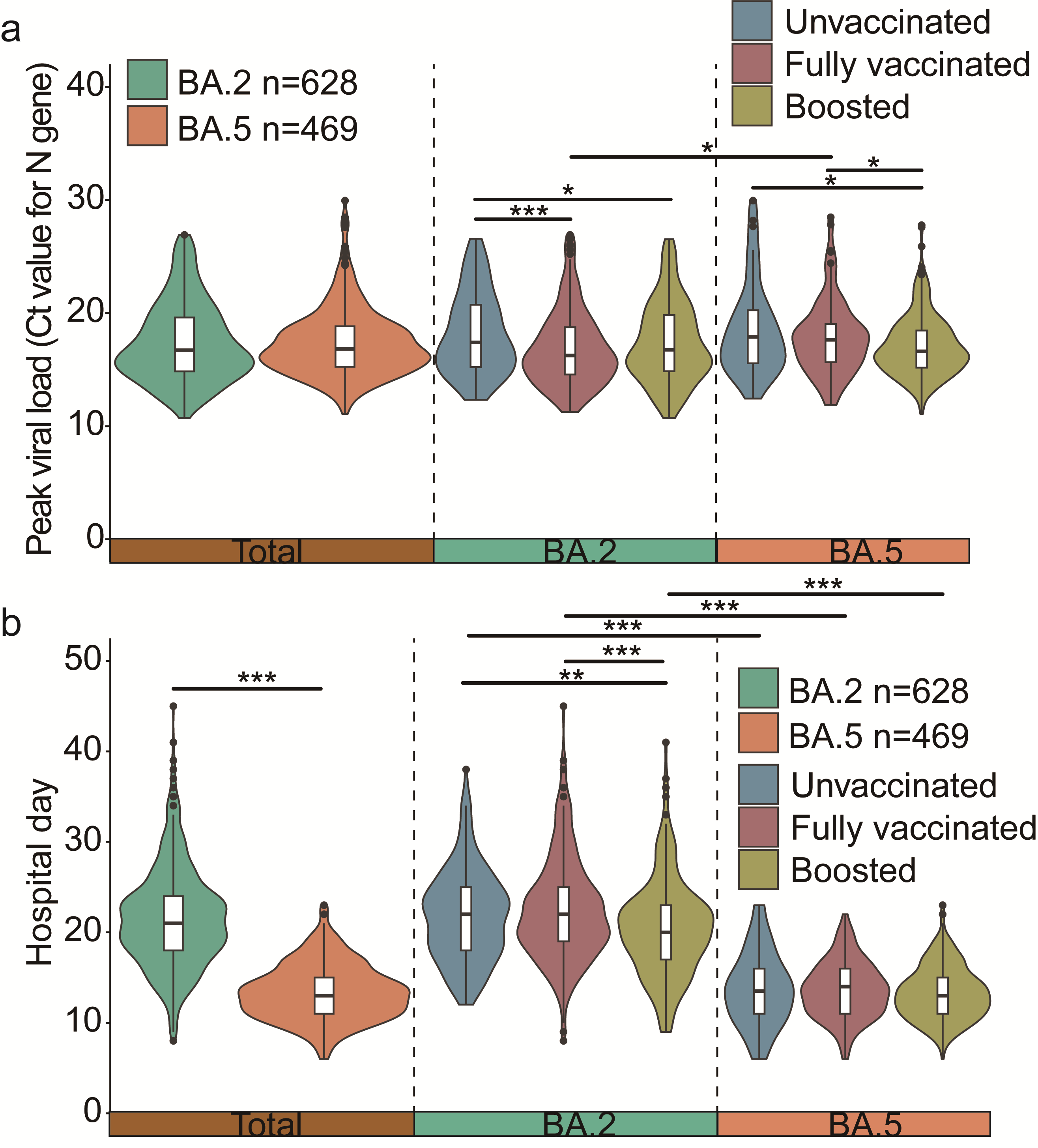


**Figure S6.** Viral replication and duration in participants with booster vaccinations and the interval of last vaccination and illness onset below six months. The peak viral load (a), hospital days (b) and days for viral clearance (c) in BA.2 and BA.5 groups with booster vaccination and the last dose was given less than 6 months before onset. *p < 0.05, **p < 0.01, ***p < 0.001. The line and bar represent the median and interquartile range (IQR) for the corresponding index.


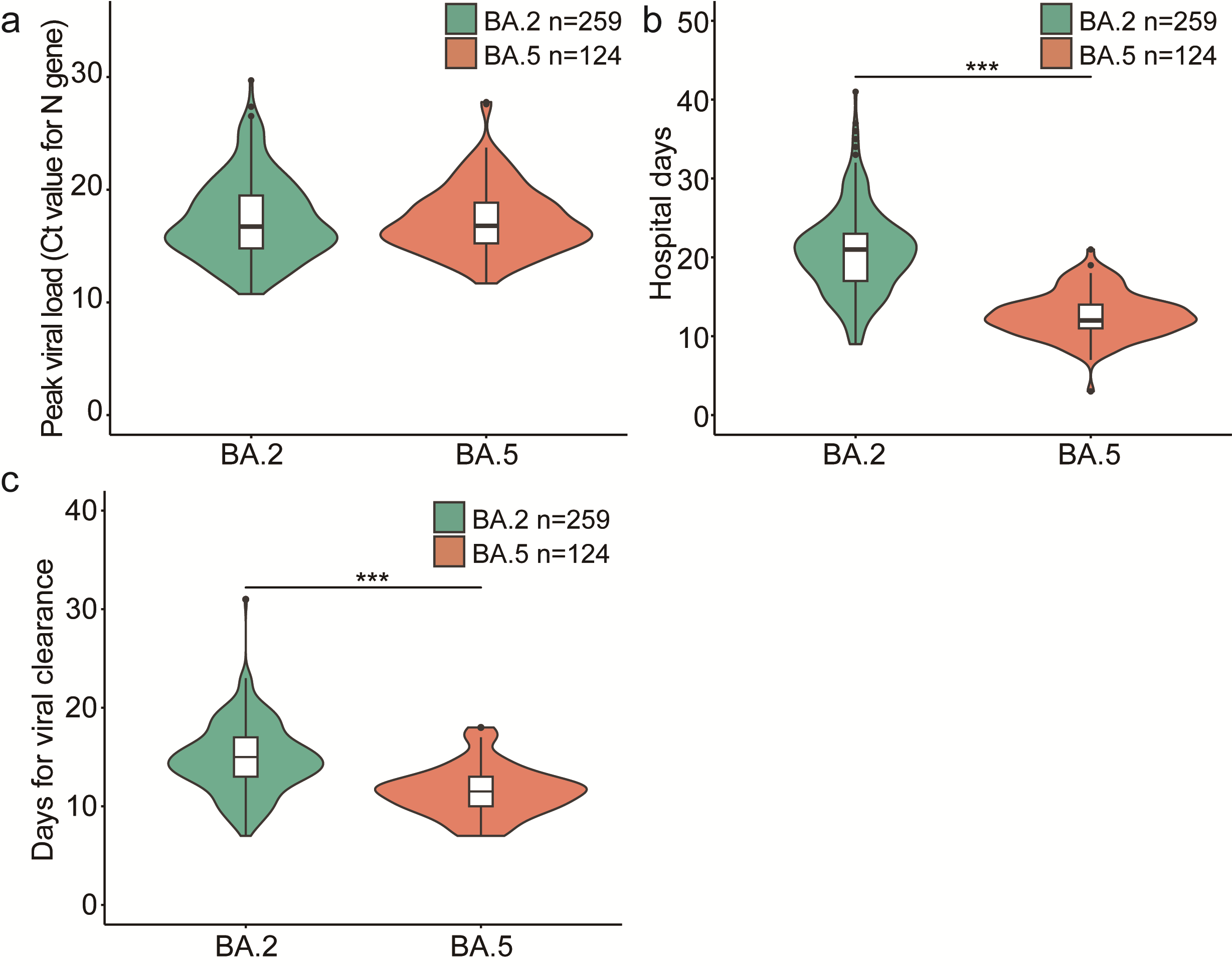


**Figure S7.** Sub-analyses of Viral replication and duration in local infection cases of BA.2 and BA.5 groups. The days for peak viral load (a), viral clearance (b), and hospital days (c) in local infection cases of BA.2 and BA.5 groups. *p < 0.05, **p < 0.01, ***p < 0.001. The line and bar represent the median and interquartile range (IQR) for the corresponding index.


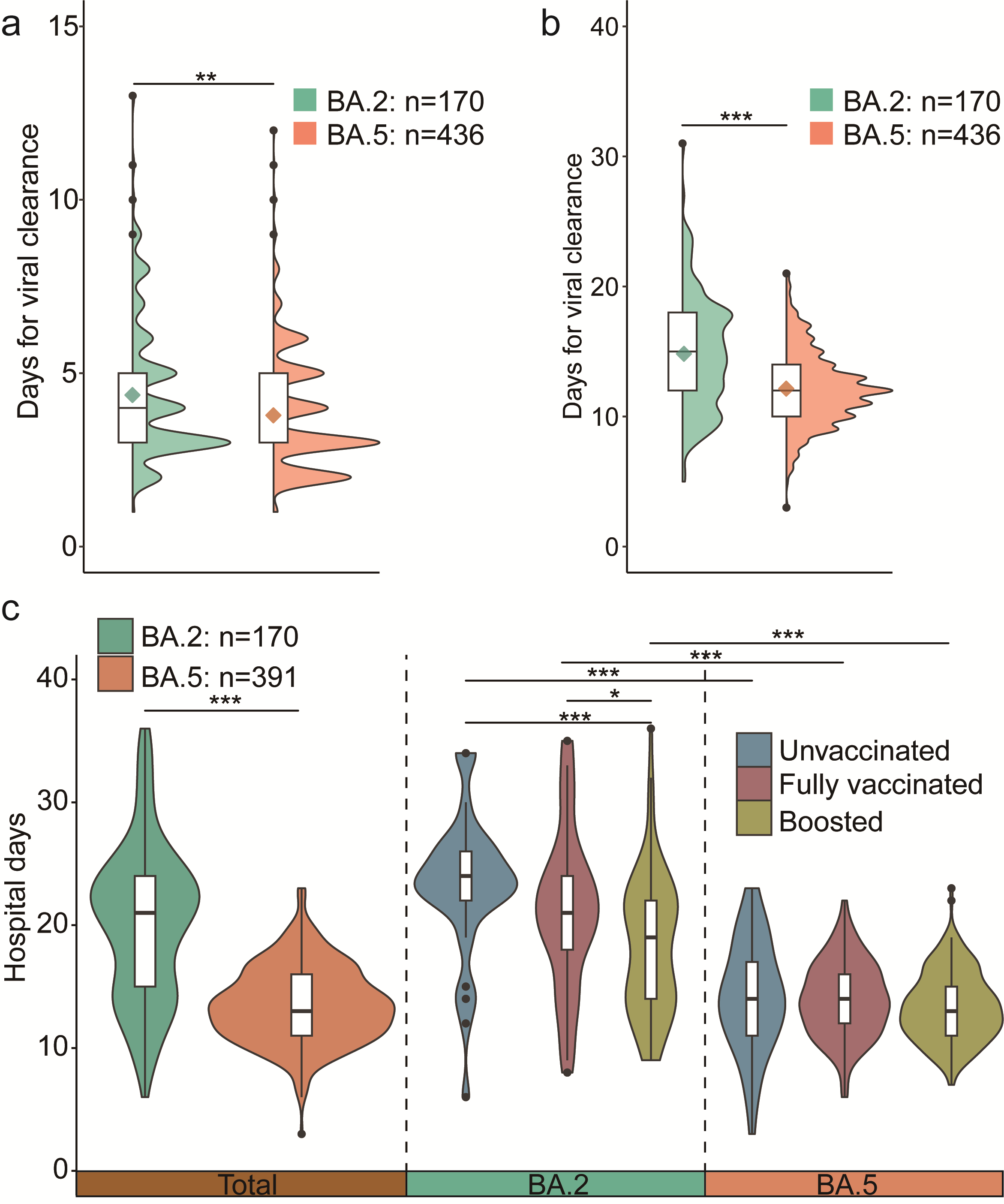


**Figure S8.** Vaccine-based analyses of IgG antibody response in early (within 2 days of infection, a) and middle-late stages (5 days after infection, b). *p < 0.05, **p < 0.01, ***p < 0.001. The line and bar represent the median and interquartile range (IQR) for the corresponding index.


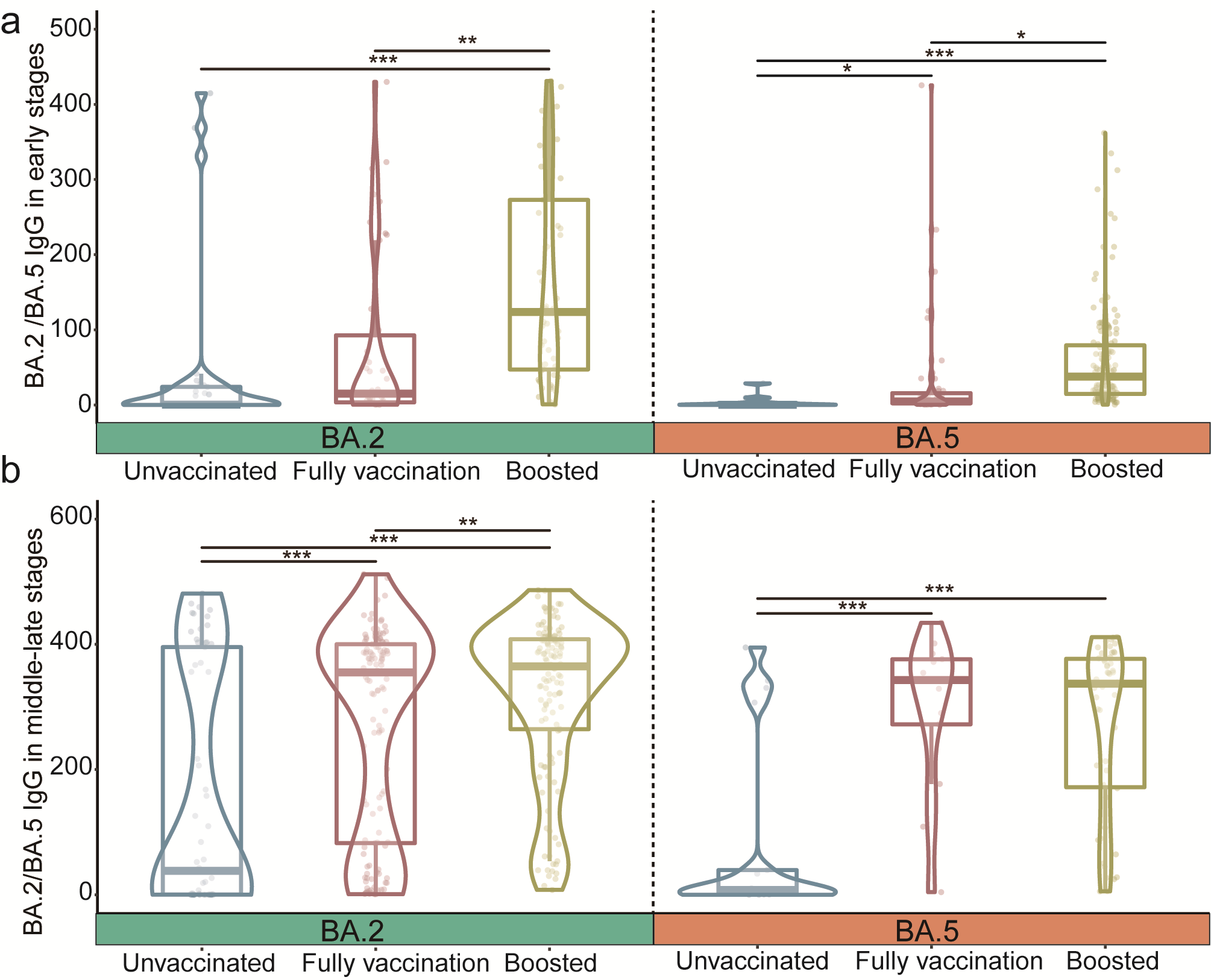


**Figure S9.** Sub-analyses of IgG antibody response in participants with booster vaccinations and the interval of last vaccination and illness onset below six months. a: the antibody dynamics of BA.2 and BA.5 in participants with booster vaccination and the last dose was given less than 6 months before onset. b-c: The IgG antibody response in early (b) and middle-late stages (c) of BA.2 and BA.5 in participants with booster vaccination and the last dose was given less than 6 months before onset.


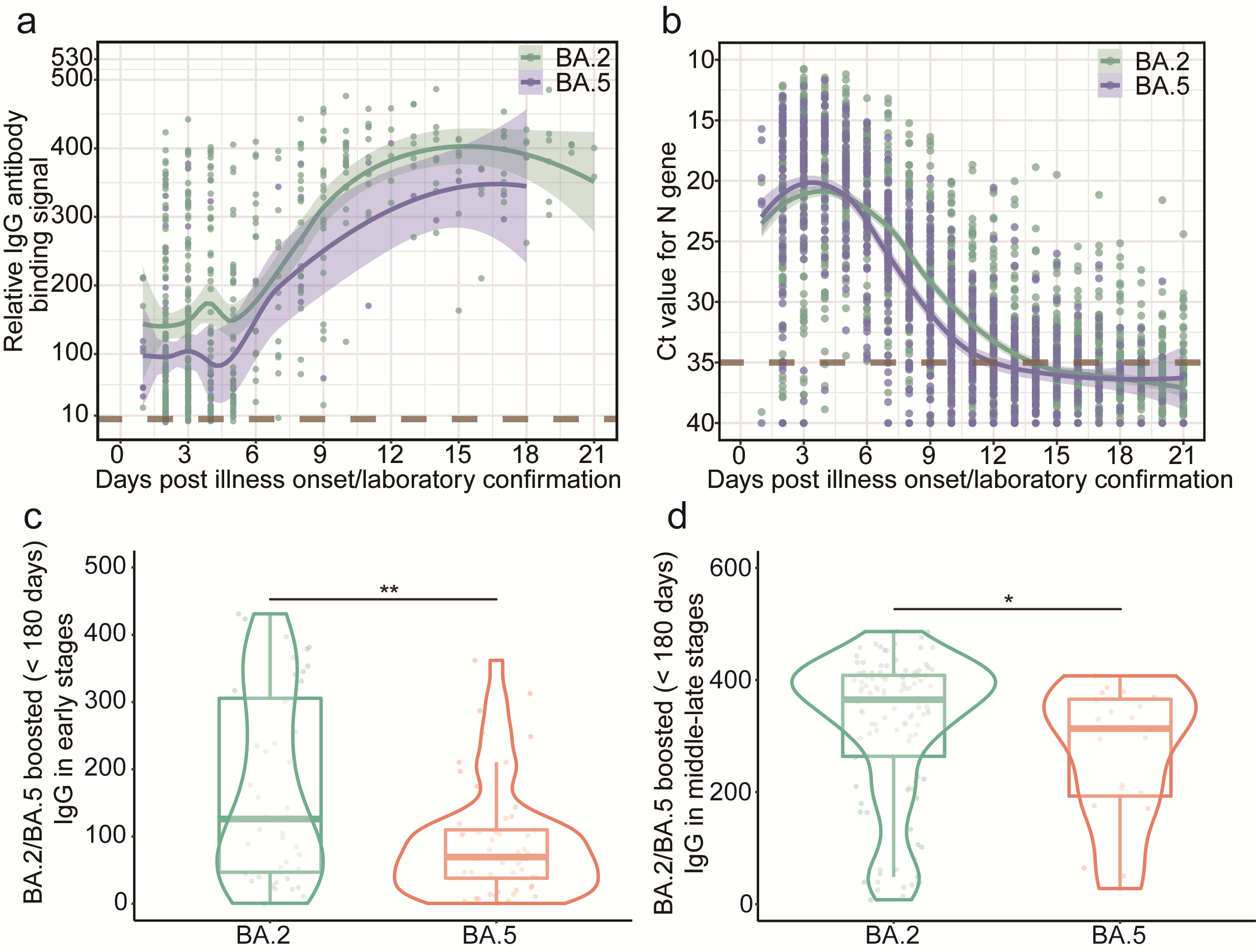


# 3 Supplementary Tables

| Lineages | Type of vaccine | Fully Vaccinated | | Boosted | | |
| --- | --- | --- | --- | --- | --- | --- |
|  |  | First dose | Second dose | First dose | Second dose | Third dose |
| BA.5 | Inactivated vaccines | 83.9% (99/118) | 83.9% (99/118) | 91.64% (340/371) | 91.37% (339/371) | 89.49% (332/371) |
|  | mRNA | 8.47% (10/118) | 8.47% (10/118) | 2.43% (9/371) | 2.7% (10/371) | 4.31% (16/371) |
|  | adenovirus-vectored vaccine | 0% (0/118) | 0% (0/118) | 0.27% (1/371) | 0.27% (1/371) | 0.81% (3/371) |
|  | Unknown | 7.63% (9/118) | 7.63% (9/118) | 5.66% (9/371) | 5.66% (21/371) | 5.39% (20/371) |
| BA.2 | Inactivated vaccines | 81.5% (229/281) | 80.78% (227/281) | 88.28% (256/290) | 88.62% (257/290) | 81.03% (235/290) |
|  | mRNA | 11.03% (31/281) | 11.39% (32/281) | 4.14% (12/290) | 3.79% (11/290) | 11.03% (32/290) |
|  | Recombinant Subunit Vaccines | 0.361% (1/281) | 0.36% (1/281) | 0.34% (1/290) | 0.34% (1/290) | 0.69% (2/290) |
|  | Unknown | 7.12% (20/281) | 7.47% (21/281) | 7.24% (21/290) | 7.24% (21/290) | 7.24% (21/290) |

**Table S1:** Types and proportions of vaccines of BA.2 and BA.5 lineages.

**Table S2. Baseline characteristics of BA.2 and BA.5 infected cases in this study.**

| **Characteristic** | **Omicron** [**variant**](javascript:;)**s infected cases** | | | **P values** | |
| --- | --- | --- | --- | --- | --- |
|  | **Total**  **(N=1349)** | **Omicron BA.2**  **(N=751)** | **Omicron BA.5**  **(N=598)** | **BA.2 vs BA.5** |  |
| **Male (%, n/N)** | 53.97% (728/1349) | 56.59% (425/751) | 50.67% (303/598) | 0.0321 |  |
| **Median age (range)** | 34 (0.08-88) | 33(0.88-88) | 36 (0.23-87) | 0.0122 |  |
| **Age subgroup (%, n/N)** |  |  |  |  |  |
| 0-14 yr | 15.27% (206/1349) | 18.11% (136/751) | 11.88% (70/598) | 0.0013 |  |
| 15--64 yr | 80.65% (1088/1349) | 77.36% (581/751) | 84.78% (507/598) | 0.0013 |  |
| ≥65 yr | 4.08% (55/1349) | 4.53% (34/751) | 3.51% (21/598) | 0.4064 |  |
| **Co-existing chronic medical conditions (%, n/N)** | 15.42% (208/1349) | 16.51% (124/751) | 14.04% (84/598) | 0.2252 |  |
| **Disease severity (%, n/N)** |  |  |  |  |  |
| Asymptomatic | 23.87% (322/1349) | 17.84% (134/751) | 31.43% (188/598) | <0.0001 |  |
| Mild | 64.05% (864/1349) | 64.71% (486/751) | 63.21% (378/598) | 0.5686 |  |
| Moderate | 12.08% (163/1349) | 17.44% (131/751) | 5.35% (32/598) | <0.0001 |  |
| **Vaccination (%, n/N)** |  |  |  |  |  |
| Unvaccinated^*^ | 17.27% (233/1349) | 23.83% (179/751) | 9.03% (54/598) | <0.0001 |  |
| Fully vaccinated | 29.73% (401/1349) | 37.55% (282/751) | 19.90% (119/598) | <0.0001 |  |
| Boosted | 49.00% (661/1349) | 38.62% (290/751) | 62.04% (371/598) | <0.0001 |  |
| Unknown | 4.00% (54/1349) | 0 | 9.03% (54/598) |  |  |
| **Breakthrough infection** | 78.58% (1060/1349) | 76.17% (570/751) | 81.94% (490/598) | 0.0075 |  |
| **Months between infection and the last vaccination (%, n/N)** ^§^ |  |  |  |  |  |
| ≤ 6 months | 49.95% (494/989) | 64.85% (347/535) | 32.37% (147/454) | <0.0001 |  |
| > 6 months | 50.05% (495/989) | 35.14% (188/535) | 67.62% (307/454) | <0.0001 |  |
| **Days between illness onset and hospitalization (Median, IQR)** | 1.239 (1, 2) | 1.282 (1, 2) | 1.186 (1, 2) | 0.02 |  |
| **Ct values at 1** **d.a.o for the acute infection(mean, 95% CI)** | 22.49 (21.80, 23.18) | 23.31 (22.26, 24.36) | 21.77 (20.94, 22.60) | 0.02 |  |

NA: Not available.

†Inter-quartile range.

*Including those who only received the first dose of inactivated vaccine.

§The exact date of the last dose was not available for 38 patients in the BA.2 group.

§The exact date of the last dose was not available for 35 patients in the BA.5 group.

d.a.o: Days post illness onset

| **Table S3.** **Basic** **Information** **of** **BA.2** **and** **BA.5** | | | | | | | | | |
| --- | --- | --- | --- | --- | --- | --- | --- | --- | --- |
| **Variants** | **ID** | **Sex** | **Age** | **Severity** | **Lineages** | **Time** **of** **admission** | **Time** **of** **onset** | **Time** **of** **discharge** | **Hospital** **days** |
| BA.2 | 158689 | female | 35 | Mild | Omicron (BA.2) | 2022/2/27 | 2022/2/26 | 2022/3/ 18 14:49 | 20 |
| BA.2 | 274953 | male | 22 | Asymptomatic | Omicron (BA.2) | 2022/ 1/20 | 2022/ 1/ 19 | 2022/2/5 8:42 | 16 |
| BA.2 | 275062 | male | 39 | Moderate | Omicron (BA.2) | 2022/ 1/22 | 2022/ 1/21 | 2022/2/21 12:39 | 30 |
| BA.2 | 275212 | male | 33 | Mild | Omicron (BA.2) | 2022/ 1/24 | 2022/ 1/22 | 2022/2/ 14 10:57 | 21 |
| BA.2 | 275481 | female | 29 | Mild | Omicron (BA.2) | 2022/ 1/28 | 2022/ 1/27 | 2022/2/ 14 10:58 | 17 |
| BA.2 | 275576 | male | 27 | Mild | Omicron (BA.2) | 2022/ 1/30 | 2022/ 1/26 | 2022/3/ 14 11:18 | 43 |
| BA.2 | 275582 | male | 36 | Mild | Omicron (BA.2) | 2022/ 1/31 | 2022/ 1/30 | 2022/3/6 13:57 | 34 |
| BA.2 | 275583 | female | 26 | Moderate | Omicron (BA.2) | 2022/ 1/31 | 2022/ 1/30 | 2022/2/ 19 12:00 | 19 |
| BA.2 | 275584 | male | 44 | Moderate | Omicron (BA.2) | 2022/ 1/31 | 2022/ 1/30 | 2022/2/ 17 10:00 | 17 |
| BA.2 | 275590 | female | 45 | Moderate | Omicron (BA.2) | 2022/ 1/31 | 2022/ 1/30 | 2022/2/ 18 11:58 | 18 |
| BA.2 | 275595 | female | 47 | Mild | Omicron (BA.2) | 2022/ 1/31 | 2022/ 1/30 | 2022/2/ 18 11:36 | 18 |
| BA.2 | 275596 | female | 31 | Moderate | Omicron (BA.2) | 2022/ 1/31 | 2022/ 1/29 | 2022/2/20 15:57 | 20 |
| BA.2 | 275600 | male | 24 | Mild | Omicron (BA.2) | 2022/2/ 1 | 2022/ 1/31 | 2022/2/ 18 11:19 | 17 |
| BA.2 | 275603 | male | 28 | Moderate | Omicron (BA.2) | 2022/2/ 1 | 2022/ 1/31 | 2022/3/2 16:16 | 29 |
| BA.2 | 275605 | female | 42 | Asymptomatic | Omicron (BA.2) | 2022/2/ 1 | 2022/ 1/31 | 2022/2/23 10:00 | 22 |
| BA.2 | 275612 | female | 55 | Moderate | Omicron (BA.2) | 2022/2/ 1 | 2022/2/ 1 | 2022/2/24 13:39 | 23 |
| BA.2 | 275619 | male | 39 | Asymptomatic | Omicron (BA.2) | 2022/2/2 | 2022/2/ 1 | 2022/2/25 9:37 | 23 |
| BA.2 | 275622 | male | 46 | Moderate | Omicron (BA.2) | 2022/2/2 | 2022/2/ 1 | 2022/2/ 18 11:19 | 16 |
| BA.2 | 275623 | male | 37 | Moderate | Omicron (BA.2) | 2022/2/2 | 2022/2/ 1 | 2022/3/3 14:57 | 29 |
| BA.2 | 275627 | female | 25 | Mild | Omicron (BA.2) | 2022/2/2 | 2022/2/ 1 | 2022/2/21 14:11 | 19 |
| BA.2 | 275631 | female | 43 | Moderate | Omicron (BA.2) | 2022/2/2 | 2022/2/ 1 | 2022/2/22 14:41 | 20 |
| BA.2 | 275643 | male | 72 | Moderate | Omicron (BA.2) | 2022/2/3 | 2022/ 1/31 | 2022/3/3 16:50 | 28 |
| BA.2 | 275650 | female | 43 | Mild | Omicron (BA.2) | 2022/2/3 | 2022/2/2 | 2022/2/25 9:42 | 22 |
| BA.2 | 275660 | female | 40 | Moderate | Omicron (BA.2) | 2022/2/3 | 2022/2/2 | 2022/3/5 13:20 | 30 |
| BA.2 | 275662 | male | 20 | Mild | Omicron (BA.2) | 2022/2/3 | 2022/2/3 | 2022/2/ 18 11:35 | 15 |
| BA.2 | 275674 | male | 44 | Mild | Omicron (BA.2) | 2022/2/4 | 2022/2/3 | 2022/3/6 17:02 | 31 |
| BA.2 | 275676 | male | 31 | Mild | Omicron (BA.2) | 2022/2/4 | 2022/2/2 | 2022/3/ 14 14:25 | 38 |
| BA.2 | 275677 | male | 49 | Mild | Omicron (BA.2) | 2022/2/4 | 2022/2/2 | 2022/3/ 17 21:47 | 42 |
| BA.2 | 275678 | male | 11 | Moderate | Omicron (BA.2) | 2022/2/4 | 2022/2/3 | 2022/2/25 9:39 | 21 |
| BA.2 | 275691 | male | 54 | Asymptomatic | Omicron (BA.2) | 2022/2/4 | 2022/2/4 | 2022/3/ 10 14:30 | 34 |
| BA.2 | 275721 | male | 11 | Mild | Omicron (BA.2) | 2022/2/5 | 2022/2/4 | 2022/3/ 11 21:38 | 34 |
| BA.2 | 275729 | male | 26 | Asymptomatic | Omicron (BA.2) | 2022/2/5 | 2022/2/4 | 2022/3/3 23:53 | 26 |
| BA.2 | 275743 | female | 41 | Moderate | Omicron (BA.2) | 2022/2/5 | 2022/2/3 | 2022/2/24 11:26 | 19 |
| BA.2 | 275767 | female | 68 | Mild | Omicron (BA.2) | 2022/2/6 | 2022/2/3 | 2022/2/20 15:09 | 14 |
| BA.2 | 275799 | female | 20 | Moderate | Omicron (BA.2) | 2022/2/6 | 2022/2/6 | 2022/2/24 11:25 | 18 |
| BA.2 | 275809 | female | 20 | Moderate | Omicron (BA.2) | 2022/2/6 | 2022/2/4 | 2022/3/ 17 22:12 | 39 |

| BA.2 | 275887 | female | 80 | Moderate | Omicron (BA.2) | 2022/2/7 | 2022/2/7 | 2022/3/ 17 16:22 | 38 |
| --- | --- | --- | --- | --- | --- | --- | --- | --- | --- |
| BA.2 | 275910 | male | 65 | Mild | Omicron (BA.2) | 2022/2/7 | 2022/2/7 | 2022/3/2 11:00 | 23 |
| BA.2 | 275921 | female | 42 | Moderate | Omicron (BA.2) | 2022/2/8 | 2022/2/5 | 2022/3/6 10:09 | 26 |
| BA.2 | 275922 | male | 62 | Moderate | Omicron (BA.2) | 2022/2/8 | 2022/2/7 | 2022/3/8 10:46 | 28 |
| BA.2 | 275923 | female | 45 | Mild | Omicron (BA.2) | 2022/2/8 | 2022/2/7 | 2022/3/ 18 20:12 | 39 |
| BA.2 | 275944 | male | 80 | Moderate | Omicron (BA.2) | 2022/2/8 | 2022/2/7 | 2022/3/25 13:33 | 45 |
| BA.2 | 275946 | male | 50 | Moderate | Omicron (BA.2) | 2022/2/8 | 2022/2/7 | 2022/3/ 17 11:48 | 37 |
| BA.2 | 276007 | female | 58 | Mild | Omicron (BA.2) | 2022/2/8 | 2022/2/7 | 2022/3/3 23:54 | 23 |
| BA.2 | 276082 | male | 39 | Mild | Omicron (BA.2) | 2022/2/ 10 | 2022/2/9 | 2022/3/5 11:40 | 23 |
| BA.2 | 276093 | male | 63 | Moderate | Omicron (BA.2) | 2022/2/ 10 | 2022/2/8 | 2022/3/4 11:19 | 22 |
| BA.2 | 276094 | male | 64 | Moderate | Omicron (BA.2) | 2022/2/ 10 | 2022/2/9 | 2022/3/ 11 12:50 | 29 |
| BA.2 | 276103 | female | 45 | Mild | Omicron (BA.2) | 2022/2/ 10 | 2022/2/7 | 2022/3/ 11 9:01 | 29 |
| BA.2 | 276145 | female | 69 | Mild | Omicron (BA.2) | 2022/2/ 10 | 2022/2/8 | 2022/3/ 15 8:43 | 32 |
| BA.2 | 276161 | male | 1 | Moderate | Omicron (BA.2) | 2022/2/ 11 | 2022/2/ 10 | 2022/3/2 10:02 | 19 |
| BA.2 | 276162 | male | 57 | Moderate | Omicron (BA.2) | 2022/2/ 11 | 2022/2/9 | 2022/3/ 17 14:12 | 34 |
| BA.2 | 276163 | male | 27 | Asymptomatic | Omicron (BA.2) | 2022/2/ 11 | 2022/2/ 10 | 2022/3/ 14 10:43 | 31 |
| BA.2 | 276164 | female | 27 | Mild | Omicron (BA.2) | 2022/2/ 11 | 2022/2/9 | 2022/3/ 14 10:54 | 31 |
| BA.2 | 276170 | female | 56 | Mild | Omicron (BA.2) | 2022/2/ 11 | 2022/2/ 10 | 2022/3/ 13 15:05 | 30 |
| BA.2 | 276171 | male | 2 | Mild | Omicron (BA.2) | 2022/2/ 11 | 2022/2/ 10 | 2022/3/8 9:38 | 25 |
| BA.2 | 276172 | female | 52 | Mild | Omicron (BA.2) | 2022/2/ 11 | 2022/2/ 10 | 2022/3/ 17 11:25 | 34 |
| BA.2 | 276179 | female | 51 | Moderate | Omicron (BA.2) | 2022/2/ 11 | 2022/2/4 | 2022/3/ 14 11:18 | 31 |
| BA.2 | 276186 | male | 52 | Moderate | Omicron (BA.2) | 2022/2/ 11 | 2022/2/ 10 | 2022/3/ 17 9:04 | 34 |
| BA.2 | 276189 | male | 51 | Mild | Omicron (BA.2) | 2022/2/ 11 | 2022/2/ 10 | 2022/3/ 12 0:48 | 28 |
| BA.2 | 276192 | male | 20 | Mild | Omicron (BA.2) | 2022/2/ 11 | 2022/2/9 | 2022/3/6 12:59 | 23 |
| BA.2 | 276232 | male | 2 | Mild | Omicron (BA.2) | 2022/2/ 11 | 2022/2/ 11 | 2022/3/7 15:27 | 24 |
| BA.2 | 276233 | female | 3 | Mild | Omicron (BA.2) | 2022/2/ 11 | 2022/2/ 11 | 2022/3/7 13:22 | 24 |
| BA.2 | 276235 | female | 29 | Asymptomatic | Omicron (BA.2) | 2022/2/ 11 | 2022/2/ 11 | 2022/3/7 13:22 | 24 |
| BA.2 | 276241 | male | 26 | Mild | Omicron (BA.2) | 2022/2/ 11 | 2022/2/ 10 | 2022/3/ 10 13:57 | 27 |
| BA.2 | 276243 | male | 34 | Mild | Omicron (BA.2) | 2022/2/ 12 | 2022/2/ 11 | 2022/3/ 17 20:00 | 34 |
| BA.2 | 276247 | male | 38 | Mild | Omicron (BA.2) | 2022/2/ 12 | 2022/2/ 11 | 2022/3/6 10:15 | 22 |
| BA.2 | 276248 | female | 62 | Mild | Omicron (BA.2) | 2022/2/ 12 | 2022/2/ 10 | 2022/3/20 11:53 | 36 |
| BA.2 | 276249 | male | 17 | Mild | Omicron (BA.2) | 2022/2/ 12 | 2022/2/ 11 | 2022/3/ 17 11:24 | 33 |
| BA.2 | 276250 | male | 49 | Mild | Omicron (BA.2) | 2022/2/ 12 | 2022/2/8 | 2022/3/ 15 10:45 | 31 |
| BA.2 | 276251 | female | 9 | Mild | Omicron (BA.2) | 2022/2/ 12 | 2022/2/ 12 | 2022/3/8 9:30 | 24 |
| BA.2 | 276252 | male | 2 | Mild | Omicron (BA.2) | 2022/2/ 12 | 2022/2/ 11 | 2022/3/7 13:21 | 23 |
| BA.2 | 276253 | female | 26 | Mild | Omicron (BA.2) | 2022/2/ 12 | 2022/2/ 11 | 2022/3/7 13:22 | 23 |
| BA.2 | 276312 | female | 59 | Mild | Omicron (BA.2) | 2022/2/ 12 | 2022/2/ 10 | 2022/3/ 17 16:30 | 33 |
| BA.2 | 276313 | female | 38 | Mild | Omicron (BA.2) | 2022/2/ 12 | 2022/2/ 11 | 2022/3/ 15 10:51 | 31 |

| BA.2 | 276314 | female | 33 | Mild | Omicron (BA.2) | 2022/2/ 12 | 2022/2/ 11 | 2022/3/ 15 13:44 | 31 |
| --- | --- | --- | --- | --- | --- | --- | --- | --- | --- |
| BA.2 | 276316 | female | 28 | Mild | Omicron (BA.2) | 2022/2/ 12 | 2022/2/ 11 | 2022/3/ 17 20:33 | 33 |
| BA.2 | 276319 | male | 47 | Mild | Omicron (BA.2) | 2022/2/ 12 | 2022/2/ 10 | 2022/3/ 12 13:43 | 28 |
| BA.2 | 276325 | male | 51 | Moderate | Omicron (BA.2) | 2022/2/ 12 | 2022/2/ 12 | 2022/3/4 18:30 | 20 |
| BA.2 | 276326 | male | 54 | Moderate | Omicron (BA.2) | 2022/2/ 12 | 2022/2/ 11 | 2022/3/ 17 16:22 | 33 |
| BA.2 | 276327 | male | 63 | Mild | Omicron (BA.2) | 2022/2/ 13 | 2022/2/ 12 | 2022/3/ 17 20:00 | 33 |
| BA.2 | 276328 | female | 36 | Mild | Omicron (BA.2) | 2022/2/ 13 | 2022/2/ 12 | 2022/3/ 15 16:17 | 31 |
| BA.2 | 276349 | female | 46 | Mild | Omicron (BA.2) | 2022/2/ 13 | 2022/2/ 10 | 2022/3/ 15 10:44 | 30 |
| BA.2 | 276369 | male | 57 | Mild | Omicron (BA.2) | 2022/2/ 13 | 2022/2/ 13 | 2022/3/ 17 17:30 | 32 |
| BA.2 | 276373 | male | 6 | Mild | Omicron (BA.2) | 2022/2/ 13 | 2022/2/ 11 | 2022/3/3 14:15 | 18 |
| BA.2 | 276405 | female | 25 | Mild | Omicron (BA.2) | 2022/2/ 13 | 2022/2/ 11 | 2022/3/ 10 9:56 | 25 |
| BA.2 | 276409 | female | 35 | Mild | Omicron (BA.2) | 2022/2/ 14 | 2022/2/ 13 | 2022/3/ 17 21:23 | 32 |
| BA.2 | 276410 | female | 23 | Mild | Omicron (BA.2) | 2022/2/ 14 | 2022/2/ 11 | 2022/3/ 15 10:57 | 29 |
| BA.2 | 276442 | male | 8 | Mild | Omicron (BA.2) | 2022/2/ 14 | 2022/2/ 13 | 2022/3/ 17 16:30 | 31 |
| BA.2 | 276444 | female | 20 | Mild | Omicron (BA.2) | 2022/2/ 14 | 2022/2/ 12 | 2022/3/ 15 10:49 | 29 |
| BA.2 | 276457 | male | 72 | Moderate | Omicron (BA.2) | 2022/2/ 14 | 2022/2/ 12 | 2022/3/ 17 17:00 | 31 |
| BA.2 | 276459 | female | 13 | Mild | Omicron (BA.2) | 2022/2/ 14 | 2022/2/ 11 | 2022/3/ 17 20:48 | 31 |
| BA.2 | 276460 | female | 35 | Mild | Omicron (BA.2) | 2022/2/ 14 | 2022/2/ 12 | 2022/3/ 16 14:00 | 30 |
| BA.2 | 276461 | female | 27 | Mild | Omicron (BA.2) | 2022/2/ 14 | 2022/2/ 12 | 2022/3/ 12 13:25 | 26 |
| BA.2 | 276471 | male | 33 | Moderate | Omicron (BA.2) | 2022/2/ 14 | 2022/2/ 13 | 2022/3/ 13 12:16 | 27 |
| BA.2 | 276472 | male | 27 | Mild | Omicron (BA.2) | 2022/2/ 14 | 2022/2/ 12 | 2022/3/ 13 11:20 | 27 |
| BA.2 | 276498 | male | 51 | Mild | Omicron (BA.2) | 2022/2/ 14 | 2022/2/ 13 | 2022/3/4 0:35 | 17 |
| BA.2 | 276503 | male | 30 | Mild | Omicron (BA.2) | 2022/2/ 14 | 2022/2/ 13 | 2022/3/ 13 12:08 | 27 |
| BA.2 | 276509 | female | 45 | Moderate | Omicron (BA.2) | 2022/2/ 15 | 2022/2/ 13 | 2022/3/ 13 9:18 | 26 |
| BA.2 | 276530 | male | 23 | Mild | Omicron (BA.2) | 2022/2/ 15 | 2022/2/ 14 | 2022/3/22 15:30 | 35 |
| BA.2 | 276531 | male | 17 | Mild | Omicron (BA.2) | 2022/2/ 15 | 2022/2/ 14 | 2022/3/7 12:00 | 20 |
| BA.2 | 276542 | male | 57 | Moderate | Omicron (BA.2) | 2022/2/ 15 | 2022/2/ 13 | 2022/3/ 17 11:22 | 30 |
| BA.2 | 276553 | female | 54 | Mild | Omicron (BA.2) | 2022/2/ 15 | 2022/2/ 14 | 2022/3/ 17 21:00 | 30 |
| BA.2 | 276570 | female | 45 | Mild | Omicron (BA.2) | 2022/2/ 15 | 2022/2/ 14 | 2022/3/ 14 12:00 | 27 |
| BA.2 | 276571 | male | 47 | Mild | Omicron (BA.2) | 2022/2/ 15 | 2022/2/ 14 | 2022/3/ 12 13:24 | 25 |
| BA.2 | 276577 | male | 48 | Mild | Omicron (BA.2) | 2022/2/ 15 | 2022/2/ 14 | 2022/3/21 10:00 | 34 |
| BA.2 | 276578 | male | 47 | Mild | Omicron (BA.2) | 2022/2/ 15 | 2022/2/ 11 | 2022/3/21 10:00 | 34 |
| BA.2 | 276587 | male | 52 | Asymptomatic | Omicron (BA.2) | 2022/2/ 16 | 2022/2/ 15 | 2022/3/ 15 11:23 | 27 |
| BA.2 | 276590 | male | 66 | Asymptomatic | Omicron (BA.2) | 2022/2/ 16 | 2022/2/ 15 | 2022/3/ 18 13:03 | 30 |
| BA.2 | 276599 | male | 46 | Moderate | Omicron (BA.2) | 2022/2/ 16 | 2022/2/ 14 | 2022/3/ 12 13:23 | 24 |
| BA.2 | 276600 | male | 38 | Moderate | Omicron (BA.2) | 2022/2/ 16 | 2022/2/ 14 | 2022/3/ 17 14:21 | 29 |
| BA.2 | 276601 | male | 39 | Mild | Omicron (BA.2) | 2022/2/ 16 | 2022/2/ 15 | 2022/3/ 17 19:09 | 29 |
| BA.2 | 276617 | female | 61 | Moderate | Omicron (BA.2) | 2022/2/ 16 | 2022/2/ 15 | 2022/3/ 17 16:00 | 29 |

| BA.2 | 276618 | female | 60 | Mild | Omicron (BA.2) | 2022/2/ 16 | 2022/2/ 15 | 2022/3/ 13 10:47 | 25 |
| --- | --- | --- | --- | --- | --- | --- | --- | --- | --- |
| BA.2 | 276632 | male | 70 | Moderate | Omicron (BA.2) | 2022/2/ 16 | 2022/2/ 15 | 2022/3/ 18 13:03 | 30 |
| BA.2 | 276634 | male | 29 | Mild | Omicron (BA.2) | 2022/2/ 16 | 2022/2/ 13 | 2022/3/ 17 21:36 | 29 |
| BA.2 | 276640 | male | 43 | Mild | Omicron (BA.2) | 2022/2/ 16 | 2022/2/ 15 | 2022/3/ 12 8:22 | 24 |
| BA.2 | 276658 | male | 26 | Mild | Omicron (BA.2) | 2022/2/ 16 | 2022/2/ 15 | 2022/3/ 15 13:41 | 27 |
| BA.2 | 276662 | male | 21 | Mild | Omicron (BA.2) | 2022/2/ 16 | 2022/2/ 15 | 2022/3/ 17 22:53 | 29 |
| BA.2 | 276671 | female | 37 | Mild | Omicron (BA.2) | 2022/2/ 16 | 2022/2/ 14 | 2022/3/ 14 9:59 | 26 |
| BA.2 | 276679 | male | 64 | Mild | Omicron (BA.2) | 2022/2/ 17 | 2022/2/ 16 | 2022/3/ 19 10:10 | 30 |
| BA.2 | 276717 | male | 30 | Mild | Omicron (BA.2) | 2022/2/ 17 | 2022/2/ 14 | 2022/3/ 17 18:54 | 28 |
| BA.2 | 276747 | male | 32 | Moderate | Omicron (BA.2) | 2022/2/ 17 | 2022/2/ 16 | 2022/3/ 13 11:21 | 24 |
| BA.2 | 276748 | male | 42 | Mild | Omicron (BA.2) | 2022/2/ 17 | 2022/2/ 17 | 2022/3/4 13:35 | 15 |
| BA.2 | 276756 | female | 23 | Mild | Omicron (BA.2) | 2022/2/ 17 | 2022/2/ 15 | 2022/3/ 15 10:56 | 26 |
| BA.2 | 276770 | male | 66 | Mild | Omicron (BA.2) | 2022/2/ 17 | 2022/2/ 15 | 2022/3/24 13:10 | 35 |
| BA.2 | 276772 | male | 15 | Mild | Omicron (BA.2) | 2022/2/ 17 | 2022/2/ 17 | 2022/3/ 17 17:55 | 28 |
| BA.2 | 276778 | male | 23 | Mild | Omicron (BA.2) | 2022/2/ 18 | 2022/2/ 16 | 2022/3/3 14:56 | 14 |
| BA.2 | 276779 | male | 56 | Moderate | Omicron (BA.2) | 2022/2/ 18 | 2022/2/ 15 | 2022/3/ 10 18:17 | 21 |
| BA.2 | 276780 | male | 24 | Mild | Omicron (BA.2) | 2022/2/ 18 | 2022/2/ 17 | 2022/3/ 16 18:33 | 27 |
| BA.2 | 276783 | male | 55 | Moderate | Omicron (BA.2) | 2022/2/ 18 | 2022/2/ 17 | 2022/3/ 17 21:46 | 28 |
| BA.2 | 276788 | male | 40 | Mild | Omicron (BA.2) | 2022/2/ 18 | 2022/2/ 17 | 2022/3/5 12:18 | 15 |
| BA.2 | 276789 | female | 3 | Mild | Omicron (BA.2) | 2022/2/ 18 | 2022/2/ 16 | 2022/3/ 16 15:54 | 26 |
| BA.2 | 276790 | male | 41 | Moderate | Omicron (BA.2) | 2022/2/ 18 | 2022/2/ 17 | 2022/3/ 17 9:49 | 27 |
| BA.2 | 276793 | female | 53 | Mild | Omicron (BA.2) | 2022/2/ 18 | 2022/2/ 17 | 2022/3/ 18 13:01 | 28 |
| BA.2 | 276801 | female | 34 | Mild | Omicron (BA.2) | 2022/2/ 18 | 2022/2/ 15 | 2022/3/ 15 11:24 | 25 |
| BA.2 | 276802 | female | 25 | Mild | Omicron (BA.2) | 2022/2/ 18 | 2022/2/ 17 | 2022/3/ 18 20:30 | 28 |
| BA.2 | 276803 | female | 47 | Mild | Omicron (BA.2) | 2022/2/ 18 | 2022/2/ 17 | 2022/3/ 18 13:05 | 28 |
| BA.2 | 276818 | female | 15 | Mild | Omicron (BA.2) | 2022/2/ 18 | 2022/2/ 15 | 2022/3/23 17:01 | 33 |
| BA.2 | 276819 | male | 68 | Moderate | Omicron (BA.2) | 2022/2/ 18 | 2022/2/ 17 | 2022/3/6 10:10 | 16 |
| BA.2 | 276821 | male | 42 | Moderate | Omicron (BA.2) | 2022/2/ 18 | 2022/2/ 17 | 2022/3/21 14:37 | 31 |
| BA.2 | 276826 | male | 5 | Mild | Omicron (BA.2) | 2022/2/ 18 | 2022/2/ 17 | 2022/3/21 13:00 | 31 |
| BA.2 | 276849 | male | 47 | Mild | Omicron (BA.2) | 2022/2/ 18 | 2022/2/ 18 | 2022/3/ 18 14:28 | 28 |
| BA.2 | 276853 | female | 45 | Mild | Omicron (BA.2) | 2022/2/ 18 | 2022/2/ 17 | 2022/3/6 16:46 | 16 |
| BA.2 | 276855 | female | 51 | Moderate | Omicron (BA.2) | 2022/2/ 18 | 2022/2/ 17 | 2022/3/ 19 18:33 | 29 |
| BA.2 | 276857 | female | 7 | Mild | Omicron (BA.2) | 2022/2/ 18 | 2022/2/ 17 | 2022/3/ 18 13:06 | 28 |
| BA.2 | 276858 | female | 35 | Mild | Omicron (BA.2) | 2022/2/ 18 | 2022/2/ 17 | 2022/3/ 14 17:05 | 24 |
| BA.2 | 276859 | female | 9 | Mild | Omicron (BA.2) | 2022/2/ 18 | 2022/2/ 17 | 2022/3/ 18 13:01 | 28 |
| BA.2 | 276860 | male | 40 | Moderate | Omicron (BA.2) | 2022/2/ 18 | 2022/2/ 17 | 2022/3/21 13:28 | 31 |
| BA.2 | 276861 | male | 49 | Mild | Omicron (BA.2) | 2022/2/ 18 | 2022/2/ 16 | 2022/3/ 15 11:23 | 24 |
| BA.2 | 276862 | male | 55 | Moderate | Omicron (BA.2) | 2022/2/ 18 | 2022/2/ 18 | 2022/3/ 15 11:26 | 25 |

| BA.2 | 276864 | female | 31 | Moderate | Omicron (BA.2) | 2022/2/ 18 | 2022/2/ 17 | 2022/3/ 18 13:01 | 28 |
| --- | --- | --- | --- | --- | --- | --- | --- | --- | --- |
| BA.2 | 276867 | male | 33 | Mild | Omicron (BA.2) | 2022/2/ 19 | 2022/2/ 17 | 2022/3/ 18 13:03 | 27 |
| BA.2 | 276868 | female | 31 | Mild | Omicron (BA.2) | 2022/2/ 19 | 2022/2/ 18 | 2022/3/ 16 18:18 | 26 |
| BA.2 | 276869 | male | 42 | Moderate | Omicron (BA.2) | 2022/2/ 19 | 2022/2/ 18 | 2022/3/ 18 13:02 | 27 |
| BA.2 | 276870 | male | 34 | Mild | Omicron (BA.2) | 2022/2/ 19 | 2022/2/ 17 | 2022/3/9 14:30 | 19 |
| BA.2 | 276871 | male | 58 | Moderate | Omicron (BA.2) | 2022/2/ 19 | 2022/2/ 18 | 2022/3/ 11 14:56 | 21 |
| BA.2 | 276872 | male | 33 | Mild | Omicron (BA.2) | 2022/2/ 19 | 2022/2/ 17 | 2022/3/ 10 17:49 | 20 |
| BA.2 | 276873 | male | 49 | Mild | Omicron (BA.2) | 2022/2/ 19 | 2022/2/ 17 | 2022/3/ 18 13:04 | 27 |
| BA.2 | 276874 | male | 44 | Asymptomatic | Omicron (BA.2) | 2022/2/ 19 | 2022/2/ 18 | 2022/3/ 18 13:02 | 27 |
| BA.2 | 276876 | male | 26 | Mild | Omicron (BA.2) | 2022/2/ 19 | 2022/2/ 18 | 2022/3/ 18 13:03 | 27 |
| BA.2 | 276879 | male | 57 | Moderate | Omicron (BA.2) | 2022/2/ 19 | 2022/2/ 18 | 2022/3/ 16 18:15 | 26 |
| BA.2 | 276880 | male | 24 | Mild | Omicron (BA.2) | 2022/2/ 19 | 2022/2/ 18 | 2022/3/ 12 13:46 | 21 |
| BA.2 | 276882 | female | 52 | Mild | Omicron (BA.2) | 2022/2/ 19 | 2022/2/ 18 | 2022/3/ 18 16:48 | 27 |
| BA.2 | 276914 | female | 28 | Mild | Omicron (BA.2) | 2022/2/ 19 | 2022/2/ 18 | 2022/3/ 18 13:04 | 27 |
| BA.2 | 276915 | female | 50 | Mild | Omicron (BA.2) | 2022/2/ 19 | 2022/2/ 18 | 2022/3/ 18 10:11 | 27 |
| BA.2 | 276931 | female | 59 | Mild | Omicron (BA.2) | 2022/2/ 19 | 2022/2/ 16 | 2022/3/ 17 20:45 | 26 |
| BA.2 | 276932 | male | 2 | Mild | Omicron (BA.2) | 2022/2/ 19 | 2022/2/ 18 | 2022/3/ 17 20:42 | 26 |
| BA.2 | 276945 | male | 28 | Moderate | Omicron (BA.2) | 2022/2/ 19 | 2022/2/ 17 | 2022/3/ 18 13:02 | 27 |
| BA.2 | 276950 | male | 50 | Mild | Omicron (BA.2) | 2022/2/ 19 | 2022/2/ 19 | 2022/3/ 16 17:19 | 25 |
| BA.2 | 276952 | female | 5 | Mild | Omicron (BA.2) | 2022/2/ 19 | 2022/2/ 18 | 2022/3/6 11:00 | 15 |
| BA.2 | 276953 | female | 32 | Mild | Omicron (BA.2) | 2022/2/ 19 | 2022/2/ 18 | 2022/3/ 18 17:12 | 27 |
| BA.2 | 276957 | female | 39 | Mild | Omicron (BA.2) | 2022/2/20 | 2022/2/ 18 | 2022/3/6 11:00 | 14 |
| BA.2 | 276958 | male | 62 | Mild | Omicron (BA.2) | 2022/2/20 | 2022/2/ 19 | 2022/3/ 18 13:03 | 26 |
| BA.2 | 276959 | male | 33 | Mild | Omicron (BA.2) | 2022/2/20 | 2022/2/ 15 | 2022/3/ 17 17:30 | 26 |
| BA.2 | 276966 | female | 20 | Mild | Omicron (BA.2) | 2022/2/20 | 2022/2/ 18 | 2022/3/21 14:38 | 29 |
| BA.2 | 276972 | female | 61 | Mild | Omicron (BA.2) | 2022/2/20 | 2022/2/ 19 | 2022/3/ 17 17:30 | 25 |
| BA.2 | 276999 | male | 45 | Mild | Omicron (BA.2) | 2022/2/20 | 2022/2/ 19 | 2022/3/ 12 11:09 | 20 |
| BA.2 | 277007 | male | 35 | Mild | Omicron (BA.2) | 2022/2/20 | 2022/2/ 19 | 2022/3/ 18 14:34 | 26 |
| BA.2 | 277024 | male | 12 | Mild | Omicron (BA.2) | 2022/2/20 | 2022/2/ 19 | 2022/3/ 18 10:11 | 26 |
| BA.2 | 277025 | female | 15 | Mild | Omicron (BA.2) | 2022/2/20 | 2022/2/ 19 | 2022/3/20 15:04 | 28 |
| BA.2 | 277034 | female | 2 | Mild | Omicron (BA.2) | 2022/2/20 | 2022/2/ 19 | 2022/3/ 14 14:01 | 22 |
| BA.2 | 277035 | female | 27 | Mild | Omicron (BA.2) | 2022/2/20 | 2022/2/20 | 2022/3/ 18 14:29 | 26 |
| BA.2 | 277037 | male | 5 | Mild | Omicron (BA.2) | 2022/2/20 | 2022/2/ 19 | 2022/3/ 19 14:37 | 27 |
| BA.2 | 277038 | male | 1 | Asymptomatic | Omicron (BA.2) | 2022/2/20 | 2022/2/20 | 2022/3/ 19 14:39 | 27 |
| BA.2 | 277048 | female | 42 | Mild | Omicron (BA.2) | 2022/2/21 | 2022/2/ 19 | 2022/3/ 18 13:01 | 26 |
| BA.2 | 277052 | female | 63 | Mild | Omicron (BA.2) | 2022/2/21 | 2022/2/ 19 | 2022/3/ 18 13:05 | 25 |
| BA.2 | 277053 | male | 42 | Mild | Omicron (BA.2) | 2022/2/21 | 2022/2/ 18 | 2022/3/ 17 9:19 | 24 |
| BA.2 | 277054 | female | 8 | Moderate | Omicron (BA.2) | 2022/2/21 | 2022/2/20 | 2022/3/ 17 17:30 | 24 |

| BA.2 | 277064 | female | 33 | Mild | Omicron (BA.2) | 2022/2/21 | 2022/2/20 | 2022/3/ 16 18:36 | 23 |
| --- | --- | --- | --- | --- | --- | --- | --- | --- | --- |
| BA.2 | 277065 | female | 8 | Mild | Omicron (BA.2) | 2022/2/21 | 2022/2/20 | 2022/3/ 13 9:55 | 20 |
| BA.2 | 277092 | female | 61 | Moderate | Omicron (BA.2) | 2022/2/21 | 2022/2/ 19 | 2022/3/ 16 17:27 | 23 |
| BA.2 | 277102 | male | 16 | Mild | Omicron (BA.2) | 2022/2/21 | 2022/2/20 | 2022/3/ 16 17:19 | 23 |
| BA.2 | 277103 | male | 31 | Mild | Omicron (BA.2) | 2022/2/21 | 2022/2/20 | 2022/3/ 17 20:51 | 24 |
| BA.2 | 277104 | female | 30 | Mild | Omicron (BA.2) | 2022/2/21 | 2022/2/20 | 2022/3/ 17 17:30 | 24 |
| BA.2 | 277107 | female | 30 | Mild | Omicron (BA.2) | 2022/2/21 | 2022/2/20 | 2022/3/ 18 16:49 | 25 |
| BA.2 | 277108 | male | 0 | Moderate | Omicron (BA.2) | 2022/2/21 | 2022/2/21 | 2022/3/27 18:09 | 34 |
| BA.2 | 277113 | male | 15 | Mild | Omicron (BA.2) | 2022/2/21 | 2022/2/20 | 2022/3/ 15 11:23 | 22 |
| BA.2 | 277118 | female | 9 | Mild | Omicron (BA.2) | 2022/2/21 | 2022/2/20 | 2022/3/ 18 19:57 | 25 |
| BA.2 | 277119 | female | 29 | Moderate | Omicron (BA.2) | 2022/2/21 | 2022/2/20 | 2022/3/ 18 19:58 | 25 |
| BA.2 | 277120 | female | 2 | Mild | Omicron (BA.2) | 2022/2/21 | 2022/2/20 | 2022/3/7 10:55 | 14 |
| BA.2 | 277138 | male | 16 | Mild | Omicron (BA.2) | 2022/2/21 | 2022/2/ 19 | 2022/3/ 17 14:20 | 24 |
| BA.2 | 277152 | male | 3 | Mild | Omicron (BA.2) | 2022/2/21 | 2022/2/21 | 2022/3/ 13 15:16 | 20 |
| BA.2 | 277153 | female | 26 | Mild | Omicron (BA.2) | 2022/2/21 | 2022/2/20 | 2022/3/ 18 16:43 | 25 |
| BA.2 | 277161 | male | 36 | Moderate | Omicron (BA.2) | 2022/2/21 | 2022/2/21 | 2022/3/ 17 18:16 | 24 |
| BA.2 | 277165 | male | 5 | Moderate | Omicron (BA.2) | 2022/2/21 | 2022/2/20 | 2022/3/ 11 11:00 | 17 |
| BA.2 | 277167 | male | 4 | Mild | Omicron (BA.2) | 2022/2/21 | 2022/2/20 | 2022/3/ 10 12:00 | 17 |
| BA.2 | 277168 | male | 59 | Mild | Omicron (BA.2) | 2022/2/22 | 2022/2/21 | 2022/3/ 17 18:55 | 24 |
| BA.2 | 277169 | female | 2 | Mild | Omicron (BA.2) | 2022/2/22 | 2022/2/21 | 2022/3/ 17 18:55 | 24 |
| BA.2 | 277170 | female | 4 | Mild | Omicron (BA.2) | 2022/2/22 | 2022/2/20 | 2022/3/ 17 18:43 | 24 |
| BA.2 | 277171 | female | 30 | Moderate | Omicron (BA.2) | 2022/2/22 | 2022/2/20 | 2022/3/20 10:22 | 26 |
| BA.2 | 277172 | female | 36 | Mild | Omicron (BA.2) | 2022/2/22 | 2022/2/21 | 2022/3/21 13:00 | 27 |
| BA.2 | 277175 | female | 54 | Moderate | Omicron (BA.2) | 2022/2/22 | 2022/2/20 | 2022/3/ 14 11:28 | 20 |
| BA.2 | 277176 | male | 34 | Mild | Omicron (BA.2) | 2022/2/22 | 2022/2/21 | 2022/3/20 10:21 | 26 |
| BA.2 | 277177 | male | 45 | Mild | Omicron (BA.2) | 2022/2/22 | 2022/2/21 | 2022/3/ 17 22:10 | 24 |
| BA.2 | 277179 | female | 32 | Mild | Omicron (BA.2) | 2022/2/22 | 2022/2/21 | 2022/3/ 17 17:30 | 24 |
| BA.2 | 277181 | male | 57 | Mild | Omicron (BA.2) | 2022/2/22 | 2022/2/20 | 2022/3/ 17 14:01 | 24 |
| BA.2 | 277182 | male | 51 | Mild | Omicron (BA.2) | 2022/2/22 | 2022/2/21 | 2022/3/ 17 9:42 | 23 |
| BA.2 | 277184 | female | 6 | Mild | Omicron (BA.2) | 2022/2/22 | 2022/2/20 | 2022/3/ 18 10:14 | 24 |
| BA.2 | 277185 | female | 31 | Mild | Omicron (BA.2) | 2022/2/22 | 2022/2/21 | 2022/3/ 12 19:29 | 19 |
| BA.2 | 277187 | male | 58 | Mild | Omicron (BA.2) | 2022/2/22 | 2022/2/ 19 | 2022/3/ 17 21:28 | 24 |
| BA.2 | 277188 | male | 10 | Mild | Omicron (BA.2) | 2022/2/22 | 2022/2/21 | 2022/3/ 17 14:32 | 23 |
| BA.2 | 277193 | female | 48 | Asymptomatic | Omicron (BA.2) | 2022/2/22 | 2022/2/21 | 2022/3/ 17 17:30 | 23 |
| BA.2 | 277194 | female | 47 | Moderate | Omicron (BA.2) | 2022/2/22 | 2022/2/21 | 2022/3/ 18 11:29 | 24 |
| BA.2 | 277195 | male | 22 | Mild | Omicron (BA.2) | 2022/2/22 | 2022/2/21 | 2022/3/ 18 10:24 | 24 |
| BA.2 | 277200 | male | 23 | Moderate | Omicron (BA.2) | 2022/2/22 | 2022/2/20 | 2022/3/ 11 14:17 | 17 |
| BA.2 | 277201 | female | 35 | Mild | Omicron (BA.2) | 2022/2/22 | 2022/2/21 | 2022/3/ 18 12:09 | 24 |

| BA.2 | 277202 | female | 68 | Mild | Omicron (BA.2) | 2022/2/22 | 2022/2/22 | 2022/3/ 18 18:23 | 24 |
| --- | --- | --- | --- | --- | --- | --- | --- | --- | --- |
| BA.2 | 277204 | male | 2 | Asymptomatic | Omicron (BA.2) | 2022/2/22 | 2022/2/21 | 2022/3/ 18 11:32 | 24 |
| BA.2 | 277205 | female | 26 | Asymptomatic | Omicron (BA.2) | 2022/2/22 | 2022/2/21 | 2022/3/ 18 12:09 | 24 |
| BA.2 | 277209 | female | 32 | Asymptomatic | Omicron (BA.2) | 2022/2/22 | 2022/2/21 | 2022/3/ 18 17:45 | 24 |
| BA.2 | 277210 | female | 33 | Asymptomatic | Omicron (BA.2) | 2022/2/22 | 2022/2/21 | 2022/3/20 9:14 | 26 |
| BA.2 | 277212 | male | 15 | Asymptomatic | Omicron (BA.2) | 2022/2/22 | 2022/2/21 | 2022/3/ 17 21:12 | 23 |
| BA.2 | 277213 | female | 26 | Mild | Omicron (BA.2) | 2022/2/22 | 2022/2/20 | 2022/3/ 17 10:34 | 23 |
| BA.2 | 277218 | female | 7 | Mild | Omicron (BA.2) | 2022/2/22 | 2022/2/21 | 2022/3/ 18 16:49 | 24 |
| BA.2 | 277219 | male | 66 | Moderate | Omicron (BA.2) | 2022/2/22 | 2022/2/21 | 2022/3/ 17 17:58 | 23 |
| BA.2 | 277220 | female | 10 | Mild | Omicron (BA.2) | 2022/2/22 | 2022/2/21 | 2022/3/ 15 12:31 | 21 |
| BA.2 | 277230 | male | 46 | Asymptomatic | Omicron (BA.2) | 2022/2/22 | 2022/2/21 | 2022/3/ 17 15:33 | 23 |
| BA.2 | 277231 | female | 42 | Asymptomatic | Omicron (BA.2) | 2022/2/22 | 2022/2/21 | 2022/3/ 17 14:33 | 23 |
| BA.2 | 277233 | female | 69 | Moderate | Omicron (BA.2) | 2022/2/22 | 2022/2/21 | 2022/3/ 17 19:17 | 23 |
| BA.2 | 277248 | male | 6 | Asymptomatic | Omicron (BA.2) | 2022/2/22 | 2022/2/21 | 2022/3/ 18 15:54 | 24 |
| BA.2 | 277250 | male | 15 | Asymptomatic | Omicron (BA.2) | 2022/2/22 | 2022/2/21 | 2022/3/ 18 15:54 | 24 |
| BA.2 | 277252 | male | 53 | Mild | Omicron (BA.2) | 2022/2/22 | 2022/2/ 19 | 2022/3/ 18 16:30 | 24 |
| BA.2 | 277281 | female | 29 | Mild | Omicron (BA.2) | 2022/2/22 | 2022/2/21 | 2022/3/ 18 14:28 | 24 |
| BA.2 | 277282 | male | 2 | Mild | Omicron (BA.2) | 2022/2/23 | 2022/2/22 | 2022/3/ 18 12:00 | 23 |
| BA.2 | 277283 | male | 31 | Mild | Omicron (BA.2) | 2022/2/23 | 2022/2/22 | 2022/3/ 17 21:13 | 23 |
| BA.2 | 277284 | female | 4 | Mild | Omicron (BA.2) | 2022/2/22 | 2022/2/21 | 2022/3/ 18 14:27 | 24 |
| BA.2 | 277287 | female | 34 | Moderate | Omicron (BA.2) | 2022/2/22 | 2022/2/21 | 2022/3/ 10 11:51 | 16 |
| BA.2 | 277288 | male | 6 | Mild | Omicron (BA.2) | 2022/2/22 | 2022/2/21 | 2022/3/ 12 14:06 | 18 |
| BA.2 | 277289 | female | 50 | Mild | Omicron (BA.2) | 2022/2/22 | 2022/2/21 | 2022/3/ 14 10:53 | 20 |
| BA.2 | 277290 | male | 50 | Moderate | Omicron (BA.2) | 2022/2/22 | 2022/2/22 | 2022/3/20 10:51 | 26 |
| BA.2 | 277291 | male | 53 | Mild | Omicron (BA.2) | 2022/2/22 | 2022/2/20 | 2022/3/ 18 16:26 | 24 |
| BA.2 | 277292 | male | 56 | Moderate | Omicron (BA.2) | 2022/2/22 | 2022/2/20 | 2022/3/ 17 17:30 | 23 |
| BA.2 | 277293 | male | 52 | Mild | Omicron (BA.2) | 2022/2/22 | 2022/2/20 | 2022/3/ 16 17:31 | 22 |
| BA.2 | 277296 | male | 55 | Mild | Omicron (BA.2) | 2022/2/22 | 2022/2/21 | 2022/3/ 17 14:31 | 23 |
| BA.2 | 277297 | female | 23 | Mild | Omicron (BA.2) | 2022/2/22 | 2022/2/21 | 2022/3/22 9:09 | 27 |
| BA.2 | 277298 | male | 2 | Mild | Omicron (BA.2) | 2022/2/23 | 2022/2/21 | 2022/3/29 15:15 | 35 |
| BA.2 | 277299 | male | 13 | Mild | Omicron (BA.2) | 2022/2/22 | 2022/2/21 | 2022/3/ 18 17:43 | 24 |
| BA.2 | 277300 | female | 40 | Mild | Omicron (BA.2) | 2022/2/22 | 2022/2/21 | 2022/3/ 18 17:43 | 24 |
| BA.2 | 277301 | male | 24 | Mild | Omicron (BA.2) | 2022/2/22 | 2022/2/21 | 2022/3/ 17 14:29 | 23 |
| BA.2 | 277302 | female | 26 | Mild | Omicron (BA.2) | 2022/2/22 | 2022/2/21 | 2022/3/21 11:09 | 26 |
| BA.2 | 277305 | male | 54 | Mild | Omicron (BA.2) | 2022/2/22 | 2022/2/21 | 2022/3/ 17 20:45 | 23 |
| BA.2 | 277306 | female | 34 | Mild | Omicron (BA.2) | 2022/2/22 | 2022/2/21 | 2022/3/ 16 19:52 | 22 |
| BA.2 | 277312 | male | 23 | Mild | Omicron (BA.2) | 2022/2/23 | 2022/2/21 | 2022/3/ 19 13:26 | 25 |
| BA.2 | 277316 | female | 57 | Mild | Omicron (BA.2) | 2022/2/23 | 2022/2/20 | 2022/3/20 16:50 | 26 |

| BA.2 | 277318 | male | 67 | Asymptomatic | Omicron (BA.2) | 2022/2/23 | 2022/2/22 | 2022/3/ 17 16:49 | 23 |
| --- | --- | --- | --- | --- | --- | --- | --- | --- | --- |
| BA.2 | 277319 | female | 29 | Mild | Omicron (BA.2) | 2022/2/23 | 2022/2/20 | 2022/3/ 18 16:27 | 24 |
| BA.2 | 277321 | female | 30 | Mild | Omicron (BA.2) | 2022/2/23 | 2022/2/20 | 2022/3/9 15:38 | 14 |
| BA.2 | 277329 | male | 60 | Moderate | Omicron (BA.2) | 2022/2/23 | 2022/2/22 | 2022/3/ 18 15:23 | 23 |
| BA.2 | 277330 | male | 3 | Asymptomatic | Omicron (BA.2) | 2022/2/23 | 2022/2/22 | 2022/3/ 16 17:53 | 21 |
| BA.2 | 277338 | female | 15 | Mild | Omicron (BA.2) | 2022/2/23 | 2022/2/22 | 2022/3/ 10 12:06 | 15 |
| BA.2 | 277339 | female | 19 | Mild | Omicron (BA.2) | 2022/2/23 | 2022/2/22 | 2022/3/ 18 20:30 | 23 |
| BA.2 | 277340 | male | 2 | Mild | Omicron (BA.2) | 2022/2/23 | 2022/2/22 | 2022/3/21 11:09 | 26 |
| BA.2 | 277342 | female | 29 | Moderate | Omicron (BA.2) | 2022/2/23 | 2022/2/22 | 2022/3/21 9:08 | 26 |
| BA.2 | 277343 | female | 34 | Mild | Omicron (BA.2) | 2022/2/23 | 2022/2/22 | 2022/3/ 16 17:42 | 21 |
| BA.2 | 277344 | female | 10 | Moderate | Omicron (BA.2) | 2022/2/23 | 2022/2/22 | 2022/3/22 9:22 | 27 |
| BA.2 | 277345 | female | 26 | Mild | Omicron (BA.2) | 2022/2/23 | 2022/2/22 | 2022/3/ 18 14:31 | 23 |
| BA.2 | 277354 | male | 1 | Mild | Omicron (BA.2) | 2022/2/23 | 2022/2/22 | 2022/3/8 10:05 | 13 |
| BA.2 | 277355 | female | 40 | Mild | Omicron (BA.2) | 2022/2/23 | 2022/2/22 | 2022/3/ 16 18:35 | 21 |
| BA.2 | 277357 | female | 26 | Mild | Omicron (BA.2) | 2022/2/23 | 2022/2/21 | 2022/3/ 18 14:26 | 23 |
| BA.2 | 277370 | male | 11 | Mild | Omicron (BA.2) | 2022/2/23 | 2022/2/21 | 2022/3/8 9:15 | 13 |
| BA.2 | 277373 | male | 56 | Mild | Omicron (BA.2) | 2022/2/23 | 2022/2/21 | 2022/3/20 16:49 | 25 |
| BA.2 | 277374 | male | 34 | Mild | Omicron (BA.2) | 2022/2/23 | 2022/2/21 | 2022/3/20 16:50 | 25 |
| BA.2 | 277387 | male | 3 | Moderate | Omicron (BA.2) | 2022/2/23 | 2022/2/22 | 2022/3/ 18 14:26 | 23 |
| BA.2 | 277394 | female | 15 | Moderate | Omicron (BA.2) | 2022/2/23 | 2022/2/22 | 2022/3/ 18 16:11 | 23 |
| BA.2 | 277395 | male | 14 | Asymptomatic | Omicron (BA.2) | 2022/2/23 | 2022/2/22 | 2022/3/ 18 16:25 | 23 |
| BA.2 | 277403 | female | 52 | Mild | Omicron (BA.2) | 2022/2/23 | 2022/2/22 | 2022/3/ 18 10:00 | 23 |
| BA.2 | 277406 | female | 32 | Mild | Omicron (BA.2) | 2022/2/23 | 2022/2/22 | 2022/3/ 19 13:24 | 24 |
| BA.2 | 277407 | female | 17 | Mild | Omicron (BA.2) | 2022/2/23 | 2022/2/22 | 2022/3/ 17 14:32 | 22 |
| BA.2 | 277410 | female | 1 | Mild | Omicron (BA.2) | 2022/2/23 | 2022/2/22 | 2022/3/20 19:57 | 25 |
| BA.2 | 277413 | female | 27 | Mild | Omicron (BA.2) | 2022/2/23 | 2022/2/22 | 2022/3/20 19:53 | 25 |
| BA.2 | 277414 | female | 15 | Mild | Omicron (BA.2) | 2022/2/23 | 2022/2/22 | 2022/3/ 18 17:43 | 23 |
| BA.2 | 277416 | female | 27 | Moderate | Omicron (BA.2) | 2022/2/23 | 2022/2/22 | 2022/3/ 18 15:04 | 23 |
| BA.2 | 277418 | male | 8 | Moderate | Omicron (BA.2) | 2022/2/23 | 2022/2/21 | 2022/3/21 13:22 | 26 |
| BA.2 | 277421 | female | 30 | Mild | Omicron (BA.2) | 2022/2/23 | 2022/2/21 | 2022/3/ 16 15:17 | 21 |
| BA.2 | 277422 | female | 3 | Mild | Omicron (BA.2) | 2022/2/23 | 2022/2/22 | 2022/3/9 12:17 | 14 |
| BA.2 | 277424 | female | 30 | Moderate | Omicron (BA.2) | 2022/2/23 | 2022/2/22 | 2022/3/ 14 16:15 | 19 |
| BA.2 | 277425 | male | 6 | Mild | Omicron (BA.2) | 2022/2/23 | 2022/2/22 | 2022/3/ 18 20:30 | 23 |
| BA.2 | 277426 | male | 33 | Mild | Omicron (BA.2) | 2022/2/23 | 2022/2/22 | 2022/3/ 10 11:38 | 15 |
| BA.2 | 277427 | female | 6 | Mild | Omicron (BA.2) | 2022/2/23 | 2022/2/22 | 2022/3/ 12 10:38 | 17 |
| BA.2 | 277428 | female | 27 | Mild | Omicron (BA.2) | 2022/2/23 | 2022/2/22 | 2022/3/ 16 15:17 | 21 |
| BA.2 | 277437 | male | 58 | Mild | Omicron (BA.2) | 2022/2/23 | 2022/2/22 | 2022/3/ 18 16:32 | 23 |
| BA.2 | 277439 | male | 15 | Asymptomatic | Omicron (BA.2) | 2022/2/23 | 2022/2/22 | 2022/3/ 18 20:46 | 23 |

| BA.2 | 277440 | female | 41 | Asymptomatic | Omicron (BA.2) | 2022/2/23 | 2022/2/22 | 2022/3/ 18 20:22 | 23 |
| --- | --- | --- | --- | --- | --- | --- | --- | --- | --- |
| BA.2 | 277442 | female | 22 | Mild | Omicron (BA.2) | 2022/2/23 | 2022/2/22 | 2022/3/ 13 9:12 | 17 |
| BA.2 | 277443 | female | 47 | Moderate | Omicron (BA.2) | 2022/2/23 | 2022/2/22 | 2022/3/ 15 12:53 | 20 |
| BA.2 | 277445 | female | 73 | Mild | Omicron (BA.2) | 2022/2/23 | 2022/2/21 | 2022/3/ 19 11:00 | 23 |
| BA.2 | 277448 | male | 3 | Mild | Omicron (BA.2) | 2022/2/23 | 2022/2/22 | 2022/3/ 12 12:04 | 17 |
| BA.2 | 277449 | female | 3 | Asymptomatic | Omicron (BA.2) | 2022/2/23 | 2022/2/22 | 2022/3/21 11:12 | 26 |
| BA.2 | 277450 | male | 8 | Asymptomatic | Omicron (BA.2) | 2022/2/23 | 2022/2/22 | 2022/3/20 18:47 | 25 |
| BA.2 | 277451 | female | 4 | Asymptomatic | Omicron (BA.2) | 2022/2/25 | 2022/2/24 | 2022/3/ 11 10:32 | 14 |
| BA.2 | 277452 | male | 31 | Asymptomatic | Omicron (BA.2) | 2022/2/25 | 2022/2/24 | 2022/3/ 15 12:03 | 18 |
| BA.2 | 277453 | male | 24 | Mild | Omicron (BA.2) | 2022/2/23 | 2022/2/22 | 2022/3/ 19 14:38 | 24 |
| BA.2 | 277454 | female | 28 | Mild | Omicron (BA.2) | 2022/2/24 | 2022/2/22 | 2022/3/20 15:00 | 25 |
| BA.2 | 277455 | male | 8 | Mild | Omicron (BA.2) | 2022/2/24 | 2022/2/23 | 2022/3/20 15:00 | 25 |
| BA.2 | 277456 | male | 2 | Asymptomatic | Omicron (BA.2) | 2022/2/24 | 2022/2/23 | 2022/3/20 12:00 | 24 |
| BA.2 | 277457 | male | 28 | Asymptomatic | Omicron (BA.2) | 2022/2/24 | 2022/2/23 | 2022/3/20 12:40 | 24 |
| BA.2 | 277458 | female | 11 | Mild | Omicron (BA.2) | 2022/2/24 | 2022/2/22 | 2022/3/23 10:00 | 27 |
| BA.2 | 277459 | female | 28 | Mild | Omicron (BA.2) | 2022/2/24 | 2022/2/ 19 | 2022/3/ 17 14:00 | 22 |
| BA.2 | 277460 | male | 55 | Asymptomatic | Omicron (BA.2) | 2022/2/24 | 2022/2/23 | 2022/3/ 17 11:37 | 21 |
| BA.2 | 277462 | female | 47 | Mild | Omicron (BA.2) | 2022/2/24 | 2022/2/23 | 2022/3/ 18 15:58 | 22 |
| BA.2 | 277463 | female | 49 | Moderate | Omicron (BA.2) | 2022/2/24 | 2022/2/23 | 2022/3/ 17 17:30 | 22 |
| BA.2 | 277464 | female | 52 | Mild | Omicron (BA.2) | 2022/2/24 | 2022/2/23 | 2022/3/ 17 21:11 | 22 |
| BA.2 | 277465 | male | 35 | Mild | Omicron (BA.2) | 2022/2/24 | 2022/2/23 | 2022/3/ 18 16:26 | 23 |
| BA.2 | 277466 | female | 52 | Mild | Omicron (BA.2) | 2022/2/24 | 2022/2/23 | 2022/3/ 16 18:30 | 21 |
| BA.2 | 277467 | male | 61 | Mild | Omicron (BA.2) | 2022/2/24 | 2022/2/22 | 2022/3/ 16 17:56 | 20 |
| BA.2 | 277468 | female | 54 | Moderate | Omicron (BA.2) | 2022/2/24 | 2022/2/22 | 2022/3/ 18 17:14 | 22 |
| BA.2 | 277469 | female | 48 | Moderate | Omicron (BA.2) | 2022/2/24 | 2022/2/23 | 2022/3/ 18 10:12 | 22 |
| BA.2 | 277470 | female | 11 | Mild | Omicron (BA.2) | 2022/2/24 | 2022/2/23 | 2022/3/ 19 10:56 | 23 |
| BA.2 | 277474 | male | 22 | Mild | Omicron (BA.2) | 2022/2/24 | 2022/2/23 | 2022/3/ 18 14:28 | 22 |
| BA.2 | 277477 | male | 7 | Moderate | Omicron (BA.2) | 2022/2/24 | 2022/2/22 | 2022/3/ 16 17:17 | 20 |
| BA.2 | 277484 | male | 45 | Mild | Omicron (BA.2) | 2022/2/24 | 2022/2/23 | 2022/3/ 11 11:39 | 15 |
| BA.2 | 277485 | female | 40 | Asymptomatic | Omicron (BA.2) | 2022/2/24 | 2022/2/23 | 2022/3/ 18 10:00 | 22 |
| BA.2 | 277487 | female | 16 | Moderate | Omicron (BA.2) | 2022/2/24 | 2022/2/23 | 2022/3/ 18 20:30 | 22 |
| BA.2 | 277488 | male | 27 | Mild | Omicron (BA.2) | 2022/2/24 | 2022/2/22 | 2022/3/ 18 16:34 | 22 |
| BA.2 | 277489 | female | 42 | Moderate | Omicron (BA.2) | 2022/2/24 | 2022/2/23 | 2022/3/26 10:45 | 30 |
| BA.2 | 277491 | male | 43 | Mild | Omicron (BA.2) | 2022/2/24 | 2022/2/23 | 2022/3/ 18 10:34 | 22 |
| BA.2 | 277496 | male | 24 | Mild | Omicron (BA.2) | 2022/2/24 | 2022/2/23 | 2022/3/ 18 16:27 | 22 |
| BA.2 | 277510 | male | 30 | Moderate | Omicron (BA.2) | 2022/2/24 | 2022/2/23 | 2022/3/ 16 17:18 | 20 |
| BA.2 | 277511 | female | 46 | Asymptomatic | Omicron (BA.2) | 2022/2/24 | 2022/2/23 | 2022/3/ 17 21:22 | 21 |
| BA.2 | 277514 | female | 58 | Mild | Omicron (BA.2) | 2022/2/24 | 2022/2/23 | 2022/3/21 11:07 | 25 |

| BA.2 | 277518 | male | 56 | Asymptomatic | Omicron (BA.2) | 2022/2/24 | 2022/2/23 | 2022/3/ 17 15:33 | 21 |
| --- | --- | --- | --- | --- | --- | --- | --- | --- | --- |
| BA.2 | 277524 | male | 55 | Mild | Omicron (BA.2) | 2022/2/24 | 2022/2/23 | 2022/3/ 14 13:50 | 18 |
| BA.2 | 277536 | female | 52 | Mild | Omicron (BA.2) | 2022/2/24 | 2022/2/23 | 2022/3/ 19 14:38 | 23 |
| BA.2 | 277538 | male | 5 | Mild | Omicron (BA.2) | 2022/2/24 | 2022/2/24 | 2022/3/ 18 17:13 | 22 |
| BA.2 | 277539 | female | 45 | Mild | Omicron (BA.2) | 2022/2/24 | 2022/2/23 | 2022/3/ 16 16:28 | 20 |
| BA.2 | 277541 | female | 36 | Asymptomatic | Omicron (BA.2) | 2022/2/24 | 2022/2/24 | 2022/3/ 18 17:14 | 22 |
| BA.2 | 277544 | female | 53 | Asymptomatic | Omicron (BA.2) | 2022/2/24 | 2022/2/23 | 2022/3/21 11:31 | 25 |
| BA.2 | 277545 | female | 30 | Asymptomatic | Omicron (BA.2) | 2022/2/24 | 2022/2/23 | 2022/3/ 18 14:26 | 22 |
| BA.2 | 277549 | female | 12 | Asymptomatic | Omicron (BA.2) | 2022/2/24 | 2022/2/23 | 2022/3/ 18 17:14 | 22 |
| BA.2 | 277550 | male | 68 | Mild | Omicron (BA.2) | 2022/2/24 | 2022/2/22 | 2022/3/20 19:54 | 24 |
| BA.2 | 277563 | male | 46 | Mild | Omicron (BA.2) | 2022/2/24 | 2022/2/23 | 2022/3/ 18 10:36 | 22 |
| BA.2 | 277565 | male | 39 | Moderate | Omicron (BA.2) | 2022/2/24 | 2022/2/23 | 2022/3/ 10 14:23 | 14 |
| BA.2 | 277569 | female | 2 | Mild | Omicron (BA.2) | 2022/2/24 | 2022/2/24 | 2022/3/23 11:14 | 27 |
| BA.2 | 277584 | male | 17 | Mild | Omicron (BA.2) | 2022/2/24 | 2022/2/22 | 2022/3/ 16 16:03 | 20 |
| BA.2 | 277585 | male | 47 | Moderate | Omicron (BA.2) | 2022/2/24 | 2022/2/23 | 2022/3/ 18 20:21 | 22 |
| BA.2 | 277589 | male | 65 | Asymptomatic | Omicron (BA.2) | 2022/2/24 | 2022/2/23 | 2022/3/22 9:47 | 25 |
| BA.2 | 277592 | male | 37 | Asymptomatic | Omicron (BA.2) | 2022/2/24 | 2022/2/24 | 2022/3/26 9:57 | 29 |
| BA.2 | 277593 | male | 63 | Asymptomatic | Omicron (BA.2) | 2022/2/24 | 2022/2/23 | 2022/3/ 18 11:29 | 22 |
| BA.2 | 277595 | male | 32 | Asymptomatic | Omicron (BA.2) | 2022/2/24 | 2022/2/23 | 2022/3/ 18 11:28 | 22 |
| BA.2 | 277596 | male | 32 | Asymptomatic | Omicron (BA.2) | 2022/2/24 | 2022/2/23 | 2022/3/ 13 15:43 | 17 |
| BA.2 | 277599 | female | 30 | Mild | Omicron (BA.2) | 2022/2/24 | 2022/2/20 | 2022/3/ 18 15:45 | 22 |
| BA.2 | 277600 | female | 4 | Asymptomatic | Omicron (BA.2) | 2022/2/24 | 2022/2/23 | 2022/3/ 16 18:00 | 20 |
| BA.2 | 277601 | male | 9 | Mild | Omicron (BA.2) | 2022/2/24 | 2022/2/23 | 2022/3/ 18 15:43 | 22 |
| BA.2 | 277602 | male | 58 | Asymptomatic | Omicron (BA.2) | 2022/2/24 | 2022/2/23 | 2022/3/ 17 21:04 | 21 |
| BA.2 | 277604 | male | 67 | Asymptomatic | Omicron (BA.2) | 2022/2/24 | 2022/2/23 | 2022/3/20 13:09 | 24 |
| BA.2 | 277607 | female | 65 | Asymptomatic | Omicron (BA.2) | 2022/2/24 | 2022/2/23 | 2022/3/ 17 19:11 | 21 |
| BA.2 | 277610 | male | 52 | Asymptomatic | Omicron (BA.2) | 2022/2/25 | 2022/2/24 | 2022/3/20 15:00 | 24 |
| BA.2 | 277611 | female | 29 | Asymptomatic | Omicron (BA.2) | 2022/2/25 | 2022/2/24 | 2022/3/ 18 15:39 | 21 |
| BA.2 | 277612 | male | 71 | Asymptomatic | Omicron (BA.2) | 2022/2/25 | 2022/2/24 | 2022/4/ 1 8:33 | 35 |
| BA.2 | 277613 | male | 32 | Asymptomatic | Omicron (BA.2) | 2022/2/25 | 2022/2/24 | 2022/3/20 13:44 | 24 |
| BA.2 | 277614 | female | 49 | Asymptomatic | Omicron (BA.2) | 2022/2/25 | 2022/2/24 | 2022/3/ 17 21:10 | 21 |
| BA.2 | 277616 | male | 49 | Mild | Omicron (BA.2) | 2022/2/25 | 2022/2/23 | 2022/3/ 18 18:21 | 22 |
| BA.2 | 277620 | male | 60 | Moderate | Omicron (BA.2) | 2022/2/25 | 2022/2/24 | 2022/3/ 17 16:26 | 21 |
| BA.2 | 277621 | male | 66 | Asymptomatic | Omicron (BA.2) | 2022/2/25 | 2022/2/24 | 2022/3/ 17 21:10 | 21 |
| BA.2 | 277622 | female | 47 | Asymptomatic | Omicron (BA.2) | 2022/2/25 | 2022/2/24 | 2022/3/21 13:00 | 24 |
| BA.2 | 277625 | female | 37 | Asymptomatic | Omicron (BA.2) | 2022/2/25 | 2022/2/24 | 2022/3/ 18 14:41 | 21 |
| BA.2 | 277627 | male | 22 | Asymptomatic | Omicron (BA.2) | 2022/2/25 | 2022/2/24 | 2022/3/ 19 22:00 | 23 |
| BA.2 | 277628 | male | 50 | Mild | Omicron (BA.2) | 2022/2/25 | 2022/2/23 | 2022/3/ 17 17:30 | 20 |

| BA.2 | 277629 | female | 23 | Moderate | Omicron (BA.2) | 2022/2/25 | 2022/2/23 | 2022/3/ 19 14:37 | 22 |
| --- | --- | --- | --- | --- | --- | --- | --- | --- | --- |
| BA.2 | 277632 | female | 28 | Asymptomatic | Omicron (BA.2) | 2022/2/25 | 2022/2/24 | 2022/3/ 14 9:00 | 17 |
| BA.2 | 277635 | female | 25 | Asymptomatic | Omicron (BA.2) | 2022/2/25 | 2022/2/24 | 2022/3/ 15 9:16 | 18 |
| BA.2 | 277637 | male | 3 | Asymptomatic | Omicron (BA.2) | 2022/2/25 | 2022/2/24 | 2022/3/ 10 10:38 | 13 |
| BA.2 | 277638 | male | 28 | Asymptomatic | Omicron (BA.2) | 2022/2/25 | 2022/2/24 | 2022/3/23 15:37 | 26 |
| BA.2 | 277640 | male | 51 | Mild | Omicron (BA.2) | 2022/2/25 | 2022/2/24 | 2022/3/ 15 14:01 | 18 |
| BA.2 | 277642 | male | 17 | Mild | Omicron (BA.2) | 2022/2/25 | 2022/2/21 | 2022/3/ 12 8:00 | 15 |
| BA.2 | 277643 | female | 30 | Mild | Omicron (BA.2) | 2022/2/25 | 2022/2/24 | 2022/3/ 17 19:11 | 20 |
| BA.2 | 277648 | male | 56 | Mild | Omicron (BA.2) | 2022/2/25 | 2022/2/24 | 2022/3/ 17 21:11 | 20 |
| BA.2 | 277650 | male | 41 | Asymptomatic | Omicron (BA.2) | 2022/2/25 | 2022/2/24 | 2022/3/25 10:51 | 28 |
| BA.2 | 277654 | male | 20 | Mild | Omicron (BA.2) | 2022/2/25 | 2022/2/21 | 2022/3/ 14 11:28 | 17 |
| BA.2 | 277655 | female | 25 | Mild | Omicron (BA.2) | 2022/2/25 | 2022/2/24 | 2022/3/ 18 18:55 | 21 |
| BA.2 | 277660 | female | 19 | Mild | Omicron (BA.2) | 2022/2/25 | 2022/2/24 | 2022/3/ 17 21:12 | 20 |
| BA.2 | 277661 | male | 53 | Mild | Omicron (BA.2) | 2022/2/25 | 2022/2/23 | 2022/4/2 9:16 | 36 |
| BA.2 | 277662 | male | 49 | Mild | Omicron (BA.2) | 2022/2/25 | 2022/2/24 | 2022/3/20 11:00 | 23 |
| BA.2 | 277663 | male | 51 | Mild | Omicron (BA.2) | 2022/2/25 | 2022/2/22 | 2022/3/ 18 15:53 | 21 |
| BA.2 | 277668 | male | 44 | Asymptomatic | Omicron (BA.2) | 2022/2/25 | 2022/2/24 | 2022/3/ 19 12:58 | 22 |
| BA.2 | 277671 | female | 54 | Asymptomatic | Omicron (BA.2) | 2022/2/25 | 2022/2/25 | 2022/3/ 18 10:00 | 21 |
| BA.2 | 277677 | female | 60 | Mild | Omicron (BA.2) | 2022/2/25 | 2022/2/24 | 2022/3/ 17 21:04 | 20 |
| BA.2 | 277689 | male | 4 | Mild | Omicron (BA.2) | 2022/2/25 | 2022/2/24 | 2022/3/ 12 8:00 | 15 |
| BA.2 | 277690 | male | 4 | Mild | Omicron (BA.2) | 2022/2/25 | 2022/2/25 | 2022/3/ 12 17:03 | 15 |
| BA.2 | 277700 | male | 49 | Mild | Omicron (BA.2) | 2022/2/25 | 2022/2/24 | 2022/3/22 15:18 | 25 |
| BA.2 | 277702 | female | 26 | Mild | Omicron (BA.2) | 2022/2/25 | 2022/2/24 | 2022/3/ 18 16:44 | 21 |
| BA.2 | 277703 | female | 28 | Mild | Omicron (BA.2) | 2022/2/25 | 2022/2/24 | 2022/3/ 18 11:25 | 21 |
| BA.2 | 277704 | female | 42 | Mild | Omicron (BA.2) | 2022/2/25 | 2022/2/23 | 2022/3/ 18 15:42 | 21 |
| BA.2 | 277705 | male | 39 | Moderate | Omicron (BA.2) | 2022/2/25 | 2022/2/23 | 2022/3/ 18 15:44 | 21 |
| BA.2 | 277706 | female | 45 | Mild | Omicron (BA.2) | 2022/2/25 | 2022/2/24 | 2022/3/21 9:40 | 24 |
| BA.2 | 277709 | female | 8 | Mild | Omicron (BA.2) | 2022/2/25 | 2022/2/24 | 2022/3/ 18 15:45 | 21 |
| BA.2 | 277710 | female | 1 | Moderate | Omicron (BA.2) | 2022/2/25 | 2022/2/24 | 2022/3/ 17 14:14 | 20 |
| BA.2 | 277711 | female | 39 | Moderate | Omicron (BA.2) | 2022/2/25 | 2022/2/24 | 2022/3/ 19 11:13 | 22 |
| BA.2 | 277712 | female | 31 | Mild | Omicron (BA.2) | 2022/2/25 | 2022/2/24 | 2022/3/20 10:30 | 23 |
| BA.2 | 277714 | female | 41 | Asymptomatic | Omicron (BA.2) | 2022/2/25 | 2022/2/24 | 2022/3/ 18 14:24 | 21 |
| BA.2 | 277715 | male | 53 | Asymptomatic | Omicron (BA.2) | 2022/2/25 | 2022/2/25 | 2022/3/ 19 14:27 | 22 |
| BA.2 | 277716 | female | 54 | Mild | Omicron (BA.2) | 2022/2/25 | 2022/2/24 | 2022/3/ 18 17:13 | 21 |
| BA.2 | 277718 | female | 30 | Mild | Omicron (BA.2) | 2022/2/25 | 2022/2/25 | 2022/3/ 18 15:50 | 21 |
| BA.2 | 277720 | male | 65 | Mild | Omicron (BA.2) | 2022/2/25 | 2022/2/22 | 2022/3/ 18 10:00 | 21 |
| BA.2 | 277722 | female | 58 | Moderate | Omicron (BA.2) | 2022/2/25 | 2022/2/22 | 2022/4/ 1 10:37 | 35 |
| BA.2 | 277723 | male | 36 | Mild | Omicron (BA.2) | 2022/2/25 | 2022/2/23 | 2022/3/ 16 15:44 | 19 |

| BA.2 | 277724 | male | 52 | Mild | Omicron (BA.2) | 2022/2/25 | 2022/2/22 | 2022/3/ 17 21:05 | 20 |
| --- | --- | --- | --- | --- | --- | --- | --- | --- | --- |
| BA.2 | 277725 | male | 32 | Mild | Omicron (BA.2) | 2022/2/25 | 2022/2/22 | 2022/3/9 15:54 | 12 |
| BA.2 | 277726 | female | 39 | Mild | Omicron (BA.2) | 2022/2/25 | 2022/2/24 | 2022/3/ 14 13:49 | 17 |
| BA.2 | 277727 | male | 63 | Mild | Omicron (BA.2) | 2022/2/25 | 2022/2/23 | 2022/3/ 18 20:30 | 21 |
| BA.2 | 277728 | female | 64 | Mild | Omicron (BA.2) | 2022/2/25 | 2022/2/22 | 2022/3/ 18 20:30 | 21 |
| BA.2 | 277730 | female | 48 | Mild | Omicron (BA.2) | 2022/2/25 | 2022/2/23 | 2022/3/ 18 10:00 | 21 |
| BA.2 | 277731 | male | 46 | Asymptomatic | Omicron (BA.2) | 2022/2/25 | 2022/2/24 | 2022/3/20 15:00 | 23 |
| BA.2 | 277733 | female | 47 | Asymptomatic | Omicron (BA.2) | 2022/2/25 | 2022/2/24 | 2022/3/ 18 10:00 | 21 |
| BA.2 | 277734 | male | 50 | Moderate | Omicron (BA.2) | 2022/2/25 | 2022/2/24 | 2022/3/24 16:10 | 27 |
| BA.2 | 277736 | male | 42 | Moderate | Omicron (BA.2) | 2022/2/25 | 2022/2/23 | 2022/3/ 18 20:30 | 21 |
| BA.2 | 277738 | female | 57 | Mild | Omicron (BA.2) | 2022/2/25 | 2022/2/22 | 2022/3/ 18 10:00 | 21 |
| BA.2 | 277739 | male | 15 | Moderate | Omicron (BA.2) | 2022/2/25 | 2022/2/23 | 2022/3/ 12 11:27 | 15 |
| BA.2 | 277740 | male | 54 | Mild | Omicron (BA.2) | 2022/2/25 | 2022/2/24 | 2022/3/ 18 10:00 | 21 |
| BA.2 | 277741 | male | 48 | Mild | Omicron (BA.2) | 2022/2/25 | 2022/2/23 | 2022/3/ 18 20:30 | 21 |
| BA.2 | 277742 | female | 42 | Mild | Omicron (BA.2) | 2022/2/25 | 2022/2/22 | 2022/3/ 12 8:59 | 14 |
| BA.2 | 277743 | female | 50 | Moderate | Omicron (BA.2) | 2022/2/25 | 2022/2/23 | 2022/3/ 18 20:30 | 21 |
| BA.2 | 277744 | male | 55 | Asymptomatic | Omicron (BA.2) | 2022/2/25 | 2022/2/25 | 2022/3/ 18 20:30 | 21 |
| BA.2 | 277747 | male | 22 | Mild | Omicron (BA.2) | 2022/2/25 | 2022/2/23 | 2022/3/ 18 18:16 | 21 |
| BA.2 | 277754 | female | 48 | Asymptomatic | Omicron (BA.2) | 2022/2/25 | 2022/2/24 | 2022/3/20 10:33 | 22 |
| BA.2 | 277756 | male | 84 | Mild | Omicron (BA.2) | 2022/2/26 | 2022/2/25 | 2022/3/20 19:57 | 23 |
| BA.2 | 277758 | female | 53 | Mild | Omicron (BA.2) | 2022/2/26 | 2022/2/24 | 2022/3/ 18 15:56 | 21 |
| BA.2 | 277759 | female | 29 | Mild | Omicron (BA.2) | 2022/2/26 | 2022/2/23 | 2022/3/ 18 15:56 | 21 |
| BA.2 | 277761 | male | 59 | Moderate | Omicron (BA.2) | 2022/2/26 | 2022/2/25 | 2022/3/ 17 21:09 | 20 |
| BA.2 | 277762 | male | 51 | Mild | Omicron (BA.2) | 2022/2/26 | 2022/2/24 | 2022/3/ 18 13:01 | 20 |
| BA.2 | 277763 | male | 54 | Mild | Omicron (BA.2) | 2022/2/26 | 2022/2/25 | 2022/3/ 16 15:51 | 19 |
| BA.2 | 277764 | male | 33 | Mild | Omicron (BA.2) | 2022/2/26 | 2022/2/24 | 2022/3/ 17 21:47 | 20 |
| BA.2 | 277768 | female | 26 | Mild | Omicron (BA.2) | 2022/2/26 | 2022/2/24 | 2022/3/ 13 10:12 | 15 |
| BA.2 | 277770 | male | 33 | Asymptomatic | Omicron (BA.2) | 2022/2/26 | 2022/2/25 | 2022/3/ 18 17:42 | 21 |
| BA.2 | 277774 | male | 30 | Moderate | Omicron (BA.2) | 2022/2/26 | 2022/2/24 | 2022/3/ 16 18:59 | 19 |
| BA.2 | 277784 | female | 75 | Asymptomatic | Omicron (BA.2) | 2022/2/26 | 2022/2/25 | 2022/3/ 18 14:27 | 20 |
| BA.2 | 277786 | male | 32 | Asymptomatic | Omicron (BA.2) | 2022/2/26 | 2022/2/25 | 2022/3/ 18 14:27 | 20 |
| BA.2 | 277814 | female | 43 | Mild | Omicron (BA.2) | 2022/2/26 | 2022/2/24 | 2022/3/ 19 10:46 | 21 |
| BA.2 | 277815 | male | 43 | Asymptomatic | Omicron (BA.2) | 2022/2/26 | 2022/2/26 | 2022/3/ 19 10:42 | 21 |
| BA.2 | 277822 | female | 2 | Mild | Omicron (BA.2) | 2022/2/26 | 2022/2/25 | 2022/3/ 16 19:00 | 18 |
| BA.2 | 277825 | male | 76 | Asymptomatic | Omicron (BA.2) | 2022/2/26 | 2022/2/23 | 2022/3/ 18 19:51 | 20 |
| BA.2 | 277826 | male | 45 | Mild | Omicron (BA.2) | 2022/2/26 | 2022/2/25 | 2022/3/ 18 18:36 | 20 |
| BA.2 | 277828 | female | 59 | Moderate | Omicron (BA.2) | 2022/2/26 | 2022/2/24 | 2022/3/ 18 17:46 | 20 |
| BA.2 | 277829 | male | 43 | Mild | Omicron (BA.2) | 2022/2/26 | 2022/2/25 | 2022/3/20 19:50 | 22 |

| BA.2 | 277833 | female | 65 | Mild | Omicron (BA.2) | 2022/2/26 | 2022/2/23 | 2022/3/ 18 14:21 | 20 |
| --- | --- | --- | --- | --- | --- | --- | --- | --- | --- |
| BA.2 | 277834 | male | 32 | Mild | Omicron (BA.2) | 2022/2/26 | 2022/2/25 | 2022/3/ 18 14:24 | 20 |
| BA.2 | 277837 | male | 28 | Mild | Omicron (BA.2) | 2022/2/26 | 2022/2/25 | 2022/3/ 16 17:49 | 18 |
| BA.2 | 277851 | female | 51 | Mild | Omicron (BA.2) | 2022/2/26 | 2022/2/24 | 2022/3/21 14:25 | 23 |
| BA.2 | 277852 | female | 27 | Mild | Omicron (BA.2) | 2022/2/26 | 2022/2/24 | 2022/3/ 18 17:32 | 20 |
| BA.2 | 277853 | female | 35 | Moderate | Omicron (BA.2) | 2022/2/26 | 2022/2/25 | 2022/3/20 19:54 | 22 |
| BA.2 | 277854 | female | 1 | Moderate | Omicron (BA.2) | 2022/2/26 | 2022/2/23 | 2022/3/ 16 14:42 | 18 |
| BA.2 | 277857 | male | 4 | Mild | Omicron (BA.2) | 2022/2/26 | 2022/2/25 | 2022/3/ 18 14:25 | 20 |
| BA.2 | 277858 | female | 6 | Mild | Omicron (BA.2) | 2022/2/26 | 2022/2/25 | 2022/3/ 18 14:26 | 20 |
| BA.2 | 277859 | female | 34 | Mild | Omicron (BA.2) | 2022/2/26 | 2022/2/25 | 2022/3/ 18 12:21 | 20 |
| BA.2 | 277860 | male | 8 | Mild | Omicron (BA.2) | 2022/2/26 | 2022/2/25 | 2022/3/ 16 17:56 | 18 |
| BA.2 | 277861 | male | 35 | Mild | Omicron (BA.2) | 2022/2/26 | 2022/2/25 | 2022/3/ 17 21:15 | 19 |
| BA.2 | 277863 | female | 48 | Mild | Omicron (BA.2) | 2022/2/26 | 2022/2/24 | 2022/3/20 14:32 | 22 |
| BA.2 | 277864 | female | 15 | Mild | Omicron (BA.2) | 2022/2/26 | 2022/2/25 | 2022/3/ 18 14:29 | 20 |
| BA.2 | 277871 | male | 31 | Moderate | Omicron (BA.2) | 2022/2/26 | 2022/2/25 | 2022/3/ 17 21:00 | 19 |
| BA.2 | 277873 | male | 38 | Mild | Omicron (BA.2) | 2022/2/26 | 2022/2/24 | 2022/3/ 18 9:31 | 19 |
| BA.2 | 277877 | male | 34 | Mild | Omicron (BA.2) | 2022/2/26 | 2022/2/25 | 2022/3/20 11:00 | 21 |
| BA.2 | 277879 | male | 10 | Mild | Omicron (BA.2) | 2022/2/26 | 2022/2/25 | 2022/3/ 18 16:55 | 20 |
| BA.2 | 277880 | female | 35 | Moderate | Omicron (BA.2) | 2022/2/26 | 2022/2/25 | 2022/3/ 18 14:30 | 20 |
| BA.2 | 277881 | male | 39 | Mild | Omicron (BA.2) | 2022/2/26 | 2022/2/23 | 2022/3/8 10:15 | 9 |
| BA.2 | 277882 | male | 68 | Mild | Omicron (BA.2) | 2022/2/26 | 2022/2/26 | 2022/3/ 18 11:27 | 20 |
| BA.2 | 277883 | female | 23 | Mild | Omicron (BA.2) | 2022/2/26 | 2022/2/25 | 2022/3/ 17 19:20 | 19 |
| BA.2 | 277884 | male | 88 | Mild | Omicron (BA.2) | 2022/2/26 | 2022/2/25 | 2022/3/20 16:54 | 22 |
| BA.2 | 277886 | female | 63 | Asymptomatic | Omicron (BA.2) | 2022/2/26 | 2022/2/25 | 2022/3/ 18 16:45 | 20 |
| BA.2 | 277890 | female | 32 | Mild | Omicron (BA.2) | 2022/2/27 | 2022/2/26 | 2022/3/23 10:43 | 24 |
| BA.2 | 277891 | female | 31 | Mild | Omicron (BA.2) | 2022/2/27 | 2022/2/26 | 2022/3/ 19 15:47 | 21 |
| BA.2 | 277892 | male | 10 | Moderate | Omicron (BA.2) | 2022/2/27 | 2022/2/26 | 2022/3/ 18 14:34 | 20 |
| BA.2 | 277893 | male | 2 | Mild | Omicron (BA.2) | 2022/2/27 | 2022/2/25 | 2022/3/ 19 15:49 | 21 |
| BA.2 | 277894 | male | 27 | Mild | Omicron (BA.2) | 2022/2/27 | 2022/2/26 | 2022/3/ 18 16:59 | 20 |
| BA.2 | 277895 | male | 20 | Mild | Omicron (BA.2) | 2022/2/27 | 2022/2/27 | 2022/3/23 11:44 | 24 |
| BA.2 | 277897 | male | 20 | Mild | Omicron (BA.2) | 2022/2/27 | 2022/2/25 | 2022/3/23 11:44 | 24 |
| BA.2 | 277898 | male | 20 | Mild | Omicron (BA.2) | 2022/2/27 | 2022/2/24 | 2022/3/25 10:09 | 26 |
| BA.2 | 277900 | female | 12 | Mild | Omicron (BA.2) | 2022/2/27 | 2022/2/26 | 2022/3/28 15:55 | 30 |
| BA.2 | 277901 | male | 42 | Mild | Omicron (BA.2) | 2022/2/27 | 2022/2/25 | 2022/3/ 18 19:43 | 20 |
| BA.2 | 277902 | male | 59 | Mild | Omicron (BA.2) | 2022/2/27 | 2022/2/24 | 2022/3/ 16 18:43 | 18 |
| BA.2 | 277904 | male | 20 | Mild | Omicron (BA.2) | 2022/2/27 | 2022/2/23 | 2022/3/23 11:43 | 24 |
| BA.2 | 277908 | male | 20 | Asymptomatic | Omicron (BA.2) | 2022/2/27 | 2022/2/26 | 2022/3/21 10:39 | 22 |
| BA.2 | 277911 | male | 36 | Mild | Omicron (BA.2) | 2022/2/27 | 2022/2/24 | 2022/3/ 18 19:09 | 20 |

| BA.2 | 277913 | male | 61 | Mild | Omicron (BA.2) | 2022/2/27 | 2022/2/26 | 2022/3/ 18 17:00 | 20 |
| --- | --- | --- | --- | --- | --- | --- | --- | --- | --- |
| BA.2 | 277914 | male | 49 | Mild | Omicron (BA.2) | 2022/2/27 | 2022/2/25 | 2022/3/ 17 19:12 | 19 |
| BA.2 | 277915 | male | 14 | Mild | Omicron (BA.2) | 2022/2/27 | 2022/2/26 | 2022/3/20 10:46 | 21 |
| BA.2 | 277916 | female | 53 | Mild | Omicron (BA.2) | 2022/2/27 | 2022/2/26 | 2022/3/ 18 16:05 | 19 |
| BA.2 | 277917 | male | 31 | Mild | Omicron (BA.2) | 2022/2/27 | 2022/2/24 | 2022/3/ 18 15:51 | 19 |
| BA.2 | 277920 | male | 26 | Moderate | Omicron (BA.2) | 2022/2/27 | 2022/2/26 | 2022/3/ 17 11:34 | 18 |
| BA.2 | 277921 | male | 26 | Mild | Omicron (BA.2) | 2022/2/27 | 2022/2/26 | 2022/3/ 18 13:02 | 19 |
| BA.2 | 277922 | male | 35 | Moderate | Omicron (BA.2) | 2022/2/27 | 2022/2/25 | 2022/3/ 18 18:56 | 20 |
| BA.2 | 277923 | female | 57 | Asymptomatic | Omicron (BA.2) | 2022/2/27 | 2022/2/26 | 2022/3/20 19:29 | 21 |
| BA.2 | 277924 | female | 83 | Asymptomatic | Omicron (BA.2) | 2022/2/27 | 2022/2/25 | 2022/3/23 20:47 | 25 |
| BA.2 | 277934 | male | 72 | Mild | Omicron (BA.2) | 2022/2/27 | 2022/2/26 | 2022/3/25 10:15 | 26 |
| BA.2 | 277947 | female | 47 | Mild | Omicron (BA.2) | 2022/2/27 | 2022/2/24 | 2022/3/ 18 16:12 | 19 |
| BA.2 | 277950 | female | 36 | Mild | Omicron (BA.2) | 2022/2/27 | 2022/2/24 | 2022/3/ 18 15:47 | 19 |
| BA.2 | 277951 | male | 3 | Mild | Omicron (BA.2) | 2022/2/27 | 2022/2/26 | 2022/3/ 18 15:57 | 19 |
| BA.2 | 277952 | female | 7 | Mild | Omicron (BA.2) | 2022/2/27 | 2022/2/26 | 2022/3/ 18 15:47 | 19 |
| BA.2 | 277954 | male | 41 | Mild | Omicron (BA.2) | 2022/2/27 | 2022/2/27 | 2022/3/ 18 11:03 | 19 |
| BA.2 | 277961 | female | 49 | Moderate | Omicron (BA.2) | 2022/2/27 | 2022/2/26 | 2022/3/ 18 14:29 | 19 |
| BA.2 | 277962 | male | 23 | Mild | Omicron (BA.2) | 2022/2/27 | 2022/2/25 | 2022/3/ 18 14:56 | 19 |
| BA.2 | 277965 | female | 2 | Mild | Omicron (BA.2) | 2022/2/27 | 2022/2/26 | 2022/3/ 18 14:21 | 19 |
| BA.2 | 277966 | male | 41 | Mild | Omicron (BA.2) | 2022/2/27 | 2022/2/26 | 2022/3/21 10:56 | 22 |
| BA.2 | 277968 | female | 14 | Mild | Omicron (BA.2) | 2022/2/27 | 2022/2/26 | 2022/3/ 18 15:42 | 19 |
| BA.2 | 277970 | male | 20 | Mild | Omicron (BA.2) | 2022/2/27 | 2022/2/22 | 2022/3/20 12:13 | 21 |
| BA.2 | 277974 | male | 20 | Mild | Omicron (BA.2) | 2022/2/27 | 2022/2/24 | 2022/3/26 8:48 | 27 |
| BA.2 | 277977 | male | 20 | Mild | Omicron (BA.2) | 2022/2/27 | 2022/2/24 | 2022/3/ 19 12:01 | 20 |
| BA.2 | 277981 | female | 13 | Mild | Omicron (BA.2) | 2022/2/27 | 2022/2/26 | 2022/3/ 17 19:14 | 18 |
| BA.2 | 277982 | female | 11 | Mild | Omicron (BA.2) | 2022/2/27 | 2022/2/26 | 2022/3/ 18 14:23 | 19 |
| BA.2 | 277986 | male | 38 | Mild | Omicron (BA.2) | 2022/2/27 | 2022/2/26 | 2022/3/ 19 17:14 | 20 |
| BA.2 | 277987 | female | 15 | Moderate | Omicron (BA.2) | 2022/2/27 | 2022/2/26 | 2022/3/ 18 11:06 | 19 |
| BA.2 | 277988 | female | 34 | Mild | Omicron (BA.2) | 2022/2/27 | 2022/2/26 | 2022/3/20 22:02 | 21 |
| BA.2 | 277991 | male | 46 | Moderate | Omicron (BA.2) | 2022/2/27 | 2022/2/26 | 2022/3/ 19 18:32 | 20 |
| BA.2 | 277993 | female | 29 | Moderate | Omicron (BA.2) | 2022/2/27 | 2022/2/26 | 2022/3/22 12:38 | 23 |
| BA.2 | 277996 | male | 24 | Mild | Omicron (BA.2) | 2022/2/27 | 2022/2/26 | 2022/3/ 17 19:11 | 18 |
| BA.2 | 277997 | male | 60 | Mild | Omicron (BA.2) | 2022/2/27 | 2022/2/26 | 2022/3/ 18 17:16 | 19 |
| BA.2 | 278001 | male | 68 | Mild | Omicron (BA.2) | 2022/2/27 | 2022/2/25 | 2022/3/ 15 10:34 | 16 |
| BA.2 | 278003 | male | 5 | Mild | Omicron (BA.2) | 2022/2/27 | 2022/2/26 | 2022/3/ 16 9:00 | 17 |
| BA.2 | 278004 | male | 6 | Mild | Omicron (BA.2) | 2022/2/27 | 2022/2/26 | 2022/3/ 18 16:12 | 19 |
| BA.2 | 278008 | female | 42 | Moderate | Omicron (BA.2) | 2022/2/27 | 2022/2/27 | 2022/3/ 18 17:04 | 19 |
| BA.2 | 278009 | male | 3 | Mild | Omicron (BA.2) | 2022/2/27 | 2022/2/27 | 2022/3/22 18:08 | 23 |

| BA.2 | 278010 | female | 45 | Mild | Omicron (BA.2) | 2022/2/27 | 2022/2/26 | 2022/3/24 9:44 | 25 |
| --- | --- | --- | --- | --- | --- | --- | --- | --- | --- |
| BA.2 | 278013 | male | 52 | Mild | Omicron (BA.2) | 2022/2/27 | 2022/2/25 | 2022/3/24 13:45 | 25 |
| BA.2 | 278014 | female | 38 | Mild | Omicron (BA.2) | 2022/2/27 | 2022/2/26 | 2022/3/ 18 16:46 | 19 |
| BA.2 | 278015 | male | 58 | Moderate | Omicron (BA.2) | 2022/2/27 | 2022/2/26 | 2022/3/20 12:06 | 21 |
| BA.2 | 278017 | male | 16 | Mild | Omicron (BA.2) | 2022/2/27 | 2022/2/26 | 2022/3/ 16 8:59 | 16 |
| BA.2 | 278019 | female | 15 | Mild | Omicron (BA.2) | 2022/2/27 | 2022/2/25 | 2022/3/ 15 10:32 | 16 |
| BA.2 | 278020 | male | 7 | Asymptomatic | Omicron (BA.2) | 2022/2/27 | 2022/2/26 | 2022/3/ 18 16:38 | 19 |
| BA.2 | 278021 | male | 4 | Mild | Omicron (BA.2) | 2022/2/27 | 2022/2/26 | 2022/3/ 19 10:32 | 19 |
| BA.2 | 278023 | male | 25 | Mild | Omicron (BA.2) | 2022/2/27 | 2022/2/25 | 2022/3/23 11:43 | 24 |
| BA.2 | 278024 | male | 27 | Mild | Omicron (BA.2) | 2022/2/27 | 2022/2/26 | 2022/3/23 11:45 | 24 |
| BA.2 | 278026 | female | 27 | Mild | Omicron (BA.2) | 2022/2/27 | 2022/2/26 | 2022/3/ 18 17:05 | 19 |
| BA.2 | 278029 | female | 26 | Mild | Omicron (BA.2) | 2022/2/28 | 2022/2/26 | 2022/3/ 18 20:05 | 19 |
| BA.2 | 278036 | female | 42 | Mild | Omicron (BA.2) | 2022/2/28 | 2022/2/27 | 2022/3/ 18 19:07 | 19 |
| BA.2 | 278037 | male | 25 | Mild | Omicron (BA.2) | 2022/2/28 | 2022/2/27 | 2022/3/22 10:31 | 22 |
| BA.2 | 278038 | female | 58 | Asymptomatic | Omicron (BA.2) | 2022/2/28 | 2022/2/27 | 2022/3/ 18 14:39 | 18 |
| BA.2 | 278039 | female | 13 | Mild | Omicron (BA.2) | 2022/2/28 | 2022/2/27 | 2022/3/ 18 15:47 | 18 |
| BA.2 | 278056 | female | 38 | Moderate | Omicron (BA.2) | 2022/2/28 | 2022/2/27 | 2022/3/22 9:10 | 22 |
| BA.2 | 278063 | male | 4 | Mild | Omicron (BA.2) | 2022/2/28 | 2022/2/27 | 2022/3/ 18 15:42 | 18 |
| BA.2 | 278076 | female | 3 | Mild | Omicron (BA.2) | 2022/2/28 | 2022/2/27 | 2022/3/ 16 19:35 | 16 |
| BA.2 | 278078 | female | 25 | Mild | Omicron (BA.2) | 2022/2/28 | 2022/2/27 | 2022/3/ 18 15:55 | 18 |
| BA.2 | 278082 | female | 41 | Mild | Omicron (BA.2) | 2022/2/28 | 2022/2/26 | 2022/3/ 18 18:11 | 18 |
| BA.2 | 278089 | male | 20 | Mild | Omicron (BA.2) | 2022/2/28 | 2022/2/24 | 2022/3/23 11:40 | 23 |
| BA.2 | 278093 | male | 62 | Moderate | Omicron (BA.2) | 2022/2/28 | 2022/2/25 | 2022/3/20 15:21 | 20 |
| BA.2 | 278097 | female | 47 | Mild | Omicron (BA.2) | 2022/2/28 | 2022/2/27 | 2022/3/21 9:34 | 21 |
| BA.2 | 278101 | female | 1 | Mild | Omicron (BA.2) | 2022/2/28 | 2022/2/27 | 2022/3/ 16 18:22 | 16 |
| BA.2 | 278102 | male | 28 | Moderate | Omicron (BA.2) | 2022/2/28 | 2022/2/25 | 2022/3/21 9:34 | 21 |
| BA.2 | 278115 | male | 6 | Mild | Omicron (BA.2) | 2022/2/28 | 2022/2/27 | 2022/3/ 18 15:55 | 18 |
| BA.2 | 278116 | female | 32 | Mild | Omicron (BA.2) | 2022/2/28 | 2022/2/25 | 2022/3/ 18 15:55 | 18 |
| BA.2 | 278117 | male | 21 | Moderate | Omicron (BA.2) | 2022/2/28 | 2022/2/27 | 2022/3/ 19 11:00 | 19 |
| BA.2 | 278118 | female | 39 | Mild | Omicron (BA.2) | 2022/2/28 | 2022/2/25 | 2022/3/ 18 16:14 | 18 |
| BA.2 | 278119 | male | 30 | Mild | Omicron (BA.2) | 2022/2/28 | 2022/2/27 | 2022/3/ 18 13:41 | 18 |
| BA.2 | 278120 | male | 30 | Mild | Omicron (BA.2) | 2022/2/28 | 2022/2/27 | 2022/3/ 18 16:39 | 18 |
| BA.2 | 278121 | male | 35 | Mild | Omicron (BA.2) | 2022/2/28 | 2022/2/27 | 2022/3/ 19 10:39 | 19 |
| BA.2 | 278122 | female | 42 | Asymptomatic | Omicron (BA.2) | 2022/2/28 | 2022/2/28 | 2022/3/ 19 10:12 | 19 |
| BA.2 | 278126 | male | 30 | Mild | Omicron (BA.2) | 2022/2/28 | 2022/2/27 | 2022/3/ 18 15:55 | 18 |
| BA.2 | 278128 | female | 54 | Mild | Omicron (BA.2) | 2022/2/28 | 2022/2/26 | 2022/3/ 19 11:00 | 19 |
| BA.2 | 278129 | male | 54 | Mild | Omicron (BA.2) | 2022/2/28 | 2022/2/26 | 2022/3/ 19 11:00 | 19 |
| BA.2 | 278131 | male | 24 | Mild | Omicron (BA.2) | 2022/2/28 | 2022/2/28 | 2022/3/23 11:42 | 23 |

| BA.2 | 278132 | male | 24 | Mild | Omicron (BA.2) | 2022/2/28 | 2022/2/25 | 2022/3/22 12:47 | 22 |
| --- | --- | --- | --- | --- | --- | --- | --- | --- | --- |
| BA.2 | 278133 | male | 26 | Asymptomatic | Omicron (BA.2) | 2022/2/28 | 2022/2/28 | 2022/3/ 17 13:42 | 17 |
| BA.2 | 278135 | male | 21 | Asymptomatic | Omicron (BA.2) | 2022/2/28 | 2022/2/28 | 2022/3/24 12:14 | 24 |
| BA.2 | 278165 | male | 31 | Mild | Omicron (BA.2) | 2022/2/28 | 2022/2/26 | 2022/3/ 16 18:14 | 16 |
| BA.2 | 278166 | male | 11 | Mild | Omicron (BA.2) | 2022/3/ 1 | 2022/2/27 | 2022/3/ 19 20:16 | 19 |
| BA.2 | 278170 | female | 34 | Mild | Omicron (BA.2) | 2022/3/ 1 | 2022/2/27 | 2022/3/ 18 9:32 | 17 |
| BA.2 | 278179 | male | 53 | Mild | Omicron (BA.2) | 2022/3/ 1 | 2022/2/26 | 2022/3/24 16:05 | 23 |
| BA.2 | 278183 | female | 6 | Mild | Omicron (BA.2) | 2022/3/ 1 | 2022/2/27 | 2022/3/20 10:32 | 19 |
| BA.2 | 278196 | female | 43 | Asymptomatic | Omicron (BA.2) | 2022/3/ 1 | 2022/2/28 | 2022/3/ 16 12:17 | 15 |
| BA.2 | 278198 | female | 28 | Moderate | Omicron (BA.2) | 2022/3/ 1 | 2022/2/27 | 2022/3/ 15 12:02 | 14 |
| BA.2 | 278200 | male | 46 | Asymptomatic | Omicron (BA.2) | 2022/3/ 1 | 2022/2/28 | 2022/3/ 18 14:22 | 17 |
| BA.2 | 278201 | female | 47 | Asymptomatic | Omicron (BA.2) | 2022/3/ 1 | 2022/2/28 | 2022/3/ 18 14:23 | 17 |
| BA.2 | 278202 | male | 12 | Asymptomatic | Omicron (BA.2) | 2022/3/ 1 | 2022/2/28 | 2022/3/ 18 16:58 | 17 |
| BA.2 | 278203 | female | 9 | Mild | Omicron (BA.2) | 2022/3/ 1 | 2022/2/28 | 2022/3/22 10:47 | 21 |
| BA.2 | 278210 | female | 43 | Mild | Omicron (BA.2) | 2022/3/ 1 | 2022/2/28 | 2022/3/20 19:57 | 19 |
| BA.2 | 278233 | female | 50 | Mild | Omicron (BA.2) | 2022/3/ 1 | 2022/2/28 | 2022/3/21 17:10 | 20 |
| BA.2 | 278235 | female | 32 | Asymptomatic | Omicron (BA.2) | 2022/3/ 1 | 2022/2/27 | 2022/3/ 18 16:10 | 17 |
| BA.2 | 278246 | male | 46 | Mild | Omicron (BA.2) | 2022/3/ 1 | 2022/2/28 | 2022/3/31 10:14 | 30 |
| BA.2 | 278249 | female | 30 | Mild | Omicron (BA.2) | 2022/3/ 1 | 2022/2/28 | 2022/3/22 10:18 | 21 |
| BA.2 | 278254 | female | 49 | Mild | Omicron (BA.2) | 2022/3/ 1 | 2022/2/28 | 2022/3/ 19 10:46 | 18 |
| BA.2 | 278255 | male | 24 | Mild | Omicron (BA.2) | 2022/3/ 1 | 2022/2/27 | 2022/3/31 9:52 | 30 |
| BA.2 | 278256 | male | 22 | Mild | Omicron (BA.2) | 2022/3/ 1 | 2022/2/28 | 2022/3/24 16:04 | 23 |
| BA.2 | 278257 | male | 45 | Mild | Omicron (BA.2) | 2022/3/ 1 | 2022/2/27 | 2022/3/ 18 16:36 | 17 |
| BA.2 | 278258 | male | 48 | Mild | Omicron (BA.2) | 2022/3/ 1 | 2022/2/28 | 2022/3/ 18 18:44 | 17 |
| BA.2 | 278259 | male | 32 | Mild | Omicron (BA.2) | 2022/3/ 1 | 2022/2/28 | 2022/3/ 18 18:49 | 17 |
| BA.2 | 278260 | female | 23 | Mild | Omicron (BA.2) | 2022/3/ 1 | 2022/2/28 | 2022/3/23 20:14 | 22 |
| BA.2 | 278261 | male | 37 | Mild | Omicron (BA.2) | 2022/3/ 1 | 2022/2/27 | 2022/3/20 15:22 | 19 |
| BA.2 | 278263 | female | 27 | Mild | Omicron (BA.2) | 2022/3/ 1 | 2022/2/28 | 2022/3/21 13:56 | 20 |
| BA.2 | 278264 | female | 62 | Mild | Omicron (BA.2) | 2022/3/ 1 | 2022/2/26 | 2022/3/ 19 11:09 | 18 |
| BA.2 | 278266 | male | 32 | Asymptomatic | Omicron (BA.2) | 2022/3/2 | 2022/2/28 | 2022/3/ 19 10:41 | 17 |
| BA.2 | 278269 | female | 40 | Mild | Omicron (BA.2) | 2022/3/ 1 | 2022/2/28 | 2022/3/22 10:39 | 21 |
| BA.2 | 278270 | female | 36 | Mild | Omicron (BA.2) | 2022/3/ 1 | 2022/2/28 | 2022/3/22 9:09 | 21 |
| BA.2 | 278280 | male | 44 | Moderate | Omicron (BA.2) | 2022/3/ 1 | 2022/2/28 | 2022/3/ 18 22:29 | 17 |
| BA.2 | 278281 | male | 23 | Mild | Omicron (BA.2) | 2022/3/ 1 | 2022/2/28 | 2022/3/28 10:17 | 27 |
| BA.2 | 278283 | male | 81 | Mild | Omicron (BA.2) | 2022/3/ 1 | 2022/2/28 | 2022/3/20 17:07 | 19 |
| BA.2 | 278286 | male | 45 | Mild | Omicron (BA.2) | 2022/3/ 1 | 2022/2/27 | 2022/3/ 18 22:30 | 17 |
| BA.2 | 278293 | female | 13 | Moderate | Omicron (BA.2) | 2022/3/ 1 | 2022/2/27 | 2022/3/ 16 19:40 | 15 |
| BA.2 | 278294 | female | 47 | Moderate | Omicron (BA.2) | 2022/3/ 1 | 2022/2/28 | 2022/3/ 18 8:19 | 16 |

| BA.2 | 278297 | male | 21 | Mild | Omicron (BA.2) | 2022/3/ 1 | 2022/2/25 | 2022/3/23 11:42 | 22 |
| --- | --- | --- | --- | --- | --- | --- | --- | --- | --- |
| BA.2 | 278298 | male | 32 | Mild | Omicron (BA.2) | 2022/3/ 1 | 2022/2/28 | 2022/3/24 12:05 | 23 |
| BA.2 | 278303 | male | 31 | Mild | Omicron (BA.2) | 2022/3/ 1 | 2022/2/26 | 2022/3/20 12:13 | 19 |
| BA.2 | 278304 | male | 23 | Mild | Omicron (BA.2) | 2022/3/ 1 | 2022/3/ 1 | 2022/3/24 12:04 | 23 |
| BA.2 | 278307 | male | 21 | Mild | Omicron (BA.2) | 2022/3/ 1 | 2022/2/27 | 2022/3/23 11:44 | 22 |
| BA.2 | 278308 | male | 25 | Mild | Omicron (BA.2) | 2022/3/ 1 | 2022/2/26 | 2022/3/ 19 12:03 | 18 |
| BA.2 | 278312 | male | 20 | Mild | Omicron (BA.2) | 2022/3/ 1 | 2022/2/28 | 2022/3/23 11:43 | 22 |
| BA.2 | 278315 | male | 25 | Mild | Omicron (BA.2) | 2022/3/ 1 | 2022/2/28 | 2022/3/23 11:42 | 22 |
| BA.2 | 278317 | female | 29 | Mild | Omicron (BA.2) | 2022/3/ 1 | 2022/2/28 | 2022/3/ 18 14:25 | 17 |
| BA.2 | 278318 | male | 9 | Moderate | Omicron (BA.2) | 2022/3/ 1 | 2022/2/28 | 2022/3/ 19 14:36 | 18 |
| BA.2 | 278319 | male | 6 | Mild | Omicron (BA.2) | 2022/3/ 1 | 2022/2/28 | 2022/3/ 18 14:25 | 17 |
| BA.2 | 278320 | female | 12 | Mild | Omicron (BA.2) | 2022/3/ 1 | 2022/3/ 1 | 2022/3/ 18 16:50 | 17 |
| BA.2 | 278328 | female | 52 | Mild | Omicron (BA.2) | 2022/3/ 1 | 2022/3/ 1 | 2022/3/22 16:58 | 21 |
| BA.2 | 278333 | female | 47 | Mild | Omicron (BA.2) | 2022/3/2 | 2022/3/ 1 | 2022/3/21 11:04 | 19 |
| BA.2 | 278337 | male | 47 | Mild | Omicron (BA.2) | 2022/3/2 | 2022/2/28 | 2022/3/21 11:00 | 19 |
| BA.2 | 278339 | female | 26 | Mild | Omicron (BA.2) | 2022/3/2 | 2022/3/ 1 | 2022/3/ 18 10:43 | 16 |
| BA.2 | 278343 | male | 10 | Mild | Omicron (BA.2) | 2022/3/2 | 2022/3/ 1 | 2022/3/ 17 15:28 | 15 |
| BA.2 | 278347 | male | 79 | Mild | Omicron (BA.2) | 2022/3/2 | 2022/2/28 | 2022/3/21 10:02 | 19 |
| BA.2 | 278349 | female | 41 | Mild | Omicron (BA.2) | 2022/3/2 | 2022/2/28 | 2022/3/ 18 13:25 | 16 |
| BA.2 | 278352 | female | 37 | Moderate | Omicron (BA.2) | 2022/3/2 | 2022/3/ 1 | 2022/3/ 19 10:40 | 17 |
| BA.2 | 278353 | male | 11 | Moderate | Omicron (BA.2) | 2022/3/2 | 2022/3/2 | 2022/3/21 3:42 | 19 |
| BA.2 | 278360 | female | 51 | Mild | Omicron (BA.2) | 2022/3/2 | 2022/3/ 1 | 2022/3/ 18 10:03 | 16 |
| BA.2 | 278361 | female | 57 | Mild | Omicron (BA.2) | 2022/3/2 | 2022/3/ 1 | 2022/3/ 18 16:00 | 16 |
| BA.2 | 278363 | female | 47 | Mild | Omicron (BA.2) | 2022/3/2 | 2022/3/ 1 | 2022/3/ 18 18:14 | 16 |
| BA.2 | 278367 | female | 48 | Moderate | Omicron (BA.2) | 2022/3/2 | 2022/2/28 | 2022/3/23 15:53 | 21 |
| BA.2 | 278368 | male | 13 | Mild | Omicron (BA.2) | 2022/3/2 | 2022/2/28 | 2022/3/ 18 15:42 | 16 |
| BA.2 | 278369 | male | 11 | Mild | Omicron (BA.2) | 2022/3/2 | 2022/2/28 | 2022/3/ 18 15:45 | 16 |
| BA.2 | 278371 | male | 49 | Mild | Omicron (BA.2) | 2022/3/2 | 2022/2/28 | 2022/3/21 11:24 | 19 |
| BA.2 | 278372 | female | 15 | Mild | Omicron (BA.2) | 2022/3/2 | 2022/2/28 | 2022/3/ 18 15:49 | 16 |
| BA.2 | 278373 | female | 29 | Mild | Omicron (BA.2) | 2022/3/2 | 2022/3/ 1 | 2022/3/21 12:11 | 19 |
| BA.2 | 278375 | female | 1 | Mild | Omicron (BA.2) | 2022/3/2 | 2022/3/ 1 | 2022/3/20 12:32 | 18 |
| BA.2 | 278378 | male | 11 | Mild | Omicron (BA.2) | 2022/3/2 | 2022/3/ 1 | 2022/3/21 15:02 | 19 |
| BA.2 | 278381 | female | 12 | Mild | Omicron (BA.2) | 2022/3/2 | 2022/3/ 1 | 2022/3/ 18 18:11 | 16 |
| BA.2 | 278393 | male | 3 | Mild | Omicron (BA.2) | 2022/3/2 | 2022/3/ 1 | 2022/3/ 16 14:32 | 14 |
| BA.2 | 278394 | female | 46 | Mild | Omicron (BA.2) | 2022/3/2 | 2022/3/ 1 | 2022/3/ 17 17:30 | 15 |
| BA.2 | 278396 | female | 57 | Moderate | Omicron (BA.2) | 2022/3/2 | 2022/3/ 1 | 2022/3/ 18 17:53 | 16 |
| BA.2 | 278397 | female | 32 | Mild | Omicron (BA.2) | 2022/3/2 | 2022/3/ 1 | 2022/3/ 18 15:46 | 16 |
| BA.2 | 278399 | female | 48 | Mild | Omicron (BA.2) | 2022/3/2 | 2022/3/ 1 | 2022/3/20 17:03 | 18 |

| BA.2 | 278400 | female | 42 | Mild | Omicron (BA.2) | 2022/3/2 | 2022/3/ 1 | 2022/3/21 15:10 | 19 |
| --- | --- | --- | --- | --- | --- | --- | --- | --- | --- |
| BA.2 | 278425 | male | 17 | Mild | Omicron (BA.2) | 2022/3/2 | 2022/3/ 1 | 2022/3/ 18 18:12 | 16 |
| BA.2 | 278426 | male | 15 | Mild | Omicron (BA.2) | 2022/3/2 | 2022/3/ 1 | 2022/3/22 16:15 | 20 |
| BA.2 | 278432 | female | 51 | Mild | Omicron (BA.2) | 2022/3/2 | 2022/3/ 1 | 2022/3/22 9:07 | 20 |
| BA.2 | 278434 | female | 11 | Mild | Omicron (BA.2) | 2022/3/2 | 2022/3/2 | 2022/3/ 18 12:20 | 16 |
| BA.2 | 278437 | male | 53 | Mild | Omicron (BA.2) | 2022/3/2 | 2022/3/ 1 | 2022/3/24 10:00 | 22 |
| BA.2 | 278438 | female | 53 | Moderate | Omicron (BA.2) | 2022/3/2 | 2022/3/ 1 | 2022/3/22 9:10 | 20 |
| BA.2 | 278440 | male | 25 | Moderate | Omicron (BA.2) | 2022/3/2 | 2022/3/ 1 | 2022/3/ 18 19:29 | 16 |
| BA.2 | 278441 | male | 58 | Mild | Omicron (BA.2) | 2022/3/2 | 2022/3/ 1 | 2022/3/25 11:15 | 23 |
| BA.2 | 278442 | female | 13 | Mild | Omicron (BA.2) | 2022/3/2 | 2022/3/ 1 | 2022/3/ 17 18:21 | 15 |
| BA.2 | 278443 | male | 8 | Mild | Omicron (BA.2) | 2022/3/2 | 2022/2/28 | 2022/3/ 18 14:30 | 16 |
| BA.2 | 278444 | female | 10 | Mild | Omicron (BA.2) | 2022/3/2 | 2022/3/ 1 | 2022/3/28 15:11 | 26 |
| BA.2 | 278445 | male | 10 | Mild | Omicron (BA.2) | 2022/3/2 | 2022/3/ 1 | 2022/3/21 16:00 | 19 |
| BA.2 | 278446 | male | 49 | Mild | Omicron (BA.2) | 2022/3/2 | 2022/3/ 1 | 2022/3/21 19:24 | 19 |
| BA.2 | 278454 | male | 12 | Mild | Omicron (BA.2) | 2022/3/2 | 2022/3/2 | 2022/3/ 18 15:54 | 16 |
| BA.2 | 278455 | female | 15 | Mild | Omicron (BA.2) | 2022/3/2 | 2022/3/ 1 | 2022/3/ 18 18:00 | 16 |
| BA.2 | 278456 | female | 16 | Mild | Omicron (BA.2) | 2022/3/2 | 2022/3/ 1 | 2022/3/24 15:42 | 22 |
| BA.2 | 278457 | male | 46 | Mild | Omicron (BA.2) | 2022/3/2 | 2022/3/ 1 | 2022/3/ 18 14:09 | 16 |
| BA.2 | 278458 | male | 52 | Mild | Omicron (BA.2) | 2022/3/3 | 2022/3/ 1 | 2022/3/28 9:45 | 25 |
| BA.2 | 278459 | male | 64 | Mild | Omicron (BA.2) | 2022/3/3 | 2022/3/ 1 | 2022/3/ 18 19:17 | 16 |
| BA.2 | 278461 | male | 11 | Mild | Omicron (BA.2) | 2022/3/3 | 2022/3/ 1 | 2022/3/ 19 11:11 | 16 |
| BA.2 | 278462 | female | 21 | Mild | Omicron (BA.2) | 2022/3/3 | 2022/3/2 | 2022/3/24 15:59 | 22 |
| BA.2 | 278463 | female | 45 | Mild | Omicron (BA.2) | 2022/3/3 | 2022/3/2 | 2022/3/ 17 13:27 | 14 |
| BA.2 | 278464 | male | 56 | Mild | Omicron (BA.2) | 2022/3/3 | 2022/3/2 | 2022/3/ 17 13:08 | 14 |
| BA.2 | 278465 | female | 42 | Mild | Omicron (BA.2) | 2022/3/3 | 2022/3/2 | 2022/3/ 19 18:07 | 17 |
| BA.2 | 278467 | female | 33 | Mild | Omicron (BA.2) | 2022/3/3 | 2022/3/ 1 | 2022/3/ 18 17:25 | 15 |
| BA.2 | 278468 | female | 47 | Mild | Omicron (BA.2) | 2022/3/3 | 2022/3/2 | 2022/4/4 11:00 | 32 |
| BA.2 | 278475 | male | 6 | Mild | Omicron (BA.2) | 2022/3/3 | 2022/3/2 | 2022/3/20 15:45 | 17 |
| BA.2 | 278478 | female | 4 | Asymptomatic | Omicron (BA.2) | 2022/3/3 | 2022/3/2 | 2022/3/ 18 19:56 | 15 |
| BA.2 | 278482 | female | 8 | Mild | Omicron (BA.2) | 2022/3/3 | 2022/3/2 | 2022/3/22 9:10 | 19 |
| BA.2 | 278489 | male | 40 | Mild | Omicron (BA.2) | 2022/3/3 | 2022/3/3 | 2022/3/ 19 9:58 | 16 |
| BA.2 | 278506 | female | 5 | Mild | Omicron (BA.2) | 2022/3/3 | 2022/3/2 | 2022/3/ 18 17:43 | 15 |
| BA.2 | 278514 | female | 14 | Mild | Omicron (BA.2) | 2022/3/3 | 2022/3/2 | 2022/3/21 19:04 | 18 |
| BA.2 | 278523 | female | 13 | Mild | Omicron (BA.2) | 2022/3/3 | 2022/3/2 | 2022/3/ 19 9:44 | 16 |
| BA.2 | 280497 | female | 50 | Asymptomatic | Omicron (BA.2) | 2022-04-01 15:58 | 2022/3/31 | 2022-04- 14 13:05 | 13 |
| BA.2 | 280502 | female | 24 | Asymptomatic | Omicron (BA.2) | 2022-04-01 21:02 | 2022/4/ 1 | 2022-04-24 12:20 | 23 |
| BA.2 | 280512 | male | 36 | Asymptomatic | Omicron (BA.2) | 2022-04-02 11:20 | 2022/4/ 1 | 2022-04- 11 11:41 | 9 |
| BA.2 | 280561 | male | 58 | Asymptomatic | Omicron (BA.2) | 2022-04-03 10:10 | 2022/4/2 | 2022-04- 15 9:54 | 12 |

| BA.2 | 280678 | female | 63 | Asymptomatic | Omicron (BA.2) | 2022-04-06 1:18 | 2022/4/4 | 2022-04-20 12:13 | 14 |
| --- | --- | --- | --- | --- | --- | --- | --- | --- | --- |
| BA.2 | 280694 | male | 34 | Asymptomatic | Omicron (BA.2) | 2022-04-06 23:04 | 2022/4/6 | 2022-04-21 9:42 | 14 |
| BA.2 | 280710 | female | 62 | Asymptomatic | Omicron (BA.2) | 2022-04-07 2:11 | 2022/4/6 | 2022-04- 13 13:29 | 6 |
| BA.2 | 280755 | male | 48 | Asymptomatic | Omicron (BA.2) | 2022-04-08 10:03 | 2022/4/7 | 2022-04-20 12:21 | 12 |
| BA.2 | 280795 | male | 34 | Moderate | Omicron (BA.2) | 2022-04-08 23:55 | 2022/4/7 | 2022-04-22 11:19 | 13 |
| BA.2 | 280800 | male | 59 | Asymptomatic | Omicron (BA.2) | 2022-04-09 8:02 | 2022/4/8 | 2022-04-24 9:18 | 15 |
| BA.2 | 280809 | male | 33 | Mild | Omicron (BA.2) | 2022-04-09 10:48 | 2022/4/8 | 2022-04- 18 11:03 | 9 |
| BA.2 | 280825 | female | 31 | Mild | Omicron (BA.2) | 2022-04-09 16:58 | 2022/4/8 | 2022-04-23 11:09 | 14 |
| BA.2 | 280835 | female | 12 | Asymptomatic | Omicron (BA.2) | 2022-04-09 20:47 | 2022/4/9 | 2022-04-23 9:44 | 14 |
| BA.2 | 280836 | female | 39 | Asymptomatic | Omicron (BA.2) | 2022-04-09 20:49 | 2022/4/9 | 2022-05-05 10:42 | 26 |
| BA.2 | 280850 | male | 24 | Mild | Omicron (BA.2) | 2022-04- 10 9:48 | 2022/4/9 | 2022-04-23 17:54 | 13 |
| BA.2 | 280851 | female | 42 | Asymptomatic | Omicron (BA.2) | 2022-04- 10 11:30 | 2022/4/9 | 2022-04-21 11:53 | 11 |
| BA.2 | 280935 | female | 28 | Asymptomatic | Omicron (BA.2) | 2022-04- 11 18:38 | 2022/4/ 11 | 2022-04-25 8:22 | 14 |
| BA.2 | 280961 | male | 24 | Asymptomatic | Omicron (BA.2) | 2022-04- 12 9:27 | 2022/4/ 11 | 2022-04-28 13:57 | 16 |
| BA.2 | 281022 | male | 27 | Mild | Omicron (BA.2) | 2022-04- 12 21:31 | 2022/4/ 12 | 2022-05-04 10:10 | 22 |
| BA.2 | 281023 | male | 21 | Asymptomatic | Omicron (BA.2) | 2022-04- 12 21:32 | 2022/4/ 12 | 2022-04-27 13:01 | 15 |
| BA.2 | 281024 | male | 27 | Mild | Omicron (BA.2) | 2022-04- 12 22:37 | 2022/4/ 11 | 2022-04-29 11:58 | 17 |
| BA.2 | 281025 | male | 38 | Asymptomatic | Omicron (BA.2) | 2022-04- 12 21:32 | 2022/4/ 12 | 2022-04-26 10:58 | 14 |
| BA.2 | 281028 | male | 34 | Asymptomatic | Omicron (BA.2) | 2022-04- 13 0:13 | 2022/4/ 12 | 2022-05-03 10:36 | 20 |
| BA.2 | 281034 | male | 25 | Mild | Omicron (BA.2) | 2022-04- 13 7:43 | 2022/4/ 12 | 2022-04-27 19:19 | 14 |
| BA.2 | 281035 | male | 45 | Asymptomatic | Omicron (BA.2) | 2022-04- 13 7:45 | 2022/4/ 12 | 2022-04-30 10:28 | 17 |
| BA.2 | 281048 | female | 27 | Mild | Omicron (BA.2) | 2022-04- 13 11:20 | 2022/4/ 12 | 2022-04-27 17:04 | 14 |
| BA.2 | 281063 | male | 47 | Asymptomatic | Omicron (BA.2) | 2022-04- 13 14:19 | 2022/4/ 13 | 2022-04-24 11:15 | 11 |
| BA.2 | 281064 | male | 59 | Mild | Omicron (BA.2) | 2022-04- 13 14:20 | 2022/4/6 | 2022-05-01 13:15 | 18 |
| BA.2 | 281079 | male | 30 | Asymptomatic | Omicron (BA.2) | 2022-04- 13 16:03 | 2022/4/ 13 | 2022-04-23 9:47 | 10 |
| BA.2 | 281085 | male | 26 | Mild | Omicron (BA.2) | 2022-04- 13 16:10 | 2022/4/9 | 2022-04-27 19:25 | 14 |
| BA.2 | 281095 | male | 28 | Asymptomatic | Omicron (BA.2) | 2022-04- 13 17:05 | 2022/4/ 13 | 2022-04-29 12:30 | 16 |
| BA.2 | 281096 | male | 36 | Asymptomatic | Omicron (BA.2) | 2022-04- 13 17:06 | 2022/4/ 13 | 2022-04-29 12:03 | 16 |
| BA.2 | 281100 | female | 57 | Mild | Omicron (BA.2) | 2022-04- 13 20:26 | 2022/4/ 11 | 2022-05-05 10:14 | 22 |
| BA.2 | 281101 | male | 38 | Mild | Omicron (BA.2) | 2022-04- 13 20:15 | 2022/4/ 12 | 2022-04-27 16:18 | 14 |
| BA.2 | 281103 | male | 33 | Mild | Omicron (BA.2) | 2022-04- 13 19:59 | 2022/4/ 12 | 2022-04-24 10:56 | 11 |
| BA.2 | 281104 | male | 33 | Asymptomatic | Omicron (BA.2) | 2022-04- 13 23:00 | 2022/4/ 13 | 2022-04-27 10:37 | 13 |
| BA.2 | 281105 | male | 31 | Asymptomatic | Omicron (BA.2) | 2022-04- 13 23:16 | 2022/4/ 13 | 2022-05-01 9:39 | 17 |
| BA.2 | 281106 | male | 33 | Mild | Omicron (BA.2) | 2022-04- 13 20:00 | 2022/4/ 12 | 2022-04-24 12:30 | 11 |
| BA.2 | 281108 | male | 25 | Asymptomatic | Omicron (BA.2) | 2022-04- 13 20:29 | 2022/4/ 13 | 2022-04-25 9:20 | 12 |
| BA.2 | 281109 | male | 38 | Asymptomatic | Omicron (BA.2) | 2022-04- 13 20:29 | 2022/4/ 13 | 2022-04-28 10:11 | 15 |
| BA.2 | 281110 | male | 29 | Moderate | Omicron (BA.2) | 2022-04- 13 20:28 | 2022/4/ 13 | 2022-04-26 12:20 | 13 |
| BA.2 | 281111 | male | 32 | Asymptomatic | Omicron (BA.2) | 2022-04- 13 21:03 | 2022/4/ 13 | 2022-05-04 10:41 | 21 |

| BA.2 | 281113 | male | 34 | Asymptomatic | Omicron (BA.2) | 2022-04- 13 21:09 | 2022/4/ 13 | 2022-04-25 8:22 | 11 |
| --- | --- | --- | --- | --- | --- | --- | --- | --- | --- |
| BA.2 | 281114 | male | 47 | Asymptomatic | Omicron (BA.2) | 2022-04- 13 23:01 | 2022/4/ 13 | 2022-04-27 15:38 | 14 |
| BA.2 | 281117 | male | 56 | Asymptomatic | Omicron (BA.2) | 2022-04- 13 22:41 | 2022/4/ 13 | 2022-05-04 9:00 | 20 |
| BA.2 | 281119 | male | 37 | Asymptomatic | Omicron (BA.2) | 2022-04- 13 23:34 | 2022/4/ 12 | 2022-04-29 11:59 | 16 |
| BA.2 | 281128 | male | 23 | Mild | Omicron (BA.2) | 2022-04- 14 6:17 | 2022/4/ 13 | 2022-04-23 9:49 | 9 |
| BA.2 | 281129 | male | 47 | Asymptomatic | Omicron (BA.2) | 2022-04- 14 6:18 | 2022/4/ 13 | 2022-04-30 10:19 | 16 |
| BA.2 | 281142 | male | 42 | Asymptomatic | Omicron (BA.2) | 2022-04- 14 11:32 | 2022/4/ 14 | 2022-04-27 13:48 | 13 |
| BA.2 | 281144 | male | 26 | Asymptomatic | Omicron (BA.2) | 2022-04- 14 11:33 | 2022/4/ 14 | 2022-04-28 10:08 | 14 |
| BA.2 | 281146 | male | 32 | Asymptomatic | Omicron (BA.2) | 2022-04- 14 11:33 | 2022/4/ 13 | 2022-04-27 11:11 | 13 |
| BA.2 | 281186 | male | 22 | Asymptomatic | Omicron (BA.2) | 2022-04- 14 23:02 | 2022/4/ 14 | 2022-04-27 14:25 | 13 |
| BA.2 | 281187 | male | 24 | Asymptomatic | Omicron (BA.2) | 2022-04- 14 23:01 | 2022/4/ 14 | 2022-04-22 11:14 | 8 |
| BA.2 | 281188 | male | 27 | Moderate | Omicron (BA.2) | 2022-04- 14 23:50 | 2022/4/ 12 | 2022-04-23 9:33 | 8 |
| BA.2 | 281189 | male | 22 | Asymptomatic | Omicron (BA.2) | 2022-04- 15 1:04 | 2022/4/ 14 | 2022-04-30 11:12 | 15 |
| BA.2 | 281205 | male | 43 | Moderate | Omicron (BA.2) | 2022-04- 15 11:07 | 2022/4/ 14 | 2022-04-28 13:57 | 13 |
| BA.2 | 281249 | male | 49 | Mild | Omicron (BA.2) | 2022-04- 15 20:34 | 2022/4/ 14 | 2022-04-29 11:22 | 14 |
| BA.2 | 281250 | male | 34 | Asymptomatic | Omicron (BA.2) | 2022-04- 15 20:34 | 2022/4/ 15 | 2022-04-24 10:47 | 9 |
| BA.2 | 281251 | male | 35 | Mild | Omicron (BA.2) | 2022-04- 15 20:33 | 2022/4/ 13 | 2022-04-24 10:48 | 9 |
| BA.2 | 281252 | male | 32 | Asymptomatic | Omicron (BA.2) | 2022-04- 15 23:13 | 2022/4/ 15 | 2022-05-03 10:40 | 17 |
| BA.2 | 281257 | male | 35 | Moderate | Omicron (BA.2) | 2022-04- 16 3:27 | 2022/4/ 15 | 2022-05-04 11:29 | 18 |
| BA.2 | 281258 | male | 27 | Mild | Omicron (BA.2) | 2022-04- 16 3:28 | 2022/4/ 15 | 2022-05-01 12:21 | 15 |
| BA.2 | 281280 | male | 49 | Mild | Omicron (BA.2) | 2022-04- 16 12:44 | 2022/4/ 15 | 2022-05-01 12:21 | 15 |
| BA.2 | 281299 | male | 38 | Mild | Omicron (BA.2) | 2022-04- 16 21:29 | 2022/4/ 16 | 2022-05-01 10:26 | 15 |
| BA.2 | 281300 | male | 21 | Mild | Omicron (BA.2) | 2022-04- 16 21:30 | 2022/4/ 16 | 2022-05-02 9:05 | 15 |
| BA.2 | 281312 | male | 38 | Asymptomatic | Omicron (BA.2) | 2022-04- 17 4:35 | 2022/4/ 16 | 2022-04-30 13:14 | 13 |
| BA.2 | 281313 | male | 32 | Asymptomatic | Omicron (BA.2) | 2022-04- 17 4:35 | 2022/4/ 16 | 2022-05- 11 10:05 | 24 |
| BA.2 | 281314 | male | 40 | Mild | Omicron (BA.2) | 2022-04- 17 4:35 | 2022/4/ 16 | 2022-04-27 11:11 | 10 |
| BA.2 | 281371 | male | 26 | Mild | Omicron (BA.2) | 2022-04- 18 0:32 | 2022/4/ 17 | 2022-04-30 11:54 | 12 |
| BA.2 | 281469 | male | 33 | Moderate | Omicron (BA.2) | 2022-04- 18 21:48 | 2022/4/ 17 | 2022-05-01 8:43 | 12 |
| BA.2 | 281737 | female | 25 | Mild | Omicron (BA.2) | 2022-04-21 23:24 | 2022/4/21 | 2022-05-07 11:07 | 15 |
| BA.2 | 281772 | female | 35 | Mild | Omicron (BA.2) | 2022-04-22 12:00 | 2022/4/20 | 2022-05- 11 10:00 | 19 |
| BA.2 | 282057 | male | 26 | Mild | Omicron (BA.2) | 2022-04-25 17:03 | 2022/4/24 | 2022-05-07 13:21 | 12 |

| **Variants** | **ID** | **Vaccination** | **vaccine** **doses** | **Type** **of** **vaccine** **(1)** | **Type** **of** **vaccine** **(2)** | **Type** **of** **vaccine** **(3)** | **Days** **between** **the**  **last** **dose** **and**  **the** **onset** **of** **illness** |
| --- | --- | --- | --- | --- | --- | --- | --- |
| BA.2 | 158689 | Boosted | 3 | Inactivated vaccines | Inactivated vaccines | Inactivated vaccines | 170 |
| BA.2 | 274953 | Fully vaccinated | 2 | Inactivated vaccines | Inactivated vaccines |  | 253 |
| BA.2 | 275062 | Fully vaccinated | 2 | Inactivated vaccines | Inactivated vaccines |  |  |
| BA.2 | 275212 | Boosted | 3 | Inactivated vaccines | Inactivated vaccines | mRNA | 53 |
| BA.2 | 275481 | Fully vaccinated | 2 | Inactivated vaccines | Inactivated vaccines |  |  |
| BA.2 | 275576 | Unvaccinated | 1 | Unknown |  |  |  |
| BA.2 | 275582 | Fully vaccinated | 2 | Inactivated vaccines | Inactivated vaccines |  | 225 |
| BA.2 | 275583 | Fully vaccinated | 2 | Inactivated vaccines | Inactivated vaccines |  |  |
| BA.2 | 275584 | Boosted | 3 | Inactivated vaccines | Inactivated vaccines | Inactivated vaccines | 10 |
| BA.2 | 275590 | Boosted | 3 | Inactivated vaccines | Inactivated vaccines | Inactivated vaccines | 42 |
| BA.2 | 275595 | Boosted | 3 | Inactivated vaccines | Inactivated vaccines | Inactivated vaccines |  |
| BA.2 | 275596 | Unvaccinated | 0 |  |  |  |  |
| BA.2 | 275600 | Boosted | 3 | Inactivated vaccines | Inactivated vaccines | Inactivated vaccines |  |
| BA.2 | 275603 | Unvaccinated | 0 | #N/A | #N/A | #N/A |  |
| BA.2 | 275605 | Boosted | 3 | Inactivated vaccines | Inactivated vaccines | Inactivated vaccines | 23 |
| BA.2 | 275612 | Boosted | 3 | Inactivated vaccines | Inactivated vaccines | Inactivated vaccines | 89 |
| BA.2 | 275619 | Boosted | 3 | Inactivated vaccines | Inactivated vaccines | Inactivated vaccines | 127 |
| BA.2 | 275622 | Boosted | 3 | Inactivated vaccines | Inactivated vaccines | Inactivated vaccines | 128 |
| BA.2 | 275623 | Boosted | 3 | Inactivated vaccines | Inactivated vaccines | Inactivated vaccines | 125 |
| BA.2 | 275627 | Fully vaccinated | 2 | Inactivated vaccines | Inactivated vaccines |  |  |
| BA.2 | 275631 | Fully vaccinated | 2 | Inactivated vaccines | Inactivated vaccines |  | 225 |
| BA.2 | 275643 | Fully vaccinated | 2 | Inactivated vaccines | Inactivated vaccines |  | 138 |
| BA.2 | 275650 | Boosted | 3 | Inactivated vaccines | Inactivated vaccines | Inactivated vaccines |  |
| BA.2 | 275660 | Fully vaccinated | 2 | Inactivated vaccines | Inactivated vaccines |  | 224 |
| BA.2 | 275662 | Unvaccinated | 1 | Inactivated vaccines |  |  | 316 |
| BA.2 | 275674 | Boosted | 3 | Inactivated vaccines | Inactivated vaccines | Inactivated vaccines | 37 |
| BA.2 | 275676 | Fully vaccinated | 2 | Inactivated vaccines | Inactivated vaccines |  |  |
| BA.2 | 275677 | Boosted | 3 | Inactivated vaccines | Inactivated vaccines | Inactivated vaccines | 51 |
| BA.2 | 275678 | Fully vaccinated | 2 | Inactivated vaccines | Inactivated vaccines |  | 18 |
| BA.2 | 275691 | Boosted | 3 | mRNA | mRNA | mRNA | 71 |
| BA.2 | 275721 | Unvaccinated | 0 |  |  |  |  |
| BA.2 | 275729 | Fully vaccinated | 2 | Inactivated vaccines | Inactivated vaccines |  | 223 |
| BA.2 | 275743 | Fully vaccinated | 2 | Inactivated vaccines | Inactivated vaccines |  |  |

| BA.2 | 275767 | Fully vaccinated | 2 | Inactivated vaccines | Inactivated vaccines |  | 122 |
| --- | --- | --- | --- | --- | --- | --- | --- |
| BA.2 | 275799 | Boosted | 3 | Inactivated vaccines | Inactivated vaccines | Inactivated vaccines | 33 |
| BA.2 | 275809 | Fully vaccinated | 2 | Inactivated vaccines | Inactivated vaccines |  | 238 |
| BA.2 | 275887 | Unvaccinated | 0 |  |  |  |  |
| BA.2 | 275910 | Fully vaccinated | 2 | mRNA | mRNA |  | 164 |
| BA.2 | 275921 | Boosted | 3 | Inactivated vaccines | Inactivated vaccines | Inactivated vaccines | 0 |
| BA.2 | 275922 | Boosted | 3 | Inactivated vaccines | Inactivated vaccines | Inactivated vaccines | 14 |
| BA.2 | 275923 | Fully vaccinated | 2 | Inactivated vaccines | Inactivated vaccines |  | 143 |
| BA.2 | 275944 | Fully vaccinated | 2 | Inactivated vaccines | Inactivated vaccines |  | 18 |
| BA.2 | 275946 | Boosted | 3 | Inactivated vaccines | Inactivated vaccines | Inactivated vaccines | 60 |
| BA.2 | 276007 | Boosted | 3 | Inactivated vaccines | Inactivated vaccines | Inactivated vaccines | 45 |
| BA.2 | 276082 | Unvaccinated | 0 |  |  |  |  |
| BA.2 | 276093 | Boosted | 3 | Inactivated vaccines | Inactivated vaccines | Inactivated vaccines | 49 |
| BA.2 | 276094 | Fully vaccinated | 2 | Inactivated vaccines | Inactivated vaccines |  | 68 |
| BA.2 | 276103 | Boosted | 3 | Inactivated vaccines | Inactivated vaccines | Inactivated vaccines | 64 |
| BA.2 | 276145 | Fully vaccinated | 2 | Inactivated vaccines | Inactivated vaccines |  | 82 |
| BA.2 | 276161 | Unvaccinated | 0 |  |  |  |  |
| BA.2 | 276162 | Fully vaccinated | 2 | Inactivated vaccines | Inactivated vaccines |  | 149 |
| BA.2 | 276163 | Fully vaccinated | 2 | Unknown | Unknown |  | 132 |
| BA.2 | 276164 | Unvaccinated | 1 | Unknown |  |  | 11 |
| BA.2 | 276170 | Unvaccinated | 1 | Inactivated vaccines |  |  | 282 |
| BA.2 | 276171 | Unvaccinated | 0 |  |  |  |  |
| BA.2 | 276172 | Fully vaccinated | 2 | Inactivated vaccines | Inactivated vaccines |  | 233 |
| BA.2 | 276179 | Boosted | 3 | Inactivated vaccines | Inactivated vaccines | Inactivated vaccines | 16 |
| BA.2 | 276186 | Fully vaccinated | 2 | Inactivated vaccines | Inactivated vaccines |  | 339 |
| BA.2 | 276189 | Fully vaccinated | 2 | Inactivated vaccines | Inactivated vaccines |  | 191 |
| BA.2 | 276192 | Boosted | 3 | Inactivated vaccines | Inactivated vaccines | Inactivated vaccines | 16 |
| BA.2 | 276232 | Unvaccinated | 0 |  |  |  |  |
| BA.2 | 276233 | Unvaccinated | 0 |  |  |  |  |
| BA.2 | 276235 | Fully vaccinated | 2 | Inactivated vaccines | Inactivated vaccines |  | 211 |
| BA.2 | 276241 | Fully vaccinated | 2 | Inactivated vaccines | Inactivated vaccines |  | 232 |
| BA.2 | 276243 | Boosted | 3 | Inactivated vaccines | Inactivated vaccines | Inactivated vaccines | 121 |
| BA.2 | 276247 | Fully vaccinated | 2 | Inactivated vaccines | Inactivated vaccines |  | 205 |
| BA.2 | 276248 | Fully vaccinated | 2 | Inactivated vaccines | Inactivated vaccines |  | 39 |
| BA.2 | 276249 | Fully vaccinated | 2 | Inactivated vaccines | Inactivated vaccines |  | 172 |
| BA.2 | 276250 | Boosted | 3 | Inactivated vaccines | Inactivated vaccines | Inactivated vaccines | 65 |

| BA.2 | 276251 | Fully vaccinated | 2 | Inactivated vaccines | Inactivated vaccines |  | 71 |
| --- | --- | --- | --- | --- | --- | --- | --- |
| BA.2 | 276252 | Unvaccinated | 0 |  |  |  |  |
| BA.2 | 276253 | Boosted | 3 | Inactivated vaccines | Inactivated vaccines | Inactivated vaccines | 4 |
| BA.2 | 276312 | Fully vaccinated | 2 | Inactivated vaccines | Inactivated vaccines |  | 224 |
| BA.2 | 276313 | Fully vaccinated | 2 | Inactivated vaccines | Inactivated vaccines |  | 245 |
| BA.2 | 276314 | Unvaccinated | 0 | #N/A | #N/A | #N/A |  |
| BA.2 | 276316 | Fully vaccinated | 2 | Inactivated vaccines | Inactivated vaccines |  | 25 |
| BA.2 | 276319 | Boosted | 3 | Inactivated vaccines | Inactivated vaccines | Inactivated vaccines | 60 |
| BA.2 | 276325 | Fully vaccinated | 2 | Inactivated vaccines | Inactivated vaccines |  | 321 |
| BA.2 | 276326 | Fully vaccinated | 2 | Inactivated vaccines | Inactivated vaccines |  | 94 |
| BA.2 | 276327 | Boosted | 3 | Inactivated vaccines | Inactivated vaccines | Inactivated vaccines | 70 |
| BA.2 | 276328 | Boosted | 3 | Inactivated vaccines | Inactivated vaccines | Inactivated vaccines | 4 |
| BA.2 | 276349 | Fully vaccinated | 2 | mRNA | mRNA |  | 179 |
| BA.2 | 276369 | Fully vaccinated | 2 | mRNA | mRNA |  | 268 |
| BA.2 | 276373 | Unvaccinated | 0 |  |  |  |  |
| BA.2 | 276405 | Unvaccinated | 0 |  |  |  |  |
| BA.2 | 276409 | Fully vaccinated | 2 | Inactivated vaccines | Inactivated vaccines |  | 42 |
| BA.2 | 276410 | Fully vaccinated | 2 | Inactivated vaccines | Inactivated vaccines |  | 136 |
| BA.2 | 276442 | Unvaccinated | 0 |  |  |  |  |
| BA.2 | 276444 | Boosted | 3 | Inactivated vaccines | Inactivated vaccines | Inactivated vaccines | 32 |
| BA.2 | 276457 | Fully vaccinated | 2 | Inactivated vaccines | Inactivated vaccines |  | 74 |
| BA.2 | 276459 | Unvaccinated | 0 |  |  |  |  |
| BA.2 | 276460 | Fully vaccinated | 2 | mRNA | mRNA |  | 168 |
| BA.2 | 276461 | Fully vaccinated | 2 | Inactivated vaccines | Inactivated vaccines |  | 244 |
| BA.2 | 276471 | Boosted | 3 | Inactivated vaccines | Inactivated vaccines | Inactivated vaccines | 50 |
| BA.2 | 276472 | Boosted | 3 | Inactivated vaccines | Inactivated vaccines | Inactivated vaccines | 155 |
| BA.2 | 276498 | Boosted | 3 | mRNA | mRNA | mRNA | 85 |
| BA.2 | 276503 | Fully vaccinated | 2 | Inactivated vaccines | Inactivated vaccines |  | 202 |
| BA.2 | 276509 | Boosted | 3 | Inactivated vaccines | Inactivated vaccines | Inactivated vaccines | 58 |
| BA.2 | 276530 | Fully vaccinated | 2 | Inactivated vaccines | Inactivated vaccines |  | 198 |
| BA.2 | 276531 | Fully vaccinated | 2 | Inactivated vaccines | Inactivated vaccines |  | 127 |
| BA.2 | 276542 | Fully vaccinated | 2 | Inactivated vaccines | Inactivated vaccines |  | 182 |
| BA.2 | 276553 | Boosted | 3 | Inactivated vaccines | Inactivated vaccines | Inactivated vaccines | 75 |
| BA.2 | 276570 | Unvaccinated | 0 |  |  |  |  |
| BA.2 | 276571 | Fully vaccinated | 2 | Inactivated vaccines | Inactivated vaccines |  | 206 |
| BA.2 | 276577 | Unvaccinated | 0 |  |  |  |  |

| BA.2 | 276578 | Unvaccinated | 0 |  |  |  |  |
| --- | --- | --- | --- | --- | --- | --- | --- |
| BA.2 | 276587 | Fully vaccinated | 2 | Inactivated vaccines | Inactivated vaccines |  | 226 |
| BA.2 | 276590 | Boosted | 3 | Inactivated vaccines | Inactivated vaccines | mRNA | 68 |
| BA.2 | 276599 | Fully vaccinated | 2 | Inactivated vaccines | Inactivated vaccines |  | 236 |
| BA.2 | 276600 | Boosted | 3 | Inactivated vaccines | Inactivated vaccines | Inactivated vaccines | 51 |
| BA.2 | 276601 | Fully vaccinated | 2 | Inactivated vaccines | Inactivated vaccines |  | 209 |
| BA.2 | 276617 | Fully vaccinated | 2 | Inactivated vaccines | Inactivated vaccines |  | 196 |
| BA.2 | 276618 | Fully vaccinated | 2 | Inactivated vaccines | Inactivated vaccines |  | 209 |
| BA.2 | 276632 | Fully vaccinated | 2 | Inactivated vaccines | Inactivated vaccines |  | 189 |
| BA.2 | 276634 | Boosted | 3 | Inactivated vaccines | Inactivated vaccines | Inactivated vaccines | 9 |
| BA.2 | 276640 | Boosted | 3 | Inactivated vaccines | Inactivated vaccines | Inactivated vaccines |  |
| BA.2 | 276658 | Boosted | 3 | Inactivated vaccines | Inactivated vaccines | Inactivated vaccines | 41 |
| BA.2 | 276662 | Boosted | 3 | Inactivated vaccines | Inactivated vaccines | Inactivated vaccines | 158 |
| BA.2 | 276671 | Boosted | 3 | Inactivated vaccines | Inactivated vaccines | Inactivated vaccines | 28 |
| BA.2 | 276679 | Fully vaccinated | 2 | mRNA | mRNA |  | 242 |
| BA.2 | 276717 | Fully vaccinated | 2 | Inactivated vaccines | Inactivated vaccines |  | 202 |
| BA.2 | 276747 | Fully vaccinated | 2 | Inactivated vaccines | Inactivated vaccines |  | 69 |
| BA.2 | 276748 | Boosted | 3 | Inactivated vaccines | Inactivated vaccines | mRNA | 21 |
| BA.2 | 276756 | Fully vaccinated | 2 | Inactivated vaccines | Inactivated vaccines |  | 269 |
| BA.2 | 276770 | Boosted | 3 | Inactivated vaccines | Inactivated vaccines | Inactivated vaccines | 39 |
| BA.2 | 276772 | Unvaccinated | 1 | mRNA |  |  | 169 |
| BA.2 | 276778 | Fully vaccinated | 2 | Inactivated vaccines | Inactivated vaccines |  | 198 |
| BA.2 | 276779 | Fully vaccinated | 2 | Inactivated vaccines | Inactivated vaccines |  | 201 |
| BA.2 | 276780 | Fully vaccinated | 2 | mRNA | mRNA |  | 209 |
| BA.2 | 276783 | Fully vaccinated | 2 | mRNA | mRNA |  | 293 |
| BA.2 | 276788 | Fully vaccinated | 2 | Inactivated vaccines | Inactivated vaccines |  | 173 |
| BA.2 | 276789 | Unvaccinated | 0 |  |  |  |  |
| BA.2 | 276790 | Boosted | 3 | Inactivated vaccines | Inactivated vaccines | mRNA | 84 |
| BA.2 | 276793 | Fully vaccinated | 2 | mRNA | mRNA |  | 272 |
| BA.2 | 276801 | Fully vaccinated | 2 | Inactivated vaccines | Inactivated vaccines |  | 188 |
| BA.2 | 276802 | Fully vaccinated | 2 | Unknown | mRNA |  | 195 |
| BA.2 | 276803 | Fully vaccinated | 2 | Inactivated vaccines | Inactivated vaccines |  | 359 |
| BA.2 | 276818 | Fully vaccinated | 2 | mRNA | mRNA |  | 205 |
| BA.2 | 276819 | Boosted | 3 | Inactivated vaccines | Inactivated vaccines | mRNA | 73 |
| BA.2 | 276821 | Fully vaccinated | 2 | Inactivated vaccines | Inactivated vaccines |  | 210 |
| BA.2 | 276826 | Unvaccinated | 0 |  |  |  |  |

| BA.2 | 276849 | Fully vaccinated | 2 | Inactivated vaccines | Inactivated vaccines |  | 250 |
| --- | --- | --- | --- | --- | --- | --- | --- |
| BA.2 | 276853 | Boosted | 3 | Inactivated vaccines | Inactivated vaccines | mRNA | 11 |
| BA.2 | 276855 | Fully vaccinated | 2 | Inactivated vaccines | Inactivated vaccines |  | 239 |
| BA.2 | 276857 | Unvaccinated | 0 |  |  |  |  |
| BA.2 | 276858 | Boosted | 3 | Inactivated vaccines | Inactivated vaccines | Inactivated vaccines | 26 |
| BA.2 | 276859 | Unvaccinated | 0 |  |  |  |  |
| BA.2 | 276860 | Fully vaccinated | 2 | Inactivated vaccines | Inactivated vaccines |  | 213 |
| BA.2 | 276861 | Boosted | 3 | mRNA | mRNA | mRNA | 97 |
| BA.2 | 276862 | Boosted | 3 | mRNA | mRNA | mRNA | 78 |
| BA.2 | 276864 | Unvaccinated | 0 |  |  |  |  |
| BA.2 | 276867 | Boosted | 3 | Inactivated vaccines | Inactivated vaccines | Inactivated vaccines | 141 |
| BA.2 | 276868 | Boosted | 3 | Inactivated vaccines | Inactivated vaccines | Inactivated vaccines | 142 |
| BA.2 | 276869 | Fully vaccinated | 2 | Inactivated vaccines | Inactivated vaccines |  | 228 |
| BA.2 | 276870 | Boosted | 3 | Inactivated vaccines | Inactivated vaccines | Inactivated vaccines | 141 |
| BA.2 | 276871 | Boosted | 3 | Inactivated vaccines | Inactivated vaccines | Inactivated vaccines | 142 |
| BA.2 | 276872 | Boosted | 3 | Inactivated vaccines | Inactivated vaccines | Inactivated vaccines | 141 |
| BA.2 | 276873 | Fully vaccinated | 2 | Inactivated vaccines | Inactivated vaccines |  | 254 |
| BA.2 | 276874 | Boosted | 3 | Unknown | Unknown | Unknown | 109 |
| BA.2 | 276876 | Fully vaccinated | 2 | Inactivated vaccines | Inactivated vaccines |  | 258 |
| BA.2 | 276879 | Boosted | 3 | Unknown | Unknown | Unknown |  |
| BA.2 | 276880 | Fully vaccinated | 2 | Inactivated vaccines | Inactivated vaccines |  | 277 |
| BA.2 | 276882 | Boosted | 3 | Inactivated vaccines | Inactivated vaccines | Inactivated vaccines | 146 |
| BA.2 | 276914 | Fully vaccinated | 2 | Inactivated vaccines | Inactivated vaccines |  | 179 |
| BA.2 | 276915 | Fully vaccinated | 2 | Inactivated vaccines | Inactivated vaccines |  | 175 |
| BA.2 | 276931 | Boosted | 3 | Inactivated vaccines | Inactivated vaccines | mRNA | 4 |
| BA.2 | 276932 | Unvaccinated | 0 |  |  |  |  |
| BA.2 | 276945 | Unvaccinated | 0 |  |  |  |  |
| BA.2 | 276950 | Boosted | 3 | Inactivated vaccines | Inactivated vaccines | Inactivated vaccines | 63 |
| BA.2 | 276952 | Unvaccinated | 0 |  |  |  |  |
| BA.2 | 276953 | Fully vaccinated | 2 | Inactivated vaccines | Inactivated vaccines |  | 241 |
| BA.2 | 276957 | Boosted | 3 | Inactivated vaccines | Inactivated vaccines | mRNA | 15 |
| BA.2 | 276958 | Boosted | 3 | Unknown | Unknown | Unknown | 264 |
| BA.2 | 276959 | Boosted | 3 | Inactivated vaccines | Inactivated vaccines | Unknown | 39 |
| BA.2 | 276966 | Fully vaccinated | 2 | mRNA | mRNA |  | 162 |
| BA.2 | 276972 | Boosted | 3 | Inactivated vaccines | Inactivated vaccines | Inactivated vaccines | 11 |
| BA.2 | 276999 | Boosted | 3 | Inactivated vaccines | Inactivated vaccines | Inactivated vaccines | 100 |

| BA.2 | 277007 | Fully vaccinated | 2 | Inactivated vaccines | Inactivated vaccines |  | 226 |
| --- | --- | --- | --- | --- | --- | --- | --- |
| BA.2 | 277024 | Unvaccinated | 1 | mRNA |  |  | 70 |
| BA.2 | 277025 | Fully vaccinated | 2 | mRNA | mRNA |  | 220 |
| BA.2 | 277034 | Unvaccinated | 0 |  |  |  |  |
| BA.2 | 277035 | Fully vaccinated | 2 | Inactivated vaccines | Inactivated vaccines |  | 202 |
| BA.2 | 277037 | Unvaccinated | 0 |  |  |  |  |
| BA.2 | 277038 | Unvaccinated | 0 |  |  |  |  |
| BA.2 | 277048 | Fully vaccinated | 2 | Inactivated vaccines | Inactivated vaccines |  | 233 |
| BA.2 | 277052 | Boosted | 3 | Inactivated vaccines | Inactivated vaccines | mRNA | 10 |
| BA.2 | 277053 | Fully vaccinated | 2 | Inactivated vaccines | Inactivated vaccines |  | 182 |
| BA.2 | 277054 | Unvaccinated | 0 |  |  |  |  |
| BA.2 | 277064 | Fully vaccinated | 2 | Inactivated vaccines | Inactivated vaccines |  | 189 |
| BA.2 | 277065 | Unvaccinated | 0 |  |  |  |  |
| BA.2 | 277092 | Fully vaccinated | 2 | Inactivated vaccines | Inactivated vaccines |  | 211 |
| BA.2 | 277102 | Fully vaccinated | 2 | mRNA | mRNA |  | 207 |
| BA.2 | 277103 | Fully vaccinated | 2 | Inactivated vaccines | Inactivated vaccines |  | 211 |
| BA.2 | 277104 | Fully vaccinated | 2 | mRNA | mRNA |  | 128 |
| BA.2 | 277107 | Unvaccinated | 0 |  |  |  |  |
| BA.2 | 277108 | Unvaccinated | 0 |  |  |  |  |
| BA.2 | 277113 | Fully vaccinated | 2 | Inactivated vaccines | Inactivated vaccines |  | 177 |
| BA.2 | 277118 | Unvaccinated | 0 |  |  |  |  |
| BA.2 | 277119 | Fully vaccinated | 2 | Inactivated vaccines | Inactivated vaccines |  | 240 |
| BA.2 | 277120 | Unvaccinated | 0 |  |  |  |  |
| BA.2 | 277138 | Fully vaccinated | 2 | Inactivated vaccines | Inactivated vaccines |  | 183 |
| BA.2 | 277152 | Unvaccinated | 0 |  |  |  |  |
| BA.2 | 277153 | Fully vaccinated | 2 | Inactivated vaccines | Inactivated vaccines |  | 255 |
| BA.2 | 277161 | Fully vaccinated | 2 | mRNA | mRNA |  | 45 |
| BA.2 | 277165 | Unvaccinated | 0 |  |  |  |  |
| BA.2 | 277167 | Unvaccinated | 0 |  |  |  |  |
| BA.2 | 277168 | Fully vaccinated | 2 | Inactivated vaccines | Inactivated vaccines |  | 237 |
| BA.2 | 277169 | Unvaccinated | 0 |  |  |  |  |
| BA.2 | 277170 | Fully vaccinated | 2 | Inactivated vaccines | Inactivated vaccines |  | 51 |
| BA.2 | 277171 | Fully vaccinated | 2 | Inactivated vaccines | Unknown |  | 241 |
| BA.2 | 277172 | Unvaccinated | 0 |  |  |  |  |
| BA.2 | 277175 | Fully vaccinated | 2 | Inactivated vaccines | Inactivated vaccines |  | 227 |
| BA.2 | 277176 | Fully vaccinated | 2 | Inactivated vaccines | Inactivated vaccines |  | 307 |

| BA.2 | 277177 | Unvaccinated | 1 | Inactivated vaccines |  |  | 206 |
| --- | --- | --- | --- | --- | --- | --- | --- |
| BA.2 | 277179 | Fully vaccinated | 2 | Inactivated vaccines | Inactivated vaccines |  | 11 |
| BA.2 | 277181 | Boosted | 3 | Inactivated vaccines | Inactivated vaccines | Inactivated vaccines | 89 |
| BA.2 | 277182 | Boosted | 3 | Inactivated vaccines | Inactivated vaccines | Inactivated vaccines | 72 |
| BA.2 | 277184 | Unvaccinated | 0 |  |  |  |  |
| BA.2 | 277185 | Boosted | 3 | Inactivated vaccines | Inactivated vaccines | mRNA | 79 |
| BA.2 | 277187 | Boosted | 3 | mRNA | mRNA | mRNA | 12 |
| BA.2 | 277188 | Unvaccinated | 1 | Inactivated vaccines |  |  |  |
| BA.2 | 277193 | Boosted | 3 | Inactivated vaccines | Inactivated vaccines | mRNA | 11 |
| BA.2 | 277194 | Boosted | 3 | Inactivated vaccines | Inactivated vaccines | Inactivated vaccines | 50 |
| BA.2 | 277195 | Fully vaccinated | 2 | Inactivated vaccines | Inactivated vaccines |  | 302 |
| BA.2 | 277200 | Fully vaccinated | 2 | Inactivated vaccines | Inactivated vaccines |  | 248 |
| BA.2 | 277201 | Fully vaccinated | 2 | Inactivated vaccines | Inactivated vaccines |  | 195 |
| BA.2 | 277202 | Fully vaccinated | 2 | Inactivated vaccines | Inactivated vaccines |  | 180 |
| BA.2 | 277204 | Unvaccinated | 0 |  |  |  |  |
| BA.2 | 277205 | Boosted | 3 | Inactivated vaccines | Inactivated vaccines | Inactivated vaccines | 30 |
| BA.2 | 277209 | Fully vaccinated | 2 | Inactivated vaccines | Inactivated vaccines |  | 296 |
| BA.2 | 277210 | Unvaccinated | 0 |  |  |  |  |
| BA.2 | 277212 | Unvaccinated | 1 | Inactivated vaccines |  |  | 133 |
| BA.2 | 277213 | Boosted | 3 | Inactivated vaccines | Inactivated vaccines | Inactivated vaccines | 52 |
| BA.2 | 277218 | Unvaccinated | 0 |  |  |  |  |
| BA.2 | 277219 | Boosted | 3 | Inactivated vaccines | Inactivated vaccines | mRNA | 62 |
| BA.2 | 277220 | Unvaccinated | 0 |  |  |  |  |
| BA.2 | 277230 | Boosted | 3 | Inactivated vaccines | Inactivated vaccines | Inactivated vaccines | 140 |
| BA.2 | 277231 | Boosted | 3 | Inactivated vaccines | Inactivated vaccines | Inactivated vaccines | 96 |
| BA.2 | 277233 | Fully vaccinated | 2 | Inactivated vaccines | Inactivated vaccines |  | 38 |
| BA.2 | 277248 | Unvaccinated | 0 |  |  |  |  |
| BA.2 | 277250 | Unvaccinated | 1 | Inactivated vaccines |  |  | 10 |
| BA.2 | 277252 | Fully vaccinated | 2 | Inactivated vaccines | Inactivated vaccines |  | 386 |
| BA.2 | 277281 | Boosted | 3 | Inactivated vaccines | Inactivated vaccines | Inactivated vaccines | 9 |
| BA.2 | 277282 | Unvaccinated | 0 |  |  |  |  |
| BA.2 | 277283 | Boosted | 3 | Inactivated vaccines | Inactivated vaccines | Inactivated vaccines | 60 |
| BA.2 | 277284 | Unvaccinated | 0 |  |  |  |  |
| BA.2 | 277287 | Fully vaccinated | 2 | Inactivated vaccines | Inactivated vaccines |  | 25 |
| BA.2 | 277288 | Unvaccinated | 0 |  |  |  |  |
| BA.2 | 277289 | Boosted | 3 | Inactivated vaccines | Inactivated vaccines | Inactivated vaccines | 36 |

| BA.2 | 277290 | Boosted | 3 | Inactivated vaccines | Inactivated vaccines | Inactivated vaccines | 101 |
| --- | --- | --- | --- | --- | --- | --- | --- |
| BA.2 | 277291 | Boosted | 3 | Inactivated vaccines | Inactivated vaccines | Inactivated vaccines | 78 |
| BA.2 | 277292 | Boosted | 3 | Inactivated vaccines | Inactivated vaccines | Inactivated vaccines | 95 |
| BA.2 | 277293 | Boosted | 3 | Inactivated vaccines | Inactivated vaccines | Inactivated vaccines | 95 |
| BA.2 | 277296 | Fully vaccinated | 2 | Inactivated vaccines | Inactivated vaccines |  | 61 |
| BA.2 | 277297 | Unvaccinated | 0 |  |  |  |  |
| BA.2 | 277298 | Unvaccinated | 0 |  |  |  |  |
| BA.2 | 277299 | Fully vaccinated | 2 | Inactivated vaccines | Inactivated vaccines |  | 149 |
| BA.2 | 277300 | Boosted | 3 | Inactivated vaccines | Inactivated vaccines | Inactivated vaccines | 48 |
| BA.2 | 277301 | Boosted | 3 | Inactivated vaccines | Inactivated vaccines | Inactivated vaccines | 64 |
| BA.2 | 277302 | Unvaccinated | 0 |  |  |  |  |
| BA.2 | 277305 | Boosted | 3 | Inactivated vaccines | Inactivated vaccines | Inactivated vaccines | 80 |
| BA.2 | 277306 | Boosted | 3 | Inactivated vaccines | Inactivated vaccines | mRNA | 65 |
| BA.2 | 277312 | Fully vaccinated | 2 | Inactivated vaccines | Inactivated vaccines |  | 241 |
| BA.2 | 277316 | Boosted | 3 | Inactivated vaccines | Inactivated vaccines | Inactivated vaccines | 56 |
| BA.2 | 277318 | Boosted | 3 | mRNA | Inactivated vaccines | Inactivated vaccines | 352 |
| BA.2 | 277319 | Fully vaccinated | 2 | Inactivated vaccines | Inactivated vaccines |  | 261 |
| BA.2 | 277321 | Boosted | 3 | Inactivated vaccines | Inactivated vaccines | Inactivated vaccines | 76 |
| BA.2 | 277329 | Boosted | 3 | Inactivated vaccines | Inactivated vaccines | Inactivated vaccines | 44 |
| BA.2 | 277330 | Unvaccinated | 0 |  |  |  |  |
| BA.2 | 277338 | Unvaccinated | 1 | mRNA |  |  | 151 |
| BA.2 | 277339 | Unvaccinated | 1 | mRNA |  |  | 130 |
| BA.2 | 277340 | Unvaccinated | 0 |  |  |  |  |
| BA.2 | 277342 | Fully vaccinated | 2 | Inactivated vaccines | Inactivated vaccines |  | 224 |
| BA.2 | 277343 | Boosted | 3 | Inactivated vaccines | Inactivated vaccines | Inactivated vaccines | 7 |
| BA.2 | 277344 | Unvaccinated | 1 | Inactivated vaccines |  |  | 15 |
| BA.2 | 277345 | Fully vaccinated | 2 | Inactivated vaccines | Inactivated vaccines |  | 253 |
| BA.2 | 277354 | Unvaccinated | 0 |  |  |  |  |
| BA.2 | 277355 | Boosted | 3 | Inactivated vaccines | Inactivated vaccines | Inactivated vaccines | 59 |
| BA.2 | 277357 | Fully vaccinated | 2 | mRNA | mRNA |  | 147 |
| BA.2 | 277370 | Fully vaccinated | 2 | Inactivated vaccines | Inactivated vaccines |  | 77 |
| BA.2 | 277373 | Boosted | 3 | Inactivated vaccines | Inactivated vaccines | Inactivated vaccines | 54 |
| BA.2 | 277374 | Boosted | 3 | Inactivated vaccines | Inactivated vaccines | Inactivated vaccines | 72 |
| BA.2 | 277387 | Unvaccinated | 0 |  |  |  |  |
| BA.2 | 277394 | Fully vaccinated | 2 | mRNA | mRNA |  | 204 |
| BA.2 | 277395 | Fully vaccinated | 2 | mRNA | mRNA |  | 201 |

| BA.2 | 277403 | Boosted | 3 | Inactivated vaccines | Inactivated vaccines | Inactivated vaccines | 39 |
| --- | --- | --- | --- | --- | --- | --- | --- |
| BA.2 | 277406 | Fully vaccinated | 2 | Inactivated vaccines | Inactivated vaccines |  | 238 |
| BA.2 | 277407 | Fully vaccinated | 2 | mRNA | mRNA |  | 225 |
| BA.2 | 277410 | Unvaccinated | 0 |  |  |  |  |
| BA.2 | 277413 | Fully vaccinated | 2 | Inactivated vaccines | Inactivated vaccines |  | 82 |
| BA.2 | 277414 | Fully vaccinated | 2 | Inactivated vaccines | Inactivated vaccines |  | 169 |
| BA.2 | 277416 | Unvaccinated | 0 |  |  |  |  |
| BA.2 | 277418 | Unvaccinated | 0 |  |  |  |  |
| BA.2 | 277421 | Fully vaccinated | 2 | Inactivated vaccines | Inactivated vaccines |  | 45 |
| BA.2 | 277422 | Unvaccinated | 1 | Inactivated vaccines |  |  | 12 |
| BA.2 | 277424 | Boosted | 3 | Inactivated vaccines | Inactivated vaccines | Inactivated vaccines | 15 |
| BA.2 | 277425 | Unvaccinated | 1 | Inactivated vaccines |  |  | 8 |
| BA.2 | 277426 | Boosted | 3 | Inactivated vaccines | Inactivated vaccines | Inactivated vaccines | 50 |
| BA.2 | 277427 | Unvaccinated | 0 |  |  |  |  |
| BA.2 | 277428 | Fully vaccinated | 2 | Inactivated vaccines | Inactivated vaccines |  | 215 |
| BA.2 | 277437 | Fully vaccinated | 2 | Inactivated vaccines | Inactivated vaccines |  | 230 |
| BA.2 | 277439 | Fully vaccinated | 2 | Inactivated vaccines | Inactivated vaccines |  | 171 |
| BA.2 | 277440 | Boosted | 3 | Inactivated vaccines | Inactivated vaccines | Inactivated vaccines | 90 |
| BA.2 | 277442 | Boosted | 3 | Inactivated vaccines | Inactivated vaccines | Inactivated vaccines | 65 |
| BA.2 | 277443 | Boosted | 3 | Inactivated vaccines | Inactivated vaccines | Inactivated vaccines | 43 |
| BA.2 | 277445 | Fully vaccinated | 2 | Inactivated vaccines | Inactivated vaccines |  | 213 |
| BA.2 | 277448 | Unvaccinated | 0 |  |  |  |  |
| BA.2 | 277449 | Unvaccinated | 0 |  |  |  |  |
| BA.2 | 277450 | Unvaccinated | 0 | #N/A | #N/A | #N/A |  |
| BA.2 | 277451 | Unvaccinated | 0 |  |  |  |  |
| BA.2 | 277452 | Fully vaccinated | 2 | Inactivated vaccines | Inactivated vaccines |  | 249 |
| BA.2 | 277453 | Fully vaccinated | 2 | Inactivated vaccines | Inactivated vaccines |  | 238 |
| BA.2 | 277454 | Boosted | 3 | Inactivated vaccines | Inactivated vaccines | Inactivated vaccines | 50 |
| BA.2 | 277455 | Unvaccinated | 1 | Inactivated vaccines |  |  | 4 |
| BA.2 | 277456 | Unvaccinated | 0 |  |  |  |  |
| BA.2 | 277457 | Boosted | 3 | Inactivated vaccines | Inactivated vaccines | Inactivated vaccines | 62 |
| BA.2 | 277458 | Unvaccinated | 0 |  |  |  |  |
| BA.2 | 277459 | Fully vaccinated | 2 | Inactivated vaccines | Inactivated vaccines |  | 187 |
| BA.2 | 277460 | Fully vaccinated | 2 | Inactivated vaccines | Inactivated vaccines |  | 193 |
| BA.2 | 277462 | Unvaccinated | 1 | Inactivated vaccines |  |  | 12 |
| BA.2 | 277463 | Boosted | 3 | Inactivated vaccines | Inactivated vaccines | Inactivated vaccines | 8 |

| BA.2 | 277464 | Boosted | 3 | Inactivated vaccines | Inactivated vaccines | Inactivated vaccines | 84 |
| --- | --- | --- | --- | --- | --- | --- | --- |
| BA.2 | 277465 | Boosted | 3 | Inactivated vaccines | Inactivated vaccines | Inactivated vaccines | 61 |
| BA.2 | 277466 | Fully vaccinated | 2 | Inactivated vaccines | Inactivated vaccines |  | 166 |
| BA.2 | 277467 | Boosted | 3 | Inactivated vaccines | Inactivated vaccines | Inactivated vaccines | 11 |
| BA.2 | 277468 | Boosted | 3 | Inactivated vaccines | Inactivated vaccines | mRNA | 31 |
| BA.2 | 277469 | Fully vaccinated | 2 | Inactivated vaccines | Inactivated vaccines |  | 171 |
| BA.2 | 277470 | Unvaccinated | 0 |  |  |  |  |
| BA.2 | 277474 | Fully vaccinated | 2 | Inactivated vaccines | Inactivated vaccines |  | 185 |
| BA.2 | 277477 | Unvaccinated | 0 |  |  |  |  |
| BA.2 | 277484 | Boosted | 3 | Inactivated vaccines | Inactivated vaccines | mRNA | 9 |
| BA.2 | 277485 | Boosted | 3 | Inactivated vaccines | Inactivated vaccines | Inactivated vaccines | 5 |
| BA.2 | 277487 | Unvaccinated | 1 | mRNA |  |  | 152 |
| BA.2 | 277488 | Boosted | 3 | Inactivated vaccines | Inactivated vaccines | Inactivated vaccines | 13 |
| BA.2 | 277489 | Boosted | 3 | Inactivated vaccines | Inactivated vaccines | Inactivated vaccines | 49 |
| BA.2 | 277491 | Fully vaccinated | 2 | mRNA | mRNA |  | 268 |
| BA.2 | 277496 | Fully vaccinated | 2 | Inactivated vaccines | Inactivated vaccines |  | 254 |
| BA.2 | 277510 | Fully vaccinated | 2 | Inactivated vaccines | Inactivated vaccines |  | 210 |
| BA.2 | 277511 | Boosted | 3 | Inactivated vaccines | Inactivated vaccines | Inactivated vaccines | 59 |
| BA.2 | 277514 | Fully vaccinated | 2 | Inactivated vaccines | Inactivated vaccines |  | 233 |
| BA.2 | 277518 | Boosted | 3 | Inactivated vaccines | Inactivated vaccines | Inactivated vaccines | 96 |
| BA.2 | 277524 | Boosted | 3 | Inactivated vaccines | Inactivated vaccines | Inactivated vaccines | 69 |
| BA.2 | 277536 | Boosted | 3 | Inactivated vaccines | Inactivated vaccines | Inactivated vaccines | 64 |
| BA.2 | 277538 | Unvaccinated | 0 |  |  |  |  |
| BA.2 | 277539 | Fully vaccinated | 2 | Inactivated vaccines | Inactivated vaccines |  | 312 |
| BA.2 | 277541 | Boosted | 3 | Inactivated vaccines | Inactivated vaccines | Inactivated vaccines | 8 |
| BA.2 | 277544 | Fully vaccinated | 2 | Inactivated vaccines | Inactivated vaccines |  | 310 |
| BA.2 | 277545 | Boosted | 3 | Inactivated vaccines | Inactivated vaccines | Inactivated vaccines | 15 |
| BA.2 | 277549 | Fully vaccinated | 2 | Inactivated vaccines | Inactivated vaccines |  | 66 |
| BA.2 | 277550 | Boosted | 3 | Inactivated vaccines | Inactivated vaccines | Inactivated vaccines | 23 |
| BA.2 | 277563 | Fully vaccinated | 2 | Inactivated vaccines | Inactivated vaccines |  | 171 |
| BA.2 | 277565 | Boosted | 3 | Inactivated vaccines | Inactivated vaccines | Inactivated vaccines | 72 |
| BA.2 | 277569 | Unvaccinated | 0 |  |  |  |  |
| BA.2 | 277584 | Fully vaccinated | 2 | Inactivated vaccines | Inactivated vaccines |  | 179 |
| BA.2 | 277585 | Fully vaccinated | 2 | Inactivated vaccines | Inactivated vaccines |  | 249 |
| BA.2 | 277589 | Fully vaccinated | 2 | Inactivated vaccines | Inactivated vaccines |  | 151 |
| BA.2 | 277592 | Unvaccinated | 0 |  |  |  |  |

| BA.2 | 277593 | Boosted | 3 | Inactivated vaccines | Inactivated vaccines | Inactivated vaccines | 92 |
| --- | --- | --- | --- | --- | --- | --- | --- |
| BA.2 | 277595 | Boosted | 3 | Inactivated vaccines | Inactivated vaccines | Inactivated vaccines | 41 |
| BA.2 | 277596 | Fully vaccinated | 2 | Inactivated vaccines | Inactivated vaccines |  | 210 |
| BA.2 | 277599 | Unvaccinated | 1 | Inactivated vaccines |  |  | 8 |
| BA.2 | 277600 | Unvaccinated | 0 |  |  |  |  |
| BA.2 | 277601 | Unvaccinated | 0 |  |  |  |  |
| BA.2 | 277602 | Unvaccinated | 0 |  |  |  |  |
| BA.2 | 277604 | Fully vaccinated | 2 | Inactivated vaccines | Inactivated vaccines |  | 198 |
| BA.2 | 277607 | Boosted | 3 | Inactivated vaccines | Inactivated vaccines | Inactivated vaccines | 47 |
| BA.2 | 277610 | Fully vaccinated | 2 | Inactivated vaccines | Inactivated vaccines |  | 397 |
| BA.2 | 277611 | Fully vaccinated | 2 | Inactivated vaccines | Inactivated vaccines |  | 153 |
| BA.2 | 277612 | Fully vaccinated | 2 | Inactivated vaccines | Inactivated vaccines |  | 170 |
| BA.2 | 277613 | Fully vaccinated | 2 | Inactivated vaccines | Inactivated vaccines |  | 224 |
| BA.2 | 277614 | Boosted | 3 | Inactivated vaccines | Inactivated vaccines | Inactivated vaccines | 78 |
| BA.2 | 277616 | Boosted | 3 | Inactivated vaccines | Inactivated vaccines | Inactivated vaccines | 85 |
| BA.2 | 277620 | Boosted | 3 | Inactivated vaccines | Inactivated vaccines | Inactivated vaccines | 61 |
| BA.2 | 277621 | Boosted | 3 | Inactivated vaccines | Inactivated vaccines | mRNA | 27 |
| BA.2 | 277622 | Fully vaccinated | 2 | mRNA | mRNA |  | 194 |
| BA.2 | 277625 | Boosted | 3 | Inactivated vaccines | Inactivated vaccines | Inactivated vaccines | 64 |
| BA.2 | 277627 | Fully vaccinated | 2 | Inactivated vaccines | Inactivated vaccines |  | 266 |
| BA.2 | 277628 | Boosted | 3 | Inactivated vaccines | Inactivated vaccines | Inactivated vaccines | 63 |
| BA.2 | 277629 | Fully vaccinated | 2 | Inactivated vaccines | Inactivated vaccines |  | 239 |
| BA.2 | 277632 | Boosted | 3 | Inactivated vaccines | Inactivated vaccines | mRNA | 50 |
| BA.2 | 277635 | Fully vaccinated | 2 | Inactivated vaccines | Inactivated vaccines |  | 55 |
| BA.2 | 277637 | Unvaccinated | 0 |  |  |  |  |
| BA.2 | 277638 | Unvaccinated | 0 |  |  |  |  |
| BA.2 | 277640 | Boosted | 3 | Inactivated vaccines | Inactivated vaccines | Inactivated vaccines | 149 |
| BA.2 | 277642 | Fully vaccinated | 2 | Inactivated vaccines | Inactivated vaccines |  | 182 |
| BA.2 | 277643 | Boosted | 3 | mRNA | mRNA | mRNA | 28 |
| BA.2 | 277648 | Boosted | 3 | mRNA | mRNA | mRNA | 7 |
| BA.2 | 277650 | Unvaccinated | 0 |  |  |  |  |
| BA.2 | 277654 | Fully vaccinated | 2 | Inactivated vaccines | Inactivated vaccines |  | 251 |
| BA.2 | 277655 | Fully vaccinated | 2 | Inactivated vaccines | Inactivated vaccines |  | 273 |
| BA.2 | 277660 | Boosted | 3 | Inactivated vaccines | Inactivated vaccines | Inactivated vaccines | 79 |
| BA.2 | 277661 | Boosted | 3 | Inactivated vaccines | Inactivated vaccines | Inactivated vaccines | 98 |
| BA.2 | 277662 | Boosted | 3 | Inactivated vaccines | Inactivated vaccines | Inactivated vaccines | 83 |

| BA.2 | 277663 | Fully vaccinated | 2 | Inactivated vaccines | Inactivated vaccines |  | 235 |
| --- | --- | --- | --- | --- | --- | --- | --- |
| BA.2 | 277668 | Boosted | 3 | Inactivated vaccines | Inactivated vaccines | Inactivated vaccines | 74 |
| BA.2 | 277671 | Boosted | 3 | Inactivated vaccines | Inactivated vaccines | Inactivated vaccines | 67 |
| BA.2 | 277677 | Fully vaccinated | 2 | Inactivated vaccines | Inactivated vaccines |  | 211 |
| BA.2 | 277689 | Unvaccinated | 0 |  |  |  |  |
| BA.2 | 277690 | Unvaccinated | 0 |  |  |  |  |
| BA.2 | 277700 | Boosted | 3 | Unknown | Unknown | Unknown | 39 |
| BA.2 | 277702 | Boosted | 3 | Inactivated vaccines | Inactivated vaccines | mRNA | 21 |
| BA.2 | 277703 | Fully vaccinated | 2 | Inactivated vaccines | Inactivated vaccines |  | 207 |
| BA.2 | 277704 | Boosted | 3 | mRNA | mRNA | mRNA | 80 |
| BA.2 | 277705 | Fully vaccinated | 2 | Inactivated vaccines | Inactivated vaccines |  | 241 |
| BA.2 | 277706 | Fully vaccinated | 2 | Inactivated vaccines | Inactivated vaccines |  | 204 |
| BA.2 | 277709 | Fully vaccinated | 2 | Unknown | Unknown |  |  |
| BA.2 | 277710 | Unvaccinated | 0 |  |  |  |  |
| BA.2 | 277711 | Unvaccinated | 0 |  |  |  |  |
| BA.2 | 277712 | Boosted | 3 | Inactivated vaccines | Inactivated vaccines | Inactivated vaccines | 56 |
| BA.2 | 277714 | Fully vaccinated | 2 | Unknown | Unknown |  | 310 |
| BA.2 | 277715 | Boosted | 3 | Inactivated vaccines | Inactivated vaccines | Inactivated vaccines | 96 |
| BA.2 | 277716 | Fully vaccinated | 1 | Adenovirus-vectored vaccine |  |  | 257 |
| BA.2 | 277718 | Fully vaccinated | 2 | Inactivated vaccines | Inactivated vaccines |  | 234 |
| BA.2 | 277720 | Boosted | 3 | Unknown | Unknown | Unknown |  |
| BA.2 | 277722 | Fully vaccinated | 2 | Unknown | Unknown |  |  |
| BA.2 | 277723 | Fully vaccinated | 2 | Inactivated vaccines | Inactivated vaccines |  | 213 |
| BA.2 | 277724 | Boosted | 3 | Unknown | Unknown | Unknown |  |
| BA.2 | 277725 | Boosted | 3 | Unknown | Unknown | Unknown |  |
| BA.2 | 277726 | Fully vaccinated | 2 | Inactivated vaccines | Inactivated vaccines |  | 174 |
| BA.2 | 277727 | Fully vaccinated | 2 | Inactivated vaccines | Inactivated vaccines |  | 192 |
| BA.2 | 277728 | Unvaccinated | 0 |  |  |  |  |
| BA.2 | 277730 | Fully vaccinated | 2 | Unknown | Unknown |  |  |
| BA.2 | 277731 | Boosted | 3 | Inactivated vaccines | Inactivated vaccines | Inactivated vaccines | 99 |
| BA.2 | 277733 | Boosted | 3 | Unknown | Unknown | Unknown |  |
| BA.2 | 277734 | Fully vaccinated | 2 | Inactivated vaccines | Inactivated vaccines |  | 193 |
| BA.2 | 277736 | Boosted | 3 | Inactivated vaccines | Inactivated vaccines | Inactivated vaccines | 14 |
| BA.2 | 277738 | Boosted | 3 | Inactivated vaccines | Inactivated vaccines | Inactivated vaccines | 31 |
| BA.2 | 277739 | Fully vaccinated | 2 | Unknown | Unknown |  |  |
| BA.2 | 277740 | Fully vaccinated | 2 | Unknown | Unknown |  |  |

| BA.2 | 277741 | Fully vaccinated | 2 | Unknown | Unknown |  |  |
| --- | --- | --- | --- | --- | --- | --- | --- |
| BA.2 | 277742 | Boosted | 3 | Inactivated vaccines | Inactivated vaccines | Inactivated vaccines | 52 |
| BA.2 | 277743 | Fully vaccinated | 2 | Unknown | Unknown |  |  |
| BA.2 | 277744 | Fully vaccinated | 2 | Inactivated vaccines | Inactivated vaccines |  | 246 |
| BA.2 | 277747 | Boosted | 3 | Inactivated vaccines | Inactivated vaccines | Inactivated vaccines | 75 |
| BA.2 | 277754 | Fully vaccinated | 2 | Inactivated vaccines | Inactivated vaccines |  | 163 |
| BA.2 | 277756 | Fully vaccinated | 2 | mRNA | mRNA |  | 6 |
| BA.2 | 277758 | Boosted | 3 | Recombinant Subunit Vaccines | Recombinant Subunit Vaccines | ombinant Subunit Vacc | 213 |
| BA.2 | 277759 | Fully vaccinated | 2 | Inactivated vaccines | Inactivated vaccines |  | 243 |
| BA.2 | 277761 | Boosted | 3 | Inactivated vaccines | Inactivated vaccines | Inactivated vaccines | 54 |
| BA.2 | 277762 | Fully vaccinated | 2 | Inactivated vaccines | Inactivated vaccines |  | 240 |
| BA.2 | 277763 | Fully vaccinated | 2 | Inactivated vaccines | Inactivated vaccines |  | 245 |
| BA.2 | 277764 | Boosted | 3 | Inactivated vaccines | Inactivated vaccines | Inactivated vaccines | 69 |
| BA.2 | 277768 | Boosted | 3 | Inactivated vaccines | Inactivated vaccines | Inactivated vaccines | 39 |
| BA.2 | 277770 | Unvaccinated | 1 | Inactivated vaccines |  |  | 27 |
| BA.2 | 277774 | Boosted | 3 | Inactivated vaccines | Inactivated vaccines | Inactivated vaccines | 65 |
| BA.2 | 277784 | Fully vaccinated | 2 | Unknown | Unknown |  |  |
| BA.2 | 277786 | Boosted | 3 | Inactivated vaccines | Inactivated vaccines | Inactivated vaccines | 418 |
| BA.2 | 277814 | Boosted | 3 | Inactivated vaccines | Inactivated vaccines | Inactivated vaccines | 105 |
| BA.2 | 277815 | Boosted | 3 | Inactivated vaccines | Inactivated vaccines | Inactivated vaccines | 104 |
| BA.2 | 277822 | Unvaccinated | 0 |  |  |  |  |
| BA.2 | 277825 | Fully vaccinated | 2 | Inactivated vaccines | Inactivated vaccines |  | 210 |
| BA.2 | 277826 | Boosted | 3 | Inactivated vaccines | Inactivated vaccines | Inactivated vaccines | 74 |
| BA.2 | 277828 | Fully vaccinated | 2 | Inactivated vaccines | Inactivated vaccines |  | 148 |
| BA.2 | 277829 | Boosted | 3 | Inactivated vaccines | Inactivated vaccines | Inactivated vaccines | 46 |
| BA.2 | 277833 | Boosted | 3 | Inactivated vaccines | Inactivated vaccines | Inactivated vaccines | 24 |
| BA.2 | 277834 | Boosted | 3 | Inactivated vaccines | Inactivated vaccines | Inactivated vaccines | 297 |
| BA.2 | 277837 | Fully vaccinated | 2 | Inactivated vaccines | Inactivated vaccines |  | 215 |
| BA.2 | 277851 | Boosted | 3 | Inactivated vaccines | Inactivated vaccines | Inactivated vaccines | 55 |
| BA.2 | 277852 | Boosted | 3 | Unknown | Unknown | Unknown |  |
| BA.2 | 277853 | Boosted | 3 | Unknown | Unknown | Unknown | 392 |
| BA.2 | 277854 | Unvaccinated | 0 |  |  |  |  |
| BA.2 | 277857 | Fully vaccinated | 2 | Inactivated vaccines | Inactivated vaccines |  | 311 |
| BA.2 | 277858 | Unvaccinated | 0 |  |  |  |  |
| BA.2 | 277859 | Boosted | 3 | Inactivated vaccines | Inactivated vaccines | Inactivated vaccines | 76 |
| BA.2 | 277860 | Fully vaccinated | 2 | Inactivated vaccines | Inactivated vaccines |  | 124 |

| BA.2 | 277861 | Boosted | 3 | Inactivated vaccines | Inactivated vaccines | Inactivated vaccines | 90 |
| --- | --- | --- | --- | --- | --- | --- | --- |
| BA.2 | 277863 | Boosted | 3 | Inactivated vaccines | Inactivated vaccines | Inactivated vaccines | 65 |
| BA.2 | 277864 | Fully vaccinated | 2 | Inactivated vaccines | Inactivated vaccines |  | 175 |
| BA.2 | 277871 | Fully vaccinated | 2 | Inactivated vaccines | Inactivated vaccines |  | 241 |
| BA.2 | 277873 | Boosted | 3 | Inactivated vaccines | Inactivated vaccines | Inactivated vaccines | 76 |
| BA.2 | 277877 | Boosted | 3 | Inactivated vaccines | Inactivated vaccines | Inactivated vaccines | 64 |
| BA.2 | 277879 | Fully vaccinated | 2 | Inactivated vaccines | Inactivated vaccines |  | 91 |
| BA.2 | 277880 | Fully vaccinated | 2 | Inactivated vaccines | Inactivated vaccines |  | 241 |
| BA.2 | 277881 | Boosted | 3 | Inactivated vaccines | Inactivated vaccines | Inactivated vaccines | 63 |
| BA.2 | 277882 | Fully vaccinated | 2 | Unknown | Unknown |  |  |
| BA.2 | 277883 | Boosted | 3 | Inactivated vaccines | Inactivated vaccines | Inactivated vaccines | 140 |
| BA.2 | 277884 | Fully vaccinated | 2 | Inactivated vaccines | Inactivated vaccines |  | 140 |
| BA.2 | 277886 | Fully vaccinated | 2 | Inactivated vaccines | Inactivated vaccines |  | 161 |
| BA.2 | 277890 | Fully vaccinated | 2 | Inactivated vaccines | Inactivated vaccines |  | 262 |
| BA.2 | 277891 | Boosted | 3 | Inactivated vaccines | Inactivated vaccines | Inactivated vaccines | 13 |
| BA.2 | 277892 | Unvaccinated | 0 |  |  |  |  |
| BA.2 | 277893 | Unvaccinated | 0 |  |  |  |  |
| BA.2 | 277894 | Boosted | 3 | Inactivated vaccines | Inactivated vaccines | Inactivated vaccines | 101 |
| BA.2 | 277895 | Unvaccinated | 0 | #N/A | #N/A | #N/A |  |
| BA.2 | 277897 | Unvaccinated | 0 | #N/A | #N/A | #N/A |  |
| BA.2 | 277898 | Unvaccinated | 0 | #N/A | #N/A | #N/A |  |
| BA.2 | 277900 | Fully vaccinated | 2 | mRNA | mRNA |  | 29 |
| BA.2 | 277901 | Fully vaccinated | 1 | Adenovirus-vectored vaccine |  |  | 246 |
| BA.2 | 277902 | Boosted | 3 | Inactivated vaccines | Inactivated vaccines | Inactivated vaccines | 75 |
| BA.2 | 277904 | Unvaccinated | 0 | #N/A | #N/A | #N/A |  |
| BA.2 | 277908 | Unvaccinated | 0 | #N/A | #N/A | #N/A |  |
| BA.2 | 277911 | Boosted | 3 | Unknown | Unknown | Inactivated vaccines | 85 |
| BA.2 | 277913 | Fully vaccinated | 2 | Unknown | Unknown |  |  |
| BA.2 | 277914 | Fully vaccinated | 2 | Inactivated vaccines | Inactivated vaccines |  | 101 |
| BA.2 | 277915 | Unvaccinated | 1 | mRNA |  |  | 19 |
| BA.2 | 277916 | Fully vaccinated | 2 | Inactivated vaccines | Inactivated vaccines |  | 241 |
| BA.2 | 277917 | Boosted | 3 | Inactivated vaccines | Inactivated vaccines | Inactivated vaccines | 4 |
| BA.2 | 277920 | Unvaccinated | 1 | Inactivated vaccines |  |  | 229 |
| BA.2 | 277921 | Fully vaccinated | 2 | Inactivated vaccines | Inactivated vaccines |  | 192 |
| BA.2 | 277922 | Boosted | 3 | Inactivated vaccines | Inactivated vaccines | Inactivated vaccines | 70 |
| BA.2 | 277923 | Boosted | 3 | Inactivated vaccines | Inactivated vaccines | Inactivated vaccines | 74 |

| BA.2 | 277924 | Unvaccinated | 0 |  |  |  |  |
| --- | --- | --- | --- | --- | --- | --- | --- |
| BA.2 | 277934 | Fully vaccinated | 2 | Unknown | Unknown |  | 178 |
| BA.2 | 277947 | Fully vaccinated | 2 | Inactivated vaccines | Inactivated vaccines |  | 208 |
| BA.2 | 277950 | Fully vaccinated | 2 | Inactivated vaccines | Inactivated vaccines |  | 58 |
| BA.2 | 277951 | Unvaccinated | 0 |  |  |  |  |
| BA.2 | 277952 | Unvaccinated | 1 | Inactivated vaccines |  |  | 12 |
| BA.2 | 277954 | Boosted | 3 | Inactivated vaccines | Inactivated vaccines | Inactivated vaccines | 44 |
| BA.2 | 277961 | Fully vaccinated | 2 | mRNA | mRNA |  | 247 |
| BA.2 | 277962 | Boosted | 3 | Inactivated vaccines | Inactivated vaccines | Inactivated vaccines | 80 |
| BA.2 | 277965 | Unvaccinated | 0 |  |  |  |  |
| BA.2 | 277966 | Boosted | 3 | Inactivated vaccines | Inactivated vaccines | Inactivated vaccines | 43 |
| BA.2 | 277968 | Fully vaccinated | 2 | Unknown | Unknown |  | 34 |
| BA.2 | 277970 | Unvaccinated | 0 | #N/A | #N/A | #N/A |  |
| BA.2 | 277974 | Unvaccinated | 0 | #N/A | #N/A | #N/A |  |
| BA.2 | 277977 | Unvaccinated | 0 | #N/A | #N/A | #N/A |  |
| BA.2 | 277981 | Unvaccinated | 1 | Inactivated vaccines |  |  | 12 |
| BA.2 | 277982 | Fully vaccinated | 2 | Inactivated vaccines | Inactivated vaccines |  | 91 |
| BA.2 | 277986 | Fully vaccinated | 2 | Inactivated vaccines | Inactivated vaccines |  | 197 |
| BA.2 | 277987 | Unvaccinated | 0 |  |  |  |  |
| BA.2 | 277988 | Fully vaccinated | 2 | Inactivated vaccines | Inactivated vaccines |  | 67 |
| BA.2 | 277991 | Boosted | 3 | Unknown | Unknown | Unknown | 125 |
| BA.2 | 277993 | Fully vaccinated | 2 | Inactivated vaccines | Inactivated vaccines |  | 252 |
| BA.2 | 277996 | Boosted | 3 | Inactivated vaccines | Inactivated vaccines | Inactivated vaccines | 63 |
| BA.2 | 277997 | Fully vaccinated | 2 | mRNA | mRNA |  | 159 |
| BA.2 | 278001 | Fully vaccinated | 2 | Unknown | Unknown |  | 112 |
| BA.2 | 278003 | Unvaccinated | 1 | Inactivated vaccines |  |  | 8 |
| BA.2 | 278004 | Unvaccinated | 1 | Inactivated vaccines |  |  | 8 |
| BA.2 | 278008 | Fully vaccinated | 2 | Inactivated vaccines | Inactivated vaccines |  | 197 |
| BA.2 | 278009 | Unvaccinated | 0 |  |  |  |  |
| BA.2 | 278010 | Boosted | 3 | Inactivated vaccines | Inactivated vaccines | mRNA | 74 |
| BA.2 | 278013 | Fully vaccinated | 2 | Unknown | Unknown |  | 334 |
| BA.2 | 278014 | Fully vaccinated | 2 | Inactivated vaccines | Inactivated vaccines |  | 234 |
| BA.2 | 278015 | Boosted | 3 | mRNA | mRNA | mRNA | 60 |
| BA.2 | 278017 | Fully vaccinated | 2 | Inactivated vaccines | Inactivated vaccines |  | 188 |
| BA.2 | 278019 | Fully vaccinated | 2 | Inactivated vaccines | Inactivated vaccines |  | 28 |
| BA.2 | 278020 | Unvaccinated | 0 |  |  |  |  |

| BA.2 | 278021 | Unvaccinated | 0 | #N/A | #N/A | #N/A |  |
| --- | --- | --- | --- | --- | --- | --- | --- |
| BA.2 | 278023 | Unvaccinated | 0 | #N/A | #N/A | #N/A |  |
| BA.2 | 278024 | Unvaccinated | 0 | #N/A | #N/A | #N/A |  |
| BA.2 | 278026 | Fully vaccinated | 2 | Inactivated vaccines | Inactivated vaccines |  | 212 |
| BA.2 | 278029 | Fully vaccinated | 2 | Inactivated vaccines | Inactivated vaccines |  | 242 |
| BA.2 | 278036 | Boosted | 3 | Inactivated vaccines | Inactivated vaccines | Inactivated vaccines | 74 |
| BA.2 | 278037 | Fully vaccinated | 2 | Inactivated vaccines | Inactivated vaccines |  | 245 |
| BA.2 | 278038 | Unvaccinated | 0 |  |  |  |  |
| BA.2 | 278039 | Unvaccinated | 1 | Inactivated vaccines |  |  | 13 |
| BA.2 | 278056 | Fully vaccinated | 2 | mRNA | mRNA |  | 233 |
| BA.2 | 278063 | Unvaccinated | 1 | Inactivated vaccines |  |  | 11 |
| BA.2 | 278076 | Unvaccinated | 0 |  |  |  |  |
| BA.2 | 278078 | Fully vaccinated | 2 | Inactivated vaccines | Inactivated vaccines |  | 242 |
| BA.2 | 278082 | Boosted | 3 | Inactivated vaccines | Inactivated vaccines | Inactivated vaccines | 42 |
| BA.2 | 278089 | Unvaccinated | 0 | #N/A | #N/A | #N/A |  |
| BA.2 | 278093 | Fully vaccinated | 2 | mRNA | mRNA |  | 179 |
| BA.2 | 278097 | Boosted | 3 | Inactivated vaccines | Inactivated vaccines | ombinant Subunit Vacc | 88 |
| BA.2 | 278101 | Unvaccinated | 0 |  |  |  |  |
| BA.2 | 278102 | Boosted | 3 | Inactivated vaccines | Inactivated vaccines | Inactivated vaccines | 102 |
| BA.2 | 278115 | Unvaccinated | 1 | Inactivated vaccines |  |  | 91 |
| BA.2 | 278116 | Fully vaccinated | 2 | Inactivated vaccines | Inactivated vaccines |  | 291 |
| BA.2 | 278117 | Boosted | 3 | Inactivated vaccines | Inactivated vaccines | Inactivated vaccines | 18 |
| BA.2 | 278118 | Boosted | 3 | Inactivated vaccines | Inactivated vaccines | Inactivated vaccines | 63 |
| BA.2 | 278119 | Fully vaccinated | 2 | Inactivated vaccines | Inactivated vaccines |  | 234 |
| BA.2 | 278120 | Boosted | 3 | Inactivated vaccines | Inactivated vaccines | Inactivated vaccines | 90 |
| BA.2 | 278121 | Boosted | 3 | Inactivated vaccines | Inactivated vaccines | Inactivated vaccines | 107 |
| BA.2 | 278122 | Fully vaccinated | 2 | Inactivated vaccines | Inactivated vaccines |  | 251 |
| BA.2 | 278126 | Fully vaccinated | 2 | Inactivated vaccines | Inactivated vaccines |  | 242 |
| BA.2 | 278128 | Boosted | 3 | Inactivated vaccines | Inactivated vaccines | Inactivated vaccines | 68 |
| BA.2 | 278129 | Boosted | 3 | Inactivated vaccines | Inactivated vaccines | Inactivated vaccines | 68 |
| BA.2 | 278131 | Unvaccinated | 0 | #N/A | #N/A | #N/A |  |
| BA.2 | 278132 | Unvaccinated | 0 | #N/A | #N/A | #N/A |  |
| BA.2 | 278133 | Unvaccinated | 0 | #N/A | #N/A | #N/A |  |
| BA.2 | 278135 | Unvaccinated | 0 | #N/A | #N/A | #N/A |  |
| BA.2 | 278165 | Fully vaccinated | 2 | mRNA | mRNA |  | 258 |
| BA.2 | 278166 | Unvaccinated | 0 | #N/A | #N/A | #N/A |  |

| BA.2 | 278170 | Boosted | 3 | Inactivated vaccines | Inactivated vaccines | Inactivated vaccines | 50 |
| --- | --- | --- | --- | --- | --- | --- | --- |
| BA.2 | 278179 | Boosted | 3 | Inactivated vaccines | Inactivated vaccines | Inactivated vaccines | 150 |
| BA.2 | 278183 | Fully vaccinated | 2 | Inactivated vaccines | Inactivated vaccines |  | 80 |
| BA.2 | 278196 | Boosted | 3 | Inactivated vaccines | Inactivated vaccines | Inactivated vaccines | 33 |
| BA.2 | 278198 | Boosted | 3 | Inactivated vaccines | Inactivated vaccines | Inactivated vaccines | 170 |
| BA.2 | 278200 | Boosted | 3 | Inactivated vaccines | Inactivated vaccines | Inactivated vaccines | 78 |
| BA.2 | 278201 | Boosted | 3 | Inactivated vaccines | Inactivated vaccines | Inactivated vaccines | 101 |
| BA.2 | 278202 | Fully vaccinated | 2 | Inactivated vaccines | Inactivated vaccines |  | 94 |
| BA.2 | 278203 | Unvaccinated | 0 |  |  |  |  |
| BA.2 | 278210 | Boosted | 3 | Unknown | Unknown | Unknown |  |
| BA.2 | 278233 | Fully vaccinated | 2 | Inactivated vaccines | Inactivated vaccines |  | 232 |
| BA.2 | 278235 | Boosted | 3 | Inactivated vaccines | Inactivated vaccines | Inactivated vaccines |  |
| BA.2 | 278246 | Boosted | 3 | Inactivated vaccines | Inactivated vaccines | Inactivated vaccines | 63 |
| BA.2 | 278249 | Fully vaccinated | 2 | Inactivated vaccines | Inactivated vaccines |  | 251 |
| BA.2 | 278254 | Boosted | 3 | Inactivated vaccines | Inactivated vaccines | Inactivated vaccines | 71 |
| BA.2 | 278255 | Fully vaccinated | 2 | Inactivated vaccines | Inactivated vaccines |  | 231 |
| BA.2 | 278256 | Fully vaccinated | 2 | Inactivated vaccines | Inactivated vaccines |  | 252 |
| BA.2 | 278257 | Fully vaccinated | 2 | Inactivated vaccines | Inactivated vaccines |  | 223 |
| BA.2 | 278258 | Fully vaccinated | 2 | Inactivated vaccines | Inactivated vaccines |  | 252 |
| BA.2 | 278259 | Fully vaccinated | 2 | Inactivated vaccines | Inactivated vaccines |  | 225 |
| BA.2 | 278260 | Fully vaccinated | 2 | Inactivated vaccines | Inactivated vaccines |  | 134 |
| BA.2 | 278261 | Fully vaccinated | 2 | Inactivated vaccines | Inactivated vaccines |  | 188 |
| BA.2 | 278263 | Fully vaccinated | 2 | Inactivated vaccines | Inactivated vaccines |  | 189 |
| BA.2 | 278264 | Fully vaccinated | 2 | Inactivated vaccines | Inactivated vaccines |  | 203 |
| BA.2 | 278266 | Boosted | 3 | Inactivated vaccines | Inactivated vaccines | Inactivated vaccines | 13 |
| BA.2 | 278269 | Boosted | 3 | Inactivated vaccines | Inactivated vaccines | Inactivated vaccines | 84 |
| BA.2 | 278270 | Boosted | 3 | Inactivated vaccines | Inactivated vaccines | Inactivated vaccines | 84 |
| BA.2 | 278280 | Fully vaccinated | 2 | Inactivated vaccines | Inactivated vaccines |  | 243 |
| BA.2 | 278281 | Fully vaccinated | 2 | Recombinant Subunit Vaccines | Recombinant Subunit Vaccines |  | 174 |
| BA.2 | 278283 | Unvaccinated | 1 | Inactivated vaccines |  |  | 8 |
| BA.2 | 278286 | Boosted | 3 | Inactivated vaccines | Inactivated vaccines | Inactivated vaccines | 152 |
| BA.2 | 278293 | Unvaccinated | 1 | Inactivated vaccines |  |  | 10 |
| BA.2 | 278294 | Boosted | 3 | Inactivated vaccines | Inactivated vaccines | Inactivated vaccines | 75 |
| BA.2 | 278297 | Unvaccinated | 0 | #N/A | #N/A | #N/A |  |
| BA.2 | 278298 | Unvaccinated | 0 | #N/A | #N/A | #N/A |  |
| BA.2 | 278303 | Unvaccinated | 0 | #N/A | #N/A | #N/A |  |

| BA.2 | 278304 | Unvaccinated | 0 | #N/A | #N/A | #N/A |  |
| --- | --- | --- | --- | --- | --- | --- | --- |
| BA.2 | 278307 | Unvaccinated | 0 | #N/A | #N/A | #N/A |  |
| BA.2 | 278308 | Unvaccinated | 0 | #N/A | #N/A | #N/A |  |
| BA.2 | 278312 | Unvaccinated | 0 | #N/A | #N/A | #N/A |  |
| BA.2 | 278315 | Unvaccinated | 0 | #N/A | #N/A | #N/A |  |
| BA.2 | 278317 | Unvaccinated | 1 | Inactivated vaccines |  |  | 243 |
| BA.2 | 278318 | Unvaccinated | 1 | Inactivated vaccines |  |  | 15 |
| BA.2 | 278319 | Unvaccinated | 0 |  |  |  |  |
| BA.2 | 278320 | Fully vaccinated | 2 | Inactivated vaccines | Inactivated vaccines |  | 6 |
| BA.2 | 278328 | Fully vaccinated | 2 | Inactivated vaccines | Inactivated vaccines |  | 253 |
| BA.2 | 278333 | Boosted | 3 | Inactivated vaccines | Inactivated vaccines | Inactivated vaccines | 18 |
| BA.2 | 278337 | Boosted | 3 | Inactivated vaccines | Inactivated vaccines | Inactivated vaccines | 53 |
| BA.2 | 278339 | Boosted | 3 | Inactivated vaccines | Inactivated vaccines | Inactivated vaccines | 9 |
| BA.2 | 278343 | Unvaccinated | 1 | Inactivated vaccines |  |  | 19 |
| BA.2 | 278347 | Fully vaccinated | 2 | Inactivated vaccines | Inactivated vaccines |  | 168 |
| BA.2 | 278349 | Fully vaccinated | 2 | Inactivated vaccines | Inactivated vaccines |  | 243 |
| BA.2 | 278352 | Boosted | 3 | Inactivated vaccines | Inactivated vaccines | Inactivated vaccines | 71 |
| BA.2 | 278353 | Boosted | 3 | Inactivated vaccines | Inactivated vaccines | Inactivated vaccines |  |
| BA.2 | 278360 | Boosted | 3 | Inactivated vaccines | Inactivated vaccines | Inactivated vaccines | 67 |
| BA.2 | 278361 | Boosted | 3 | Inactivated vaccines | Inactivated vaccines | Inactivated vaccines | 26 |
| BA.2 | 278363 | Boosted | 3 | Inactivated vaccines | Inactivated vaccines | Inactivated vaccines | 32 |
| BA.2 | 278367 | Boosted | 3 | Unknown | Unknown | Unknown | 66 |
| BA.2 | 278368 | Fully vaccinated | 2 | Inactivated vaccines | Unknown |  | 175 |
| BA.2 | 278369 | Fully vaccinated | 2 | Unknown | Unknown |  |  |
| BA.2 | 278371 | Boosted | 3 | Inactivated vaccines | Inactivated vaccines | Inactivated vaccines | 68 |
| BA.2 | 278372 | Fully vaccinated | 2 | Unknown | Unknown |  |  |
| BA.2 | 278373 | Fully vaccinated | 2 | Inactivated vaccines | Inactivated vaccines |  | 212 |
| BA.2 | 278375 | Unvaccinated | 0 |  |  |  |  |
| BA.2 | 278378 | Unvaccinated | 0 |  |  |  |  |
| BA.2 | 278381 | Unvaccinated | 1 | Inactivated vaccines |  |  | 31 |
| BA.2 | 278393 | Unvaccinated | 0 |  |  |  |  |
| BA.2 | 278394 | Boosted | 3 | Inactivated vaccines | Inactivated vaccines | Inactivated vaccines | 61 |
| BA.2 | 278396 | Boosted | 3 | Inactivated vaccines | Inactivated vaccines | Inactivated vaccines | 65 |
| BA.2 | 278397 | Fully vaccinated | 2 | Inactivated vaccines | Inactivated vaccines |  | 248 |
| BA.2 | 278399 | Fully vaccinated | 2 | Inactivated vaccines | Inactivated vaccines |  | 154 |
| BA.2 | 278400 | Boosted | 3 | Inactivated vaccines | Inactivated vaccines | Inactivated vaccines | 53 |

| BA.2 | 278425 | Fully vaccinated | 2 | Inactivated vaccines | Inactivated vaccines |  | 416 |
| --- | --- | --- | --- | --- | --- | --- | --- |
| BA.2 | 278426 | Fully vaccinated | 2 | Inactivated vaccines | Inactivated vaccines |  | 163 |
| BA.2 | 278432 | Fully vaccinated | 2 | Inactivated vaccines | Inactivated vaccines |  | 189 |
| BA.2 | 278434 | Unvaccinated | 0 |  |  |  |  |
| BA.2 | 278437 | Boosted | 3 | Inactivated vaccines | Inactivated vaccines | Inactivated vaccines | 250 |
| BA.2 | 278438 | Fully vaccinated | 2 | Inactivated vaccines | Inactivated vaccines |  | 144 |
| BA.2 | 278440 | Fully vaccinated | 2 | Inactivated vaccines | Inactivated vaccines |  | 249 |
| BA.2 | 278441 | Unvaccinated | 0 |  |  |  |  |
| BA.2 | 278442 | Fully vaccinated | 2 | Inactivated vaccines | Inactivated vaccines |  | 177 |
| BA.2 | 278443 | Fully vaccinated | 2 | Inactivated vaccines | Inactivated vaccines |  | 94 |
| BA.2 | 278444 | Unvaccinated | 0 |  |  |  |  |
| BA.2 | 278445 | Unvaccinated | 0 |  |  |  |  |
| BA.2 | 278446 | Boosted | 3 | Inactivated vaccines | Inactivated vaccines | mRNA | 101 |
| BA.2 | 278454 | Unvaccinated | 1 | Inactivated vaccines |  |  | 13 |
| BA.2 | 278455 | Fully vaccinated | 2 | mRNA | mRNA |  | 227 |
| BA.2 | 278456 | Fully vaccinated | 2 | mRNA | mRNA |  | 190 |
| BA.2 | 278457 | Boosted | 3 | Inactivated vaccines | Inactivated vaccines | Inactivated vaccines | 4 |
| BA.2 | 278458 | Boosted | 3 | Inactivated vaccines | Inactivated vaccines | Inactivated vaccines | 75 |
| BA.2 | 278459 | Fully vaccinated | 2 | Inactivated vaccines | Inactivated vaccines |  | 165 |
| BA.2 | 278461 | Unvaccinated | 1 | Inactivated vaccines |  |  | 4 |
| BA.2 | 278462 | Boosted | 3 | mRNA | mRNA | mRNA | 47 |
| BA.2 | 278463 | Boosted | 3 | Inactivated vaccines | Inactivated vaccines | Inactivated vaccines | 107 |
| BA.2 | 278464 | Boosted | 3 | Inactivated vaccines | Inactivated vaccines | Inactivated vaccines | 71 |
| BA.2 | 278465 | Unvaccinated | 1 | mRNA |  |  | 74 |
| BA.2 | 278467 | Boosted | 3 | Inactivated vaccines | Inactivated vaccines | Inactivated vaccines | 79 |
| BA.2 | 278468 | Boosted | 3 | Inactivated vaccines | Inactivated vaccines | Inactivated vaccines | 62 |
| BA.2 | 278475 | Unvaccinated | 0 | #N/A | #N/A | #N/A |  |
| BA.2 | 278478 | Fully vaccinated | 2 | Inactivated vaccines | Inactivated vaccines |  | 61 |
| BA.2 | 278482 | Unvaccinated | 1 | Inactivated vaccines |  |  | 9 |
| BA.2 | 278489 | Boosted | 3 | Inactivated vaccines | Inactivated vaccines | Inactivated vaccines | 68 |
| BA.2 | 278506 | Unvaccinated | 1 | Inactivated vaccines |  |  | 14 |
| BA.2 | 278514 | Fully vaccinated | 2 | mRNA | mRNA |  | 191 |
| BA.2 | 278523 | Fully vaccinated | 2 | Inactivated vaccines | Inactivated vaccines |  | 146 |
| BA.2 | 280497 | Unvaccinated | 0 |  |  |  |  |
| BA.2 | 280502 | Boosted | 3 | Inactivated vaccines | Inactivated vaccines | Inactivated vaccines | 86 |
| BA.2 | 280512 | Fully vaccinated | 2 | Inactivated vaccines | Inactivated vaccines |  | 277 |

| BA.2 | 280561 | Boosted | 3 | Inactivated vaccines | Inactivated vaccines | Inactivated vaccines | 135 |
| --- | --- | --- | --- | --- | --- | --- | --- |
| BA.2 | 280678 | Boosted | 3 | mRNA | mRNA | mRNA | 93 |
| BA.2 | 280694 | Unvaccinated | 0 |  |  |  |  |
| BA.2 | 280710 | Unvaccinated | 1 |  |  |  |  |
| BA.2 | 280755 | Boosted | 3 | Inactivated vaccines | Inactivated vaccines | Inactivated vaccines | 178 |
| BA.2 | 280795 | Boosted | 3 | Inactivated vaccines | Inactivated vaccines | Inactivated vaccines | 136 |
| BA.2 | 280800 | Boosted | 4 | Inactivated vaccines | Inactivated vaccines | Inactivated vaccines | 297 |
| BA.2 | 280809 | Fully vaccinated | 2 | Unknown | Unknown |  |  |
| BA.2 | 280825 | Unvaccinated | 0 |  |  |  |  |
| BA.2 | 280835 | Fully vaccinated | 2 | Inactivated vaccines | Inactivated vaccines |  | 127 |
| BA.2 | 280836 | Boosted | 3 | Inactivated vaccines | Inactivated vaccines | Inactivated vaccines | 113 |
| BA.2 | 280850 | Boosted | 3 | Inactivated vaccines | Inactivated vaccines | Inactivated vaccines | 103 |
| BA.2 | 280851 | Boosted | 3 | Inactivated vaccines | Inactivated vaccines | Inactivated vaccines | 106 |
| BA.2 | 280935 | Unvaccinated | 0 |  |  |  |  |
| BA.2 | 280961 | Boosted | 3 | Inactivated vaccines | Inactivated vaccines | Inactivated vaccines | 52 |
| BA.2 | 281022 | Boosted | 3 | Inactivated vaccines | Inactivated vaccines | Inactivated vaccines | 115 |
| BA.2 | 281023 | Boosted | 3 | Inactivated vaccines | Inactivated vaccines | Inactivated vaccines | 43 |
| BA.2 | 281024 | Boosted | 3 | Unknown | Unknown | Unknown |  |
| BA.2 | 281025 | Boosted | 3 | Inactivated vaccines | Inactivated vaccines | Inactivated vaccines | 90 |
| BA.2 | 281028 | Boosted | 3 | Inactivated vaccines | Inactivated vaccines | Inactivated vaccines | 185 |
| BA.2 | 281034 | Boosted | 3 | Unknown | Unknown | Unknown |  |
| BA.2 | 281035 | Boosted | 3 | Unknown | Unknown | Unknown |  |
| BA.2 | 281048 | Unvaccinated | 0 |  |  |  |  |
| BA.2 | 281063 | Fully vaccinated | 2 | mRNA | mRNA |  | 327 |
| BA.2 | 281064 | Boosted | 3 | Inactivated vaccines | Inactivated vaccines | Inactivated vaccines | 107 |
| BA.2 | 281079 | Boosted | 3 | Inactivated vaccines | Inactivated vaccines | Inactivated vaccines | 129 |
| BA.2 | 281085 | Boosted | 3 | Inactivated vaccines | Inactivated vaccines | Inactivated vaccines | 80 |
| BA.2 | 281095 | Boosted | 3 | Inactivated vaccines | Inactivated vaccines | Inactivated vaccines | 34 |
| BA.2 | 281096 | Fully vaccinated | 2 | Inactivated vaccines | Inactivated vaccines |  | 147 |
| BA.2 | 281100 | Boosted | 3 | Inactivated vaccines | Inactivated vaccines | Inactivated vaccines | 94 |
| BA.2 | 281101 | Boosted | 3 | Inactivated vaccines | Inactivated vaccines | Inactivated vaccines | 21 |
| BA.2 | 281103 | Boosted | 3 | Inactivated vaccines | Inactivated vaccines | Inactivated vaccines | 103 |
| BA.2 | 281104 | Boosted | 3 | Unknown | Unknown | Unknown |  |
| BA.2 | 281105 | Fully vaccinated | 2 | Inactivated vaccines | Inactivated vaccines |  | 297 |
| BA.2 | 281106 | Boosted | 3 | Inactivated vaccines | Inactivated vaccines | Inactivated vaccines | 104 |
| BA.2 | 281108 | Boosted | 3 | Inactivated vaccines | Inactivated vaccines | Inactivated vaccines | 24 |

| BA.2 | 281109 | Fully vaccinated | 2 | Inactivated vaccines | Inactivated vaccines |  | 226 |
| --- | --- | --- | --- | --- | --- | --- | --- |
| BA.2 | 281110 | Boosted | 3 | Inactivated vaccines | Inactivated vaccines | Inactivated vaccines | 120 |
| BA.2 | 281111 | Boosted | 3 | Inactivated vaccines | Inactivated vaccines | Inactivated vaccines | 278 |
| BA.2 | 281113 | Boosted | 3 | Inactivated vaccines | Inactivated vaccines | Inactivated vaccines | 17 |
| BA.2 | 281114 | Boosted | 3 | Inactivated vaccines | Inactivated vaccines | Inactivated vaccines | 313 |
| BA.2 | 281117 | Boosted | 3 | Unknown | Unknown | Unknown |  |
| BA.2 | 281119 | Boosted | 3 | Inactivated vaccines | Inactivated vaccines | Inactivated vaccines | 170 |
| BA.2 | 281128 | Boosted | 3 | Inactivated vaccines | Inactivated vaccines | Inactivated vaccines | 52 |
| BA.2 | 281129 | Boosted | 3 | Inactivated vaccines | Inactivated vaccines | Inactivated vaccines | 124 |
| BA.2 | 281142 | Boosted | 3 | Unknown | Unknown | Unknown |  |
| BA.2 | 281144 | Fully vaccinated | 2 | Inactivated vaccines | Inactivated vaccines |  | 230 |
| BA.2 | 281146 | Fully vaccinated | 2 | Inactivated vaccines | Inactivated vaccines |  | 252 |
| BA.2 | 281186 | Boosted | 3 | Inactivated vaccines | Inactivated vaccines | Inactivated vaccines | 42 |
| BA.2 | 281187 | Fully vaccinated | 2 | Inactivated vaccines | Inactivated vaccines |  | 313 |
| BA.2 | 281188 | Boosted | 3 | Inactivated vaccines | Inactivated vaccines | Inactivated vaccines | 40 |
| BA.2 | 281189 | Boosted | 3 | Inactivated vaccines | Inactivated vaccines | Inactivated vaccines | 33 |
| BA.2 | 281205 | Boosted | 3 | Inactivated vaccines | Inactivated vaccines | Inactivated vaccines | 10 |
| BA.2 | 281249 | Boosted | 3 | Inactivated vaccines | Inactivated vaccines | Inactivated vaccines | 55 |
| BA.2 | 281250 | Boosted | 3 | Inactivated vaccines | Inactivated vaccines | Inactivated vaccines | 37 |
| BA.2 | 281251 | Boosted | 3 | Inactivated vaccines | Inactivated vaccines | Inactivated vaccines | 107 |
| BA.2 | 281252 | Boosted | 3 | Inactivated vaccines | Inactivated vaccines | Inactivated vaccines | 11 |
| BA.2 | 281257 | Boosted | 3 | Inactivated vaccines | Inactivated vaccines | Inactivated vaccines | 34 |
| BA.2 | 281258 | Boosted | 3 | Inactivated vaccines | Inactivated vaccines | Inactivated vaccines | 260 |
| BA.2 | 281280 | Boosted | 3 | Inactivated vaccines | Inactivated vaccines | Inactivated vaccines | 24 |
| BA.2 | 281299 | Boosted | 3 | Inactivated vaccines | Inactivated vaccines | Inactivated vaccines | 110 |
| BA.2 | 281300 | Fully vaccinated | 2 | Inactivated vaccines | Inactivated vaccines |  | 288 |
| BA.2 | 281312 | Boosted | 3 | Inactivated vaccines | Inactivated vaccines | Inactivated vaccines | 107 |
| BA.2 | 281313 | Fully vaccinated | 2 | Inactivated vaccines | Inactivated vaccines |  | 206 |
| BA.2 | 281314 | Fully vaccinated | 2 | Inactivated vaccines | Inactivated vaccines |  | 291 |
| BA.2 | 281371 | Boosted | 3 | Inactivated vaccines | Inactivated vaccines | Inactivated vaccines | 16 |
| BA.2 | 281469 | Fully vaccinated | 2 | Inactivated vaccines | Inactivated vaccines |  | 257 |
| BA.2 | 281737 | Boosted | 3 | Inactivated vaccines | Inactivated vaccines | mRNA | 90 |
| BA.2 | 281772 | Boosted | 3 | Unknown | Unknown | Unknown | 145 |
| BA.2 | 282057 | Unvaccinated | 0 |  |  |  |  |

| **Variants** | **ID** | **Sex** | **Age** | **Severity** | **Lineages** **(Omicorn)** | **Time** **of** **admission** | **Time** **of** **onset** | **Time** **of** **discharge** | **Hospital** **days** |
| --- | --- | --- | --- | --- | --- | --- | --- | --- | --- |
| BA.5 | 69911 | female | 59 | Mild | BA.5.2 (Omicorn) | 2022-08-31 15:47 | 2022/8/30 | 2022-09- 12 9:33 | 12 |
| BA.5 | 287655 | male | 28 | Mild | BA.5.2 (Omicorn) | 2022-06-22 23:35 | 2022/6/22 | 2022-07-02 13:00 | 10 |
| BA.5 | 288461 | male | 21 | Asymptomatic | BA.5. 1 (Omicorn) | 2022-07-02 0:42 | 2022/7/ 1 | 2022-07- 16 13:30 | 15 |
| BA.5 | 288530 | male | 53 | Mild | BA.5. 1 (Omicorn) | 2022-07-03 0:36 | 2022/7/2 | 2022-07- 18 10:26 | 15 |
| BA.5 | 288532 | male | 24 | Asymptomatic | BA.5. 1 (Omicorn) | 2022-07-03 7:00 | 2022/7/2 | 2022-07- 16 13:30 | 13 |
| BA.5 | 288572 | female | 20 | Asymptomatic | BA.5. 1 (Omicorn) | 2022-07-03 11:34 | 2022/7/2 | 2022-07- 13 19:13 | 10 |
| BA.5 | 288700 | male | 24 | Mild | BA.5.2 (Omicorn) | 2022-07-04 18:40 | 2022/7/4 | 2022-07- 14 13:51 | 10 |
| BA.5 | 288707 | female | 18 | Moderate | BA.5.2 (Omicorn) | 2022-07-04 21:56 | 2022/7/4 | 2022-07- 18 13:33 | 14 |
| BA.5 | 288822 | male | 20 | Mild | BA.5. 1 (Omicorn) | 2022-07-06 0:48 | 2022/7/5 | 2022-07- 16 13:35 | 11 |
| BA.5 | 289508 | male | 39 | Mild | BA.5. 1 (Omicorn) | 2022-07- 12 12:51 | 2022/7/ 11 | 2022-07-23 9:24 | 11 |
| BA.5 | 289528 | male | 54 | Mild | BA.5.2 (Omicorn) | 2022-07- 12 16:37 | 2022/7/ 11 | 2022-07-27 10:57 | 15 |
| BA.5 | 289545 | male | 53 | Mild | BA.5.2. 1 (Omicorn) | 2022-07- 13 2:07 | 2022/7/ 12 | 2022-07-22 12:43 | 9 |
| BA.5 | 289771 | female | 38 | Mild | BA.5. 1 (Omicorn) | 2022-07- 15 11:58 | 2022/7/ 14 | 2022-07- 18 13:32 | 3 |
| BA.5 | 289949 | male | 58 | Moderate | BA.5.2 (Omicorn) | 2022-07- 17 0:43 | 2022/7/ 16 | 2022-07-31 18:51 | 15 |
| BA.5 | 290447 | male | 22 | Mild | BA.5.2. 1 (Omicorn) | 2022-07-20 22:07 | 2022/7/ 19 | 2022-07-31 12:34 | 11 |
| BA.5 | 290449 | male | 21 | Mild | BA.5.2. 1 (Omicorn) | 2022-07-20 22:12 | 2022/7/20 | 2022-07-26 11:19 | 6 |
| BA.5 | 290451 | male | 27 | Mild | BA.5.2. 1 (Omicorn) | 2022-07-20 22:18 | 2022/7/ 19 | 2022-08-01 14:01 | 12 |
| BA.5 | 290452 | male | 28 | Mild | BA.5.2. 1 (Omicorn) | 2022-07-20 22:16 | 2022/7/20 | 2022-07-28 13:38 | 8 |
| BA.5 | 290761 | male | 10 | Mild | BA.5. 1 (Omicorn) | 2022-07-24 4:51 | 2022/7/24 | 2022-08-01 13:37 | 8 |
| BA.5 | 290955 | male | 52 | Mild | BA.5. 1 (Omicorn) | 2022-07-25 20:37 | 2022/7/23 | 2022-08- 13 8:00 | 18 |
| BA.5 | 290970 | male | 33 | Mild | BA.5.2 (Omicorn) | 2022-07-25 22:14 | 2022/7/24 | 2022-08-08 10:44 | 14 |
| BA.5 | 291064 | female | 33 | Mild | BA.5.2 (Omicorn) | 2022-07-26 17:41 | 2022/7/25 | 2022-08-08 10:41 | 13 |
| BA.5 | 291075 | male | 38 | Mild | BA.5.2 (Omicorn) | 2022-07-26 22:45 | 2022/7/25 | 2022-08-07 11:47 | 12 |
| BA.5 | 291091 | female | 32 | Mild | BA.5. 1 (Omicorn) | 2022-07-27 7:35 | 2022/7/26 | 2022-08-07 9:47 | 11 |
| BA.5 | 291207 | male | 61 | Mild | BA.5.2. 1 (Omicorn) | 2022-07-28 10:35 | 2022/7/27 | 2022-08-07 10:10 | 10 |

| BA.5 | 291285 | female | 50 | Mild | BA.5.2 (Omicorn) | 2022-07-29 3:28 | 2022/7/29 | 2022-08-08 11:20 | 10 |
| --- | --- | --- | --- | --- | --- | --- | --- | --- | --- |
| BA.5 | 291286 | female | 2 | Mild | BA.5.2 (Omicorn) | 2022-07-29 3:29 | 2022/7/28 | 2022-08-03 16:56 | 6 |
| BA.5 | 291289 | male | 49 | Asymptomatic | BA.5.2. 1 (Omicorn) | 2022-07-29 9:24 | 2022/7/28 | 2022-08-09 10:00 | 11 |
| BA.5 | 291398 | male | 27 | Mild | BA.5.2 (Omicorn) | 2022-07-30 11:53 | 2022/7/29 | 2022-08-06 10:00 | 7 |
| BA.5 | 291444 | female | 24 | Mild | BA.5.2. 1 (Omicorn) | 2022-07-31 3:40 | 2022/7/30 | 2022-08- 10 11:48 | 10 |
| BA.5 | 291446 | male | 25 | Mild | BA.5.2 (Omicorn) | 2022-07-31 5:42 | 2022/7/30 | 2022-08-08 8:58 | 8 |
| BA.5 | 291494 | male | 35 | Mild | BA.5.2. 1 (Omicorn) | 2022-07-31 15:18 | 2022/7/28 | 2022-08- 14 9:50 | 14 |
| BA.5 | 291495 | male | 25 | Mild | BA.5.2. 1 (Omicorn) | 2022-07-31 15:19 | 2022/7/31 | 2022-08- 10 11:42 | 10 |
| BA.5 | 291500 | male | 54 | Mild | BA.5.2 (Omicorn) | 2022-07-31 15:20 | 2022/7/29 | 2022-08- 15 11:05 | 15 |
| BA.5 | 291501 | male | 39 | Asymptomatic | BA.5.2 (Omicorn) | 2022-07-31 15:18 | 2022/7/30 | 2022-08- 13 17:21 | 13 |
| BA.5 | 291502 | male | 44 | Asymptomatic | BA.5.2 (Omicorn) | 2022-07-31 15:19 | 2022/7/31 | 2022-08- 14 9:54 | 14 |
| BA.5 | 291660 | female | 54 | Mild | BA.5.2. 1 (Omicorn) | 2022-08-02 9:35 | 2022/8/ 1 | 2022-08-05 11:26 | 3 |
| BA.5 | 291662 | male | 47 | Mild | BA.5.2. 1 (Omicorn) | 2022-08-02 10:13 | 2022/8/2 | 2022-08- 13 8:59 | 11 |
| BA.5 | 291696 | male | 28 | Mild | BA.5.2 (Omicorn) | 2022-08-02 15:37 | 2022/8/ 1 | 2022-08- 18 9:42 | 16 |
| BA.5 | 291753 | female | 34 | Mild | BA.5.2. 1 (Omicorn) | 2022-08-02 23:03 | 2022/8/ 1 | 2022-08- 14 10:43 | 11 |
| BA.5 | 291811 | male | 33 | Mild | BA.5.2 (Omicorn) | 2022-08-03 12:49 | 2022/8/2 | 2022-08- 14 10:35 | 11 |
| BA.5 | 291860 | female | 19 | Mild | BA.5.2 (Omicorn) | 2022-08-03 20:49 | 2022/8/2 | 2022-08- 18 10:01 | 15 |
| BA.5 | 291867 | male | 31 | Mild | BA.5.2. 1 (Omicorn) | 2022-08-04 0:43 | 2022/8/3 | 2022-08- 16 10:29 | 12 |
| BA.5 | 291918 | male | 28 | Mild | BA.5.2 (Omicorn) | 2022-08-04 12:24 | 2022/8/ 1 | 2022-08- 15 10:43 | 11 |
| BA.5 | 292048 | male | 50 | Mild | BA.5.2. 1 (Omicorn) | 2022-08-06 3:54 | 2022/8/5 | 2022-08- 15 11:05 | 9 |
| BA.5 | 292049 | female | 30 | Asymptomatic | BA.5.5 (Omicorn) | 2022-08-06 3:56 | 2022/8/5 | 2022-08- 10 11:28 | 4 |
| BA.5 | 292064 | male | 24 | Mild | BA.5.2 (Omicorn) | 2022-08-06 10:13 | 2022/8/5 | 2022-08- 14 9:52 | 8 |
| BA.5 | 292065 | male | 52 | Mild | BA.5.2 (Omicorn) | 2022-08-06 9:58 | 2022/8/5 | 2022-08- 11 11:38 | 5 |
| BA.5 | 292066 | female | 38 | Mild | BA.5.2 (Omicorn) | 2022-08-06 10:00 | 2022/8/5 | 2022-08-21 10:00 | 15 |
| BA.5 | 292067 | male | 33 | Asymptomatic | BA.5.2. 1 (Omicorn) | 2022-08-06 10:02 | 2022/8/5 | 2022-08- 13 9:00 | 7 |
| BA.5 | 292069 | female | 49 | Mild | BA.5.2 (Omicorn) | 2022-08-06 10:24 | 2022/8/4 | 2022-08- 15 11:05 | 9 |
| BA.5 | 292073 | male | 22 | Mild | BA.5.2 (Omicorn) | 2022-08-06 10:20 | 2022/8/6 | 2022-08- 17 15:15 | 11 |

| BA.5 | 292078 | female | 25 | Mild | BF.5 (Omicorn) | 2022-08-06 11:27 | 2022/8/5 | 2022-08- 17 17:31 | 11 |
| --- | --- | --- | --- | --- | --- | --- | --- | --- | --- |
| BA.5 | 292124 | male | 40 | Asymptomatic | BA.5.2 (Omicorn) | 2022-08-06 21:12 | 2022/8/6 | 2022-08-21 10:00 | 15 |
| BA.5 | 292181 | male | 23 | Mild | BA.5.2 (Omicorn) | 2022-08-07 12:56 | 2022/8/7 | 2022-08-27 10:00 | 20 |
| BA.5 | 292184 | male | 30 | Mild | BA.5.2 (Omicorn) | 2022-08-07 13:59 | 2022/8/7 | 2022-08- 15 10:22 | 8 |
| BA.5 | 292212 | female | 46 | Mild | BA.5.2 (Omicorn) | 2022-08-07 21:03 | 2022/8/6 | 2022-08-20 11:00 | 13 |
| BA.5 | 292222 | male | 59 | Mild | BA.5.2. 1 (Omicorn) | 2022-08-08 3:16 | 2022/8/7 | 2022-08- 16 9:43 | 8 |
| BA.5 | 292406 | female | 25 | Mild | BA.5.2. 1 (Omicorn) | 2022-08-09 20:47 | 2022/8/8 | 2022-08- 19 9:00 | 10 |
| BA.5 | 292419 | female | 53 | Asymptomatic | BA.5.2 (Omicorn) | 2022-08- 10 0:29 | 2022/8/9 | 2022-08-22 9:00 | 12 |
| BA.5 | 292421 | female | 21 | Mild | BA.5.2. 1 (Omicorn) | 2022-08- 10 2:10 | 2022/8/9 | 2022-08-25 15:00 | 16 |
| BA.5 | 292615 | male | 36 | Mild B | A.5.6 (Omicorn) (Omicorn) | 2022-08- 12 9:05 | 2022/8/ 11 | 2022-08-27 10:00 | 15 |
| BA.5 | 292633 | male | 37 | Mild | BA.5.2 (Omicorn) | 2022-08- 12 13:00 | 2022/8/ 11 | 2022-08-26 9:00 | 14 |
| BA.5 | 292653 | male | 30 | Mild | BA.5.2 (Omicorn) | 2022-08- 12 15:57 | 2022/8/ 11 | 2022-08-23 9:00 | 11 |
| BA.5 | 293347 | female | 21 | Moderate | BA.5. 1 (Omicorn) | 2022-08-20 10:07 | 2022/8/ 19 | 2022-08-30 9:00 | 10 |
| BA.5 | 293703 | male | 28 | Mild | BF. 15 (Omicorn) | 2022-08-23 8:48 | 2022/8/22 | 2022-09-05 10:00 | 13 |
| BA.5 | 293746 | female | 28 | Mild | BF. 15 (Omicorn) | 2022-08-23 11:38 | 2022/8/23 | 2022-09-08 10:00 | 16 |
| BA.5 | 293760 | female | 22 | Mild | BA.5.2. 1 (Omicorn) | 2022-08-23 16:19 | 2022/8/23 | 2022-09- 13 10:00 | 21 |
| BA.5 | 293777 | female | 26 | Mild | BA.5.2. 1 (Omicorn) | 2022-08-23 19:31 | 2022/8/22 | 2022-09-05 10:00 | 13 |
| BA.5 | 293780 | female | 25 | Asymptomatic | BA.5.2. 1 (Omicorn) | 2022-08-23 18:19 | 2022/8/22 | 2022-09-05 10:00 | 13 |
| BA.5 | 293795 | male | 26 | Mild | BA.5.2. 1 (Omicorn) | 2022-08-23 20:59 | 2022/8/23 | 2022-09-05 10:00 | 13 |
| BA.5 | 293868 | male | 29 | Asymptomatic | BA.5.2. 1 (Omicorn) | 2022-08-24 14:40 | 2022/8/23 | 2022-09-05 10:00 | 12 |
| BA.5 | 293920 | male | 46 | Mild | BA.5.2. 1 (Omicorn) | 2022-08-25 2:44 | 2022/8/24 | 2022-09-05 10:00 | 11 |
| BA.5 | 293961 | male | 9 | Mild | BA.5.2. 1 (Omicorn) | 2022-08-25 11:15 | 2022/8/24 | 2022-09-05 10:34 | 11 |
| BA.5 | 293986 | female | 42 | Mild | BA.5.2 (Omicorn) | 2022-08-25 16:24 | 2022/8/24 | 2022-09-08 10:00 | 14 |
| BA.5 | 293988 | female | 30 | Mild | BA.5.2 (Omicorn) | 2022-08-25 16:23 | 2022/8/24 | 2022-09-06 11:00 | 12 |
| BA.5 | 293996 | male | 34 | Asymptomatic | BA.5.2 (Omicorn) | 2022-08-25 17:58 | 2022/8/25 | 2022-09- 10 10:00 | 16 |
| BA.5 | 294005 | female | 35 | Asymptomatic | BA.5.2 (Omicorn) | 2022-08-25 20:57 | 2022/8/25 | 2022-09- 14 8:04 | 19 |
| BA.5 | 294019 | female | 39 | Mild | BA.5.2 (Omicorn) | 2022-08-26 7:15 | 2022/8/26 | 2022-09- 10 10:00 | 15 |

| BA.5 | 294052 | female | 58 | Mild | BA.5.2 (Omicorn) | 2022-08-26 12:14 | 2022/8/25 | 2022-09- 14 8:04 | 19 |
| --- | --- | --- | --- | --- | --- | --- | --- | --- | --- |
| BA.5 | 294076 | male | 31 | Mild | BF. 15 (Omicorn) | 2022-08-26 17:00 | 2022/8/26 | 2022-09-05 10:00 | 10 |
| BA.5 | 294089 | male | 29 | Mild | BF. 15 (Omicorn) | 2022-08-26 23:09 | 2022/8/26 | 2022-09-09 8:12 | 13 |
| BA.5 | 294090 | female | 29 | Mild | BF. 15 (Omicorn) | 2022-08-26 23:07 | 2022/8/26 | 2022-09-05 10:00 | 9 |
| BA.5 | 294091 | male | 29 | Asymptomatic | BF. 15 (Omicorn) | 2022-08-26 23:08 | 2022/8/26 | 2022-09-08 10:00 | 12 |
| BA.5 | 294099 | male | 60 | Mild | BA.5.2. 1 (Omicorn) | 2022-08-27 0:34 | 2022/8/26 | 2022-09- 10 10:00 | 14 |
| BA.5 | 294100 | male | 29 | Mild | BF. 15 (Omicorn) | 2022-08-27 0:35 | 2022/8/27 | 2022-09-09 10:00 | 13 |
| BA.5 | 294101 | female | 25 | Moderate | BF. 15 (Omicorn) | 2022-08-27 0:35 | 2022/8/26 | 2022-09-09 10:00 | 13 |
| BA.5 | 294115 | male | 49 | Mild | BA.5.2 (Omicorn) | 2022-08-27 8:09 | 2022/8/27 | 2022-09- 10 10:00 | 14 |
| BA.5 | 294137 | male | 20 | Mild | BA.5.2 (Omicorn) | 2022-08-27 11:40 | 2022/8/26 | 2022-09- 10 10:00 | 14 |
| BA.5 | 294141 | male | 27 | Asymptomatic | BF. 15 (Omicorn) | 2022-08-27 13:01 | 2022/8/27 | 2022-09-08 11:30 | 12 |
| BA.5 | 294167 | male | 27 | Mild | BA.5.2 (Omicorn) | 2022-08-27 17:22 | 2022/8/27 | 2022-09- 10 9:00 | 14 |
| BA.5 | 294186 | male | 31 | Mild | BF. 15 (Omicorn) | 2022-08-27 22:08 | 2022/8/26 | 2022-09-08 11:30 | 12 |
| BA.5 | 294199 | male | 50 | Mild | BF. 15 (Omicorn) | 2022-08-28 9:23 | 2022/8/27 | 2022-09- 11 9:00 | 14 |
| BA.5 | 294200 | male | 41 | Mild | BF. 15 (Omicorn) | 2022-08-28 8:22 | 2022/8/27 | 2022/9/ 13 | 16 |
| BA.5 | 294203 | female | 25 | Mild | BF. 15 (Omicorn) | 2022-08-28 9:23 | 2022/8/27 | 2022/9/ 13 | 16 |
| BA.5 | 294204 | male | 49 | Mild | BA.5.2 (Omicorn) | 2022-08-28 10:05 | 2022/8/27 | 2022-09- 11 9:00 | 14 |
| BA.5 | 294214 | female | 77 | Mild | BA.5.2 (Omicorn) | 2022-08-28 12:14 | 2022/8/27 | 2022-09- 16 11:00 | 19 |
| BA.5 | 294244 | male | 50 | Asymptomatic | BA.5.2 (Omicorn) | 2022-08-28 12:03 | 2022/8/27 | 2022-09- 14 8:01 | 17 |
| BA.5 | 294249 | female | 9 | Mild | BF. 15 (Omicorn) | 2022-08-28 14:35 | 2022/8/27 | 2022-09- 12 9:23 | 15 |
| BA.5 | 294250 | female | 54 | Mild | BA.5.2 (Omicorn) | 2022-08-28 14:36 | 2022/8/27 | 2022-09- 14 8:02 | 17 |
| BA.5 | 294251 | male | 13 | Mild | BA.5.2 (Omicorn) | 2022-08-28 14:37 | 2022/8/27 | 2022-09- 10 10:00 | 13 |
| BA.5 | 294295 | female | 64 | Moderate | BA.5.2 (Omicorn) | 2022-08-29 3:11 | 2022/8/28 | 2022-09- 14 10:00 | 16 |
| BA.5 | 294296 | male | 36 | Asymptomatic | BF. 15 (Omicorn) | 2022-08-29 4:36 | 2022/8/28 | 2022-09- 16 11:54 | 18 |
| BA.5 | 294297 | female | 1 | Mild | BF. 15 (Omicorn) | 2022-08-29 4:37 | 2022/8/28 | 2022-09-06 15:00 | 8 |
| BA.5 | 294298 | male | 43 | Moderate | BA.5.2. 1 (Omicorn) | 2022-08-29 4:15 | 2022/8/29 | 2022-09- 12 9:22 | 14 |
| BA.5 | 294299 | female | 36 | Mild | BA.5.2. 1 (Omicorn) | 2022-08-29 4:15 | 2022/8/29 | 2022-09- 10 10:00 | 12 |

| BA.5 | 294301 | female | 32 | Asymptomatic | BF. 15 (Omicorn) | 2022-08-29 4:33 | 2022/8/28 | 2022-09- 13 8:29 | 15 |
| --- | --- | --- | --- | --- | --- | --- | --- | --- | --- |
| BA.5 | 294302 | female | 63 | Asymptomatic | BA.5.2 (Omicorn) | 2022-08-29 4:34 | 2022/8/28 | 2022/9/ 13 | 15 |
| BA.5 | 294303 | male | 14 | Mild | BA.5.2 (Omicorn) | 2022-08-29 4:35 | 2022/8/28 | 2022-09- 10 9:00 | 12 |
| BA.5 | 294304 | female | 32 | Mild | BA.5.2 (Omicorn) | 2022-08-29 4:47 | 2022/8/28 | 2022/9/ 13 | 15 |
| BA.5 | 294305 | male | 43 | Mild | BA.5.2 (Omicorn) | 2022-08-29 5:10 | 2022/8/28 | 2022-09-08 11:30 | 10 |
| BA.5 | 294312 | male | 40 | Mild | BA.5.2 (Omicorn) | 2022-08-29 7:59 | 2022/8/28 | 2022-09- 11 9:00 | 13 |
| BA.5 | 294319 | male | 45 | Mild | BA.5.2 (Omicorn) | 2022-08-29 9:05 | 2022/8/28 | 2022-09- 15 8:56 | 17 |
| BA.5 | 294322 | male | 36 | Mild | BA.5.2 (Omicorn) | 2022-08-29 9:12 | 2022/8/28 | 2022-09-06 15:00 | 8 |
| BA.5 | 294323 | female | 36 | Asymptomatic | BA.5.2 (Omicorn) | 2022-08-29 9:23 | 2022/8/28 | 2022-09- 11 9:00 | 13 |
| BA.5 | 294332 | female | 26 | Mild | BA.5.2 (Omicorn) | 2022-08-29 9:58 | 2022/8/28 | 2022-09- 16 10:00 | 18 |
| BA.5 | 294334 | male | 13 | Mild | BA.5.2 (Omicorn) | 2022-08-29 11:06 | 2022/8/28 | 2022-09- 10 10:00 | 12 |
| BA.5 | 294335 | female | 55 | Mild | BA.5.2 (Omicorn) | 2022-08-29 11:05 | 2022/8/28 | 2022-09- 11 9:00 | 13 |
| BA.5 | 294339 | female | 56 | Mild | BA.5.2 (Omicorn) | 2022-08-29 10:38 | 2022/8/28 | 2022-09- 15 11:45 | 17 |
| BA.5 | 294340 | male | 51 | Asymptomatic B | A.5.6 (Omicorn) (Omicorn) | 2022-08-29 13:34 | 2022/8/29 | 2022/9/ 13 | 14 |
| BA.5 | 294347 | female | 31 | Asymptomatic | BA.5.2 (Omicorn) | 2022-08-29 15:40 | 2022/8/29 | 2022-09-20 10:00 | 22 |
| BA.5 | 294368 | female | 28 | Asymptomatic | BF. 15 (Omicorn) | 2022-08-29 15:42 | 2022/8/28 | 2022-09- 10 10:00 | 12 |
| BA.5 | 294369 | female | 39 | Mild | BA.5.2 (Omicorn) | 2022-08-29 15:41 | 2022/8/28 | 2022/9/ 13 | 14 |
| BA.5 | 294378 | female | 40 | Mild | BA.5.2 (Omicorn) | 2022-08-29 17:59 | 2022/8/28 | 2022-09-09 14:00 | 11 |
| BA.5 | 294383 | female | 23 | Mild | BA.5.2 (Omicorn) | 2022-08-29 17:28 | 2022/8/28 | 2022-09-09 10:30 | 11 |
| BA.5 | 294384 | male | 64 | Asymptomatic | BA.5.2 (Omicorn) | 2022-08-29 18:00 | 2022/8/29 | 2022-09- 12 10:00 | 14 |
| BA.5 | 294385 | female | 50 | Asymptomatic | BA.5.2 (Omicorn) | 2022-08-29 17:58 | 2022/8/29 | 2022/9/ 13 | 14 |
| BA.5 | 294386 | male | 24 | Asymptomatic | BA.5.2 (Omicorn) | 2022-08-29 17:59 | 2022/8/28 | 2022-09- 10 10:00 | 12 |
| BA.5 | 294390 | male | 30 | Mild | BF. 15 (Omicorn) | 2022-08-29 20:25 | 2022/8/28 | 2022-09-08 11:00 | 10 |
| BA.5 | 294392 | male | 40 | Mild | BA.5.2 (Omicorn) | 2022-08-29 19:43 | 2022/8/29 | 2022/9/ 13 | 14 |
| BA.5 | 294393 | male | 35 | Mild | BA.5.2 (Omicorn) | 2022-08-29 19:44 | 2022/8/29 | 2022-09- 15 11:00 | 17 |
| BA.5 | 294397 | male | 40 | Asymptomatic | BA.5.2 (Omicorn) | 2022-08-29 18:14 | 2022/8/29 | 2022-09- 15 10:56 | 17 |
| BA.5 | 294398 | female | 41 | Mild | BA.5.2 (Omicorn) | 2022-08-29 17:26 | 2022/8/29 | 2022-09- 11 9:00 | 13 |

| BA.5 | 294406 | female | 23 | Mild | BA.5.2 (Omicorn) | 2022-08-29 16:54 | 2022/8/29 | 2022-09-08 10:00 | 10 |
| --- | --- | --- | --- | --- | --- | --- | --- | --- | --- |
| BA.5 | 294408 | female | 15 | Mild | BA.5.2 (Omicorn) | 2022-08-29 21:33 | 2022/8/28 | 2022-09- 12 10:00 | 14 |
| BA.5 | 294409 | female | 32 | Asymptomatic | BA.5.2 (Omicorn) | 2022-08-29 20:16 | 2022/8/28 | 2022-09- 14 9:00 | 16 |
| BA.5 | 294410 | male | 65 | Asymptomatic | BA.5.2 (Omicorn) | 2022-08-29 21:32 | 2022/8/28 | 2022-09- 17 10:00 | 19 |
| BA.5 | 294411 | female | 60 | Asymptomatic | BA.5.2 (Omicorn) | 2022-08-29 21:32 | 2022/8/28 | 2022-09- 14 9:30 | 15 |
| BA.5 | 294420 | male | 19 | Asymptomatic | BA.5.2 (Omicorn) | 2022-08-29 22:06 | 2022/8/29 | 2022-09-06 10:00 | 7 |
| BA.5 | 294421 | male | 51 | Mild | BA.5.2 (Omicorn) | 2022-08-29 22:07 | 2022/8/28 | 2022-09- 10 10:00 | 11 |
| BA.5 | 294424 | female | 57 | Mild | BA.5.2 (Omicorn) | 2022-08-29 22:54 | 2022/8/28 | 2022-09- 18 10:00 | 19 |
| BA.5 | 294425 | female | 15 | Mild | BA.5.2 (Omicorn) | 2022-08-29 23:04 | 2022/8/28 | 2022-09-09 15:00 | 11 |
| BA.5 | 294427 | female | 30 | Asymptomatic | BA.5.2 (Omicorn) | 2022-08-29 23:05 | 2022/8/28 | 2022-09- 11 10:00 | 12 |
| BA.5 | 294431 | female | 55 | Mild | BA.5.2 (Omicorn) | 2022-08-29 23:05 | 2022/8/28 | 2022-09- 10 10:00 | 11 |
| BA.5 | 294441 | female | 29 | Asymptomatic | BA.5.2 (Omicorn) | 2022-08-30 2:21 | 2022/8/29 | 2022-09- 12 9:00 | 13 |
| BA.5 | 294442 | male | 43 | Mild | BA.5.2. 1 (Omicorn) | 2022-08-30 2:22 | 2022/8/29 | 2022-09- 15 10:00 | 16 |
| BA.5 | 294443 | male | 51 | Asymptomatic | BA.5.2 (Omicorn) | 2022-08-30 3:18 | 2022/8/29 | 2022-09- 11 10:00 | 12 |
| BA.5 | 294444 | female | 24 | Mild B | A.5.6 (Omicorn) (Omicorn) | 2022-08-30 3:18 | 2022/8/28 | 2022-09- 10 10:00 | 11 |
| BA.5 | 294445 | male | 3 | Mild | BA.5.2. 1 (Omicorn) | 2022-08-30 3:22 | 2022/8/29 | 2022-09- 11 10:00 | 12 |
| BA.5 | 294447 | male | 18 | Mild | BF. 15 (Omicorn) | 2022-08-30 4:21 | 2022/8/30 | 2022-09- 10 10:00 | 11 |
| BA.5 | 294449 | male | 46 | Asymptomatic | BA.5.2 (Omicorn) | 2022-08-30 6:22 | 2022/8/29 | 2022-09- 10 10:00 | 11 |
| BA.5 | 294450 | male | 27 | Mild | BA.5.2 (Omicorn) | 2022-08-30 7:37 | 2022/8/30 | 2022-09- 14 9:00 | 15 |
| BA.5 | 294474 | male | 34 | Mild | BA.5.2 (Omicorn) | 2022-08-30 12:19 | 2022/8/29 | 2022-09-07 10:00 | 8 |
| BA.5 | 294476 | female | 28 | Mild | BA.5.2 (Omicorn) | 2022-08-30 9:57 | 2022/8/29 | 2022-09-08 9:00 | 9 |
| BA.5 | 294485 | female | 78 | Asymptomatic | BA.5.2 (Omicorn) | 2022-08-30 12:15 | 2022/8/30 | 2022-09- 14 9:30 | 15 |
| BA.5 | 294492 | female | 32 | Mild B | A.5.6 (Omicorn) (Omicorn) | 2022-08-30 14:10 | 2022/8/29 | 2022-09- 11 10:00 | 12 |
| BA.5 | 294495 | female | 53 | Asymptomatic | BF. 15 (Omicorn) | 2022-08-30 14:12 | 2022/8/29 | 2022-09- 13 10:00 | 14 |
| BA.5 | 294497 | male | 33 | Asymptomatic | BA.5.2 (Omicorn) | 2022-08-30 14:09 | 2022/8/30 | 2022-09-08 8:55 | 9 |
| BA.5 | 294506 | male | 46 | Moderate | BA.5.2 (Omicorn) | 2022-08-30 14:10 | 2022/8/29 | 2022-09- 10 10:00 | 11 |
| BA.5 | 294510 | male | 20 | Mild | BA.5.2 (Omicorn) | 2022-08-30 14:11 | 2022/8/30 | 2022-09- 13 10:00 | 14 |

| BA.5 | 294519 | male | 70 | Asymptomatic | BA.5.2 (Omicorn) | 2022-08-30 17:05 | 2022/8/30 | 2022-09- 10 10:00 | 11 |
| --- | --- | --- | --- | --- | --- | --- | --- | --- | --- |
| BA.5 | 294526 | male | 63 | Asymptomatic | BA.5.2 (Omicorn) | 2022-08-30 17:51 | 2022/8/30 | 2022-09- 14 9:00 | 15 |
| BA.5 | 294527 | male | 50 | Asymptomatic | BA.5.2 (Omicorn) | 2022-08-30 17:52 | 2022/8/30 | 2022-09- 17 10:00 | 18 |
| BA.5 | 294528 | female | 41 | Mild | BA.5.2 (Omicorn) | 2022-08-30 17:09 | 2022/8/30 | 2022-09- 17 8:25 | 18 |
| BA.5 | 294534 | female | 24 | Mild | BA.5.2 (Omicorn) | 2022-08-30 18:02 | 2022/8/29 | 2022-09-09 14:00 | 10 |
| BA.5 | 294535 | male | 24 | Asymptomatic | BA.5.2 (Omicorn) | 2022-08-30 18:13 | 2022/8/30 | 2022-09- 12 10:00 | 13 |
| BA.5 | 294537 | female | 52 | Asymptomatic | BA.5.2 (Omicorn) | 2022-08-30 18:16 | 2022/8/30 | 2022-09- 14 9:30 | 15 |
| BA.5 | 294540 | male | 12 | Asymptomatic | BA.5.2 (Omicorn) | 2022-08-30 18:01 | 2022/8/30 | 2022-09- 11 10:00 | 12 |
| BA.5 | 294546 | male | 4 | Mild | BA.5.2 (Omicorn) | 2022-08-30 19:28 | 2022/8/29 | 2022-09-08 10:00 | 9 |
| BA.5 | 294548 | female | 45 | Mild | BA.5.2 (Omicorn) | 2022-08-30 21:23 | 2022/8/30 | 2022-09- 14 9:00 | 14 |
| BA.5 | 294554 | male | 60 | Mild | BA.5.2 (Omicorn) | 2022-08-30 23:09 | 2022/8/30 | 2022-09- 12 10:00 | 12 |
| BA.5 | 294558 | female | 42 | Asymptomatic | BA.5.2 (Omicorn) | 2022-08-31 0:50 | 2022/8/30 | 2022-09- 14 9:30 | 14 |
| BA.5 | 294559 | female | 59 | Mild | BA.5.2 (Omicorn) | 2022-08-31 1:29 | 2022/8/30 | 2022-09- 16 10:00 | 16 |
| BA.5 | 294562 | male | 59 | Asymptomatic | BA.5.2 (Omicorn) | 2022-08-31 1:08 | 2022/8/30 | 2022-09- 16 10:00 | 16 |
| BA.5 | 294563 | male | 57 | Asymptomatic | BA.5.2 (Omicorn) | 2022-08-31 1:32 | 2022/8/30 | 2022-09- 12 10:00 | 12 |
| BA.5 | 294565 | female | 49 | Asymptomatic | BA.5.2 (Omicorn) | 2022-08-31 1:30 | 2022/8/30 | 2022-09- 16 10:00 | 16 |
| BA.5 | 294566 | female | 46 | Mild | BA.5.2 (Omicorn) | 2022-08-31 2:08 | 2022/8/30 | 2022-09- 12 10:00 | 12 |
| BA.5 | 294575 | female | 50 | Mild | BA.5.2 (Omicorn) | 2022-08-31 3:09 | 2022/8/30 | 2022-09- 10 10:00 | 10 |
| BA.5 | 294577 | female | 30 | Mild | BA.5.2 (Omicorn) | 2022-08-31 3:22 | 2022/8/30 | 2022-09- 11 10:00 | 11 |
| BA.5 | 294578 | female | 37 | Mild | BA.5.2 (Omicorn) | 2022-08-31 3:32 | 2022/8/31 | 2022-09- 14 9:00 | 14 |
| BA.5 | 294579 | male | 10 | Asymptomatic | BA.5.2 (Omicorn) | 2022-08-31 3:33 | 2022/8/30 | 2022-09- 14 9:00 | 14 |
| BA.5 | 294580 | female | 49 | Mild | BA.5.2. 1 (Omicorn) | 2022-08-31 6:45 | 2022/8/30 | 2022-09- 15 10:00 | 15 |
| BA.5 | 294581 | male | 33 | Asymptomatic | BA.5.2. 1 (Omicorn) | 2022-08-31 6:45 | 2022/8/30 | 2022-09- 16 10:00 | 16 |
| BA.5 | 294582 | male | 22 | Asymptomatic | BA.5.2 (Omicorn) | 2022-08-31 6:45 | 2022/8/30 | 2022-09- 17 10:00 | 17 |
| BA.5 | 294584 | male | 27 | Mild | BA.5.2 (Omicorn) | 2022-08-31 9:43 | 2022/8/30 | 2022-09- 18 10:00 | 18 |
| BA.5 | 294585 | male | 13 | Mild | BA.5.2 (Omicorn) | 2022-08-31 7:04 | 2022/8/30 | 2022-09- 17 10:00 | 17 |
| BA.5 | 294587 | male | 28 | Asymptomatic | BA.5.2 (Omicorn) | 2022-08-31 6:46 | 2022/8/30 | 2022-09- 10 10:00 | 10 |

| BA.5 | 294589 | female | 47 | Moderate | BA.5.2 (Omicorn) | 2022-08-31 7:10 | 2022/8/30 | 2022-09- 15 10:00 | 15 |
| --- | --- | --- | --- | --- | --- | --- | --- | --- | --- |
| BA.5 | 294590 | female | 31 | Moderate | BA.5.2 (Omicorn) | 2022-08-31 8:43 | 2022/8/30 | 2022-09- 12 10:00 | 12 |
| BA.5 | 294593 | male | 45 | Asymptomatic | BA.5.2 (Omicorn) | 2022-08-31 9:58 | 2022/8/31 | 2022-09- 17 10:00 | 17 |
| BA.5 | 294594 | male | 52 | Asymptomatic | BA.5.2 (Omicorn) | 2022-08-31 9:24 | 2022/8/31 | 2022-09- 13 10:00 | 13 |
| BA.5 | 294596 | female | 43 | Mild | BA.5.2 (Omicorn) | 2022-08-31 9:24 | 2022/8/30 | 2022-09- 16 10:00 | 16 |
| BA.5 | 294597 | female | 58 | Asymptomatic | BA.5.2 (Omicorn) | 2022-08-31 10:12 | 2022/8/30 | 2022-09- 11 10:00 | 11 |
| BA.5 | 294604 | male | 42 | Asymptomatic | BA.5.2 (Omicorn) | 2022-08-31 11:24 | 2022/8/30 | 2022-09- 17 10:00 | 17 |
| BA.5 | 294605 | male | 12 | Mild | BA.5.2 (Omicorn) | 2022-08-31 11:23 | 2022/8/30 | 2022-09- 15 10:00 | 15 |
| BA.5 | 294619 | male | 35 | Asymptomatic | BA.5.2 (Omicorn) | 2022-08-31 12:36 | 2022/8/30 | 2022-09- 11 10:00 | 11 |
| BA.5 | 294621 | male | 34 | Asymptomatic | BA.5.2 (Omicorn) | 2022-08-31 12:34 | 2022/8/30 | 2022-09-07 10:00 | 7 |
| BA.5 | 294622 | male | 51 | Asymptomatic | BA.5.2 (Omicorn) | 2022-08-31 11:51 | 2022/8/30 | 2022-09- 11 10:00 | 11 |
| BA.5 | 294623 | male | 46 | Asymptomatic | BA.5.2 (Omicorn) | 2022-08-31 11:52 | 2022/8/30 | 2022-09- 12 10:00 | 12 |
| BA.5 | 294624 | male | 54 | Mild | BA.5.2 (Omicorn) | 2022-08-31 11:51 | 2022/8/30 | 2022-09- 17 10:00 | 17 |
| BA.5 | 294625 | male | 48 | Mild | BA.5.2 (Omicorn) | 2022-08-31 12:36 | 2022/8/30 | 2022-09- 18 10:00 | 18 |
| BA.5 | 294626 | female | 29 | Asymptomatic | BA.5.2 (Omicorn) | 2022-08-31 12:02 | 2022/8/30 | 2022-09- 14 9:12 | 14 |
| BA.5 | 294627 | male | 71 | Moderate | BA.5.2 (Omicorn) | 2022-08-31 12:36 | 2022/8/30 | 2022-09- 11 10:00 | 11 |
| BA.5 | 294641 | male | 27 | Mild | BA.5.2 (Omicorn) | 2022-08-31 13:09 | 2022/8/30 | 2022-09- 10 10:00 | 10 |
| BA.5 | 294651 | male | 56 | Asymptomatic | BA.5.2 (Omicorn) | 2022-08-31 15:05 | 2022/8/30 | 2022-09- 19 9:30 | 19 |
| BA.5 | 294652 | male | 39 | Mild | BA.5.2 (Omicorn) | 2022-08-31 15:48 | 2022/8/31 | 2022-09- 11 10:00 | 11 |
| BA.5 | 294653 | male | 51 | Mild | BA.5.2 (Omicorn) | 2022-08-31 15:50 | 2022/8/30 | 2022-09- 17 9:00 | 17 |
| BA.5 | 294657 | female | 36 | Mild | BA.5.2 (Omicorn) | 2022-08-31 16:23 | 2022/8/30 | 2022-09- 10 10:00 | 10 |
| BA.5 | 294664 | female | 30 | Mild | BA.5.2 (Omicorn) | 2022-08-31 17:09 | 2022/8/31 | 2022-09- 13 10:00 | 13 |
| BA.5 | 294665 | female | 38 | Mild | BA.5.2 (Omicorn) | 2022-08-31 16:35 | 2022/8/31 | 2022-09- 15 11:49 | 15 |
| BA.5 | 294669 | male | 31 | Mild | BA.5.2 (Omicorn) | 2022-08-31 17:09 | 2022/8/31 | 2022-09- 14 8:15 | 14 |
| BA.5 | 294674 | female | 9 | Mild | BA.5.2 (Omicorn) | 2022-08-31 18:02 | 2022/8/30 | 2022-09- 11 10:00 | 11 |
| BA.5 | 294677 | male | 19 | Asymptomatic | BA.5.2 (Omicorn) | 2022-08-31 17:12 | 2022/8/31 | 2022-09-09 10:00 | 9 |
| BA.5 | 294678 | male | 46 | Mild | BA.5.2 (Omicorn) | 2022-08-31 17:14 | 2022/8/30 | 2022-09- 11 8:08 | 11 |

| BA.5 | 294681 | female | 56 | Mild | BA.5.2 (Omicorn) | 2022-08-31 17:08 | 2022/8/30 | 2022-09- 15 10:00 | 15 |
| --- | --- | --- | --- | --- | --- | --- | --- | --- | --- |
| BA.5 | 294682 | male | 48 | Mild | BA.5.2 (Omicorn) | 2022-08-31 18:53 | 2022/8/31 | 2022-09- 10 9:00 | 10 |
| BA.5 | 294684 | male | 58 | Mild | BA.5.2 (Omicorn) | 2022-08-31 17:38 | 2022/8/31 | 2022-09- 15 10:00 | 15 |
| BA.5 | 294685 | female | 36 | Asymptomatic | BA.5.2 (Omicorn) | 2022-08-31 17:38 | 2022/8/31 | 2022-09- 17 10:00 | 17 |
| BA.5 | 294690 | male | 33 | Moderate | BA.5.2 (Omicorn) | 2022-08-31 20:29 | 2022/8/31 | 2022-09- 17 8:15 | 16 |
| BA.5 | 294691 | female | 41 | Asymptomatic | BA.5.2 (Omicorn) | 2022-08-31 23:41 | 2022/8/30 | 2022-09- 16 8:43 | 15 |
| BA.5 | 294696 | female | 46 | Mild | BA.5.2 (Omicorn) | 2022-08-31 20:57 | 2022/8/31 | 2022-09- 16 8:36 | 15 |
| BA.5 | 294700 | male | 15 | Asymptomatic | BA.5.2 (Omicorn) | 2022-08-31 23:09 | 2022/8/31 | 2022-09- 16 10:00 | 15 |
| BA.5 | 294703 | female | 46 | Mild | BA.5.2 (Omicorn) | 2022-08-31 23:11 | 2022/8/31 | 2022-09- 16 8:40 | 15 |
| BA.5 | 294719 | female | 31 | Asymptomatic | BA.5.2 (Omicorn) | 2022-09-01 11:56 | 2022/8/31 | 2022-09- 18 10:00 | 17 |
| BA.5 | 294726 | female | 50 | Mild | BA.5.2 (Omicorn) | 2022-09-01 4:47 | 2022/9/ 1 | 2022-09-23 0:00 | 22 |
| BA.5 | 294727 | male | 37 | Mild | BA.5.2 (Omicorn) | 2022-09-01 3:45 | 2022/8/31 | 2022-09- 14 8:05 | 13 |
| BA.5 | 294728 | female | 40 | Asymptomatic | BA.5.2 (Omicorn) | 2022-09-01 3:47 | 2022/8/31 | 2022-09- 18 11:00 | 17 |
| BA.5 | 294730 | female | 53 | Asymptomatic | BA.5.2 (Omicorn) | 2022-09-01 3:47 | 2022/9/ 1 | 2022-09- 13 10:00 | 12 |
| BA.5 | 294734 | male | 47 | Mild | BA.5.2 (Omicorn) | 2022-09-01 4:55 | 2022/9/ 1 | 2022-09- 18 11:00 | 17 |
| BA.5 | 294736 | male | 40 | Asymptomatic | BA.5.2 (Omicorn) | 2022-09-01 3:54 | 2022/9/ 1 | 2022-09- 16 8:40 | 15 |
| BA.5 | 294738 | male | 17 | Mild | BA.5.2 (Omicorn) | 2022-09-01 4:20 | 2022/9/ 1 | 2022-09- 15 9:25 | 14 |
| BA.5 | 294739 | female | 57 | Mild | BA.5.2. 1 (Omicorn) | 2022-09-01 4:25 | 2022/8/31 | 2022-09- 18 11:00 | 17 |
| BA.5 | 294740 | female | 23 | Mild | BA.5.2. 1 (Omicorn) | 2022-09-01 4:28 | 2022/9/ 1 | 2022-09- 13 10:00 | 12 |
| BA.5 | 294741 | female | 59 | Asymptomatic | BA.5.2 (Omicorn) | 2022-09-01 6:11 | 2022/8/31 | 2022-09- 15 10:01 | 14 |
| BA.5 | 294742 | male | 52 | Mild | BA.5.2 (Omicorn) | 2022-09-01 7:26 | 2022/8/31 | 2022-09- 18 11:00 | 17 |
| BA.5 | 294743 | female | 47 | Asymptomatic | BA.5.2 (Omicorn) | 2022-09-01 5:02 | 2022/9/ 1 | 2022-09- 15 10:00 | 14 |
| BA.5 | 294744 | female | 41 | Asymptomatic | BA.5.2 (Omicorn) | 2022-09-01 7:25 | 2022/9/ 1 | 2022-09- 12 9:37 | 11 |
| BA.5 | 294746 | female | 36 | Asymptomatic | BA.5.2 (Omicorn) | 2022-09-01 9:36 | 2022/8/31 | 2022-09- 16 8:42 | 15 |
| BA.5 | 294747 | female | 67 | Asymptomatic | BA.5.2 (Omicorn) | 2022-09-01 9:37 | 2022/8/31 | 2022-09- 12 10:15 | 11 |
| BA.5 | 294749 | male | 46 | Mild | BA.5.2 (Omicorn) | 2022-09-01 8:43 | 2022/8/31 | 2022-09-20 10:00 | 19 |
| BA.5 | 294752 | female | 54 | Asymptomatic | BA.5.2 (Omicorn) | 2022-09-01 8:44 | 2022/8/31 | 2022-09-20 10:00 | 19 |

| BA.5 | 294758 | male | 35 | Mild | BA.5.2 (Omicorn) | 2022-09-01 10:28 | 2022/8/31 | 2022-09- 11 10:00 | 10 |
| --- | --- | --- | --- | --- | --- | --- | --- | --- | --- |
| BA.5 | 294759 | female | 24 | Mild | BA.5.2 (Omicorn) | 2022-09-01 10:29 | 2022/9/ 1 | 2022-09- 15 10:00 | 14 |
| BA.5 | 294761 | male | 12 | Mild | BA.5.2 (Omicorn) | 2022-09-01 10:29 | 2022/9/ 1 | 2022-09- 15 10:00 | 14 |
| BA.5 | 294767 | male | 60 | Asymptomatic | BA.5.2 (Omicorn) | 2022-09-01 10:30 | 2022/8/31 | 2022-09- 18 11:00 | 17 |
| BA.5 | 294771 | female | 55 | Mild | BA.5.2 (Omicorn) | 2022-09-01 10:50 | 2022/8/31 | 2022-09- 13 10:00 | 12 |
| BA.5 | 294777 | male | 64 | Mild | BA.5.2 (Omicorn) | 2022-09-01 14:23 | 2022/8/31 | 2022-09- 18 10:00 | 17 |
| BA.5 | 294778 | female | 56 | Mild | BA.5.2 (Omicorn) | 2022-09-01 14:20 | 2022/9/ 1 | 2022-09- 15 11:45 | 14 |
| BA.5 | 294783 | male | 44 | Mild | BA.5.2 (Omicorn) | 2022-09-01 14:05 | 2022/9/ 1 | 2022-09- 15 11:47 | 14 |
| BA.5 | 294792 | female | 44 | Mild | BA.5.2 (Omicorn) | 2022-09-01 11:57 | 2022/9/ 1 | 2022-09- 15 10:00 | 14 |
| BA.5 | 294794 | female | 44 | Moderate | BA.5.2 (Omicorn) | 2022-09-01 14:32 | 2022/9/ 1 | 2022-09- 12 10:00 | 11 |
| BA.5 | 294795 | female | 57 | Asymptomatic | BA.5.2 (Omicorn) | 2022-09-01 14:30 | 2022/9/ 1 | 2022-09- 13 10:00 | 12 |
| BA.5 | 294796 | male | 41 | Mild | BA.5.2 (Omicorn) | 2022-09-01 11:00 | 2022/8/31 | 2022-09- 10 9:00 | 9 |
| BA.5 | 294803 | female | 41 | Mild | BA.5.2 (Omicorn) | 2022-09-01 15:05 | 2022/9/ 1 | 2022-09- 11 8:04 | 10 |
| BA.5 | 294806 | male | 6 | Asymptomatic | BA.5.2 (Omicorn) | 2022-09-01 15:02 | 2022/8/31 | 2022-09- 15 11:49 | 14 |
| BA.5 | 294822 | female | 56 | Mild | BA.5.2 (Omicorn) | 2022-09-01 14:54 | 2022/9/ 1 | 2022-09- 18 10:00 | 17 |
| BA.5 | 294825 | male | 31 | Mild | BA.5.2 (Omicorn) | 2022-09-01 13:27 | 2022/8/31 | 2022-09- 11 10:00 | 10 |
| BA.5 | 294826 | female | 50 | Asymptomatic | BA.5.2 (Omicorn) | 2022-09-01 14:56 | 2022/8/31 | 2022-09- 14 17:00 | 13 |
| BA.5 | 294831 | male | 80 | Asymptomatic | BA.5.2 (Omicorn) | 2022-09-01 14:32 | 2022/9/ 1 | 2022-09- 14 10:20 | 13 |
| BA.5 | 294838 | male | 68 | Moderate | BA.5.2 (Omicorn) | 2022-09-01 15:08 | 2022/8/31 | 2022-09-22 10:00 | 21 |
| BA.5 | 294839 | male | 54 | Asymptomatic | BA.5.2 (Omicorn) | 2022-09-01 15:52 | 2022/8/31 | 2022-09-08 9:00 | 7 |
| BA.5 | 294841 | male | 1 | Asymptomatic | BA.5.2 (Omicorn) | 2022-09-01 15:10 | 2022/8/31 | 2022-09- 14 14:32 | 13 |
| BA.5 | 294842 | male | 5 | Mild | BA.5.2 (Omicorn) | 2022-09-01 15:06 | 2022/9/ 1 | 2022-09- 14 9:50 | 13 |
| BA.5 | 294845 | female | 29 | Asymptomatic | BA.5.2 (Omicorn) | 2022-09-01 15:45 | 2022/9/ 1 | 2022-09- 13 10:00 | 12 |
| BA.5 | 294846 | male | 27 | Mild | BA.5.2 (Omicorn) | 2022-09-01 15:45 | 2022/8/31 | 2022-09-08 10:00 | 7 |
| BA.5 | 294847 | male | 44 | Mild | BA.5.2 (Omicorn) | 2022-09-01 17:52 | 2022/8/31 | 2022-09- 12 9:47 | 11 |
| BA.5 | 294848 | female | 45 | Asymptomatic | BA.5.2 (Omicorn) | 2022-09-01 17:01 | 2022/8/31 | 2022-09-24 0:00 | 22 |
| BA.5 | 294849 | female | 25 | Mild | BA.5.2 (Omicorn) | 2022-09-01 16:06 | 2022/8/31 | 2022-09- 15 16:15 | 14 |

| BA.5 | 294850 | female | 57 | Mild | BA.5.2 (Omicorn) | 2022-09-01 16:31 | 2022/8/31 | 2022-09- 16 10:00 | 15 |
| --- | --- | --- | --- | --- | --- | --- | --- | --- | --- |
| BA.5 | 294852 | male | 25 | Mild | BA.5.2 (Omicorn) | 2022-09-01 16:33 | 2022/9/ 1 | 2022-09- 13 8:40 | 12 |
| BA.5 | 294868 | female | 34 | Asymptomatic | BA.5.2 (Omicorn) | 2022-09-01 16:58 | 2022/9/ 1 | 2022-09- 15 11:46 | 14 |
| BA.5 | 294875 | male | 34 | Asymptomatic | BA.5.2 (Omicorn) | 2022-09-01 21:04 | 2022/8/31 | 2022-09- 15 11:43 | 14 |
| BA.5 | 294876 | male | 23 | Mild | BA.5.2 (Omicorn) | 2022-09-01 21:03 | 2022/9/ 1 | 2022-09- 14 10:00 | 13 |
| BA.5 | 294880 | male | 55 | Asymptomatic | BA.5.2 (Omicorn) | 2022-09-01 18:06 | 2022/9/ 1 | 2022-09- 15 10:00 | 14 |
| BA.5 | 294882 | female | 70 | Mild | BA.5.2 (Omicorn) | 2022-09-01 20:14 | 2022/9/ 1 | 2022-09- 15 10:04 | 14 |
| BA.5 | 294884 | female | 53 | Moderate | BA.5.2 (Omicorn) | 2022-09-01 19:16 | 2022/8/31 | 2022-09- 15 11:47 | 14 |
| BA.5 | 294885 | male | 51 | Asymptomatic | BA.5.2 (Omicorn) | 2022-09-01 19:31 | 2022/8/31 | 2022-09- 11 9:00 | 10 |
| BA.5 | 294886 | female | 19 | Mild | BA.5.2 (Omicorn) | 2022-09-01 21:01 | 2022/8/31 | 2022-09- 16 11:48 | 15 |
| BA.5 | 294888 | female | 24 | Mild | BA.5.2 (Omicorn) | 2022-09-01 21:02 | 2022/8/31 | 2022-09- 16 11:00 | 15 |
| BA.5 | 294889 | female | 22 | Mild | BA.5.2 (Omicorn) | 2022-09-01 21:01 | 2022/8/31 | 2022-09- 13 8:20 | 11 |
| BA.5 | 294890 | male | 37 | Mild | BA.5.2 (Omicorn) | 2022-09-01 20:05 | 2022/9/ 1 | 2022-09- 10 10:00 | 9 |
| BA.5 | 294891 | female | 50 | Mild | BA.5.2 (Omicorn) | 2022-09-01 20:40 | 2022/8/31 | 2022-09- 17 8:28 | 15 |
| BA.5 | 294892 | female | 83 | Mild | BA.5.2 (Omicorn) | 2022-09-01 20:41 | 2022/8/31 | 2022-09- 17 8:17 | 15 |
| BA.5 | 294893 | female | 21 | Mild | BA.5.2 (Omicorn) | 2022-09-01 20:41 | 2022/9/ 1 | 2022-09- 15 10:00 | 14 |
| BA.5 | 294894 | male | 50 | Mild | BA.5.2 (Omicorn) | 2022-09-01 22:21 | 2022/8/31 | 2022-09-20 10:00 | 18 |
| BA.5 | 294895 | female | 1 | Mild | BA.5.2 (Omicorn) | 2022-09-01 21:56 | 2022/9/ 1 | 2022-09- 12 9:23 | 10 |
| BA.5 | 294896 | male | 41 | Asymptomatic | BA.5.2 (Omicorn) | 2022-09-01 21:05 | 2022/8/31 | 2022-09- 13 8:20 | 11 |
| BA.5 | 294898 | male | 60 | Asymptomatic | BA.5.2 (Omicorn) | 2022-09-01 21:58 | 2022/8/31 | 2022-09- 12 10:05 | 11 |
| BA.5 | 294900 | male | 40 | Asymptomatic | BA.5.2 (Omicorn) | 2022-09-01 23:47 | 2022/9/ 1 | 2022-09- 13 8:36 | 11 |
| BA.5 | 294901 | male | 34 | Asymptomatic | BA.5.2 (Omicorn) | 2022-09-01 22:59 | 2022/9/ 1 | 2022-09- 16 11:00 | 15 |
| BA.5 | 294902 | male | 16 | Asymptomatic | BA.5.2 (Omicorn) | 2022-09-01 23:46 | 2022/8/31 | 2022-09-21 8:12 | 19 |
| BA.5 | 294903 | male | 22 | Asymptomatic | BA.5.2 (Omicorn) | 2022-09-01 22:59 | 2022/9/ 1 | 2022-09- 15 11:45 | 14 |
| BA.5 | 294904 | female | 47 | Asymptomatic | BA.5.2 (Omicorn) | 2022-09-01 22:20 | 2022/8/31 | 2022-09- 17 8:25 | 15 |
| BA.5 | 294905 | male | 48 | Asymptomatic | BA.5.2 (Omicorn) | 2022-09-01 22:22 | 2022/8/31 | 2022-09-20 10:00 | 18 |
| BA.5 | 294906 | male | 29 | Asymptomatic | BA.5.2 (Omicorn) | 2022-09-01 22:58 | 2022/9/ 1 | 2022-09- 13 8:20 | 11 |

| BA.5 | 294907 | male | 39 | Asymptomatic | BA.5.2 (Omicorn) | 2022-09-01 23:47 | 2022/8/31 | 2022-09- 18 10:00 | 16 |
| --- | --- | --- | --- | --- | --- | --- | --- | --- | --- |
| BA.5 | 294908 | male | 33 | Asymptomatic | BA.5.2 (Omicorn) | 2022-09-01 22:20 | 2022/9/ 1 | 2022-09- 19 9:00 | 17 |
| BA.5 | 294909 | male | 34 | Mild | BA.5.2 (Omicorn) | 2022-09-02 2:56 | 2022/9/ 1 | 2022-09- 14 9:00 | 12 |
| BA.5 | 294916 | male | 9 | Mild | BA.5.2 (Omicorn) | 2022-09-01 22:45 | 2022/8/31 | 2022-09- 18 10:00 | 16 |
| BA.5 | 294917 | male | 11 | Mild | BA.5.2 (Omicorn) | 2022-09-01 22:46 | 2022/8/31 | 2022-09- 12 9:23 | 10 |
| BA.5 | 294919 | female | 32 | Mild | BA.5.2 (Omicorn) | 2022-09-01 23:45 | 2022/9/ 1 | 2022-09- 14 9:00 | 12 |
| BA.5 | 294921 | female | 25 | Mild | BA.5.2 (Omicorn) | 2022-09-02 1:06 | 2022/9/ 1 | 2022-09- 18 10:00 | 16 |
| BA.5 | 294922 | female | 47 | Mild | BA.5.2 (Omicorn) | 2022-09-02 2:31 | 2022/9/ 1 | 2022-09- 15 8:59 | 13 |
| BA.5 | 294923 | female | 63 | Mild | BA.5.2 (Omicorn) | 2022-09-02 2:17 | 2022/9/ 1 | 2022-09- 18 11:00 | 16 |
| BA.5 | 294925 | female | 49 | Mild | BA.5.2 (Omicorn) | 2022-09-02 2:11 | 2022/9/ 1 | 2022-09- 17 8:18 | 15 |
| BA.5 | 294926 | female | 5 | Asymptomatic | BA.5.2 (Omicorn) | 2022-09-02 2:10 | 2022/9/ 1 | 2022-09- 15 10:00 | 13 |
| BA.5 | 294929 | male | 44 | Mild | BA.5.2 (Omicorn) | 2022-09-02 3:24 | 2022/9/ 1 | 2022-09- 16 10:00 | 14 |
| BA.5 | 294930 | female | 54 | Mild | BA.5.2 (Omicorn) | 2022-09-02 1:25 | 2022/9/2 | 2022-09-25 0:00 | 23 |
| BA.5 | 294935 | female | 54 | Asymptomatic | BA.5.2 (Omicorn) | 2022-09-02 2:43 | 2022/9/ 1 | 2022-09- 11 10:00 | 9 |
| BA.5 | 294937 | female | 51 | Mild | BA.5.2 (Omicorn) | 2022-09-02 1:50 | 2022/9/2 | 2022-09- 19 9:30 | 17 |
| BA.5 | 294938 | female | 23 | Mild | BA.5.2 (Omicorn) | 2022-09-02 1:50 | 2022/9/2 | 2022-09- 14 9:00 | 12 |
| BA.5 | 294940 | female | 71 | Asymptomatic | BA.5.2 (Omicorn) | 2022-09-02 2:31 | 2022/9/ 1 | 2022-09- 17 8:23 | 15 |
| BA.5 | 294941 | female | 27 | Mild | BA.5.2 (Omicorn) | 2022-09-02 2:54 | 2022/9/2 | 2022-09- 13 8:40 | 11 |
| BA.5 | 294942 | female | 1 | Mild | BA.5.2 (Omicorn) | 2022-09-02 10:25 | 2022/9/2 | 2022-09-09 9:04 | 7 |
| BA.5 | 294943 | female | 34 | Mild | BA.5.2 (Omicorn) | 2022-09-02 10:24 | 2022/9/ 1 | 2022-09- 13 10:00 | 11 |
| BA.5 | 294944 | female | 56 | Asymptomatic | BA.5.2 (Omicorn) | 2022-09-02 12:32 | 2022/9/ 1 | 2022-09- 14 9:54 | 12 |
| BA.5 | 294948 | female | 31 | Mild | BA.5.2 (Omicorn) | 2022-09-02 2:23 | 2022/9/ 1 | 2022-09- 14 8:35 | 12 |
| BA.5 | 294949 | female | 3 | Mild | BA.5.2 (Omicorn) | 2022-09-02 2:23 | 2022/9/2 | 2022-09-09 10:38 | 7 |
| BA.5 | 294950 | male | 33 | Mild | BA.5.2 (Omicorn) | 2022-09-02 2:23 | 2022/9/ 1 | 2022-09-24 0:00 | 22 |
| BA.5 | 294951 | male | 34 | Mild | BA.5.2 (Omicorn) | 2022-09-02 3:33 | 2022/9/ 1 | 2022-09- 12 9:40 | 10 |
| BA.5 | 294953 | female | 51 | Asymptomatic | BA.5.2 (Omicorn) | 2022-09-02 2:23 | 2022/9/ 1 | 2022-09- 18 10:00 | 16 |
| BA.5 | 294954 | male | 42 | Mild | BA.5.2 (Omicorn) | 2022-09-02 3:35 | 2022/9/ 1 | 2022-09- 12 10:00 | 10 |

| BA.5 | 294955 | male | 35 | Mild | BA.5.2 (Omicorn) | 2022-09-02 4:08 | 2022/9/2 | 2022-09- 13 10:00 | 11 |
| --- | --- | --- | --- | --- | --- | --- | --- | --- | --- |
| BA.5 | 294956 | female | 57 | Asymptomatic | BA.5.2 (Omicorn) | 2022-09-02 4:08 | 2022/9/ 1 | 2022-09- 19 9:30 | 17 |
| BA.5 | 294957 | male | 46 | Mild | BA.5.2 (Omicorn) | 2022-09-02 5:00 | 2022/9/ 1 | 2022-09- 18 10:00 | 16 |
| BA.5 | 294958 | female | 7 | Mild | BA.5.2 (Omicorn) | 2022-09-02 2:57 | 2022/9/ 1 | 2022-09- 13 10:00 | 11 |
| BA.5 | 294959 | female | 55 | Mild | BA.5.2 (Omicorn) | 2022-09-02 2:58 | 2022/9/ 1 | 2022-09-20 11:00 | 18 |
| BA.5 | 294960 | female | 30 | Mild | BA.5.2 (Omicorn) | 2022-09-02 2:56 | 2022/9/ 1 | 2022-09- 14 9:00 | 12 |
| BA.5 | 294961 | female | 44 | Mild | BA.5.2 (Omicorn) | 2022-09-02 5:00 | 2022/9/ 1 | 2022-09- 14 9:31 | 12 |
| BA.5 | 294962 | male | 62 | Mild | BA.5.2 (Omicorn) | 2022-09-02 3:21 | 2022/9/ 1 | 2022-09-21 12:00 | 19 |
| BA.5 | 294963 | female | 29 | Mild | BA.5.2 (Omicorn) | 2022-09-02 3:32 | 2022/9/ 1 | 2022-09- 19 9:30 | 17 |
| BA.5 | 294964 | female | 1 | Mild | BA.5.2 (Omicorn) | 2022-09-02 3:57 | 2022/9/ 1 | 2022-09- 11 9:00 | 9 |
| BA.5 | 294965 | female | 4 | Asymptomatic | BA.5.2 (Omicorn) | 2022-09-02 5:49 | 2022/9/ 1 | 2022-09- 15 10:01 | 13 |
| BA.5 | 294966 | female | 47 | Asymptomatic | BA.5.2 (Omicorn) | 2022-09-02 5:08 | 2022/9/ 1 | 2022-09- 13 10:00 | 11 |
| BA.5 | 294967 | female | 41 | Asymptomatic | BA.5.2 (Omicorn) | 2022-09-02 6:25 | 2022/9/2 | 2022-09- 10 9:00 | 8 |
| BA.5 | 294968 | male | 34 | Mild | BA.5.2 (Omicorn) | 2022-09-02 10:26 | 2022/9/2 | 2022-09- 16 9:00 | 14 |
| BA.5 | 294970 | male | 54 | Asymptomatic | BA.5.2 (Omicorn) | 2022-09-02 5:29 | 2022/9/2 | 2022-09- 18 11:00 | 16 |
| BA.5 | 294972 | male | 26 | Mild | BA.5.2 (Omicorn) | 2022-09-02 8:24 | 2022/9/ 1 | 2022-09- 13 10:00 | 11 |
| BA.5 | 294974 | male | 39 | Mild | BA.5.2 (Omicorn) | 2022-09-02 9:23 | 2022/9/ 1 | 2022-09- 17 10:00 | 15 |
| BA.5 | 294975 | male | 62 | Mild | BA.5.2 (Omicorn) | 2022-09-02 10:19 | 2022/9/ 1 | 2022-09-22 0:00 | 20 |
| BA.5 | 294977 | female | 32 | Mild | BA.5.2 (Omicorn) | 2022-09-02 10:37 | 2022/9/ 1 | 2022-09- 14 9:34 | 12 |
| BA.5 | 294982 | male | 57 | Mild | BA.5.2 (Omicorn) | 2022-09-02 12:35 | 2022/9/2 | 2022-09- 17 8:19 | 15 |
| BA.5 | 294990 | female | 53 | Asymptomatic | BA.5.2 (Omicorn) | 2022-09-02 10:39 | 2022/9/ 1 | 2022-09- 17 10:00 | 15 |
| BA.5 | 294999 | male | 63 | Asymptomatic | BA.5.2 (Omicorn) | 2022-09-02 13:22 | 2022/9/ 1 | 2022-09- 12 10:00 | 10 |
| BA.5 | 295009 | male | 49 | Mild | BA.5.2 (Omicorn) | 2022-09-02 12:07 | 2022/9/ 1 | 2022-09- 16 8:41 | 14 |
| BA.5 | 295015 | female | 36 | Mild | BA.5.2 (Omicorn) | 2022-09-02 10:55 | 2022/9/ 1 | 2022-09- 18 10:00 | 16 |
| BA.5 | 295029 | female | 46 | Asymptomatic | BA.5.2 (Omicorn) | 2022-09-02 12:36 | 2022/9/ 1 | 2022-09- 19 10:00 | 17 |
| BA.5 | 295030 | male | 55 | Asymptomatic | BA.5.2 (Omicorn) | 2022-09-02 15:39 | 2022/9/2 | 2022-09- 16 8:44 | 14 |
| BA.5 | 295031 | male | 46 | Mild | BA.5.2 (Omicorn) | 2022-09-02 15:40 | 2022/9/2 | 2022-09-21 8:12 | 19 |

| BA.5 | 295034 | male | 11 | Mild | BA.5.2 (Omicorn) | 2022-09-02 16:16 | 2022/9/2 | 2022-09- 13 10:00 | 11 |
| --- | --- | --- | --- | --- | --- | --- | --- | --- | --- |
| BA.5 | 295035 | female | 12 | Mild | BA.5.2 (Omicorn) | 2022-09-02 16:18 | 2022/9/2 | 2022-09- 13 10:00 | 11 |
| BA.5 | 295036 | female | 40 | Mild | BA.5.2 (Omicorn) | 2022-09-02 16:20 | 2022/9/2 | 2022-09- 13 10:00 | 11 |
| BA.5 | 295037 | female | 10 | Mild | BA.5.2 (Omicorn) | 2022-09-02 21:26 | 2022/9/2 | 2022-09- 13 10:00 | 11 |
| BA.5 | 295038 | male | 12 | Mild | BA.5.2 (Omicorn) | 2022-09-02 16:38 | 2022/9/2 | 2022-09-22 8:00 | 20 |
| BA.5 | 295040 | male | 20 | Asymptomatic | BA.5.2 (Omicorn) | 2022-09-02 14:06 | 2022/9/2 | 2022-09- 18 10:00 | 16 |
| BA.5 | 295041 | female | 24 | Mild | BA.5.2 (Omicorn) | 2022-09-02 14:26 | 2022/9/ 1 | 2022-09- 16 10:00 | 14 |
| BA.5 | 295047 | male | 30 | Mild | BA.5.2 (Omicorn) | 2022-09-02 14:39 | 2022/9/2 | 2022-09- 12 10:00 | 10 |
| BA.5 | 295048 | male | 58 | Mild | BA.5.2 (Omicorn) | 2022-09-02 14:44 | 2022/9/2 | 2022-09- 12 10:00 | 10 |
| BA.5 | 295051 | female | 30 | Mild | BA.5.2 (Omicorn) | 2022-09-02 16:04 | 2022/9/2 | 2022-09- 14 10:00 | 12 |
| BA.5 | 295052 | male | 51 | Mild | BA.5.2 (Omicorn) | 2022-09-02 17:49 | 2022/9/ 1 | 2022-09- 13 10:00 | 11 |
| BA.5 | 295054 | female | 50 | Mild | BA.5.2 (Omicorn) | 2022-09-02 17:29 | 2022/9/2 | 2022-09- 18 10:00 | 16 |
| BA.5 | 295055 | male | 43 | Asymptomatic | BA.5.2 (Omicorn) | 2022-09-02 17:30 | 2022/9/2 | 2022-09- 13 10:00 | 11 |
| BA.5 | 295056 | female | 3 | Mild | BA.5.2 (Omicorn) | 2022-09-02 17:46 | 2022/9/ 1 | 2022-09- 14 10:00 | 12 |
| BA.5 | 295057 | male | 37 | Mild | BA.5.2 (Omicorn) | 2022-09-02 16:03 | 2022/9/ 1 | 2022-09- 17 8:22 | 15 |
| BA.5 | 295058 | female | 39 | Asymptomatic | BA.5.2 (Omicorn) | 2022-09-02 16:45 | 2022/9/2 | 2022-09- 12 10:00 | 10 |
| BA.5 | 295059 | female | 40 | Mild | BA.5.2 (Omicorn) | 2022-09-02 19:13 | 2022/9/2 | 2022-09- 18 12:00 | 16 |
| BA.5 | 295061 | female | 50 | Mild | BA.5.2 (Omicorn) | 2022-09-02 17:32 | 2022/9/2 | 2022-09- 16 9:00 | 14 |
| BA.5 | 295063 | male | 85 | Asymptomatic | BA.5.2 (Omicorn) | 2022-09-02 19:09 | 2022/9/2 | 2022-09- 19 17:00 | 17 |
| BA.5 | 295064 | female | 81 | Asymptomatic | BA.5.2 (Omicorn) | 2022-09-02 19:12 | 2022/9/2 | 2022-09- 19 17:00 | 17 |
| BA.5 | 295069 | female | 29 | Mild | BA.5.2 (Omicorn) | 2022-09-02 19:48 | 2022/9/2 | 2022-09- 12 10:00 | 10 |
| BA.5 | 295070 | female | 13 | Mild | BA.5.2 (Omicorn) | 2022-09-02 19:58 | 2022/9/2 | 2022-09- 12 10:00 | 10 |
| BA.5 | 295071 | female | 45 | Mild | BA.5.2 (Omicorn) | 2022-09-02 20:00 | 2022/9/2 | 2022-09- 12 10:00 | 10 |
| BA.5 | 295072 | female | 54 | Asymptomatic | BA.5.2 (Omicorn) | 2022-09-02 19:28 | 2022/9/2 | 2022-09- 12 10:00 | 10 |
| BA.5 | 295074 | female | 43 | Mild | BA.5.2 (Omicorn) | 2022-09-02 20:30 | 2022/9/ 1 | 2022-09- 17 10:00 | 15 |
| BA.5 | 295076 | male | 11 | Mild | BA.5.2 (Omicorn) | 2022-09-02 21:56 | 2022/9/ 1 | 2022-09- 14 10:00 | 12 |
| BA.5 | 295077 | male | 58 | Asymptomatic | BA.5.2 (Omicorn) | 2022-09-02 21:57 | 2022/9/ 1 | 2022-09-20 10:00 | 18 |

| BA.5 | 295079 | male | 48 | Mild | BA.5.2 (Omicorn) | 2022-09-02 20:22 | 2022/9/2 | 2022-09- 16 9:00 | 14 |
| --- | --- | --- | --- | --- | --- | --- | --- | --- | --- |
| BA.5 | 295080 | female | 21 | Moderate | BA.5.2 (Omicorn) | 2022-09-02 20:32 | 2022/9/ 1 | 2022-09- 15 11:46 | 13 |
| BA.5 | 295081 | female | 54 | Mild | BA.5.2 (Omicorn) | 2022-09-02 21:58 | 2022/9/ 1 | 2022-09- 13 10:00 | 11 |
| BA.5 | 295087 | female | 33 | Asymptomatic | BA.5.2 (Omicorn) | 2022-09-02 22:11 | 2022/9/2 | 2022-09-21 9:00 | 18 |
| BA.5 | 295090 | female | 67 | Asymptomatic | BA.5.2 (Omicorn) | 2022-09-02 22:03 | 2022/9/ 1 | 2022-09-21 8:52 | 18 |
| BA.5 | 295092 | female | 18 | Mild | BA.5.2 (Omicorn) | 2022-09-02 22:48 | 2022/9/ 1 | 2022-09- 12 10:00 | 9 |
| BA.5 | 295094 | female | 31 | Mild | BA.5.2 (Omicorn) | 2022-09-03 0:37 | 2022/9/2 | 2022-09-23 0:00 | 20 |
| BA.5 | 295095 | male | 31 | Asymptomatic | BA.5.2 (Omicorn) | 2022-09-03 0:35 | 2022/9/2 | 2022-09- 14 10:00 | 11 |
| BA.5 | 295100 | female | 52 | Moderate | BA.5.2 (Omicorn) | 2022-09-03 0:41 | 2022/9/2 | 2022-09- 12 10:00 | 9 |
| BA.5 | 295101 | female | 14 | Mild | BA.5.2 (Omicorn) | 2022-09-02 23:14 | 2022/9/2 | 2022-09- 12 10:00 | 9 |
| BA.5 | 295102 | male | 54 | Asymptomatic | BA.5.2 (Omicorn) | 2022-09-03 5:22 | 2022/9/2 | 2022-09-20 10:00 | 17 |
| BA.5 | 295106 | female | 46 | Mild | BA.5.2 (Omicorn) | 2022-09-03 1:43 | 2022/9/2 | 2022-09- 17 8:22 | 14 |
| BA.5 | 295109 | female | 10 | Mild | BA.5.2 (Omicorn) | 2022-09-03 1:36 | 2022/9/2 | 2022-09- 18 12:18 | 15 |
| BA.5 | 295112 | female | 24 | Mild | BA.5.2 (Omicorn) | 2022-09-03 3:31 | 2022/9/2 | 2022-09- 18 10:00 | 15 |
| BA.5 | 295113 | female | 30 | Mild | BA.5.2 (Omicorn) | 2022-09-03 3:29 | 2022/9/2 | 2022-09- 16 10:00 | 13 |
| BA.5 | 295114 | female | 37 | Mild | BA.5.2 (Omicorn) | 2022-09-03 3:43 | 2022/9/2 | 2022-09- 17 10:00 | 14 |
| BA.5 | 295115 | female | 9 | Mild | BA.5.2 (Omicorn) | 2022-09-03 3:43 | 2022/9/2 | 2022-09- 15 8:01 | 12 |
| BA.5 | 295117 | male | 21 | Mild | BA.5.2 (Omicorn) | 2022-09-03 4:25 | 2022/9/2 | 2022-09- 15 10:00 | 12 |
| BA.5 | 295118 | female | 39 | Mild | BA.5.2 (Omicorn) | 2022-09-03 4:09 | 2022/9/2 | 2022-09- 12 10:00 | 9 |
| BA.5 | 295122 | male | 36 | Mild | BA.5.2 (Omicorn) | 2022-09-03 6:48 | 2022/9/2 | 2022-09- 16 9:00 | 13 |
| BA.5 | 295124 | female | 26 | Mild | BA.5.2 (Omicorn) | 2022-09-03 11:52 | 2022/9/2 | 2022-09- 15 11:13 | 12 |
| BA.5 | 295125 | male | 31 | Asymptomatic | BA.5.2 (Omicorn) | 2022-09-03 6:46 | 2022/9/2 | 2022-09- 13 10:00 | 10 |
| BA.5 | 295126 | male | 5 | Mild | BA.5.2 (Omicorn) | 2022-09-03 12:58 | 2022/9/2 | 2022-09- 14 9:45 | 11 |
| BA.5 | 295127 | female | 57 | Mild | BA.5.2 (Omicorn) | 2022-09-03 11:58 | 2022/9/2 | 2022-09- 14 8:00 | 11 |
| BA.5 | 295128 | female | 48 | Mild | BA.5.2 (Omicorn) | 2022-09-03 11:53 | 2022/9/2 | 2022-09-20 9:27 | 17 |
| BA.5 | 295129 | male | 43 | Mild | BA.5.2 (Omicorn) | 2022-09-03 8:01 | 2022/9/2 | 2022-09- 16 16:13 | 13 |
| BA.5 | 295130 | female | 44 | Mild | BA.5.2 (Omicorn) | 2022-09-03 8:01 | 2022/9/2 | 2022-09- 14 20:25 | 12 |

| BA.5 | 295131 | female | 10 | Mild | BA.5.2 (Omicorn) | 2022-09-03 10:00 | 2022/9/2 | 2022-09- 17 9:00 | 14 |
| --- | --- | --- | --- | --- | --- | --- | --- | --- | --- |
| BA.5 | 295133 | male | 26 | Mild | BA.5.2 (Omicorn) | 2022-09-03 9:28 | 2022/9/2 | 2022-09- 11 10:00 | 8 |
| BA.5 | 295144 | female | 22 | Mild | BA.5.2 (Omicorn) | 2022-09-03 9:29 | 2022/9/2 | 2022-09- 12 10:00 | 9 |
| BA.5 | 295148 | female | 58 | Mild | BA.5.2 (Omicorn) | 2022-09-03 9:29 | 2022/9/2 | 2022-09- 13 10:00 | 10 |
| BA.5 | 295160 | male | 64 | Asymptomatic | BA.5.2 (Omicorn) | 2022-09-03 11:29 | 2022/9/2 | 2022-09- 18 10:00 | 15 |
| BA.5 | 295174 | female | 4 | Mild | BA.5.2 (Omicorn) | 2022-09-03 12:30 | 2022/9/3 | 2022-09-20 8:37 | 17 |
| BA.5 | 295175 | female | 24 | Mild | BA.5.2 (Omicorn) | 2022-09-03 16:22 | 2022/9/2 | 2022-09-22 10:00 | 19 |
| BA.5 | 295177 | female | 30 | Asymptomatic | BA.5.2 (Omicorn) | 2022-09-03 14:38 | 2022/9/3 | 2022-09- 18 12:00 | 15 |
| BA.5 | 295180 | female | 46 | Mild | BA.5.2 (Omicorn) | 2022-09-03 14:40 | 2022/9/2 | 2022-09- 12 10:00 | 9 |
| BA.5 | 295181 | female | 49 | Moderate | BA.5.2 (Omicorn) | 2022-09-03 16:17 | 2022/9/3 | 2022-09- 16 10:31 | 13 |
| BA.5 | 295182 | female | 46 | Moderate | BA.5.2 (Omicorn) | 2022-09-03 15:04 | 2022/9/2 | 2022-09- 15 11:15 | 12 |
| BA.5 | 295183 | male | 25 | Mild | BA.5.2 (Omicorn) | 2022-09-03 15:06 | 2022/9/3 | 2022-09- 15 10:46 | 12 |
| BA.5 | 295184 | female | 43 | Mild | BA.5.2 (Omicorn) | 2022-09-03 16:16 | 2022/9/2 | 2022-09- 14 8:13 | 11 |
| BA.5 | 295185 | female | 52 | Mild | BA.5.2 (Omicorn) | 2022-09-03 16:22 | 2022/9/2 | 2022-09-20 9:30 | 17 |
| BA.5 | 295187 | female | 47 | Mild | BA.5.2 (Omicorn) | 2022-09-03 16:26 | 2022/9/3 | 2022-09- 18 9:13 | 15 |
| BA.5 | 295188 | female | 50 | Mild | BA.5.2 (Omicorn) | 2022-09-03 15:02 | 2022/9/ 1 | 2022-09-20 9:02 | 17 |
| BA.5 | 295193 | female | 30 | Mild | BA.5.2 (Omicorn) | 2022-09-03 14:42 | 2022/9/2 | 2022-09- 15 11:11 | 12 |
| BA.5 | 295194 | female | 45 | Mild | BA.5.2 (Omicorn) | 2022-09-03 16:19 | 2022/9/2 | 2022-09- 15 10:41 | 12 |
| BA.5 | 295195 | female | 46 | Mild | BA.5.2 (Omicorn) | 2022-09-03 14:41 | 2022/9/2 | 2022-09-20 9:19 | 17 |
| BA.5 | 295197 | male | 37 | Moderate | BA.5.2 (Omicorn) | 2022-09-03 14:52 | 2022/9/3 | 2022-09- 17 9:00 | 14 |
| BA.5 | 295198 | female | 27 | Mild | BA.5.2 (Omicorn) | 2022-09-03 14:28 | 2022/9/2 | 2022-09- 19 9:19 | 16 |
| BA.5 | 295199 | female | 13 | Mild | BA.5.2 (Omicorn) | 2022-09-03 16:25 | 2022/9/2 | 2022-09-20 10:00 | 17 |
| BA.5 | 295201 | male | 33 | Moderate | BA.5.2 (Omicorn) | 2022-09-03 14:27 | 2022/9/2 | 2022-09- 12 10:00 | 9 |
| BA.5 | 295202 | male | 48 | Asymptomatic | BA.5.2 (Omicorn) | 2022-09-03 16:23 | 2022/9/2 | 2022-09- 17 10:00 | 14 |
| BA.5 | 295203 | female | 54 | Mild | BA.5.2 (Omicorn) | 2022-09-03 14:29 | 2022/9/ 1 | 2022-09-20 9:31 | 17 |
| BA.5 | 295205 | male | 38 | Mild | BA.5.2 (Omicorn) | 2022-09-03 17:16 | 2022/9/2 | 2022-09- 14 8:21 | 11 |
| BA.5 | 295207 | female | 39 | Asymptomatic | BA.5.2 (Omicorn) | 2022-09-03 16:33 | 2022/9/2 | 2022-09- 18 9:34 | 15 |

| BA.5 | 295210 | male | 34 | Mild | BA.5.2 (Omicorn) | 2022-09-03 16:34 | 2022/9/2 | 2022-09- 16 8:00 | 13 |
| --- | --- | --- | --- | --- | --- | --- | --- | --- | --- |
| BA.5 | 295214 | female | 36 | Mild | BA.5.2 (Omicorn) | 2022-09-03 15:21 | 2022/9/3 | 2022-09- 17 10:00 | 14 |
| BA.5 | 295216 | female | 29 | Moderate | BA.5.2 (Omicorn) | 2022-09-03 15:15 | 2022/9/2 | 2022-09- 15 11:17 | 12 |
| BA.5 | 295222 | female | 14 | Mild | BA.5.2 (Omicorn) | 2022-09-03 18:01 | 2022/9/3 | 2022-09- 19 10:00 | 16 |
| BA.5 | 295223 | male | 28 | Moderate | BA.5.2 (Omicorn) | 2022-09-03 16:59 | 2022/9/3 | 2022-09- 17 10:00 | 14 |
| BA.5 | 295224 | male | 33 | Asymptomatic | BA.5.2 (Omicorn) | 2022-09-03 18:12 | 2022/9/2 | 2022-09- 14 8:13 | 11 |
| BA.5 | 295230 | female | 87 | Asymptomatic | BA.5.2 (Omicorn) | 2022-09-03 22:31 | 2022/9/3 | 2022-09-09 19:00 | 6 |
| BA.5 | 295231 | female | 27 | Mild | BA.5.2 (Omicorn) | 2022-09-03 21:48 | 2022/9/3 | 2022-09- 17 10:00 | 14 |
| BA.5 | 295232 | female | 47 | Mild | BA.5.2 (Omicorn) | 2022-09-03 21:26 | 2022/9/3 | 2022-09-20 10:00 | 17 |
| BA.5 | 295236 | male | 30 | Mild | BA.5.2 (Omicorn) | 2022-09-03 20:53 | 2022/9/3 | 2022-09- 15 11:17 | 12 |
| BA.5 | 295244 | male | 16 | Mild | BA.5.2 (Omicorn) | 2022-09-04 0:15 | 2022/9/3 | 2022-09-20 9:57 | 16 |
| BA.5 | 295245 | female | 67 | Asymptomatic | BA.5.2 (Omicorn) | 2022-09-03 22:50 | 2022/9/3 | 2022-09-20 15:30 | 17 |
| BA.5 | 295247 | male | 37 | Mild | BA.5.2 (Omicorn) | 2022-09-03 22:28 | 2022/9/ 1 | 2022-09- 14 8:23 | 10 |
| BA.5 | 295248 | female | 11 | Asymptomatic | BA.5.2 (Omicorn) | 2022-09-04 0:28 | 2022/9/3 | 2022-09-20 0:00 | 16 |
| BA.5 | 295249 | female | 34 | Mild | BA.5.2 (Omicorn) | 2022-09-03 22:30 | 2022/9/3 | 2022-09- 12 10:00 | 8 |
| BA.5 | 295252 | male | 30 | Mild | BA.5.2 (Omicorn) | 2022-09-04 0:59 | 2022/9/3 | 2022-09- 15 10:56 | 11 |
| BA.5 | 295256 | female | 36 | Mild | BA.5.2 (Omicorn) | 2022-09-04 1:30 | 2022/9/3 | 2022-09- 17 8:14 | 13 |
| BA.5 | 295260 | male | 73 | Moderate | BA.5.2 (Omicorn) | 2022-09-04 0:49 | 2022/9/3 | 2022-09- 15 10:00 | 11 |
| BA.5 | 295261 | female | 1 | Mild | BA.5.2 (Omicorn) | 2022-09-04 1:47 | 2022/9/3 | 2022-09- 14 15:51 | 11 |
| BA.5 | 295262 | male | 50 | Asymptomatic | BA.5.2 (Omicorn) | 2022-09-04 3:00 | 2022/9/3 | 2022-09- 16 10:00 | 12 |
| BA.5 | 295266 | male | 29 | Mild | BA.5.2 (Omicorn) | 2022-09-04 3:46 | 2022/9/3 | 2022-09- 15 11:08 | 11 |
| BA.5 | 295267 | male | 28 | Mild | BA.5.2 (Omicorn) | 2022-09-04 3:46 | 2022/9/3 | 2022-09- 13 8:07 | 9 |
| BA.5 | 295268 | male | 56 | Asymptomatic | BA.5.2 (Omicorn) | 2022-09-04 3:47 | 2022/9/3 | 2022-09-20 8:54 | 16 |
| BA.5 | 295270 | male | 51 | Mild | BA.5.2 (Omicorn) | 2022-09-04 3:48 | 2022/9/3 | 2022-09-20 9:01 | 16 |
| BA.5 | 295271 | female | 40 | Mild | BA.5.2 (Omicorn) | 2022-09-04 4:15 | 2022/9/3 | 2022-09- 16 10:00 | 12 |
| BA.5 | 295277 | male | 28 | Mild | BA.5.2 (Omicorn) | 2022-09-04 8:06 | 2022/9/3 | 2022-09- 14 8:23 | 10 |
| BA.5 | 295278 | male | 30 | Asymptomatic | BA.5.2 (Omicorn) | 2022-09-04 8:06 | 2022/9/3 | 2022-09- 15 11:11 | 11 |

| BA.5 | 295286 | female | 45 | Asymptomatic | BA.5.2 (Omicorn) | 2022-09-04 8:53 | 2022/9/3 | 2022-09- 13 10:00 | 9 |
| --- | --- | --- | --- | --- | --- | --- | --- | --- | --- |
| BA.5 | 295288 | female | 57 | Moderate | BA.5.2 (Omicorn) | 2022-09-04 12:11 | 2022/9/3 | 2022-09-21 10:00 | 17 |
| BA.5 | 295308 | male | 41 | Mild | BA.5.2 (Omicorn) | 2022-09-04 13:26 | 2022/9/4 | 2022-09-27 0:00 | 22 |
| BA.5 | 295329 | female | 40 | Asymptomatic | BA.5.2 (Omicorn) | 2022-09-04 15:20 | 2022/9/4 | 2022-09- 18 10:07 | 14 |
| BA.5 | 295333 | female | 22 | Mild | BA.5.2 (Omicorn) | 2022-09-04 15:19 | 2022/9/4 | 2022-09- 15 10:00 | 11 |
| BA.5 | 295344 | male | 22 | Mild | BA.5.2 (Omicorn) | 2022-09-04 15:48 | 2022/9/4 | 2022-09- 17 10:00 | 13 |
| BA.5 | 295346 | male | 45 | Moderate | BA.5.2 (Omicorn) | 2022-09-04 15:49 | 2022/9/4 | 2022-09- 18 12:15 | 14 |
| BA.5 | 295347 | male | 30 | Mild | BA.5.2 (Omicorn) | 2022-09-04 15:49 | 2022/9/4 | 2022-09- 16 10:00 | 12 |
| BA.5 | 295356 | female | 20 | Asymptomatic | BA.5.2 (Omicorn) | 2022-09-04 19:37 | 2022/9/4 | 2022-09- 19 9:30 | 15 |
| BA.5 | 295357 | female | 27 | Mild | BA.5.2 (Omicorn) | 2022-09-04 20:05 | 2022/9/3 | 2022-09-23 0:00 | 18 |
| BA.5 | 295362 | female | 49 | Mild | BA.5.2 (Omicorn) | 2022-09-04 23:30 | 2022/9/3 | 2022-09- 18 10:00 | 13 |
| BA.5 | 295364 | female | 46 | Mild | BA.5.2 (Omicorn) | 2022-09-04 23:31 | 2022/9/4 | 2022-09- 17 10:00 | 12 |
| BA.5 | 295367 | male | 38 | Asymptomatic | BA.5.2 (Omicorn) | 2022-09-04 22:20 | 2022/9/3 | 2022-09-22 10:00 | 17 |
| BA.5 | 295368 | male | 32 | Asymptomatic | BA.5.2 (Omicorn) | 2022-09-04 22:54 | 2022/9/3 | 2022-09- 17 8:22 | 12 |
| BA.5 | 295371 | male | 35 | Mild | BA.5.2 (Omicorn) | 2022-09-04 23:23 | 2022/9/4 | 2022-09- 15 11:00 | 10 |
| BA.5 | 295372 | female | 8 | Asymptomatic | BA.5.2 (Omicorn) | 2022-09-04 23:34 | 2022/9/3 | 2022-09- 14 9:00 | 9 |
| BA.5 | 295373 | female | 38 | Asymptomatic | BA.5.2 (Omicorn) | 2022-09-04 23:30 | 2022/9/4 | 2022-09- 15 11:00 | 10 |
| BA.5 | 295375 | female | 67 | Mild | BA.5.2 (Omicorn) | 2022-09-05 4:42 | 2022/9/4 | 2022-09-20 10:00 | 15 |
| BA.5 | 295376 | male | 26 | Mild | BA.5.2 (Omicorn) | 2022-09-05 3:33 | 2022/9/4 | 2022-09- 14 9:00 | 9 |
| BA.5 | 295377 | male | 34 | Moderate | BA.5.2 (Omicorn) | 2022-09-05 2:10 | 2022/9/4 | 2022-09- 19 9:00 | 14 |
| BA.5 | 295378 | female | 53 | Mild | BA.5.2 (Omicorn) | 2022-09-05 2:11 | 2022/9/4 | 2022-09- 18 10:00 | 13 |
| BA.5 | 295379 | male | 22 | Asymptomatic | BA.5.2 (Omicorn) | 2022-09-05 2:12 | 2022/9/4 | 2022-09- 16 11:00 | 11 |
| BA.5 | 295386 | female | 33 | Asymptomatic | BA.5.2 (Omicorn) | 2022-09-05 4:34 | 2022/9/4 | 2022-09- 18 10:00 | 13 |
| BA.5 | 295388 | male | 33 | Mild | BA.5.2 (Omicorn) | 2022-09-05 3:59 | 2022/9/4 | 2022-09- 18 10:00 | 13 |
| BA.5 | 295389 | female | 25 | Mild | BA.5.2 (Omicorn) | 2022-09-05 4:35 | 2022/9/4 | 2022-09- 19 10:00 | 14 |
| BA.5 | 295390 | female | 37 | Mild | BA.5.2 (Omicorn) | 2022-09-05 5:52 | 2022/9/4 | 2022-09- 16 11:00 | 11 |
| BA.5 | 295391 | female | 2 | Mild | BA.5.2 (Omicorn) | 2022-09-05 5:53 | 2022/9/4 | 2022-09-08 10:00 | 3 |

| BA.5 | 295392 | male | 57 | Asymptomatic | BA.5.2 (Omicorn) | 2022-09-05 5:55 | 2022/9/4 | 2022-09-20 8:43 | 15 |
| --- | --- | --- | --- | --- | --- | --- | --- | --- | --- |
| BA.5 | 295393 | female | 52 | Mild | BA.5.2 (Omicorn) | 2022-09-05 5:54 | 2022/9/4 | 2022-09- 19 9:00 | 14 |
| BA.5 | 295394 | male | 51 | Mild | BA.5.2 (Omicorn) | 2022-09-05 5:56 | 2022/9/4 | 2022-09- 19 10:00 | 14 |
| BA.5 | 295395 | male | 25 | Mild | BA.5.2 (Omicorn) | 2022-09-05 4:55 | 2022/9/5 | 2022-09- 15 10:00 | 10 |
| BA.5 | 295396 | female | 30 | Mild | BA.5.2 (Omicorn) | 2022-09-05 5:15 | 2022/9/4 | 2022-09- 14 8:12 | 9 |
| BA.5 | 295397 | male | 3 | Mild | BA.5.2 (Omicorn) | 2022-09-05 4:37 | 2022/9/4 | 2022-09-20 9:25 | 15 |
| BA.5 | 295398 | female | 39 | Mild | BA.5.2 (Omicorn) | 2022-09-05 4:57 | 2022/9/5 | 2022-09- 17 10:00 | 12 |
| BA.5 | 295402 | male | 38 | Mild | BA.5.2 (Omicorn) | 2022-09-05 9:56 | 2022/9/4 | 2022-09-21 8:33 | 16 |
| BA.5 | 295403 | female | 35 | Asymptomatic | BA.5.2 (Omicorn) | 2022-09-05 9:55 | 2022/9/4 | 2022-09- 17 10:00 | 12 |
| BA.5 | 295405 | female | 22 | Asymptomatic | BA.5.2 (Omicorn) | 2022-09-05 8:37 | 2022/9/3 | 2022-09- 15 10:00 | 10 |
| BA.5 | 295406 | female | 25 | Asymptomatic | BA.5.2 (Omicorn) | 2022-09-05 7:55 | 2022/9/4 | 2022-09- 18 10:00 | 13 |
| BA.5 | 295408 | male | 25 | Mild | BA.5.2 (Omicorn) | 2022-09-05 10:02 | 2022/9/5 | 2022-09- 14 9:00 | 9 |
| BA.5 | 295445 | male | 25 | Mild | BA.5.2 (Omicorn) | 2022-09-05 11:03 | 2022/9/5 | 2022-09- 19 9:18 | 14 |
| BA.5 | 295446 | male | 19 | Mild | BA.5.2 (Omicorn) | 2022-09-05 11:45 | 2022/9/5 | 2022-09- 18 11:00 | 13 |
| BA.5 | 295448 | male | 48 | Mild | BA.5.2 (Omicorn) | 2022-09-05 11:32 | 2022/9/5 | 2022-09-23 0:00 | 18 |
| BA.5 | 295477 | female | 48 | Mild | BA.5.2 (Omicorn) | 2022-09-05 17:12 | 2022/9/5 | 2022-09-20 10:00 | 15 |
| BA.5 | 295479 | female | 53 | Mild | BA.5.2 (Omicorn) | 2022-09-05 19:57 | 2022/9/5 | 2022-09- 19 10:00 | 14 |
| BA.5 | 295482 | male | 34 | Mild | BA.5.2 (Omicorn) | 2022-09-05 21:27 | 2022/9/5 | 2022-09- 19 8:18 | 13 |
| BA.5 | 295487 | female | 4 | Mild | BA.5.2 (Omicorn) | 2022-09-05 21:26 | 2022/9/5 | 2022-09- 18 12:01 | 13 |
| BA.5 | 295488 | male | 10 | Mild | BA.5.2 (Omicorn) | 2022-09-05 21:27 | 2022/9/5 | 2022-09- 12 10:00 | 7 |
| BA.5 | 295490 | male | 47 | Mild | BA.5.2 (Omicorn) | 2022-09-05 23:46 | 2022/9/5 | 2022-09- 19 9:30 | 13 |
| BA.5 | 295499 | male | 72 | Asymptomatic B | A.5.6 (Omicorn) (Omicorn) | 2022-09-06 0:07 | 2022/9/5 | 2022-09-22 10:00 | 16 |
| BA.5 | 295500 | male | 39 | Mild | BA.5.2 (Omicorn) | 2022-09-06 1:14 | 2022/9/5 | 2022-09- 15 10:00 | 9 |
| BA.5 | 295501 | male | 36 | Mild | BA.5.2 (Omicorn) | 2022-09-06 3:36 | 2022/9/4 | 2022-09- 18 11:00 | 12 |
| BA.5 | 295503 | female | 30 | Moderate | BA.5.2 (Omicorn) | 2022-09-06 3:34 | 2022/9/5 | 2022-09-23 0:00 | 17 |
| BA.5 | 295505 | male | 58 | Asymptomatic | BA.5.2 (Omicorn) | 2022-09-06 2:36 | 2022/9/5 | 2022-09-26 0:00 | 20 |
| BA.5 | 295508 | female | 3 | Mild | BA.5.2 (Omicorn) | 2022-09-06 3:47 | 2022/9/5 | 2022-09- 14 8:01 | 8 |

| BA.5 | 295510 | female | 16 | Moderate | BA.5.2 (Omicorn) | 2022-09-06 4:30 | 2022/9/5 | 2022-09- 18 10:00 | 12 |
| --- | --- | --- | --- | --- | --- | --- | --- | --- | --- |
| BA.5 | 295511 | female | 53 | Mild | BA.5.2 (Omicorn) | 2022-09-06 7:21 | 2022/9/5 | 2022-09- 18 12:32 | 12 |
| BA.5 | 295512 | female | 24 | Asymptomatic | BA.5.2 (Omicorn) | 2022-09-06 4:43 | 2022/9/5 | 2022-09- 19 10:00 | 13 |
| BA.5 | 295514 | male | 29 | Moderate | BA.5.2 (Omicorn) | 2022-09-06 9:17 | 2022/9/5 | 2022-09-23 0:00 | 17 |
| BA.5 | 295528 | male | 37 | Mild | BA.5.2 (Omicorn) | 2022-09-06 10:19 | 2022/9/5 | 2022-09- 16 10:00 | 10 |
| BA.5 | 295546 | male | 31 | Asymptomatic | BA.5.2 (Omicorn) | 2022-09-06 12:41 | 2022/9/6 | 2022-09-20 10:15 | 14 |
| BA.5 | 295549 | male | 52 | Mild | BA.5.2 (Omicorn) | 2022-09-06 15:16 | 2022/9/6 | 2022-09- 16 10:00 | 10 |
| BA.5 | 295561 | female | 26 | Asymptomatic | BA.5.2 (Omicorn) | 2022-09-06 15:12 | 2022/9/6 | 2022-09- 19 10:00 | 13 |
| BA.5 | 295570 | female | 8 | Asymptomatic | BA.5.2 (Omicorn) | 2022-09-06 17:39 | 2022/9/6 | 2022-09-26 0:00 | 19 |
| BA.5 | 295572 | female | 26 | Mild | BA.5.2 (Omicorn) | 2022-09-06 16:37 | 2022/9/6 | 2022-09- 19 9:09 | 13 |
| BA.5 | 295583 | male | 52 | Asymptomatic | BA.5.2 (Omicorn) | 2022-09-06 20:29 | 2022/9/6 | 2022-09- 19 10:00 | 13 |
| BA.5 | 295584 | female | 41 | Asymptomatic | BA.5.2 (Omicorn) | 2022-09-06 20:30 | 2022/9/6 | 2022-09- 19 9:00 | 13 |
| BA.5 | 295585 | male | 52 | Asymptomatic | BA.5.2 (Omicorn) | 2022-09-06 22:04 | 2022/9/6 | 2022-09- 19 10:12 | 13 |
| BA.5 | 295586 | male | 56 | Mild | BA.5.2 (Omicorn) | 2022-09-06 20:17 | 2022/9/6 | 2022-09- 18 15:44 | 12 |
| BA.5 | 295588 | female | 5 | Asymptomatic | BA.5.2 (Omicorn) | 2022-09-06 21:12 | 2022/9/6 | 2022-09- 16 11:00 | 10 |
| BA.5 | 295589 | female | 8 | Asymptomatic | BA.5.2 (Omicorn) | 2022-09-06 21:57 | 2022/9/6 | 2022-09- 19 9:30 | 12 |
| BA.5 | 295590 | male | 72 | Mild | BA.5.2 (Omicorn) | 2022-09-06 21:51 | 2022/9/6 | 2022-09- 19 9:00 | 12 |
| BA.5 | 295592 | female | 53 | Mild | BA.5.2 (Omicorn) | 2022-09-06 21:31 | 2022/9/5 | 2022-09- 18 12:27 | 12 |
| BA.5 | 295593 | male | 30 | Moderate | BA.5.2 (Omicorn) | 2022-09-06 21:31 | 2022/9/6 | 2022-09-22 0:00 | 15 |
| BA.5 | 295595 | male | 57 | Asymptomatic | BA.5.2 (Omicorn) | 2022-09-06 21:34 | 2022/9/5 | 2022-09- 19 10:00 | 13 |
| BA.5 | 295596 | male | 31 | Mild | BA.5.2 (Omicorn) | 2022-09-06 21:35 | 2022/9/6 | 2022-09-21 10:00 | 15 |
| BA.5 | 295597 | female | 51 | Mild | BA.5.2 (Omicorn) | 2022-09-06 21:34 | 2022/9/6 | 2022-09-26 0:00 | 19 |
| BA.5 | 295598 | male | 57 | Mild | BA.5.2 (Omicorn) | 2022-09-06 21:37 | 2022/9/6 | 2022-09-20 10:00 | 14 |
| BA.5 | 295599 | male | 54 | Mild | BA.5.2 (Omicorn) | 2022-09-06 21:36 | 2022/9/5 | 2022-09- 19 10:00 | 13 |
| BA.5 | 295600 | male | 36 | Mild | BA.5.2 (Omicorn) | 2022-09-06 21:39 | 2022/9/6 | 2022-09- 19 10:00 | 13 |
| BA.5 | 295601 | female | 21 | Moderate | BA.5.2 (Omicorn) | 2022-09-06 21:39 | 2022/9/6 | 2022-09- 19 21:39 | 13 |
| BA.5 | 295603 | male | 11 | Asymptomatic | BA.5.2 (Omicorn) | 2022-09-06 21:47 | 2022/9/6 | 2022-09- 18 12:31 | 12 |

| BA.5 | 295607 | male | 52 | Asymptomatic | BA.5.2 (Omicorn) | 2022-09-07 1:02 | 2022/9/6 | 2022-09- 19 10:00 | 12 |
| --- | --- | --- | --- | --- | --- | --- | --- | --- | --- |
| BA.5 | 295608 | male | 14 | Mild | BA.5.2 (Omicorn) | 2022-09-07 0:52 | 2022/9/6 | 2022-09-20 8:26 | 13 |
| BA.5 | 295610 | female | 53 | Mild | BA.5.2 (Omicorn) | 2022-09-07 0:53 | 2022/9/6 | 2022-09- 19 9:00 | 12 |
| BA.5 | 295612 | female | 5 | Mild | BA.5.2 (Omicorn) | 2022-09-07 0:08 | 2022/9/6 | 2022-09- 13 10:00 | 6 |
| BA.5 | 295614 | male | 31 | Mild | BA.5.2 (Omicorn) | 2022-09-07 0:10 | 2022/9/6 | 2022-09-20 8:52 | 13 |
| BA.5 | 295619 | male | 28 | Mild | BA.5.2 (Omicorn) | 2022-09-07 1:00 | 2022/9/6 | 2022-09-21 10:00 | 14 |
| BA.5 | 295622 | male | 50 | Asymptomatic | BA.5.2 (Omicorn) | 2022-09-07 2:44 | 2022/9/6 | 2022-09-20 9:26 | 13 |
| BA.5 | 295636 | male | 60 | Asymptomatic | BA.5.2 (Omicorn) | 2022-09-07 9:05 | 2022/9/6 | 2022-09-20 10:21 | 13 |
| BA.5 | 295655 | male | 18 | Mild | BA.5.2 (Omicorn) | 2022-09-07 15:02 | 2022/9/6 | 2022-09-22 9:51 | 15 |
| BA.5 | 295656 | male | 38 | Mild | BA.5.2 (Omicorn) | 2022-09-07 22:45 | 2022/9/6 | 2022-09-23 0:00 | 15 |
| BA.5 | 295664 | male | 10 | Mild | BA.5.2 (Omicorn) | 2022-09-07 12:23 | 2022/9/7 | 2022-09-26 0:00 | 18 |
| BA.5 | 295676 | female | 69 | Asymptomatic | BA.5.2 (Omicorn) | 2022-09-07 13:58 | 2022/9/6 | 2022-09-25 0:00 | 17 |
| BA.5 | 295677 | male | 20 | Asymptomatic | BA.5.2 (Omicorn) | 2022-09-07 21:45 | 2022/9/7 | 2022-09-22 0:00 | 14 |
| BA.5 | 295681 | female | 16 | Asymptomatic | BA.5.2 (Omicorn) | 2022-09-07 15:02 | 2022/9/7 | 2022-09-27 0:00 | 19 |
| BA.5 | 295696 | male | 59 | Mild | BA.5.2 (Omicorn) | 2022-09-07 18:12 | 2022/9/7 | 2022-09- 18 12:42 | 11 |
| BA.5 | 295702 | female | 3 | Mild | BA.5.2 (Omicorn) | 2022-09-08 6:58 | 2022/9/6 | 2022-09- 18 12:34 | 10 |
| BA.5 | 295704 | male | 33 | Asymptomatic | BA.5.2 (Omicorn) | 2022-09-07 22:45 | 2022/9/7 | 2022-09-21 10:00 | 13 |
| BA.5 | 295705 | female | 27 | Mild | BA.5.2 (Omicorn) | 2022-09-07 21:28 | 2022/9/7 | 2022-09-20 11:00 | 13 |
| BA.5 | 295706 | male | 53 | Mild | BA.5.2 (Omicorn) | 2022-09-07 21:33 | 2022/9/7 | 2022-09-23 0:00 | 15 |
| BA.5 | 295708 | female | 27 | Mild | BA.5.2 (Omicorn) | 2022-09-07 21:31 | 2022/9/7 | 2022-09- 17 10:00 | 10 |
| BA.5 | 295709 | male | 32 | Asymptomatic | BA.5.2 (Omicorn) | 2022-09-07 23:06 | 2022/9/7 | 2022-09-22 10:00 | 14 |
| BA.5 | 295710 | female | 6 | Mild | BA.5.2 (Omicorn) | 2022-09-07 23:06 | 2022/9/7 | 2022-09- 17 9:00 | 9 |
| BA.5 | 295711 | male | 44 | Mild | BA.5.2 (Omicorn) | 2022-09-07 23:04 | 2022/9/7 | 2022-09-23 0:00 | 15 |
| BA.5 | 295712 | male | 35 | Asymptomatic | BA.5.2 (Omicorn) | 2022-09-07 23:43 | 2022/9/7 | 2022-09-23 0:00 | 15 |
| BA.5 | 295713 | male | 54 | Mild | BA.5.2 (Omicorn) | 2022-09-07 23:44 | 2022/9/7 | 2022-09-22 10:00 | 14 |
| BA.5 | 295714 | female | 44 | Asymptomatic | BA.5.2 (Omicorn) | 2022-09-07 23:46 | 2022/9/7 | 2022-09-21 12:00 | 14 |
| BA.5 | 295717 | male | 33 | Mild | BA.5.2 (Omicorn) | 2022-09-07 23:53 | 2022/9/7 | 2022-09- 18 10:00 | 10 |

| BA.5 | 295719 | female | 38 | Asymptomatic | BA.5.2 (Omicorn) | 2022-09-07 23:11 | 2022/9/7 | 2022-09-25 0:00 | 17 |
| --- | --- | --- | --- | --- | --- | --- | --- | --- | --- |
| BA.5 | 295721 | female | 58 | Mild | BA.5.2 (Omicorn) | 2022-09-07 23:42 | 2022/9/6 | 2022-09-23 0:00 | 15 |
| BA.5 | 295725 | male | 34 | Mild | BA.5.2 (Omicorn) | 2022-09-08 0:38 | 2022/9/8 | 2022-09- 16 9:00 | 8 |
| BA.5 | 295728 | male | 22 | Mild | BA.5.2 (Omicorn) | 2022-09-08 0:40 | 2022/9/7 | 2022-09-22 10:00 | 14 |
| BA.5 | 295729 | female | 3 | Mild | BA.5.2 (Omicorn) | 2022-09-08 0:22 | 2022/9/8 | 2022-09- 15 10:00 | 7 |
| BA.5 | 295730 | male | 35 | Asymptomatic | BA.5.2 (Omicorn) | 2022-09-08 0:44 | 2022/9/7 | 2022-09-21 10:00 | 13 |
| BA.5 | 295731 | male | 10 | Mild | BA.5.2 (Omicorn) | 2022-09-08 0:43 | 2022/9/7 | 2022-09-21 10:00 | 13 |
| BA.5 | 295733 | female | 34 | Asymptomatic | BA.5.2 (Omicorn) | 2022-09-08 2:56 | 2022/9/8 | 2022-09-26 0:00 | 18 |
| BA.5 | 295735 | female | 60 | Asymptomatic | BA.5.2 (Omicorn) | 2022-09-08 3:09 | 2022/9/7 | 2022-09-23 0:00 | 15 |
| BA.5 | 295736 | female | 43 | Moderate | BA.5.2 (Omicorn) | 2022-09-08 3:07 | 2022/9/7 | 2022-09-20 10:00 | 12 |
| BA.5 | 295737 | female | 52 | Mild | BA.5.2 (Omicorn) | 2022-09-08 3:53 | 2022/9/7 | 2022-09-26 0:00 | 18 |
| BA.5 | 295738 | male | 7 | Mild | BA.5.2 (Omicorn) | 2022-09-08 3:50 | 2022/9/7 | 2022-09-20 10:00 | 12 |
| BA.5 | 295739 | male | 1 | Asymptomatic | BA.5.2 (Omicorn) | 2022-09-08 3:52 | 2022/9/7 | 2022-09-26 0:00 | 18 |
| BA.5 | 295743 | female | 37 | Mild | BA.5.2 (Omicorn) | 2022-09-08 3:07 | 2022/9/7 | 2022-09-22 3:07 | 14 |
| BA.5 | 295745 | female | 36 | Mild | BA.5.2 (Omicorn) | 2022-09-08 5:59 | 2022/9/7 | 2022-09-22 10:00 | 14 |
| BA.5 | 295746 | female | 38 | Asymptomatic | BA.5.2 (Omicorn) | 2022-09-08 5:30 | 2022/9/7 | 2022-09- 18 12:08 | 10 |
| BA.5 | 295748 | male | 53 | Asymptomatic | BA.5.2 (Omicorn) | 2022-09-08 4:52 | 2022/9/8 | 2022-09- 19 9:30 | 11 |
| BA.5 | 295749 | female | 37 | Asymptomatic | BA.5.2 (Omicorn) | 2022-09-08 8:50 | 2022/9/7 | 2022-09-25 0:00 | 17 |
| BA.5 | 295753 | male | 21 | Mild | BA.5.2 (Omicorn) | 2022-09-08 9:06 | 2022/9/7 | 2022-09-21 10:00 | 13 |
| BA.5 | 295767 | female | 57 | Asymptomatic | BA.5.2 (Omicorn) | 2022-09-08 11:14 | 2022/9/7 | 2022-09-24 0:00 | 16 |
| BA.5 | 295768 | female | 9 | Asymptomatic | BA.5.2 (Omicorn) | 2022-09-08 11:13 | 2022/9/7 | 2022-09-20 11:00 | 12 |
| BA.5 | 295770 | male | 49 | Asymptomatic | BA.5.2 (Omicorn) | 2022-09-08 11:12 | 2022/9/7 | 2022-09-21 10:00 | 13 |
| BA.5 | 295778 | female | 32 | Mild | BA.5.2 (Omicorn) | 2022-09-08 10:40 | 2022/9/7 | 2022-09-26 0:00 | 18 |
| BA.5 | 295779 | male | 3 | Mild | BA.5.2 (Omicorn) | 2022-09-08 10:41 | 2022/9/7 | 2022-09-26 0:00 | 18 |
| BA.5 | 295782 | male | 8 | Mild | BA.5.2 (Omicorn) | 2022-09-08 12:19 | 2022/9/8 | 2022-09- 19 9:30 | 11 |
| BA.5 | 295786 | female | 56 | Asymptomatic | BA.5.2 (Omicorn) | 2022-09-08 11:13 | 2022/9/8 | 2022-09-20 8:29 | 12 |
| BA.5 | 295787 | female | 33 | Asymptomatic | BA.5.2 (Omicorn) | 2022-09-08 11:16 | 2022/9/7 | 2022-09- 19 9:38 | 11 |

| BA.5 | 295790 | female | 8 | Asymptomatic | BA.5.2 (Omicorn) | 2022-09-08 11:42 | 2022/9/8 | 2022-09-27 0:00 | 19 |
| --- | --- | --- | --- | --- | --- | --- | --- | --- | --- |
| BA.5 | 295791 | male | 50 | Mild | BA.5.2 (Omicorn) | 2022-09-08 11:45 | 2022/9/7 | 2022-09-27 0:00 | 19 |
| BA.5 | 295797 | male | 41 | Mild | BA.5.2 (Omicorn) | 2022-09-08 13:55 | 2022/9/8 | 2022-09-20 10:17 | 12 |
| BA.5 | 295817 | male | 30 | Mild | BA.5.2 (Omicorn) | 2022-09-08 17:22 | 2022/9/8 | 2022-09- 18 10:00 | 10 |
| BA.5 | 295818 | female | 5 | Mild | BA.5.2 (Omicorn) | 2022-09-08 17:30 | 2022/9/8 | 2022-09-24 0:00 | 15 |
| BA.5 | 295832 | female | 3 | Mild | BA.5.6 (Omicorn) | 2022-09-08 21:33 | 2022/9/8 | 2022-09-25 0:00 | 16 |

| **Variants** | **ID** | **Vaccination** | **vaccine** **doses** | **Type** **of** **vaccine** **(1)** | **Type** **of** **vaccine** **(2)** | **Type** **of** **vaccine** **(3)** | **Days** **between** **the**  **last** **dose** **and**  **the** **onset** **of** **illness** |
| --- | --- | --- | --- | --- | --- | --- | --- |
| BA.5 | 69911 | Boosted | 3 | Inactivated vaccines | Inactivated vaccines | Inactivated vaccines | 125 |
| BA.5 | 287655 | Boosted | 3 | Inactivated vaccines | Inactivated vaccines | Inactivated vaccines | 286 |
| BA.5 | 288461 | Fully vaccinated | 2 | mRNA | mRNA |  | 395 |
| BA.5 | 288530 | Boosted | 3 | Inactivated vaccines | Inactivated vaccines | Inactivated vaccines | 166 |
| BA.5 | 288532 | Boosted | 3 | Inactivated vaccines | Inactivated vaccines | mRNA | 163 |
| BA.5 | 288572 | Boosted | 3 | Inactivated vaccines | Inactivated vaccines | Inactivated vaccines | 147 |
| BA.5 | 288700 | Unvaccinated | 1 | Inactivated vaccines |  |  |  |
| BA.5 | 288707 | Boosted | 3 | Inactivated vaccines | Inactivated vaccines | mRNA | 81 |
| BA.5 | 288822 | Fully vaccinated | 2 | Inactivated vaccines | Inactivated vaccines |  | 337 |
| BA.5 | 289508 | Boosted | 3 | Inactivated vaccines | mRNA | mRNA | 164 |
| BA.5 | 289528 | Fully vaccinated | 2 | mRNA | mRNA |  | 300 |
| BA.5 | 289545 | Boosted | 3 | Inactivated vaccines | Inactivated vaccines | Inactivated vaccines | 80 |
| BA.5 | 289771 | Boosted | 3 | Inactivated vaccines | Inactivated vaccines | mRNA | 42 |
| BA.5 | 289949 | Boosted | 3 | mRNA | mRNA | mRNA | 129 |
| BA.5 | 290447 | Boosted | 3 | Inactivated vaccines | Inactivated vaccines | Inactivated vaccines | 202 |
| BA.5 | 290449 | Boosted | 3 | Inactivated vaccines | Inactivated vaccines | Inactivated vaccines | 199 |
| BA.5 | 290451 | Boosted | 3 | Unknown | Unknown | Unknown |  |
| BA.5 | 290452 | Boosted | 3 | Inactivated vaccines | Inactivated vaccines | Inactivated vaccines | 172 |
| BA.5 | 290761 | Fully vaccinated | 2 | mRNA | mRNA |  | 121 |
| BA.5 | 290955 | Boosted | 3 | Inactivated vaccines | Inactivated vaccines | Inactivated vaccines | 164 |
| BA.5 | 290970 | Unvaccinated | 0 |  |  |  |  |
| BA.5 | 291064 | Fully vaccinated | 2 | mRNA | mRNA |  | 358 |
| BA.5 | 291075 | Boosted | 3 | mRNA | mRNA | mRNA | 65 |
| BA.5 | 291091 | Boosted | 3 | Inactivated vaccines | Inactivated vaccines | mRNA | 41 |
| BA.5 | 291207 | Boosted | 3 | Inactivated vaccines | Inactivated vaccines | Inactivated vaccines | 124 |
| BA.5 | 291285 | Fully vaccinated | 2 | Inactivated vaccines | Inactivated vaccines |  | 498 |
| BA.5 | 291286 | Unvaccinated | 0 |  |  |  |  |
| BA.5 | 291289 | Boosted | 4 | Inactivated vaccines | Inactivated vaccines A | denovirus vector vaccine | 89 |
| BA.5 | 291398 | Fully vaccinated | 1 | Adenovirus-vectored vaccine |  |  |  |
| BA.5 | 291444 | Boosted | 3 | Inactivated vaccines | Inactivated vaccines | Inactivated vaccines | 204 |
| BA.5 | 291446 | Boosted | 4 | Inactivated vaccines | Inactivated vaccines A | denovirus vector vaccine | 204 |
| BA.5 | 291494 | Boosted | 4 | Inactivated vaccines | Inactivated vaccines | Inactivated vaccines | 382 |
| BA.5 | 291495 | Boosted | 3 | Inactivated vaccines | Inactivated vaccines | Inactivated vaccines | 192 |

| BA.5 | 291500 | Boosted | 3 | Inactivated vaccines | Inactivated vaccines | Inactivated vaccines | 241 |
| --- | --- | --- | --- | --- | --- | --- | --- |
| BA.5 | 291501 | Boosted | 3 | Inactivated vaccines | Inactivated vaccines | mRNA | 72 |
| BA.5 | 291502 | Boosted | 3 | Inactivated vaccines | Inactivated vaccines | Inactivated vaccines | 260 |
| BA.5 | 291660 | Fully vaccinated | 2 | mRNA | mRNA |  | 382 |
| BA.5 | 291662 | Boosted | 3 | mRNA | mRNA | mRNA | 228 |
| BA.5 | 291696 | Fully vaccinated | 2 | mRNA | mRNA |  | 41 |
| BA.5 | 291753 | Boosted | 3 | Inactivated vaccines | Inactivated vaccines | mRNA | 300 |
| BA.5 | 291811 | Boosted | 3 | Inactivated vaccines | Inactivated vaccines | Inactivated vaccines | 261 |
| BA.5 | 291860 | Boosted | 3 | mRNA | mRNA | mRNA | 72 |
| BA.5 | 291867 | Boosted | 3 | mRNA | mRNA | mRNA | 130 |
| BA.5 | 291918 | Boosted | 3 | Inactivated vaccines | Inactivated vaccines | Inactivated vaccines | 325 |
| BA.5 | 292048 | Boosted | 3 | Inactivated vaccines | Inactivated vaccines | Inactivated vaccines | 192 |
| BA.5 | 292049 | Fully vaccinated | 2 | mRNA | mRNA |  | 227 |
| BA.5 | 292064 | Fully vaccinated | 2 | Inactivated vaccines | Inactivated vaccines |  | 413 |
| BA.5 | 292065 | Boosted | 3 | Inactivated vaccines | Inactivated vaccines | Inactivated vaccines | 262 |
| BA.5 | 292066 | Boosted | 4 | Adenovirus-vectored vaccine | Adenovirus vector vaccine A | denovirus vector vaccine | 227 |
| BA.5 | 292067 | Boosted | 3 | mRNA | mRNA | mRNA | 168 |
| BA.5 | 292069 | Unvaccinated | 1 | Unknown |  |  |  |
| BA.5 | 292073 | Fully vaccinated | 2 | Inactivated vaccines | Inactivated vaccines |  | 404 |
| BA.5 | 292078 | Unvaccinated | 0 |  |  |  |  |
| BA.5 | 292124 | Boosted | 3 | Inactivated vaccines | Inactivated vaccines | Inactivated vaccines | 316 |
| BA.5 | 292181 | Fully vaccinated | 2 | Inactivated vaccines | Inactivated vaccines |  | 470 |
| BA.5 | 292184 | Fully vaccinated | 2 | Inactivated vaccines | Inactivated vaccines |  | 473 |
| BA.5 | 292212 | Fully vaccinated | 2 | mRNA | mRNA |  | 415 |
| BA.5 | 292222 | Boosted | 4 | Inactivated vaccines | Inactivated vaccines | Inactivated vaccines | 37 |
| BA.5 | 292406 | Boosted | 3 | mRNA | mRNA | mRNA | 181 |
| BA.5 | 292419 | Fully vaccinated | 2 | Unknown | Unknown |  |  |
| BA.5 | 292421 | Fully vaccinated | 2 | mRNA | mRNA |  | 310 |
| BA.5 | 292615 | Boosted | 3 | Inactivated vaccines | Inactivated vaccines | Inactivated vaccines | 177 |
| BA.5 | 292633 | Boosted | 5 | Inactivated vaccines | Inactivated vaccines | Inactivated vaccines | 40 |
| BA.5 | 292653 | Fully vaccinated | 2 | mRNA | mRNA |  | 176 |
| BA.5 | 293347 | Boosted | 3 | Inactivated vaccines | Inactivated vaccines | Inactivated vaccines | 217 |
| BA.5 | 293703 | Boosted | 3 | Inactivated vaccines | Inactivated vaccines | Inactivated vaccines | 338 |
| BA.5 | 293746 | Boosted | 3 | Inactivated vaccines | Inactivated vaccines | Inactivated vaccines | 146 |
| BA.5 | 293760 | Boosted | 3 | Inactivated vaccines | Inactivated vaccines | Inactivated vaccines | 14 |
| BA.5 | 293777 | Boosted | 3 | Inactivated vaccines | Inactivated vaccines | Inactivated vaccines | 266 |

| BA.5 | 293780 | Boosted | 3 | Inactivated vaccines | Inactivated vaccines | Inactivated vaccines | 290 |
| --- | --- | --- | --- | --- | --- | --- | --- |
| BA.5 | 293795 | Boosted | 3 | Inactivated vaccines | Inactivated vaccines | Inactivated vaccines | 333 |
| BA.5 | 293868 | Boosted | 3 | Inactivated vaccines | Inactivated vaccines | Inactivated vaccines | 211 |
| BA.5 | 293920 | Boosted | 3 | Inactivated vaccines | Inactivated vaccines | Inactivated vaccines | 294 |
| BA.5 | 293961 | Fully vaccinated | 2 | Inactivated vaccines | Inactivated vaccines |  | 273 |
| BA.5 | 293986 | Boosted | 3 | Inactivated vaccines | Inactivated vaccines | Inactivated vaccines | 218 |
| BA.5 | 293988 | Boosted | 3 | Inactivated vaccines | Inactivated vaccines | Inactivated vaccines | 211 |
| BA.5 | 293996 | Boosted | 3 | mRNA | mRNA | mRNA | 225 |
| BA.5 | 294005 | Fully vaccinated | 2 | Inactivated vaccines | Inactivated vaccines |  | 134 |
| BA.5 | 294019 | Boosted | 3 | Inactivated vaccines | Inactivated vaccines | Inactivated vaccines | 298 |
| BA.5 | 294052 | Boosted | 3 | Inactivated vaccines | Inactivated vaccines | Inactivated vaccines | 227 |
| BA.5 | 294076 | Boosted | 3 | Inactivated vaccines | Inactivated vaccines | Inactivated vaccines | 230 |
| BA.5 | 294089 | Boosted | 3 | Inactivated vaccines | Inactivated vaccines | Inactivated vaccines | 136 |
| BA.5 | 294090 | Boosted | 3 | Inactivated vaccines | Inactivated vaccines | Inactivated vaccines | 136 |
| BA.5 | 294091 | Boosted | 3 | Inactivated vaccines | Inactivated vaccines | Inactivated vaccines | 130 |
| BA.5 | 294099 | Fully vaccinated | 2 | Inactivated vaccines | Inactivated vaccines |  | 373 |
| BA.5 | 294100 | Boosted | 3 | Inactivated vaccines | Inactivated vaccines | Inactivated vaccines | 121 |
| BA.5 | 294101 | Boosted | 3 | Inactivated vaccines | Inactivated vaccines | Inactivated vaccines | 128 |
| BA.5 | 294115 | Boosted | 3 | Inactivated vaccines | Inactivated vaccines | Inactivated vaccines | 323 |
| BA.5 | 294137 | Fully vaccinated | 2 | Inactivated vaccines | Inactivated vaccines |  | 338 |
| BA.5 | 294141 | Fully vaccinated | 2 | Inactivated vaccines | Inactivated vaccines |  | 343 |
| BA.5 | 294167 | Boosted | 3 | Inactivated vaccines | Inactivated vaccines | Inactivated vaccines | 271 |
| BA.5 | 294186 | Boosted | 3 | Inactivated vaccines | Inactivated vaccines | Inactivated vaccines | 186 |
| BA.5 | 294199 | Boosted | 3 | Inactivated vaccines | Inactivated vaccines | Inactivated vaccines | 264 |
| BA.5 | 294200 | Boosted | 3 | Inactivated vaccines | Inactivated vaccines | Inactivated vaccines | 243 |
| BA.5 | 294203 | Fully vaccinated | 2 | Inactivated vaccines | Inactivated vaccines |  | 503 |
| BA.5 | 294204 | Boosted | 3 | Inactivated vaccines | Inactivated vaccines | Inactivated vaccines | 249 |
| BA.5 | 294214 | Boosted | 3 | Inactivated vaccines | Inactivated vaccines | Inactivated vaccines |  |
| BA.5 | 294244 | Boosted | 3 | Inactivated vaccines | Inactivated vaccines | Inactivated vaccines | 265 |
| BA.5 | 294249 | Fully vaccinated | 2 | Inactivated vaccines | Inactivated vaccines |  | 143 |
| BA.5 | 294250 | Boosted | 3 | Inactivated vaccines | Inactivated vaccines | Inactivated vaccines | 283 |
| BA.5 | 294251 | Fully vaccinated | 2 | Inactivated vaccines | Inactivated vaccines |  | 357 |
| BA.5 | 294295 | Fully vaccinated | 2 | Inactivated vaccines | Inactivated vaccines |  |  |
| BA.5 | 294296 | Boosted | 3 | Inactivated vaccines | Inactivated vaccines | Inactivated vaccines | 140 |
| BA.5 | 294297 | Unvaccinated | 0 |  |  |  |  |
| BA.5 | 294298 | Boosted | 3 | Inactivated vaccines | Inactivated vaccines | Inactivated vaccines |  |

| BA.5 | 294299 | Boosted | 3 | Inactivated vaccines | Inactivated vaccines | Inactivated vaccines | 269 |
| --- | --- | --- | --- | --- | --- | --- | --- |
| BA.5 | 294301 | Unvaccinated | 0 |  |  |  |  |
| BA.5 | 294302 | Boosted | 3 | Inactivated vaccines | Inactivated vaccines | Inactivated vaccines | 198 |
| BA.5 | 294303 | Fully vaccinated | 2 | Inactivated vaccines | Inactivated vaccines |  | 359 |
| BA.5 | 294304 | Boosted | 3 | Inactivated vaccines | Inactivated vaccines | Inactivated vaccines | 255 |
| BA.5 | 294305 | Boosted | 3 | Inactivated vaccines | Inactivated vaccines | Inactivated vaccines | 306 |
| BA.5 | 294312 | Boosted | 3 | Inactivated vaccines | Inactivated vaccines | Inactivated vaccines | 290 |
| BA.5 | 294319 | Boosted | 3 | Inactivated vaccines | Inactivated vaccines | Inactivated vaccines | 290 |
| BA.5 | 294322 | Boosted | 3 | Inactivated vaccines | Inactivated vaccines | Inactivated vaccines | 279 |
| BA.5 | 294323 | Unknown | Unknown |  |  |  |  |
| BA.5 | 294332 | Boosted | 3 | Inactivated vaccines | Inactivated vaccines | Inactivated vaccines | 147 |
| BA.5 | 294334 | Fully vaccinated | 2 | Inactivated vaccines | Inactivated vaccines |  | 354 |
| BA.5 | 294335 | Boosted | 3 | Inactivated vaccines | Inactivated vaccines | Inactivated vaccines | 265 |
| BA.5 | 294339 | Boosted | 3 | Inactivated vaccines | Inactivated vaccines | Inactivated vaccines | 125 |
| BA.5 | 294340 | Boosted | 3 | Inactivated vaccines | Inactivated vaccines | Inactivated vaccines | 242 |
| BA.5 | 294347 | Boosted | 3 | Inactivated vaccines | Inactivated vaccines | Inactivated vaccines | 259 |
| BA.5 | 294368 | Boosted | 3 | Inactivated vaccines | Inactivated vaccines | Inactivated vaccines | 161 |
| BA.5 | 294369 | Boosted | 3 | Inactivated vaccines | Inactivated vaccines | Inactivated vaccines | 237 |
| BA.5 | 294378 | Boosted | 3 | Inactivated vaccines | Inactivated vaccines | Inactivated vaccines | 289 |
| BA.5 | 294383 | Boosted | 3 | Inactivated vaccines | Inactivated vaccines | Inactivated vaccines | 244 |
| BA.5 | 294384 | Fully vaccinated | 2 | Inactivated vaccines | Inactivated vaccines |  | 90 |
| BA.5 | 294385 | Boosted | 3 | Inactivated vaccines | Inactivated vaccines | Inactivated vaccines | 198 |
| BA.5 | 294386 | Boosted | 3 | Inactivated vaccines | Inactivated vaccines | Inactivated vaccines | 237 |
| BA.5 | 294390 | Boosted | 3 | Inactivated vaccines | Inactivated vaccines | Inactivated vaccines | 144 |
| BA.5 | 294392 | Fully vaccinated | 2 | Inactivated vaccines | Inactivated vaccines |  | 231 |
| BA.5 | 294393 | Boosted | 3 | Inactivated vaccines | Inactivated vaccines | Inactivated vaccines | 245 |
| BA.5 | 294397 | Unvaccinated | 0 |  |  |  |  |
| BA.5 | 294398 | Boosted | 3 | Inactivated vaccines | Inactivated vaccines | Inactivated vaccines | 237 |
| BA.5 | 294406 | Boosted | 3 | Inactivated vaccines | Inactivated vaccines | Inactivated vaccines | 243 |
| BA.5 | 294408 | Fully vaccinated | 2 | Inactivated vaccines | Inactivated vaccines |  | 364 |
| BA.5 | 294409 | Unknown | Unknown |  |  |  |  |
| BA.5 | 294410 | Boosted | 3 | Inactivated vaccines | Inactivated vaccines | Inactivated vaccines | 242 |
| BA.5 | 294411 | Unknown | Unknown |  |  |  |  |
| BA.5 | 294420 | Boosted | 3 | Inactivated vaccines | Inactivated vaccines | Inactivated vaccines | 126 |
| BA.5 | 294421 | Boosted | 3 | Inactivated vaccines | Inactivated vaccines | Inactivated vaccines | 143 |
| BA.5 | 294424 | Boosted | 3 | Inactivated vaccines | Inactivated vaccines | Inactivated vaccines | 255 |

| BA.5 | 294425 | Fully vaccinated | 2 | Inactivated vaccines | Inactivated vaccines |  | 350 |
| --- | --- | --- | --- | --- | --- | --- | --- |
| BA.5 | 294427 | Boosted | 3 | Inactivated vaccines | Inactivated vaccines | Inactivated vaccines | 233 |
| BA.5 | 294431 | Boosted | 3 | Inactivated vaccines | Inactivated vaccines | Inactivated vaccines | 216 |
| BA.5 | 294441 | Unvaccinated | 0 |  |  |  |  |
| BA.5 | 294442 | Boosted | 3 | Inactivated vaccines | Inactivated vaccines | Inactivated vaccines | 247 |
| BA.5 | 294443 | Unknown | Unknown |  |  |  |  |
| BA.5 | 294444 | Fully vaccinated | 2 | Inactivated vaccines | Inactivated vaccines |  | 457 |
| BA.5 | 294445 | Unvaccinated | 0 |  |  |  |  |
| BA.5 | 294447 | Boosted | 3 | Inactivated vaccines | Inactivated vaccines | Inactivated vaccines | 148 |
| BA.5 | 294449 | Boosted | 3 | Inactivated vaccines | Inactivated vaccines | Inactivated vaccines | 247 |
| BA.5 | 294450 | Unvaccinated | 0 |  |  |  |  |
| BA.5 | 294474 | Boosted | 3 | Inactivated vaccines | Inactivated vaccines | Inactivated vaccines | 147 |
| BA.5 | 294476 | Boosted | 3 | Inactivated vaccines | Inactivated vaccines | Inactivated vaccines | 252 |
| BA.5 | 294485 | Boosted | 3 | Inactivated vaccines | Inactivated vaccines | Inactivated vaccines | 172 |
| BA.5 | 294492 | Boosted | 3 | Inactivated vaccines | Inactivated vaccines | Inactivated vaccines | 247 |
| BA.5 | 294495 | Boosted | 3 | Inactivated vaccines | Inactivated vaccines | Inactivated vaccines | 343 |
| BA.5 | 294497 | Boosted | 3 | Inactivated vaccines | Inactivated vaccines | Inactivated vaccines | 245 |
| BA.5 | 294506 | Boosted | 3 | Inactivated vaccines | Inactivated vaccines | Inactivated vaccines | 226 |
| BA.5 | 294510 | Boosted | 3 | Inactivated vaccines | Inactivated vaccines | Inactivated vaccines | 168 |
| BA.5 | 294519 | Boosted | 3 | Inactivated vaccines | Inactivated vaccines | Inactivated vaccines | 125 |
| BA.5 | 294526 | Boosted | 3 | Inactivated vaccines | Inactivated vaccines | Inactivated vaccines | 200 |
| BA.5 | 294527 | Boosted | 3 | Inactivated vaccines | Inactivated vaccines | Inactivated vaccines | 240 |
| BA.5 | 294528 | Fully vaccinated | 2 | Unknown | Unknown |  |  |
| BA.5 | 294534 | Boosted | 3 | Inactivated vaccines | Inactivated vaccines | Inactivated vaccines | 244 |
| BA.5 | 294535 | Boosted | 3 | Inactivated vaccines | Inactivated vaccines | Inactivated vaccines | 258 |
| BA.5 | 294537 | Boosted | 3 | Inactivated vaccines | Inactivated vaccines | Inactivated vaccines | 122 |
| BA.5 | 294540 | Fully vaccinated | 2 | Inactivated vaccines | Inactivated vaccines |  | 276 |
| BA.5 | 294546 | Unvaccinated | 1 | Inactivated vaccines |  |  |  |
| BA.5 | 294548 | Boosted | 3 | Inactivated vaccines | Inactivated vaccines | Inactivated vaccines | 266 |
| BA.5 | 294554 | Boosted | 3 | Inactivated vaccines | Inactivated vaccines | Inactivated vaccines | 340 |
| BA.5 | 294558 | Boosted | 3 | Inactivated vaccines | Inactivated vaccines | Inactivated vaccines | 133 |
| BA.5 | 294559 | Unknown | Unknown |  |  |  |  |
| BA.5 | 294562 | Boosted | 3 | Unknown | Unknown | Unknown |  |
| BA.5 | 294563 | Unknown | Unknown |  |  |  |  |
| BA.5 | 294565 | Unknown | Unknown |  |  |  |  |
| BA.5 | 294566 | Boosted | 3 | Inactivated vaccines | Inactivated vaccines | Inactivated vaccines | 220 |

| BA.5 | 294575 | Boosted | 3 | Inactivated vaccines | Inactivated vaccines | Inactivated vaccines | 129 |
| --- | --- | --- | --- | --- | --- | --- | --- |
| BA.5 | 294577 | Boosted | 3 | Inactivated vaccines | Inactivated vaccines | Inactivated vaccines | 277 |
| BA.5 | 294578 | Boosted | 3 | Inactivated vaccines | Inactivated vaccines | Inactivated vaccines | 155 |
| BA.5 | 294579 | Fully vaccinated | 2 | Inactivated vaccines | Inactivated vaccines |  | 296 |
| BA.5 | 294580 | Boosted | 3 | Inactivated vaccines | Inactivated vaccines | Inactivated vaccines | 272 |
| BA.5 | 294581 | Boosted | 3 | Inactivated vaccines | Inactivated vaccines | Inactivated vaccines | 248 |
| BA.5 | 294582 | Boosted | 3 | Inactivated vaccines | Inactivated vaccines | Inactivated vaccines | 237 |
| BA.5 | 294584 | Fully vaccinated | 2 | Inactivated vaccines | Inactivated vaccines |  | 427 |
| BA.5 | 294585 | Boosted | 3 | Inactivated vaccines | Inactivated vaccines | Unknown |  |
| BA.5 | 294587 | Boosted | 3 | Inactivated vaccines | Inactivated vaccines | Inactivated vaccines | 112 |
| BA.5 | 294589 | Unknown | Unknown |  |  |  |  |
| BA.5 | 294590 | Boosted | 3 | Inactivated vaccines | Inactivated vaccines | Inactivated vaccines | 294 |
| BA.5 | 294593 | Boosted | 3 | Inactivated vaccines | Inactivated vaccines | Inactivated vaccines | 276 |
| BA.5 | 294594 | Boosted | 3 | Inactivated vaccines | Inactivated vaccines | Inactivated vaccines | 254 |
| BA.5 | 294596 | Boosted | 3 | Inactivated vaccines | Inactivated vaccines | Inactivated vaccines | 256 |
| BA.5 | 294597 | Boosted | 3 | Inactivated vaccines | Inactivated vaccines | Inactivated vaccines | 253 |
| BA.5 | 294604 | Boosted | 3 | Inactivated vaccines | Inactivated vaccines | Inactivated vaccines | 256 |
| BA.5 | 294605 | Unknown | Unknown |  |  |  |  |
| BA.5 | 294619 | Fully vaccinated | 2 | Inactivated vaccines | Inactivated vaccines |  | 347 |
| BA.5 | 294621 | Unvaccinated | 1 | Inactivated vaccines |  |  |  |
| BA.5 | 294622 | Boosted | 3 | Inactivated vaccines | Inactivated vaccines | Inactivated vaccines | 272 |
| BA.5 | 294623 | Boosted | 3 | Inactivated vaccines | Inactivated vaccines | Inactivated vaccines | 137 |
| BA.5 | 294624 | Boosted | 3 | Unknown | Unknown | Unknown |  |
| BA.5 | 294625 | Boosted | 3 | Inactivated vaccines | Inactivated vaccines | Inactivated vaccines | 305 |
| BA.5 | 294626 | Fully vaccinated | 2 | Inactivated vaccines | Inactivated vaccines |  |  |
| BA.5 | 294627 | Boosted | 3 | Inactivated vaccines | Inactivated vaccines | Inactivated vaccines | 184 |
| BA.5 | 294641 | Boosted | 3 | Inactivated vaccines | Inactivated vaccines | Inactivated vaccines | 127 |
| BA.5 | 294651 | Boosted | 3 | Inactivated vaccines | Inactivated vaccines | Inactivated vaccines | 294 |
| BA.5 | 294652 | Boosted | 3 | Inactivated vaccines | Inactivated vaccines | Inactivated vaccines | 223 |
| BA.5 | 294653 | Unknown | Unknown |  |  |  |  |
| BA.5 | 294657 | Boosted | 3 | Inactivated vaccines | Inactivated vaccines | Inactivated vaccines | 255 |
| BA.5 | 294664 | Boosted | 3 | Inactivated vaccines | Inactivated vaccines | Inactivated vaccines | 154 |
| BA.5 | 294665 | Fully vaccinated | 2 | Inactivated vaccines | Inactivated vaccines |  | 300 |
| BA.5 | 294669 | Unknown | Unknown |  |  |  |  |
| BA.5 | 294674 | Fully vaccinated | 2 | Inactivated vaccines | Inactivated vaccines |  | 268 |
| BA.5 | 294677 | Boosted | 3 | Inactivated vaccines | Inactivated vaccines | Inactivated vaccines | 139 |

| BA.5 | 294678 | Boosted | 3 | Inactivated vaccines | Inactivated vaccines | Inactivated vaccines | 302 |
| --- | --- | --- | --- | --- | --- | --- | --- |
| BA.5 | 294681 | Boosted | 3 | Inactivated vaccines | Inactivated vaccines | Inactivated vaccines | 142 |
| BA.5 | 294682 | Boosted | 3 | Inactivated vaccines | Inactivated vaccines | Inactivated vaccines | 259 |
| BA.5 | 294684 | Boosted | 3 | Inactivated vaccines | Inactivated vaccines | Inactivated vaccines | 243 |
| BA.5 | 294685 | Unvaccinated | 1 | Inactivated vaccines |  |  |  |
| BA.5 | 294690 | Boosted | 3 | Inactivated vaccines | Inactivated vaccines | Inactivated vaccines | 277 |
| BA.5 | 294691 | Boosted | 3 | Inactivated vaccines | Inactivated vaccines | Inactivated vaccines | 245 |
| BA.5 | 294696 | Boosted | 3 | Inactivated vaccines | Inactivated vaccines | Inactivated vaccines | 259 |
| BA.5 | 294700 | Fully vaccinated | 2 | Inactivated vaccines | Inactivated vaccines |  | 362 |
| BA.5 | 294703 | Boosted | 3 | Unknown | Unknown | Unknown |  |
| BA.5 | 294719 | Boosted | 3 | Inactivated vaccines | Inactivated vaccines | Inactivated vaccines |  |
| BA.5 | 294726 | Fully vaccinated | 2 | Inactivated vaccines | Inactivated vaccines |  | 458 |
| BA.5 | 294727 | Boosted | 3 | Inactivated vaccines | Inactivated vaccines | Inactivated vaccines | 270 |
| BA.5 | 294728 | Boosted | 3 | Inactivated vaccines | Inactivated vaccines | Inactivated vaccines | 136 |
| BA.5 | 294730 | Boosted | 3 | Inactivated vaccines | Inactivated vaccines | Inactivated vaccines | 142 |
| BA.5 | 294734 | Boosted | 3 | Inactivated vaccines | Inactivated vaccines | Inactivated vaccines | 258 |
| BA.5 | 294736 | Boosted | 3 | Inactivated vaccines | Inactivated vaccines | Inactivated vaccines | 296 |
| BA.5 | 294738 | Fully vaccinated | 2 | Inactivated vaccines | Inactivated vaccines |  | 376 |
| BA.5 | 294739 | Boosted | 3 | Inactivated vaccines | Inactivated vaccines | Inactivated vaccines | 245 |
| BA.5 | 294740 | Boosted | 3 | Inactivated vaccines | Inactivated vaccines | Inactivated vaccines | 179 |
| BA.5 | 294741 | Boosted | 3 | Inactivated vaccines | Inactivated vaccines | Inactivated vaccines | 135 |
| BA.5 | 294742 | Boosted | 3 | Inactivated vaccines | Inactivated vaccines | Inactivated vaccines | 248 |
| BA.5 | 294743 | Unknown | Unknown |  |  |  |  |
| BA.5 | 294744 | Boosted | 3 | Inactivated vaccines | Inactivated vaccines | Inactivated vaccines | 131 |
| BA.5 | 294746 | Unvaccinated | 0 |  |  |  |  |
| BA.5 | 294747 | Boosted | 3 | Inactivated vaccines | Inactivated vaccines | Inactivated vaccines |  |
| BA.5 | 294749 | Unknown | Unknown |  |  |  |  |
| BA.5 | 294752 | Unknown | Unknown |  |  |  |  |
| BA.5 | 294758 | Unknown | Unknown |  |  |  |  |
| BA.5 | 294759 | Boosted | 3 | Inactivated vaccines | Inactivated vaccines | Inactivated vaccines | 249 |
| BA.5 | 294761 | Fully vaccinated | 2 | Inactivated vaccines | Inactivated vaccines |  | 277 |
| BA.5 | 294767 | Boosted | 3 | Inactivated vaccines | Inactivated vaccines | Inactivated vaccines | 149 |
| BA.5 | 294771 | Boosted | 3 | Inactivated vaccines | Inactivated vaccines | Inactivated vaccines | 253 |
| BA.5 | 294777 | Fully vaccinated | 2 | Inactivated vaccines | Inactivated vaccines |  | 355 |
| BA.5 | 294778 | Boosted | 5 | Unknown | Unknown | Unknown |  |
| BA.5 | 294783 | Fully vaccinated | 2 | Inactivated vaccines | Inactivated vaccines |  | 402 |

| BA.5 | 294792 | Boosted | 3 | Unknown | Unknown | Unknown |  |
| --- | --- | --- | --- | --- | --- | --- | --- |
| BA.5 | 294794 | Boosted | 3 | Inactivated vaccines | Inactivated vaccines | Inactivated vaccines | 259 |
| BA.5 | 294795 | Boosted | 3 | Inactivated vaccines | Inactivated vaccines | Inactivated vaccines | 293 |
| BA.5 | 294796 | Boosted | 3 | Inactivated vaccines | Inactivated vaccines | Inactivated vaccines | 239 |
| BA.5 | 294803 | Boosted | 3 | Inactivated vaccines | Inactivated vaccines | Inactivated vaccines | 141 |
| BA.5 | 294806 | Unvaccinated | 1 | Unknown |  |  |  |
| BA.5 | 294822 | Unknown | Unknown |  |  |  |  |
| BA.5 | 294825 | Unvaccinated | 0 |  |  |  |  |
| BA.5 | 294826 | Unvaccinated | 0 |  |  |  |  |
| BA.5 | 294831 | Boosted | 3 | Inactivated vaccines | Inactivated vaccines | Inactivated vaccines | 153 |
| BA.5 | 294838 | Unknown | Unknown |  |  |  |  |
| BA.5 | 294839 | Boosted | 3 | Inactivated vaccines | Inactivated vaccines | Inactivated vaccines |  |
| BA.5 | 294841 | Unvaccinated | 0 |  |  |  |  |
| BA.5 | 294842 | Fully vaccinated | 2 | Inactivated vaccines | Inactivated vaccines |  | 257 |
| BA.5 | 294845 | Boosted | 3 | Inactivated vaccines | Inactivated vaccines | Inactivated vaccines | 137 |
| BA.5 | 294846 | Boosted | 3 | Inactivated vaccines | Inactivated vaccines | Inactivated vaccines | 138 |
| BA.5 | 294847 | Boosted | 3 | Inactivated vaccines | Inactivated vaccines | Inactivated vaccines | 257 |
| BA.5 | 294848 | Unvaccinated | 0 |  |  |  |  |
| BA.5 | 294849 | Unknown | Unknown |  |  |  |  |
| BA.5 | 294850 | Boosted | 3 | Inactivated vaccines | Inactivated vaccines | Inactivated vaccines |  |
| BA.5 | 294852 | Fully vaccinated | 2 | Inactivated vaccines | Inactivated vaccines |  | 138 |
| BA.5 | 294868 | Boosted | 3 | Inactivated vaccines | Inactivated vaccines | Inactivated vaccines | 251 |
| BA.5 | 294875 | Boosted | 3 | Inactivated vaccines | Inactivated vaccines | Inactivated vaccines | 194 |
| BA.5 | 294876 | Boosted | 3 | Inactivated vaccines | Inactivated vaccines | Inactivated vaccines | 130 |
| BA.5 | 294880 | Boosted | 3 | Inactivated vaccines | Inactivated vaccines | Inactivated vaccines | 253 |
| BA.5 | 294882 | Boosted | 3 | Inactivated vaccines | Inactivated vaccines | Inactivated vaccines | 258 |
| BA.5 | 294884 | Boosted | 3 | Inactivated vaccines | Inactivated vaccines | Inactivated vaccines | 268 |
| BA.5 | 294885 | Boosted | 3 | Inactivated vaccines | Inactivated vaccines | Inactivated vaccines | 144 |
| BA.5 | 294886 | Boosted | 3 | Inactivated vaccines | Inactivated vaccines | Inactivated vaccines | 236 |
| BA.5 | 294888 | Boosted | 3 | Inactivated vaccines | Inactivated vaccines | Inactivated vaccines | 259 |
| BA.5 | 294889 | Boosted | 3 | Inactivated vaccines | Inactivated vaccines | Inactivated vaccines | 258 |
| BA.5 | 294890 | Boosted | 3 | Inactivated vaccines | Inactivated vaccines | Inactivated vaccines | 252 |
| BA.5 | 294891 | Boosted | 3 | Inactivated vaccines | Inactivated vaccines | Inactivated vaccines | 239 |
| BA.5 | 294892 | Unvaccinated | 0 |  |  |  |  |
| BA.5 | 294893 | Boosted | 3 | Inactivated vaccines | Inactivated vaccines | Inactivated vaccines | 286 |
| BA.5 | 294894 | Boosted | 3 | Inactivated vaccines | Inactivated vaccines | Inactivated vaccines | 257 |

| BA.5 | 294895 | Unvaccinated | 0 |  |  |  |  |
| --- | --- | --- | --- | --- | --- | --- | --- |
| BA.5 | 294896 | Boosted | 3 | Inactivated vaccines | Inactivated vaccines | Inactivated vaccines | 138 |
| BA.5 | 294898 | Boosted | 3 | Inactivated vaccines | Inactivated vaccines | Inactivated vaccines | 256 |
| BA.5 | 294900 | Fully vaccinated | 2 | Inactivated vaccines | Inactivated vaccines |  | 360 |
| BA.5 | 294901 | Boosted | 3 | Inactivated vaccines | Inactivated vaccines | Inactivated vaccines | 255 |
| BA.5 | 294902 | Unvaccinated | 1 | Inactivated vaccines |  |  |  |
| BA.5 | 294903 | Boosted | 3 | Inactivated vaccines | Inactivated vaccines | Inactivated vaccines | 256 |
| BA.5 | 294904 | Unknown | Unknown |  |  |  |  |
| BA.5 | 294905 | Boosted | 3 | Inactivated vaccines | Inactivated vaccines | Inactivated vaccines | 273 |
| BA.5 | 294906 | Boosted | 3 | Inactivated vaccines | Inactivated vaccines | Inactivated vaccines | 131 |
| BA.5 | 294907 | Fully vaccinated | 2 | Inactivated vaccines | Inactivated vaccines |  | 435 |
| BA.5 | 294908 | Boosted | 3 | Inactivated vaccines | Inactivated vaccines | Inactivated vaccines | 337 |
| BA.5 | 294909 | Boosted | 3 | Inactivated vaccines | Inactivated vaccines | Inactivated vaccines | 264 |
| BA.5 | 294916 | Fully vaccinated | 2 | Inactivated vaccines | Inactivated vaccines |  | 275 |
| BA.5 | 294917 | Fully vaccinated | 2 | Inactivated vaccines | Inactivated vaccines |  | 277 |
| BA.5 | 294919 | Boosted | 3 | Inactivated vaccines | Inactivated vaccines | Inactivated vaccines | 148 |
| BA.5 | 294921 | Fully vaccinated | 2 | Unknown | Unknown |  |  |
| BA.5 | 294922 | Boosted | 3 | Inactivated vaccines | Inactivated vaccines | Inactivated vaccines | 270 |
| BA.5 | 294923 | Fully vaccinated | 2 | Inactivated vaccines | Inactivated vaccines |  | 136 |
| BA.5 | 294925 | Boosted | 3 | Inactivated vaccines | Inactivated vaccines | Inactivated vaccines | 258 |
| BA.5 | 294926 | Fully vaccinated | 2 | Inactivated vaccines | Inactivated vaccines |  | 199 |
| BA.5 | 294929 | Boosted | 3 | Inactivated vaccines | Inactivated vaccines | Inactivated vaccines | 156 |
| BA.5 | 294930 | Boosted | 3 | Inactivated vaccines | Inactivated vaccines | Inactivated vaccines | 260 |
| BA.5 | 294935 | Boosted | 3 | Inactivated vaccines | Inactivated vaccines | Inactivated vaccines | 253 |
| BA.5 | 294937 | Fully vaccinated | 2 | Inactivated vaccines | Inactivated vaccines |  | 453 |
| BA.5 | 294938 | Boosted | 3 | Inactivated vaccines | Inactivated vaccines | Inactivated vaccines | 251 |
| BA.5 | 294940 | Boosted | 3 | Unknown | Unknown | Unknown |  |
| BA.5 | 294941 | Boosted | 3 | Inactivated vaccines | Inactivated vaccines | Inactivated vaccines | 321 |
| BA.5 | 294942 | Unknown | Unknown |  |  |  |  |
| BA.5 | 294943 | Unvaccinated | 1 | Inactivated vaccines |  |  |  |
| BA.5 | 294944 | Boosted | 3 | Inactivated vaccines | Inactivated vaccines | Inactivated vaccines | 263 |
| BA.5 | 294948 | Fully vaccinated | 2 | Inactivated vaccines | Inactivated vaccines |  | 429 |
| BA.5 | 294949 | Unvaccinated | 0 |  |  |  |  |
| BA.5 | 294950 | Boosted | 3 | Inactivated vaccines | Inactivated vaccines | Inactivated vaccines | 252 |
| BA.5 | 294951 | Boosted | 3 | Unknown | Unknown | Unknown |  |
| BA.5 | 294953 | Fully vaccinated | 2 | Inactivated vaccines | Inactivated vaccines |  | 69 |

| BA.5 | 294954 | Boosted | 3 | Inactivated vaccines | Inactivated vaccines | Inactivated vaccines | 307 |
| --- | --- | --- | --- | --- | --- | --- | --- |
| BA.5 | 294955 | Boosted | 3 | Inactivated vaccines | Inactivated vaccines | Inactivated vaccines | 138 |
| BA.5 | 294956 | Unknown | Unknown |  |  |  |  |
| BA.5 | 294957 | Boosted | 3 | Inactivated vaccines | Inactivated vaccines | Inactivated vaccines | 254 |
| BA.5 | 294958 | Fully vaccinated | 2 | Inactivated vaccines | Inactivated vaccines |  | 274 |
| BA.5 | 294959 | Boosted | 3 | Inactivated vaccines | Inactivated vaccines | Inactivated vaccines | 241 |
| BA.5 | 294960 | Boosted | 3 | Inactivated vaccines | Inactivated vaccines | Inactivated vaccines | 246 |
| BA.5 | 294961 | Boosted | 3 | Inactivated vaccines | Inactivated vaccines | Inactivated vaccines | 132 |
| BA.5 | 294962 | Fully vaccinated | 2 | Unknown | Unknown |  |  |
| BA.5 | 294963 | Boosted | 3 | Inactivated vaccines | Inactivated vaccines | Inactivated vaccines | 241 |
| BA.5 | 294964 | Unvaccinated | 0 |  |  |  |  |
| BA.5 | 294965 | Fully vaccinated | 2 | Inactivated vaccines | Inactivated vaccines |  | 241 |
| BA.5 | 294966 | Boosted | 3 | Inactivated vaccines | Inactivated vaccines | Inactivated vaccines | 159 |
| BA.5 | 294967 | Unknown | Unknown |  |  |  |  |
| BA.5 | 294968 | Boosted | 3 | Inactivated vaccines | Inactivated vaccines | Inactivated vaccines | 243 |
| BA.5 | 294970 | Boosted | 3 | Inactivated vaccines | Inactivated vaccines | Inactivated vaccines | 224 |
| BA.5 | 294972 | Boosted | 3 | Inactivated vaccines | Inactivated vaccines | Inactivated vaccines | 291 |
| BA.5 | 294974 | Boosted | 3 | Inactivated vaccines | Inactivated vaccines | Inactivated vaccines | 392 |
| BA.5 | 294975 | Unvaccinated | 0 |  |  |  |  |
| BA.5 | 294977 | Boosted | 3 | Inactivated vaccines | Inactivated vaccines | Inactivated vaccines | 150 |
| BA.5 | 294982 | Boosted | 3 | Inactivated vaccines | Inactivated vaccines | Inactivated vaccines | 341 |
| BA.5 | 294990 | Fully vaccinated | 2 | Inactivated vaccines | Inactivated vaccines |  | 129 |
| BA.5 | 294999 | Unknown | Unknown |  |  |  |  |
| BA.5 | 295009 | Boosted | 3 | Inactivated vaccines | Inactivated vaccines | Inactivated vaccines | 258 |
| BA.5 | 295015 | Boosted | 3 | Inactivated vaccines | Inactivated vaccines | Inactivated vaccines | 2 |
| BA.5 | 295029 | Boosted | 3 | Inactivated vaccines | Inactivated vaccines | Inactivated vaccines | 61 |
| BA.5 | 295030 | Unvaccinated | 0 |  |  |  |  |
| BA.5 | 295031 | Unvaccinated | 0 |  |  |  |  |
| BA.5 | 295034 | Fully vaccinated | 2 | Inactivated vaccines | Inactivated vaccines |  | 270 |
| BA.5 | 295035 | Fully vaccinated | 2 | Inactivated vaccines | Inactivated vaccines |  | 278 |
| BA.5 | 295036 | Boosted | 3 | Inactivated vaccines | Inactivated vaccines | Inactivated vaccines | 202 |
| BA.5 | 295037 | Unknown | Unknown |  |  |  |  |
| BA.5 | 295038 | Fully vaccinated | 2 | Inactivated vaccines | Inactivated vaccines |  | 280 |
| BA.5 | 295040 | Boosted | 3 | Inactivated vaccines | Inactivated vaccines | Inactivated vaccines | 154 |
| BA.5 | 295041 | Boosted | 3 | Inactivated vaccines | Inactivated vaccines | Inactivated vaccines |  |
| BA.5 | 295047 | Boosted | 3 | Inactivated vaccines | Inactivated vaccines | Inactivated vaccines | 341 |

| BA.5 | 295048 | Boosted | 3 | Inactivated vaccines | Inactivated vaccines | Inactivated vaccines | 341 |
| --- | --- | --- | --- | --- | --- | --- | --- |
| BA.5 | 295051 | Boosted | 3 | Inactivated vaccines | Inactivated vaccines | Inactivated vaccines | 216 |
| BA.5 | 295052 | Boosted | 3 | Inactivated vaccines | Inactivated vaccines | Inactivated vaccines | 296 |
| BA.5 | 295054 | Boosted | 3 | Inactivated vaccines | Inactivated vaccines | Inactivated vaccines | 152 |
| BA.5 | 295055 | Boosted | 3 | Inactivated vaccines | Inactivated vaccines | Inactivated vaccines | 131 |
| BA.5 | 295056 | Unvaccinated | 0 |  |  |  |  |
| BA.5 | 295057 | Unknown | Unknown |  |  |  |  |
| BA.5 | 295058 | Fully vaccinated | 2 | Inactivated vaccines | Inactivated vaccines |  | 459 |
| BA.5 | 295059 | Fully vaccinated | 2 | Inactivated vaccines | Inactivated vaccines |  | 432 |
| BA.5 | 295061 | Boosted | 3 | Inactivated vaccines | Inactivated vaccines | Inactivated vaccines | 252 |
| BA.5 | 295063 | Boosted | 3 | Inactivated vaccines | Inactivated vaccines | Inactivated vaccines | 274 |
| BA.5 | 295064 | Fully vaccinated | 2 | Inactivated vaccines | Inactivated vaccines |  | 460 |
| BA.5 | 295069 | Boosted | 3 | Inactivated vaccines | Inactivated vaccines | Inactivated vaccines | 319 |
| BA.5 | 295070 | Fully vaccinated | 2 | Inactivated vaccines | Inactivated vaccines |  | 336 |
| BA.5 | 295071 | Fully vaccinated | 2 | Inactivated vaccines | Inactivated vaccines |  | 441 |
| BA.5 | 295072 | Boosted | 3 | Inactivated vaccines | Inactivated vaccines | Inactivated vaccines | 141 |
| BA.5 | 295074 | Boosted | 3 | Inactivated vaccines | Inactivated vaccines | Inactivated vaccines | 273 |
| BA.5 | 295076 | Unknown | Unknown |  |  |  |  |
| BA.5 | 295077 | Unknown | Unknown |  |  |  |  |
| BA.5 | 295079 | Boosted | 3 | Inactivated vaccines | Inactivated vaccines | Inactivated vaccines | 257 |
| BA.5 | 295080 | Boosted | 3 | Inactivated vaccines | Inactivated vaccines | Inactivated vaccines | 230 |
| BA.5 | 295081 | Boosted | 3 | Inactivated vaccines | Inactivated vaccines | Inactivated vaccines | 266 |
| BA.5 | 295087 | Boosted | 3 | Inactivated vaccines | Inactivated vaccines | Inactivated vaccines | 250 |
| BA.5 | 295090 | Unknown | Unknown |  |  |  |  |
| BA.5 | 295092 | Unknown | Unknown |  |  |  |  |
| BA.5 | 295094 | Unknown | Unknown |  |  |  |  |
| BA.5 | 295095 | Boosted | 3 | Inactivated vaccines | Inactivated vaccines | Inactivated vaccines | 155 |
| BA.5 | 295100 | Boosted | 3 | Inactivated vaccines | Inactivated vaccines | Inactivated vaccines | 145 |
| BA.5 | 295101 | Fully vaccinated | 2 | Inactivated vaccines | Inactivated vaccines |  | 361 |
| BA.5 | 295102 | Unknown | Unknown |  |  |  |  |
| BA.5 | 295106 | Boosted | 3 | Inactivated vaccines | Inactivated vaccines | Inactivated vaccines | 164 |
| BA.5 | 295109 | Fully vaccinated | 2 | Inactivated vaccines | Inactivated vaccines |  | 271 |
| BA.5 | 295112 | Unknown | Unknown |  |  |  |  |
| BA.5 | 295113 | Unknown | Unknown |  |  |  |  |
| BA.5 | 295114 | Fully vaccinated | 2 | Inactivated vaccines | Inactivated vaccines |  | 377 |
| BA.5 | 295115 | Fully vaccinated | 2 | Inactivated vaccines | Inactivated vaccines |  | 278 |

| BA.5 | 295117 | Boosted | 3 | Inactivated vaccines | Inactivated vaccines | Inactivated vaccines | 65 |
| --- | --- | --- | --- | --- | --- | --- | --- |
| BA.5 | 295118 | Boosted | 3 | Inactivated vaccines | Inactivated vaccines | Inactivated vaccines | 247 |
| BA.5 | 295122 | Boosted | 3 | Inactivated vaccines | Inactivated vaccines | Inactivated vaccines | 228 |
| BA.5 | 295124 | Unknown | Unknown |  |  |  |  |
| BA.5 | 295125 | Boosted | 3 | Inactivated vaccines | Inactivated vaccines | Inactivated vaccines |  |
| BA.5 | 295126 | Fully vaccinated | 2 | Inactivated vaccines | Inactivated vaccines |  | 268 |
| BA.5 | 295127 | Fully vaccinated | 2 |  |  |  |  |
| BA.5 | 295128 | Boosted | 3 | Inactivated vaccines | Inactivated vaccines | Inactivated vaccines | 134 |
| BA.5 | 295129 | Boosted | 3 | Inactivated vaccines | Inactivated vaccines | Inactivated vaccines | 294 |
| BA.5 | 295130 | Boosted | 3 | Inactivated vaccines | Inactivated vaccines | Inactivated vaccines | 274 |
| BA.5 | 295131 | Fully vaccinated | 2 | Inactivated vaccines | Inactivated vaccines |  | 225 |
| BA.5 | 295133 | Boosted | 3 | Inactivated vaccines | Inactivated vaccines | Inactivated vaccines | 240 |
| BA.5 | 295144 | Boosted | 3 | Inactivated vaccines | Inactivated vaccines | Inactivated vaccines | 204 |
| BA.5 | 295148 | Boosted | 3 | Inactivated vaccines | Inactivated vaccines | Inactivated vaccines | 290 |
| BA.5 | 295160 | Unvaccinated | 0 |  |  |  |  |
| BA.5 | 295174 | Fully vaccinated | 2 | Inactivated vaccines | Inactivated vaccines |  | 29 |
| BA.5 | 295175 | Boosted | 3 | Inactivated vaccines | Inactivated vaccines | Inactivated vaccines | 237 |
| BA.5 | 295177 | Boosted | 3 | Inactivated vaccines | Inactivated vaccines | Inactivated vaccines | 245 |
| BA.5 | 295180 | Fully vaccinated | 2 | Inactivated vaccines | Inactivated vaccines |  | 1 |
| BA.5 | 295181 | Boosted | 3 | Inactivated vaccines | Inactivated vaccines | Inactivated vaccines | 251 |
| BA.5 | 295182 | Boosted | 3 | Inactivated vaccines | Inactivated vaccines | Inactivated vaccines | 153 |
| BA.5 | 295183 | Boosted | 3 | Inactivated vaccines | Inactivated vaccines | Inactivated vaccines | 149 |
| BA.5 | 295184 | Boosted | 3 |  |  |  |  |
| BA.5 | 295185 | Boosted | 3 | Inactivated vaccines | Inactivated vaccines | Inactivated vaccines | 235 |
| BA.5 | 295187 | Boosted | 3 |  |  |  |  |
| BA.5 | 295188 | Boosted | 3 | Inactivated vaccines | Inactivated vaccines | Inactivated vaccines | 247 |
| BA.5 | 295193 | Boosted | 3 | Inactivated vaccines | Inactivated vaccines | Inactivated vaccines | 150 |
| BA.5 | 295194 | Boosted | 3 | Inactivated vaccines | Inactivated vaccines | Inactivated vaccines | 150 |
| BA.5 | 295195 | Boosted | 3 | Inactivated vaccines | Inactivated vaccines | Unknown |  |
| BA.5 | 295197 | Unvaccinated | 0 |  |  |  |  |
| BA.5 | 295198 | Boosted | 3 | Inactivated vaccines | Inactivated vaccines | Inactivated vaccines | 242 |
| BA.5 | 295199 | Fully vaccinated | 2 | Inactivated vaccines | Inactivated vaccines |  | 361 |
| BA.5 | 295201 | Boosted | 3 | Inactivated vaccines | Inactivated vaccines | Inactivated vaccines | 87 |
| BA.5 | 295202 | Fully vaccinated | 2 | Inactivated vaccines | Inactivated vaccines |  | 87 |
| BA.5 | 295203 | Fully vaccinated | 2 |  |  |  |  |
| BA.5 | 295205 | Boosted | 3 | Inactivated vaccines | Inactivated vaccines | Inactivated vaccines | 141 |

| BA.5 | 295207 | Boosted | 3 | Inactivated vaccines | Inactivated vaccines | Inactivated vaccines | 244 |
| --- | --- | --- | --- | --- | --- | --- | --- |
| BA.5 | 295210 | Boosted | 3 | Inactivated vaccines | Inactivated vaccines | Inactivated vaccines | 237 |
| BA.5 | 295214 | Boosted | 3 | Inactivated vaccines | Inactivated vaccines | Inactivated vaccines | 269 |
| BA.5 | 295216 | Boosted | 3 | Inactivated vaccines | Inactivated vaccines | Inactivated vaccines | 252 |
| BA.5 | 295222 | Fully vaccinated | 2 | Inactivated vaccines | Inactivated vaccines |  | 364 |
| BA.5 | 295223 | Fully vaccinated | 2 | Inactivated vaccines | Inactivated vaccines |  | 225 |
| BA.5 | 295224 | Unknown | Unknown |  |  |  |  |
| BA.5 | 295230 | Unvaccinated | 1 | Inactivated vaccines |  |  |  |
| BA.5 | 295231 | Boosted | 3 | Inactivated vaccines | Inactivated vaccines | Inactivated vaccines | 178 |
| BA.5 | 295232 | Boosted | 3 | Inactivated vaccines | Inactivated vaccines | Inactivated vaccines | 262 |
| BA.5 | 295236 | Unknown | Unknown |  |  |  |  |
| BA.5 | 295244 | Fully vaccinated | 2 | Inactivated vaccines | Inactivated vaccines |  | 373 |
| BA.5 | 295245 | Unknown | Unknown |  |  |  |  |
| BA.5 | 295247 | Boosted | 3 | Inactivated vaccines | Inactivated vaccines | Inactivated vaccines | 200 |
| BA.5 | 295248 | Fully vaccinated | 2 | Inactivated vaccines | Inactivated vaccines |  | 280 |
| BA.5 | 295249 | Boosted | 3 | Inactivated vaccines | Inactivated vaccines | Inactivated vaccines | 247 |
| BA.5 | 295252 | Boosted | 3 | Inactivated vaccines | Inactivated vaccines | Inactivated vaccines | 135 |
| BA.5 | 295256 | Fully vaccinated | 2 | Inactivated vaccines | Inactivated vaccines |  | 436 |
| BA.5 | 295260 | Unvaccinated | 0 |  |  |  |  |
| BA.5 | 295261 | Unknown | Unknown |  |  |  |  |
| BA.5 | 295262 | Boosted | 3 | Inactivated vaccines | Inactivated vaccines | Inactivated vaccines | 274 |
| BA.5 | 295266 | Boosted | 3 | Inactivated vaccines | Inactivated vaccines | Inactivated vaccines | 143 |
| BA.5 | 295267 | Boosted | 3 | Inactivated vaccines | Inactivated vaccines | Inactivated vaccines | 131 |
| BA.5 | 295268 | Unknown | Unknown |  |  |  |  |
| BA.5 | 295270 | Unknown | Unknown |  |  |  |  |
| BA.5 | 295271 | Boosted | 3 | Inactivated vaccines | Inactivated vaccines | Inactivated vaccines | 239 |
| BA.5 | 295277 | Boosted | 3 | Inactivated vaccines | Inactivated vaccines | Inactivated vaccines | 343 |
| BA.5 | 295278 | Boosted | 3 | Inactivated vaccines | Inactivated vaccines | Inactivated vaccines | 143 |
| BA.5 | 295286 | Boosted | 3 | Inactivated vaccines | Inactivated vaccines | Inactivated vaccines | 288 |
| BA.5 | 295288 | Boosted | 3 | Unknown | Unknown | Unknown |  |
| BA.5 | 295308 | Unvaccinated | 0 |  |  |  |  |
| BA.5 | 295329 | Unvaccinated | 0 |  |  |  |  |
| BA.5 | 295333 | Fully vaccinated | 2 | Inactivated vaccines | Inactivated vaccines |  | 255 |
| BA.5 | 295344 | Boosted | 3 | Inactivated vaccines | Inactivated vaccines | Inactivated vaccines | 129 |
| BA.5 | 295346 | Boosted | 3 | Inactivated vaccines | Inactivated vaccines | Inactivated vaccines | 255 |
| BA.5 | 295347 | Boosted | 3 | Inactivated vaccines | Inactivated vaccines | Inactivated vaccines | 264 |

| BA.5 | 295356 | Unknown | Unknown |  |  |  |  |
| --- | --- | --- | --- | --- | --- | --- | --- |
| BA.5 | 295357 | Boosted | 3 | Inactivated vaccines | Inactivated vaccines | Inactivated vaccines | 275 |
| BA.5 | 295362 | Boosted | 3 | Inactivated vaccines | Inactivated vaccines | Inactivated vaccines | 249 |
| BA.5 | 295364 | Boosted | 3 | Inactivated vaccines | Inactivated vaccines | Inactivated vaccines | 183 |
| BA.5 | 295367 | Boosted | 3 | Inactivated vaccines | Inactivated vaccines | Inactivated vaccines | 152 |
| BA.5 | 295368 | Unknown | Unknown |  |  |  |  |
| BA.5 | 295371 | Unknown | Unknown |  |  |  |  |
| BA.5 | 295372 | Unknown | Unknown |  |  |  |  |
| BA.5 | 295373 | Unknown | Unknown |  |  |  |  |
| BA.5 | 295375 | Unknown | Unknown |  |  |  |  |
| BA.5 | 295376 | Boosted | 3 | Inactivated vaccines | Inactivated vaccines | Inactivated vaccines | 262 |
| BA.5 | 295377 | Unvaccinated | 0 |  |  |  |  |
| BA.5 | 295378 | Boosted | 3 | Inactivated vaccines | Inactivated vaccines | Inactivated vaccines | 262 |
| BA.5 | 295379 | Fully vaccinated | 2 | Inactivated vaccines | Inactivated vaccines |  | 477 |
| BA.5 | 295386 | Boosted | 3 | Inactivated vaccines | Inactivated vaccines | Inactivated vaccines | 158 |
| BA.5 | 295388 | Fully vaccinated | 2 | Inactivated vaccines | Inactivated vaccines |  | 178 |
| BA.5 | 295389 | Boosted | 3 | Inactivated vaccines | Inactivated vaccines | Inactivated vaccines | 263 |
| BA.5 | 295390 | Boosted | 3 | Inactivated vaccines | Inactivated vaccines | Inactivated vaccines | 239 |
| BA.5 | 295391 | Unvaccinated | 0 |  |  |  |  |
| BA.5 | 295392 | Boosted | 3 | Inactivated vaccines | Inactivated vaccines | Inactivated vaccines | 269 |
| BA.5 | 295393 | Fully vaccinated | 2 | Inactivated vaccines | Inactivated vaccines |  | 315 |
| BA.5 | 295394 | Boosted | 3 | Inactivated vaccines | Inactivated vaccines | Inactivated vaccines | 240 |
| BA.5 | 295395 | Boosted | 3 | Inactivated vaccines | Inactivated vaccines | Inactivated vaccines | 279 |
| BA.5 | 295396 | Boosted | 3 | Inactivated vaccines | Inactivated vaccines | Inactivated vaccines | 340 |
| BA.5 | 295397 | Unvaccinated | 0 |  |  |  |  |
| BA.5 | 295398 | Unknown | Unknown |  |  |  |  |
| BA.5 | 295402 | Boosted | 3 | Inactivated vaccines | Inactivated vaccines | Inactivated vaccines | 77 |
| BA.5 | 295403 | Boosted | 3 | Inactivated vaccines | Inactivated vaccines | Inactivated vaccines | 266 |
| BA.5 | 295405 | Boosted | 3 | Inactivated vaccines | Inactivated vaccines | Inactivated vaccines | 148 |
| BA.5 | 295406 | Boosted | 3 | Inactivated vaccines | Inactivated vaccines | Inactivated vaccines | 242 |
| BA.5 | 295408 | Fully vaccinated | 2 | Inactivated vaccines | Inactivated vaccines |  | 141 |
| BA.5 | 295445 | Unknown | Unknown |  |  |  |  |
| BA.5 | 295446 | Boosted | 3 | Inactivated vaccines | Inactivated vaccines | Inactivated vaccines | 137 |
| BA.5 | 295448 | Boosted | 3 | Inactivated vaccines | Inactivated vaccines | Inactivated vaccines | 160 |
| BA.5 | 295477 | Unknown | Unknown |  |  |  |  |
| BA.5 | 295479 | Boosted | 3 | Inactivated vaccines | Inactivated vaccines | Inactivated vaccines | 133 |

| BA.5 | 295482 | Fully vaccinated | 2 | Inactivated vaccines | Inactivated vaccines |  | 163 |
| --- | --- | --- | --- | --- | --- | --- | --- |
| BA.5 | 295487 | Unvaccinated | 1 | Inactivated vaccines |  |  |  |
| BA.5 | 295488 | Fully vaccinated | 2 | Inactivated vaccines | Inactivated vaccines |  | 283 |
| BA.5 | 295490 | Boosted | 3 | Inactivated vaccines | Inactivated vaccines | Inactivated vaccines | 146 |
| BA.5 | 295499 | Unknown | Unknown |  |  |  |  |
| BA.5 | 295500 | Boosted | 3 | Inactivated vaccines | Inactivated vaccines | Inactivated vaccines | 101 |
| BA.5 | 295501 | Boosted | 3 | Inactivated vaccines | Inactivated vaccines | Inactivated vaccines | 140 |
| BA.5 | 295503 | Boosted | 3 | Inactivated vaccines | Inactivated vaccines | Inactivated vaccines | 153 |
| BA.5 | 295505 | Unvaccinated | 1 | Unknown |  |  |  |
| BA.5 | 295508 | Unknown | Unknown |  |  |  |  |
| BA.5 | 295510 | Fully vaccinated | 2 | Inactivated vaccines | Inactivated vaccines |  | 346 |
| BA.5 | 295511 | Boosted | 3 | Unknown | Unknown | Unknown |  |
| BA.5 | 295512 | Boosted | 3 | Inactivated vaccines | Inactivated vaccines | Inactivated vaccines | 256 |
| BA.5 | 295514 | Boosted | 3 | Inactivated vaccines | Inactivated vaccines | Inactivated vaccines | 262 |
| BA.5 | 295528 | Boosted | 3 | Unknown | Unknown | Unknown |  |
| BA.5 | 295546 | Fully vaccinated | 2 | Inactivated vaccines | Inactivated vaccines |  |  |
| BA.5 | 295549 | Boosted | 3 | mRNA | mRNA | mRNA | 158 |
| BA.5 | 295561 | Fully vaccinated | 2 | Inactivated vaccines | Inactivated vaccines |  | 423 |
| BA.5 | 295570 | Fully vaccinated | 2 | Inactivated vaccines | Inactivated vaccines |  | 213 |
| BA.5 | 295572 | Unknown | Unknown |  |  |  |  |
| BA.5 | 295583 | Boosted | 3 | Inactivated vaccines | Inactivated vaccines | Inactivated vaccines | 247 |
| BA.5 | 295584 | Boosted | 3 | Inactivated vaccines | Inactivated vaccines | Inactivated vaccines | 268 |
| BA.5 | 295585 | Boosted | 3 | Inactivated vaccines | Inactivated vaccines | Inactivated vaccines | 207 |
| BA.5 | 295586 | Boosted | 3 | Unknown | Unknown | Inactivated vaccines | 248 |
| BA.5 | 295588 | Fully vaccinated | 2 | Inactivated vaccines | Inactivated vaccines |  | 309 |
| BA.5 | 295589 | Fully vaccinated | 2 | Inactivated vaccines | Inactivated vaccines |  | 284 |
| BA.5 | 295590 | Unvaccinated | 0 |  |  |  |  |
| BA.5 | 295592 | Unknown | Unknown |  |  |  |  |
| BA.5 | 295593 | Boosted | 3 | Inactivated vaccines | Inactivated vaccines | Inactivated vaccines | 13 |
| BA.5 | 295595 | Unknown | Unknown |  |  |  |  |
| BA.5 | 295596 | Boosted | 3 | Inactivated vaccines | Inactivated vaccines | Inactivated vaccines | 242 |
| BA.5 | 295597 | Unvaccinated | 1 | Inactivated vaccines |  |  |  |
| BA.5 | 295598 | Boosted | 3 | Inactivated vaccines | Inactivated vaccines | Inactivated vaccines | 240 |
| BA.5 | 295599 | Boosted | 3 | Inactivated vaccines | Inactivated vaccines | Inactivated vaccines | 297 |
| BA.5 | 295600 | Boosted | 3 | Inactivated vaccines | Inactivated vaccines | Inactivated vaccines | 156 |
| BA.5 | 295601 | Boosted | 3 | Inactivated vaccines | Inactivated vaccines | Inactivated vaccines | 447 |

| BA.5 | 295603 | Boosted | 3 | Inactivated vaccines | Inactivated vaccines | Inactivated vaccines | 175 |
| --- | --- | --- | --- | --- | --- | --- | --- |
| BA.5 | 295607 | Boosted | 3 | Inactivated vaccines | Inactivated vaccines | Inactivated vaccines | 298 |
| BA.5 | 295608 | Fully vaccinated | 2 | Inactivated vaccines | Inactivated vaccines |  | 363 |
| BA.5 | 295610 | Boosted | 3 | Inactivated vaccines | Inactivated vaccines | Inactivated vaccines | 228 |
| BA.5 | 295612 | Fully vaccinated | 2 | Inactivated vaccines | Inactivated vaccines |  | 226 |
| BA.5 | 295614 | Boosted | 3 | Inactivated vaccines | Inactivated vaccines | Inactivated vaccines | 136 |
| BA.5 | 295619 | Fully vaccinated | 2 | Inactivated vaccines | Inactivated vaccines |  | 444 |
| BA.5 | 295622 | Boosted | 3 | Inactivated vaccines | Inactivated vaccines | Inactivated vaccines | 99 |
| BA.5 | 295636 | Boosted | 3 | Unknown | Unknown | Inactivated vaccines | 221 |
| BA.5 | 295655 | Fully vaccinated | 2 | Inactivated vaccines | Inactivated vaccines |  |  |
| BA.5 | 295656 | Unvaccinated | 1 | Inactivated vaccines |  |  |  |
| BA.5 | 295664 | Fully vaccinated | 2 | Inactivated vaccines | Inactivated vaccines |  | 269 |
| BA.5 | 295676 | Fully vaccinated | 2 | Inactivated vaccines | Inactivated vaccines |  | 66 |
| BA.5 | 295677 | Fully vaccinated | 2 | Unknown | Unknown |  |  |
| BA.5 | 295681 | Unvaccinated | 1 | Unknown |  |  |  |
| BA.5 | 295696 | Boosted | 3 | Unknown | Unknown | Inactivated vaccines | 234 |
| BA.5 | 295702 | Unvaccinated | 0 |  |  |  |  |
| BA.5 | 295704 | Boosted | 3 | Inactivated vaccines | Inactivated vaccines | Inactivated vaccines | 143 |
| BA.5 | 295705 | Fully vaccinated | 2 | Inactivated vaccines | Inactivated vaccines |  |  |
| BA.5 | 295706 | Fully vaccinated | 2 | Inactivated vaccines | Inactivated vaccines |  | 124 |
| BA.5 | 295708 | Boosted | 3 | Inactivated vaccines | Inactivated vaccines | Inactivated vaccines | 190 |
| BA.5 | 295709 | Fully vaccinated | 2 | Inactivated vaccines | Inactivated vaccines |  | 114 |
| BA.5 | 295710 | Fully vaccinated | 2 | Inactivated vaccines | Inactivated vaccines |  | 243 |
| BA.5 | 295711 | Boosted | 3 | Inactivated vaccines | Inactivated vaccines | Inactivated vaccines | 271 |
| BA.5 | 295712 | Boosted | 3 | Unknown | Unknown | Unknown | 190 |
| BA.5 | 295713 | Boosted | 3 | Unknown | Unknown | Unknown |  |
| BA.5 | 295714 | Boosted | 3 | Unknown | Unknown | Unknown |  |
| BA.5 | 295717 | Boosted | 3 | Inactivated vaccines | Inactivated vaccines | Inactivated vaccines | 139 |
| BA.5 | 295719 | Boosted | 3 | Inactivated vaccines | Inactivated vaccines | Inactivated vaccines | 343 |
| BA.5 | 295721 | Boosted | 3 | Unknown | Unknown | Unknown |  |
| BA.5 | 295725 | Boosted | 3 | Inactivated vaccines | Inactivated vaccines | Inactivated vaccines |  |
| BA.5 | 295728 | Boosted | 3 | Inactivated vaccines | Inactivated vaccines | Inactivated vaccines | 137 |
| BA.5 | 295729 | Unvaccinated | 0 |  |  |  |  |
| BA.5 | 295730 | Boosted | 3 | Inactivated vaccines | Inactivated vaccines | Inactivated vaccines | 280 |
| BA.5 | 295731 | Fully vaccinated | 2 | Unknown | Unknown |  |  |
| BA.5 | 295733 | Boosted | 3 | Inactivated vaccines | Inactivated vaccines | Inactivated vaccines | 259 |

| BA.5 | 295735 | Boosted | 3 | Inactivated vaccines | Inactivated vaccines | Inactivated vaccines | 137 |
| --- | --- | --- | --- | --- | --- | --- | --- |
| BA.5 | 295736 | Boosted | 3 | Inactivated vaccines | Inactivated vaccines | Inactivated vaccines | 273 |
| BA.5 | 295737 | Boosted | 3 | Inactivated vaccines | Inactivated vaccines | Inactivated vaccines | 137 |
| BA.5 | 295738 | Unvaccinated | 0 |  |  |  |  |
| BA.5 | 295739 | Unvaccinated | 0 |  |  |  |  |
| BA.5 | 295743 | Boosted | 3 | Inactivated vaccines | Inactivated vaccines | Inactivated vaccines | 147 |
| BA.5 | 295745 | Boosted | 3 | Inactivated vaccines | Inactivated vaccines | Inactivated vaccines | 363 |
| BA.5 | 295746 | Unvaccinated | 0 |  |  |  |  |
| BA.5 | 295748 | Boosted | 3 | Inactivated vaccines | Inactivated vaccines | Inactivated vaccines | 269 |
| BA.5 | 295749 | Unvaccinated | 1 | Unknown |  |  |  |
| BA.5 | 295753 | Boosted | 3 | Inactivated vaccines | Inactivated vaccines | Inactivated vaccines | 157 |
| BA.5 | 295767 | Boosted | 3 | Unknown | Unknown | Unknown |  |
| BA.5 | 295768 | Fully vaccinated | 2 | Inactivated vaccines | Inactivated vaccines |  | 256 |
| BA.5 | 295770 | Boosted | 3 | Inactivated vaccines | Inactivated vaccines | Inactivated vaccines | 270 |
| BA.5 | 295778 | Unvaccinated | 0 |  |  |  |  |
| BA.5 | 295779 | Fully vaccinated | 2 | Inactivated vaccines | Inactivated vaccines |  | 115 |
| BA.5 | 295782 | Unvaccinated | 1 | Inactivated vaccines |  |  |  |
| BA.5 | 295786 | Fully vaccinated | 2 | Inactivated vaccines | Inactivated vaccines |  | 25 |
| BA.5 | 295787 | Boosted | 3 | Inactivated vaccines | Inactivated vaccines | Inactivated vaccines | 229 |
| BA.5 | 295790 | Fully vaccinated | 2 | Inactivated vaccines | Inactivated vaccines |  | 270 |
| BA.5 | 295791 | Boosted | 3 | Inactivated vaccines | Inactivated vaccines | Inactivated vaccines | 272 |
| BA.5 | 295797 | Boosted | 3 | Inactivated vaccines | Inactivated vaccines | Inactivated vaccines | 162 |
| BA.5 | 295817 | Boosted | 3 | Inactivated vaccines | Inactivated vaccines | Inactivated vaccines | 151 |
| BA.5 | 295818 | Fully vaccinated | 2 | Unknown | Unknown |  |  |
| BA.5 | 295832 | Unvaccinated | 1 | Inactivated vaccines |  |  |  |

**Table S4.** **Odds** **Ratios** **for** **Symptoms** **and** **Computed** **Tomography** **of** **BA.5** ***vs*** **BA.2**

| **Table S4-1:** **Odds** **Ratio** **for** **Symptoms** **of** **BA.5** **vs** **BA.2** | | | | | | | |
| --- | --- | --- | --- | --- | --- | --- | --- |
| **Symptoms** | **BA.2** **proportion** | **BA.5** **proportion** | **BA.2** **vs** **BA.5**  **OR** **[95%** **CI]** | **BA.5** **vs** **BA.2**  **OR** **[95%** **CI]** | **BA.5** **vs** **BA.2**  **adjusted** **odds** **ratio** **[95%** **CI]** | **P** **value** | **Significant** |
| Fever | 30.96 | 44.63 | 0.56 [0.43-0.73] | 1.80 [ 1.38-2.35] | 2. 11 [ 1.57-2.81] | 9.09E-06 | YES |
| Cough | 46.84 | 21.46 | 3.22 [2.41-4.33] | 0.31 [0.23-0.42] | 0.31 [0.23-0.42] | < 2.2e- 16 | YES |
| Hawking | 16.21 | 2.20 | 8.61 [4.3- 19.62] | 0. 12 [0.05-0.23] | 0. 11 [0.05-0.23] | 9. 16E- 15 | YES |
| Chilly or Shiver | 0.00 | 1.71 | 0 [0-0.46] | Inf [2. 19-Inf] |  | 0.001567 | YES |
| Skipped Meals | 0.32 | 0.00 |  |  |  | 0.5198 | NO |
| Eye Soreness | 0. 16 | 0.24 | 0.66 [0.008-52.22] | 1.5 [0.02- 118.31] |  | 1 | NO |
| Hoarse Voice | 0.65 | 0.49 | 1.33 [0. 19- 14.78] | 0.75 [0.07-5.27] |  | 1 | NO |
| Palpitation | 0.32 | 0.00 | Inf [0. 12-Inf] | 0 [0-8.01] |  | 0.5198 | NO |
| Abdominal Pain | 0.32 | 0.24 | 1.33 [0.07-78.63] | 0.75 [0.01- 14.49] |  | 1 | NO |
| Unusual Joint Pain | 0. 16 | 0.00 |  |  |  | 1 | NO |
| Xerostomia | 0.65 | 0.00 |  |  |  | 0. 1549 | NO |
| Hypogeusia | 0.32 | 0.49 | 0.66 [0.05-9. 19] | 1.51 [0. 11-20.86] |  | 0.6534 | NO |
| Hyposmia | 0. 16 | 0.00 |  |  |  | 1 | NO |
| Shortness of breath | 0.65 | 0.00 |  |  |  | 0. 1549 | NO |
| Neck discomfort | 0. 16 | 0.00 |  |  |  | 1 | NO |
| Chest tightness | 2. 11 | 0.00 |  |  |  | 0.002473 | YES |
| Sneezing | 0. 16 | 0.00 |  |  |  | 1 | NO |
| Pharyngeal discomfort | 0.65 | 2.44 | 0.26 [0.06-0.91] | 3.83 [ 1.09- 16.82] | 4.41 [ 1. 19- 16.28] | 0.02452 | YES |
| Sore Throat | 23.82 | 23. 17 | 1.04 [0.76- 1.41] | 0.96 [0.71- 1.31] |  | 0.8222 | NO |
| Dry throat | 6.81 | 1.71 | 4.2 [ 1.85- 11.20] | 0.24 [0.09-0.54] | 0.2 [0.09-0.44] | 0.0001314 | YES |
| Throat Itching | 6.48 | 1.71 | 3.97 [ 1.74- 10.65] | 0.25 [0.09-0.57] | 0.22 [0.09-0.51] | 0.0002027 | YES |
| Diarrhea | 3.57 | 1.46 | 2.49 [0.97-7.57] | 0.40 [0. 13- 1.03] | 0.34 [0. 13-0.87] | 0.04994 | YES |
| Nasal Obstruction | 7.94 | 5. 12 | 1.60 [0.92-2.85] | 0.63 [0.35- 1.08] |  | 0.09967 | NO |
| Runny Nose | 7.62 | 3.66 | 2. 17 [ 1. 17-4.24] | 0.46 [0.24-0.85] | 0.40 [0.22-0.75] | 0.01053 | YES |
| Fatigue | 10.21 | 14. 15 | 0.69 [0.46- 1.03] | 1.45 [0.97-2. 16] |  | 0.06057 | NO |
| Nausea | 0.97 | 0.49 | 2.00 [0.36-20.38] | 0.50 [0.05-2.81] |  | 0.4878 | NO |
| Emesis | 0.81 | 1.22 | 0.66 [0. 15-2.90] | 1.51 [0.35-6.61] |  | 0.5317 | NO |
| Heavy-headedness | 0. 16 | 0.00 |  |  |  | 1 | NO |
| Headache | 4.05 | 5.61 | 0.71 [0.38- 1.33] | 1.41 [0.75-2.62] |  | 0.2908 | NO |
| Dizzy | 1.78 | 3.41 | 0.51 [0.21- 1.23] | 1.95 [0.81-4.79] |  | 0. 1025 | NO |
| Unusual Muscle Pains | 3.40 | 8.29 | 0.39 [0.211-0.70] | 2.56 [ 1.42-4.72] | 2.42 [ 1.34-4.39] | 0.0009763 | YES |

| **Table S4-2:** **Odds** **Ratio** **for** **Symptoms** **of** **BA.5** **vs** **BA.2** | | | | | | | |
| --- | --- | --- | --- | --- | --- | --- | --- |
| **Computed** **Tomography** | **BA.2** **proportion** | **BA.5** **proportion** | **BA.2** **vs** **BA.5**  **OR** **[95%** **CI]** | **BA.5** **vs** **BA.2**  **OR** **[95%** **CI]** | **BA.5** **vs** **BA.2**  **adjusted** **odds** **ratio** **[95%** **CI]** | **P** **value** | **Significant** |
| Ground Glass Nodules Ground Glass Stove | 21.94304858 | 9.638554217 | 2.63 [ 1.72-4. 12] | 0.38 [0.24-0.58] | 0.34 [0.21-0.50] | 1.5E-06 | NO |
| Lung nodules | 31.83 | 26.20 | 1.31 [0.97- 1.80] | 0.76 [0.56- 1.04] |  | 0.08511 | NO |
| Fibrous lesions | 4.02 | 9.04 | 0.42 [0.23-0.76] | 2.37 [ 1.31-4.32] | 2.05 [ 1.09-3.62] | 0.00305 | YES |
| Cable stove | 17.09 | 14.46 | 1.22 [0.83- 1.81] | 0.82 [0.55- 1.21] |  | 0.31 | NO |
| Chronic inflammation | 16.42 | 15.36 | 1.08 [0.74- 1.60] | 0.92 [0.63- 1.35] |  | 0.7097 | NO |
| Calcification | 7.71 | 7.83 | 0.98 [0.58- 1.69] | 1.02 [0.59- 1.72] |  | 1 | NO |
| Bullae | 3.85 | 4.22 | 0.91 [0.44- 1.94] | 1. 10 [0.51-2.26] |  | 0.8613 | NO |
| Emphysema | 3.02 | 2. 11 | 1.44 [0.57-4. 13] | 0.69 [0.24- 1.76] |  | 0.5273 | NO |
| Bad gas | 0.50 | 0.30 | 1.67 [0. 13-87.99] | 0.60 [0.01-7.49] |  | 1 | NO |
| Secondary pulmonary tuberculosis | 0.84 | 1.20 | 0.69 [0. 15-3.52] | 1.44 [0.28-6.76] |  | 0.7287 | NO |
| Old tuberculosis | 0.50 |  |  |  |  | 0.5566 | NO |
| Bronchiectasis | 1.01 | 2.41 | 0.41 [0. 12- 1.37] | 2.43 [0.73-8.57] |  | 0.1 | NO |
| Infection focus | 6.87 | 1.20 | 6.04 [2. 16-23.42] | 0. 17 [0.04-0.46] | 0.40 [0. 11- 1. 18] | 4.24E-05 | YES |
| Pericardial effusion | 0. 17 | 1.20 | 0. 14 [0.002- 1.4] 7 | .25 [0.71-357.85] | | 0.05774 | NO |
| Mucus plug | 0. 17 |  |  |  |  | 1 | NO |
| Pleural effusion | 0.34 | 0.90 | 0.37 [0.03-3.24] | 2.71 [0.31-32.60] |  | 0.3552 | NO |
| Pleural thickening | 1.51 | 1.51 | 0.98 [0.29-3.77] | 1.00 [0.26-3.35] |  | 1 | NO |
| Single lung involved | 5.03 | 3.01 | 1.7 [0.80-3.96] | 0.59 [0.25- 1.25] |  | 0. 1779 | NO |
| Double lung involved | 41.37 | 28.61 | 1.76 [ 1.31-2.38] | 0.57 [0.42-0.76] | 0.42 [0.29-0.55] | 0.00012 | YES |
| Multilobar lesions | 1.34 | 8.73 | 0. 14 [0.06-0.32] | 7.03 [3.01- 18.02] | 5.67 [2.47- 13.78] | 1.32E-07 | YES |
| Unilobular lesions | 18.93 | 19.28 | 0.98 [0.69- 1.40] | 1.02 [0.71- 1.46] |  | 0.9306 | NO |
| Normal | 31.66 | 41.57 | 0.65 [0.49-0.87] | 1.53 [ 1. 15-2.04] | 1.01 [0.79- 1.41] | 0.00263 | YES |

| **Table S4-3:** **Correlation** **between** **symptoms** **and** **CT** | | | | |
| --- | --- | --- | --- | --- |
| **Symptoms** **or** **CT** | **Symptoms** **or** **CT** | **Relevance** | **P** **value** | **Correlation** |
| Cable stove | Normal | -0.3089572 | 1.50E- 11 | negative |
| Chronic inflammation | Normal | -0.3016224 | 6.65E- 11 | negative |
| Cough | Hawking | 0.3409337 | 1.35E- 14 | positive |
| Double lung involved | Lung nodules | 0.4190407 | 8.72E-27 | positive |
| Double lung involved | Normal | -0.571762 | 5.36E-50 | negative |
| Double lung involved | Unilobular lesions | -0.4059112 | 5.78E-22 | negative |
| Emesis | Nausea | 0.3592839 | 1.25E- 19 | positive |
| Hawking | Cough | 0.3409337 | 1.03E- 17 | positive |
| Hypogeusia | Hyposmia | 0.7065133 | 2.80E-88 | positive |
| Hyposmia | Hypogeusia | 0.7065133 | 2. 11E-91 | positive |
| Lung nodules | Double lung involved | 0.4190407 | 1. 15E-23 | positive |
| Lung nodules | Normal | -0.4650294 | 3.05E-30 | negative |
| Nasal Obstruction | Runny Nose | 0.411287 | 1. 19E-22 | positive |
| Nausea | Emesis | 0.3592839 | 1.65E- 16 | positive |
| Nausea | Xerostomia | 0.4034216 | 9.01E-25 | positive |
| Normal | Cable stove | -0.3089572 | 1. 14E- 14 | negative |
| Normal | Chronic inflammation | -0.3016224 | 5.06E- 14 | negative |
| Normal | Double lung involved | -0.571762 | 4.05E-53 | negative |
| Normal | Lung nodules | -0.4650294 | 2.30E-33 | negative |
| Normal | Unilobular lesions | -0.3288648 | 1.60E- 16 | negative |
| Runny Nose | Nasal Obstruction | 0.411287 | 8.99E-26 | positive |
| Unilobular lesions | Double lung involved | -0.4059112 | 4.37E-25 | negative |
| Unilobular lesions | Normal | -0.3288648 | 2. 10E- 13 | negative |
| Xerostomia | Nausea | 0.4034216 | 1. 19E-21 | positive |

| **Table S4-4:** **Correlation** **between** **symptoms** **and** **severity** | | | | | | | |
| --- | --- | --- | --- | --- | --- | --- | --- |
| **Variants** | **Symptoms** | **Moderate** **(n)** | **Mild** **(n)** | **Moderate** **vs** **Mild** **OR** **[95%** **CI]** | **Moderate** **vs** **Mild** **adjusted** **odds** **ratio** **[CI** | **P** **value** | **Significant** |
| BA.2 | Fever | 32 | 159 | 0.67 [0.41- 1.05] |  | 0.07113 | NO |
| BA.2 | Cough | 72 | 217 | 1.51 [ 1.01-2.27] | 1.49 [0.93-2.35] | 0.03857 | YES |
| BA.2 | Hawking | 25 | 75 | 1.29 [0.75-2. 17] |  | 0.3495 | NO |
| BA.2 | Skipped Meals | 0 | 2 |  |  |  | NO |
| BA.2 | Eye Soreness | 0 | 1 |  |  |  | NO |
| BA.2 | Hoarse Voice | 1 | 3 |  |  | 1 | NO |
| BA.2 | Palpitation | 0 | 2 |  |  |  | NO |
| BA.2 | Abdominal Pain | 1 | 1 | 3.72 [0.05-292.73] |  | 0.3798 | NO |
| BA.2 | Unusual Joint Pain | 0 | 1 |  |  |  | NO |
| BA.2 | Xerostomia | 1 | 3 |  |  | 1 | NO |
| BA.2 | Hypogeusia | 0 | 2 |  |  |  | NO |
| BA.2 | Hyposmia | 0 | 1 |  |  |  | NO |
| BA.2 | Shortness of breath | 1 | 3 |  |  | 1 | NO |
| BA.2 | Neck discomfort | 0 | 1 |  |  |  | NO |
| BA.2 | Chest tightness | 1 | 12 | 0.3 [0.01-2.09] |  | 0.3187 | NO |
| BA.2 | Sneezing | 1 | 0 |  |  |  | NO |
| BA.2 | Pharyngeal discomfort | 1 | 3 |  |  | 1 | NO |
| BA.2 | Sore Throat | 34 | 113 | 1. 16 [0.72- 1.83] |  | 0.5635 | NO |
| BA.2 | Dry throat | 10 | 32 | 1. 17 [0.5-2.53] |  | 0.6962 | NO |
| BA.2 | Throat Itching | 9 | 31 | 1.08 [0.44-2.41] |  | 0.8421 | NO |
| BA.2 | Diarrhea | 3 | 19 | 0.58 [0. 11-2.0] |  | 0.5944 | NO |
| BA.2 | Nasal Obstruction | 8 | 41 | 0.71 [0.28- 1.58] |  | 0.4683 | NO |
| BA.2 | Runny Nose | 9 | 38 | 0.87 [0.36- 1.89] |  | 0.8534 | NO |
| BA.2 | Fatigue | 12 | 51 | 0.86 [0.40- 1.70] |  | 0.7465 | NO |
| BA.2 | Nausea | 1 | 5 | 0.74 [0.02-6.70] |  | 1 | NO |
| BA.2 | Emesis | 0 | 5 |  |  |  | NO |
| BA.2 | Heavy-headedness | 0 | 1 |  |  |  | NO |
| BA.2 | Headache | 5 | 20 | 0.92 [0.27-2.61] |  | 1 | NO |
| BA.2 | Dizzy | 1 | 10 | 0.37 [0.01-2.62] |  | 0.4724 | NO |
| BA.2 | Unusual Muscle Pains | 5 | 16 | 1. 17 [0.33-3.41] |  | 0.7865 | NO |

| BA.5 | Fever | 16 | 167 | 1.26 [0.57-2.79] |  | 0.5806 | NO |
| --- | --- | --- | --- | --- | --- | --- | --- |
| BA.5 | Cough | 6 | 82 | 0.833 [0.27-2. 16] |  | 0.8248 | NO |
| BA.5 | Hawking | 1 | 8 | 1.49 [0.03- 11.74] |  | 0.5224 | NO |
| BA.5 | Chilly or Shiver | 1 | 6 | 2.00 [0.04- 17.27] |  | 0.4363 | NO |
| BA.5 | Pharyngeal discomfort | 0 | 10 |  |  |  | NO |
| BA.5 | Hoarse Voice | 0 | 2 |  |  |  | NO |
| BA.5 | Dry throat | 1 | 6 | 2.00 [0.04- 17.27] |  | 0.4363 | NO |
| BA.5 | Sore Throat | 11 | 84 | 1.83 [0.76-4. 16] |  | 0. 1284 | NO |
| BA.5 | Throat Itching | 0 | 7 |  |  |  | NO |
| BA.5 | Dizzy | 0 | 14 |  |  |  | NO |
| BA.5 | Headache | 3 | 20 | 1.85 [0.33-6.80] |  | 0.4094 | NO |
| BA.5 | Fatigue | 5 | 53 | 1. 13 [0.33-3. 17] |  | 0.792 | NO |
| BA.5 | Nasal Obstruction | 1 | 20 | 0.58 [0.01-3.87] |  | 1 | NO |
| BA.5 | Runny Nose | 1 | 14 | 0.84 [0.02-5.88] |  | 1 | NO |
| BA.5 | Diarrhea | 1 | 5 | 2.40 [0.05-22.43] |  | 0.3878 | NO |
| BA.5 | Unusual Muscle Pains | 4 | 30 | 1.65 [0.39-5.20] |  | 0.3239 | NO |
| BA.5 | Abdominal Pain | 0 | 1 |  |  |  | NO |
| BA.5 | Nausea | 0 | 2 |  |  |  | NO |
| BA.5 | Emesis | 0 | 5 |  |  |  | NO |
| BA.5 | Hypogeusia | 0 | 2 |  |  |  | NO |
| BA.5 | Eye Soreness | 0 | 1 |  |  |  | NO |

**Table S5.** **Nucleic** **acid** **information** **of** **BA.2** **and** **BA.5**

| **Variants** | **ID** | **Gender** | **Age** | **Severity** | **Vaccination** | **Peak** **Ct** **(N** **gene)** | **Days** **for** **peak** **viral** **load** | **Days** **for** **viral** **clearance** |
| --- | --- | --- | --- | --- | --- | --- | --- | --- |
| BA.2 | 158689 | female | 35 | Mild | Boosted | 17.29 | 3 | 15 |
| BA.2 | 274953 | male | 22 | Asymptomatic | Fully Vaccinated | 21. 13 | 2 | 9 |
| BA.2 | 275062 | male | 39 | Moderate | Fully Vaccinated | 13.83 | 4 | 16 |
| BA.2 | 275212 | male | 33 | Mild | Boosted | 14.66 | 3 | 18 |
| BA.2 | 275481 | female | 29 | Mild | Fully Vaccinated | 15.44 | 4 | 15 |
| BA.2 | 275576 | male | 27 | Mild | Unvaccinated | 21.68 | 6 | 19 |
| BA.2 | 275582 | male | 36 | Mild | Fully Vaccinated | 14. 18 | 5 | 21 |
| BA.2 | 275583 | female | 26 | Moderate | Fully Vaccinated | 15.35 | 5 | 21 |
| BA.2 | 275584 | male | 44 | Moderate | Boosted | 16.9 | 2 | 14 |
| BA.2 | 275590 | female | 45 | Moderate | Boosted | 16.25 | 2 | 15 |
| BA.2 | 275595 | female | 47 | Mild | Boosted | 16.81 | 6 | 15 |
| BA.2 | 275596 | female | 31 | Moderate | Unvaccinated | 18.48 | 4 | 14 |
| BA.2 | 275600 | male | 24 | Mild | Boosted | 15.32 | 2 | 13 |
| BA.2 | 275603 | male | 28 | Moderate | Unvaccinated | 15.5 | 5 | 26 |
| BA.2 | 275605 | female | 42 | Asymptomatic | Boosted | 15.73 | 5 | 14 |
| BA.2 | 275612 | female | 55 | Moderate | Boosted | 20. 16 | 7 | 14 |
| BA.2 | 275619 | male | 39 | Asymptomatic | Boosted | 15.22 | 5 | 22 |
| BA.2 | 275622 | male | 46 | Moderate | Boosted | 18.02 | 5 | 10 |
| BA.2 | 275623 | male | 37 | Moderate | Boosted | 15.21 | 2 | 13 |
| BA.2 | 275627 | female | 25 | Mild | Fully Vaccinated | 17.62 | 5 | 15 |
| BA.2 | 275631 | female | 43 | Moderate | Fully Vaccinated | 17.46 | 5 | 12 |
| BA.2 | 275643 | male | 72 | Moderate | Fully Vaccinated | 15.33 | 7 | 17 |
| BA.2 | 275650 | female | 43 | Mild | Boosted | 21.09 | 5 | 13 |
| BA.2 | 275660 | female | 40 | Moderate | Fully Vaccinated | 16.32 | 3 | 13 |
| BA.2 | 275662 | male | 20 | Mild | Unvaccinated | 21.82 | 6 | 14 |
| BA.2 | 275674 | male | 44 | Mild | Boosted | 15.53 | 5 | 12 |
| BA.2 | 275676 | male | 31 | Mild | Fully Vaccinated | 16.85 | 3 | 16 |
| BA.2 | 275677 | male | 49 | Mild | Boosted | 13.08 | 3 | 13 |
| BA.2 | 275678 | male | 10.8 | Moderate | Fully Vaccinated | 19.79 | 4 | 12 |

| BA.2 | 275691 | male | 54 | Asymptomatic | Boosted | 17.7 | 4 | 19 |
| --- | --- | --- | --- | --- | --- | --- | --- | --- |
| BA.2 | 275721 | male | 10.9 | Mild | Unvaccinated | 19.66 | 2 | 14 |
| BA.2 | 275729 | male | 26 | Asymptomatic | Fully Vaccinated | 15.88 | 7 | 12 |
| BA.2 | 275743 | female | 41 | Moderate | Fully Vaccinated | 12.21 | 4 | 15 |
| BA.2 | 275767 | female | 68 | Mild | Fully Vaccinated | 16.32 | 6 | 28 |
| BA.2 | 275799 | female | 20 | Moderate | Boosted | 18.35 | 3 | 15 |
| BA.2 | 275809 | female | 20 | Moderate | Fully Vaccinated | 21.49 | 4 | 20 |
| BA.2 | 275887 | female | 80 | Moderate | Unvaccinated | 14.86 | 2 | 16 |
| BA.2 | 275910 | male | 65 | Mild | Fully Vaccinated | 20.59 | 4 | 15 |
| BA.2 | 275921 | female | 42 | Moderate | Boosted | 14.46 | 7 | 20 |
| BA.2 | 275922 | male | 62 | Moderate | Boosted | 18.86 | 5 | 14 |
| BA.2 | 275923 | female | 45 | Mild | Fully Vaccinated | 12.43 | 5 | 19 |
| BA.2 | 275944 | male | 80 | Moderate | Fully Vaccinated | 15. 17 | 5 | 18 |
| BA.2 | 275946 | male | 50 | Moderate | Boosted | 12.9 | 5 | 17 |
| BA.2 | 276007 | female | 58 | Mild | Boosted | 16.99 | 6 | 14 |
| BA.2 | 276082 | male | 39 | Mild | Unvaccinated | 17.47 | 5 | 16 |
| BA.2 | 276093 | male | 63 | Moderate | Boosted | 19.8 | 6 | 17 |
| BA.2 | 276094 | male | 64 | Moderate | Fully Vaccinated | 15.73 | 5 | 12 |
| BA.2 | 276103 | female | 45 | Mild | Boosted | 19. 17 | 7 | 16 |
| BA.2 | 276145 | female | 69 | Mild | Fully Vaccinated | 13.78 | 4 | 24 |
| BA.2 | 276161 | male | 1. 17 | Moderate | Unvaccinated | 12.8 | 2 | 9 |
| BA.2 | 276162 | male | 57 | Moderate | Fully Vaccinated | 17.58 | 8 | 17 |
| BA.2 | 276163 | male | 27 | Asymptomatic | Fully Vaccinated | 21.81 | 7 | 13 |
| BA.2 | 276164 | female | 27 | Mild | Unvaccinated | 13.57 | 3 | 14 |
| BA.2 | 276170 | female | 56 | Mild | Unvaccinated | 16. 15 | 2 | 13 |
| BA.2 | 276171 | male | 2. 12 | Mild | Unvaccinated | 13.92 | 2 | 13 |
| BA.2 | 276172 | female | 52 | Mild | Fully Vaccinated | 11.89 | 2 | 13 |
| BA.2 | 276179 | female | 51 | Moderate | Boosted | 15.23 | 8 | 23 |
| BA.2 | 276186 | male | 52 | Moderate | Fully Vaccinated | 18.03 | 3 | 17 |
| BA.2 | 276189 | male | 51 | Mild | Fully Vaccinated | 18.95 | 8 | 20 |
| BA.2 | 276192 | male | 20 | Mild | Boosted | 17.65 | 4 | 14 |
| BA.2 | 276232 | male | 1.84 | Mild | Unvaccinated | 14.99 | 2 | 11 |

| BA.2 | 276233 | female | 2.98 | Mild | Unvaccinated | 12.43 | 2 | 16 |
| --- | --- | --- | --- | --- | --- | --- | --- | --- |
| BA.2 | 276235 | female | 29 | Asymptomatic | Fully Vaccinated | 22.43 | 7 | 14 |
| BA.2 | 276241 | male | 26 | Mild | Fully Vaccinated | 18.79 | 3 | 15 |
| BA.2 | 276243 | male | 34 | Mild | Boosted | 13.63 | 5 | 13 |
| BA.2 | 276247 | male | 38 | Mild | Fully Vaccinated | 15.58 | 2 | 13 |
| BA.2 | 276248 | female | 62 | Mild | Fully Vaccinated | 12.87 | 3 | 21 |
| BA.2 | 276249 | male | 17 | Mild | Fully Vaccinated | 16.25 | 2 | 11 |
| BA.2 | 276250 | male | 49 | Mild | Boosted | 19.49 | 8 | 15 |
| BA.2 | 276251 | female | 9.42 | Mild | Fully Vaccinated | 25.22 | 1 | 8 |
| BA.2 | 276252 | male | 2.38 | Mild | Unvaccinated | 26.48 | 2 | 13 |
| BA.2 | 276253 | female | 26 | Mild | Boosted | 20.76 | 2 | 12 |
| BA.2 | 276312 | female | 59 | Mild | Fully Vaccinated | 13.36 | 4 | 16 |
| BA.2 | 276313 | female | 38 | Mild | Fully Vaccinated | 15.05 | 3 | 25 |
| BA.2 | 276314 | female | 33 | Mild | Unvaccinated | 14.39 | 3 | 17 |
| BA.2 | 276316 | female | 28 | Mild | Fully Vaccinated | 13.04 | 6 | 27 |
| BA.2 | 276319 | male | 47 | Mild | Boosted | 13.58 | 7 | 19 |
| BA.2 | 276325 | male | 51 | Moderate | Fully Vaccinated | 16.55 | 2 | 15 |
| BA.2 | 276326 | male | 54 | Moderate | Fully Vaccinated | 14.32 | 3 | 16 |
| BA.2 | 276327 | male | 63 | Mild | Boosted | 16. 17 | 8 | 23 |
| BA.2 | 276328 | female | 36 | Mild | Boosted | 12.98 | 2 | 8 |
| BA.2 | 276349 | female | 46 | Mild | Fully Vaccinated | 13.38 | 4 | 15 |
| BA.2 | 276369 | male | 57 | Mild | Fully Vaccinated | 14.83 | 4 | 16 |
| BA.2 | 276373 | male | 5.67 | Mild | Unvaccinated | 21.01 | 3 | 11 |
| BA.2 | 276405 | female | 25 | Mild | Unvaccinated | 17.57 | 7 | 16 |
| BA.2 | 276409 | female | 35 | Mild | Fully Vaccinated | 19.46 | 6 | 22 |
| BA.2 | 276410 | female | 23 | Mild | Fully Vaccinated | 14. 15 | 4 | 19 |
| BA.2 | 276442 | male | 7.92 | Mild | Unvaccinated | 21. 16 | 2 | 11 |
| BA.2 | 276444 | female | 20 | Mild | Boosted | 15.52 | 3 | 14 |
| BA.2 | 276457 | male | 72 | Moderate | Fully Vaccinated | 14.58 | 6 | 15 |
| BA.2 | 276459 | female | 12.9 | Mild | Unvaccinated | 15.67 | 5 | 27 |
| BA.2 | 276460 | female | 35 | Mild | Fully Vaccinated | 14.45 | 4 | 11 |
| BA.2 | 276461 | female | 27 | Mild | Fully Vaccinated | 16.61 | 7 | 15 |

| BA.2 | 276471 | male | 33 | Moderate | Boosted | 14.08 | 3 | 15 |
| --- | --- | --- | --- | --- | --- | --- | --- | --- |
| BA.2 | 276472 | male | 27 | Mild | Boosted | 21.35 | 5 | 21 |
| BA.2 | 276498 | male | 51 | Mild | Boosted | 17. 11 | 6 | 14 |
| BA.2 | 276503 | male | 30 | Mild | Fully Vaccinated | 15.09 | 3 | 19 |
| BA.2 | 276509 | female | 45 | Moderate | Boosted | 13.07 | 3 | 10 |
| BA.2 | 276530 | male | 23 | Mild | Fully Vaccinated | 22.06 | 9 | 13 |
| BA.2 | 276531 | male | 17 | Mild | Fully Vaccinated | 22.78 | 8 | 11 |
| BA.2 | 276542 | male | 57 | Moderate | Fully Vaccinated | 15.54 | 6 | 19 |
| BA.2 | 276553 | female | 54 | Mild | Boosted | 20.9 | 8 | 15 |
| BA.2 | 276570 | female | 45 | Mild | Unvaccinated | 13. 1 | 5 | 18 |
| BA.2 | 276571 | male | 47 | Mild | Fully Vaccinated | 14.8 | 6 | 16 |
| BA.2 | 276577 | male | 48 | Mild | Unvaccinated | 16.05 | 6 | 17 |
| BA.2 | 276578 | male | 47 | Mild | Unvaccinated | 15. 11 | 8 | 21 |
| BA.2 | 276587 | male | 52 | Asymptomatic | Fully Vaccinated | 13.25 | 5 | 17 |
| BA.2 | 276590 | male | 66 | Asymptomatic | Boosted | 13.46 | 5 | 20 |
| BA.2 | 276599 | male | 46 | Moderate | Fully Vaccinated | 12.7 | 6 | 17 |
| BA.2 | 276600 | male | 38 | Moderate | Boosted | 18.53 | 6 | 14 |
| BA.2 | 276601 | male | 39 | Mild | Fully Vaccinated | 15.36 | 5 | 16 |
| BA.2 | 276617 | female | 61 | Moderate | Fully Vaccinated | 13.74 | 5 | 11 |
| BA.2 | 276618 | female | 60 | Mild | Fully Vaccinated | 15.7 | 2 | 15 |
| BA.2 | 276632 | male | 70 | Moderate | Fully Vaccinated | 15.48 | 3 | 19 |
| BA.2 | 276634 | male | 29 | Mild | Boosted | 14.39 | 7 | 19 |
| BA.2 | 276640 | male | 43 | Mild | Boosted | 17.37 | 5 | 16 |
| BA.2 | 276658 | male | 26 | Mild | Boosted | 17.21 | 5 | 13 |
| BA.2 | 276662 | male | 21 | Mild | Boosted | 15.64 | 3 | 13 |
| BA.2 | 276671 | female | 37 | Mild | Boosted | 19. 1 | 4 | 21 |
| BA.2 | 276679 | male | 64 | Mild | Fully Vaccinated | 11.64 | 4 | 23 |
| BA.2 | 276717 | male | 30 | Mild | Fully Vaccinated | 13.47 | 4 | 12 |
| BA.2 | 276747 | male | 32 | Moderate | Fully Vaccinated | 19.27 | 8 | 16 |
| BA.2 | 276748 | male | 42 | Mild | Boosted | 20.08 | 2 | 14 |
| BA.2 | 276756 | female | 23 | Mild | Fully Vaccinated | 14.94 | 4 | 19 |
| BA.2 | 276770 | male | 66 | Mild | Boosted | 15.28 | 4 | 21 |

| BA.2 | 276772 | male | 14.9 | Mild | Unvaccinated | 13.4 | 2 | 19 |
| --- | --- | --- | --- | --- | --- | --- | --- | --- |
| BA.2 | 276778 | male | 23 | Mild | Fully Vaccinated | 16.42 | 3 | 12 |
| BA.2 | 276779 | male | 56 | Moderate | Fully Vaccinated | 17.71 | 7 | 14 |
| BA.2 | 276780 | male | 24 | Mild | Fully Vaccinated | 14.89 | 2 | 9 |
| BA.2 | 276783 | male | 55 | Moderate | Fully Vaccinated | 17.45 | 5 | 20 |
| BA.2 | 276788 | male | 40 | Mild | Fully Vaccinated | 18.24 | 2 | 11 |
| BA.2 | 276789 | female | 2.99 | Mild | Unvaccinated | 17. 11 | 5 | 22 |
| BA.2 | 276790 | male | 41 | Moderate | Boosted | 13.77 | 4 | 13 |
| BA.2 | 276793 | female | 53 | Mild | Fully Vaccinated | 16.5 | 2 | 16 |
| BA.2 | 276801 | female | 34 | Mild | Fully Vaccinated | 14. 11 | 4 | 15 |
| BA.2 | 276802 | female | 25 | Mild | Fully Vaccinated | 19.23 | 5 | 13 |
| BA.2 | 276803 | female | 47 | Mild | Fully Vaccinated | 13.95 | 2 | 13 |
| BA.2 | 276818 | female | 14.8 | Mild | Fully Vaccinated | 16.05 | 4 | 27 |
| BA.2 | 276819 | male | 68 | Moderate | Boosted | 16.46 | 5 | 13 |
| BA.2 | 276821 | male | 42 | Moderate | Fully Vaccinated | 15. 17 | 2 | 24 |
| BA.2 | 276826 | male | 4.99 | Mild | Unvaccinated | 16.26 | 6 | 10 |
| BA.2 | 276849 | male | 47 | Mild | Fully Vaccinated | 22.45 | 2 | 13 |
| BA.2 | 276853 | female | 45 | Mild | Boosted | 23.6 | 3 | 10 |
| BA.2 | 276855 | female | 51 | Moderate | Fully Vaccinated | 14.86 | 6 | 14 |
| BA.2 | 276857 | female | 6.58 | Mild | Unvaccinated | 14.73 | 3 | 16 |
| BA.2 | 276858 | female | 35 | Mild | Boosted | 13.72 | 3 | 16 |
| BA.2 | 276859 | female | 8.75 | Mild | Unvaccinated | 14.38 | 3 | 19 |
| BA.2 | 276860 | male | 40 | Moderate | Fully Vaccinated | 19.6 | 8 | 16 |
| BA.2 | 276861 | male | 49 | Mild | Boosted | 18.76 | 9 | 16 |
| BA.2 | 276862 | male | 55 | Moderate | Boosted | 14.06 | 2 | 15 |
| BA.2 | 276864 | female | 31 | Moderate | Unvaccinated | 16.99 | 3 | 15 |
| BA.2 | 276867 | male | 33 | Mild | Boosted | 14.86 | 3 | 8 |
| BA.2 | 276868 | female | 31 | Mild | Boosted | 18.65 | 2 | 12 |
| BA.2 | 276869 | male | 42 | Moderate | Fully Vaccinated | 12.78 | 2 | 19 |
| BA.2 | 276870 | male | 34 | Mild | Boosted | 25.43 | 9 | 14 |
| BA.2 | 276871 | male | 58 | Moderate | Boosted | 20.38 | 2 | 7 |
| BA.2 | 276872 | male | 33 | Mild | Boosted | 16.73 | 3 | 12 |

| BA.2 | 276873 | male | 49 | Mild | Fully Vaccinated | 20. 12 | 3 | 14 |
| --- | --- | --- | --- | --- | --- | --- | --- | --- |
| BA.2 | 276874 | male | 44 | Asymptomatic | Boosted | 15.06 | 2 | 13 |
| BA.2 | 276876 | male | 26 | Mild | Fully Vaccinated | 20.04 | 6 | 13 |
| BA.2 | 276879 | male | 57 | Moderate | Boosted | 20. 14 | 5 | 14 |
| BA.2 | 276880 | male | 24 | Mild | Fully Vaccinated | 15.53 | 5 | 17 |
| BA.2 | 276882 | female | 52 | Mild | Boosted | 12. 11 | 2 | 17 |
| BA.2 | 276914 | female | 28 | Mild | Fully Vaccinated | 23.34 | 9 | 15 |
| BA.2 | 276915 | female | 50 | Mild | Fully Vaccinated | 18.59 | 6 | 24 |
| BA.2 | 276931 | female | 59 | Mild | Boosted | 12.68 | 5 | 19 |
| BA.2 | 276932 | male | 2.33 | Mild | Unvaccinated | 23. 11 | 5 | 14 |
| BA.2 | 276945 | male | 28 | Moderate | Unvaccinated | 14.47 | 4 | 15 |
| BA.2 | 276950 | male | 50 | Mild | Boosted | 23.69 | 9 | 14 |
| BA.2 | 276952 | female | 5 | Mild | Unvaccinated | 16.92 | 3 | 12 |
| BA.2 | 276953 | female | 32 | Mild | Fully Vaccinated | 24.75 | 7 | 17 |
| BA.2 | 276957 | female | 39 | Mild | Boosted | 23.42 | 7 | 15 |
| BA.2 | 276958 | male | 62 | Mild | Boosted | 17.26 | 6 | 16 |
| BA.2 | 276959 | male | 33 | Mild | Boosted | 16.32 | 6 | 15 |
| BA.2 | 276966 | female | 20 | Mild | Fully Vaccinated | 15.29 | 6 | 17 |
| BA.2 | 276972 | female | 61 | Mild | Boosted | 16.84 | 2 | 13 |
| BA.2 | 276999 | male | 45 | Mild | Boosted | 16.3 | 5 | 14 |
| BA.2 | 277007 | male | 35 | Mild | Fully Vaccinated | 16.83 | 6 | 21 |
| BA.2 | 277024 | male | 12.3 | Mild | Unvaccinated | 14.53 | 3 | 15 |
| BA.2 | 277025 | female | 14.9 | Mild | Fully Vaccinated | 14.79 | 3 | 19 |
| BA.2 | 277034 | female | 2.42 | Mild | Unvaccinated | 15.23 | 3 | 14 |
| BA.2 | 277035 | female | 27 | Mild | Fully Vaccinated | 16.77 | 4 | 10 |
| BA.2 | 277037 | male | 5. 17 | Mild | Unvaccinated | 15.42 | 3 | 8 |
| BA.2 | 277038 | male | 1.29 | Asymptomatic | Unvaccinated | 22.63 | 2 | 17 |
| BA.2 | 277048 | female | 42 | Mild | Fully Vaccinated | 15.83 | 3 | 14 |
| BA.2 | 277052 | female | 63 | Mild | Boosted | 14.44 | 3 | 14 |
| BA.2 | 277053 | male | 42 | Mild | Fully Vaccinated | 12.6 | 4 | 15 |
| BA.2 | 277054 | female | 8.42 | Moderate | Unvaccinated | 19.73 | 8 | 14 |
| BA.2 | 277064 | female | 33 | Mild | Fully Vaccinated | 20.46 | 6 | 20 |

| BA.2 | 277065 | female | 7.92 | Mild | Unvaccinated | 25.02 | 6 | 12 |
| --- | --- | --- | --- | --- | --- | --- | --- | --- |
| BA.2 | 277092 | female | 61 | Moderate | Fully Vaccinated | 15. 13 | 4 | 16 |
| BA.2 | 277102 | male | 16 | Mild | Fully Vaccinated | 16. 13 | 3 | 16 |
| BA.2 | 277103 | male | 31 | Mild | Fully Vaccinated | 17.35 | 8 | 15 |
| BA.2 | 277104 | female | 30 | Mild | Fully Vaccinated | 16.6 | 3 | 15 |
| BA.2 | 277107 | female | 30 | Mild | Unvaccinated | 15.43 | 6 | 21 |
| BA.2 | 277108 | male | 0.08 | Moderate | Unvaccinated | 20.76 | 5 | 15 |
| BA.2 | 277113 | male | 14.9 | Mild | Fully Vaccinated | 17.9 | 8 | 12 |
| BA.2 | 277118 | female | 8.58 | Mild | Unvaccinated | 16.39 | 3 | 6 |
| BA.2 | 277119 | female | 29 | Moderate | Fully Vaccinated | 16.02 | 3 | 17 |
| BA.2 | 277120 | female | 1.63 | Mild | Unvaccinated | 15.75 | 3 | 8 |
| BA.2 | 277138 | male | 16 | Mild | Fully Vaccinated | 16.42 | 4 | 17 |
| BA.2 | 277152 | male | 2.55 | Mild | Unvaccinated | 20.84 | 5 | 17 |
| BA.2 | 277153 | female | 26 | Mild | Fully Vaccinated | 14.36 | 3 | 22 |
| BA.2 | 277161 | male | 36 | Moderate | Fully Vaccinated | 14.56 | 7 | 17 |
| BA.2 | 277165 | male | 5. 17 | Moderate | Unvaccinated | 23.83 | 6 | 12 |
| BA.2 | 277167 | male | 3.51 | Mild | Unvaccinated | 24.49 | 3 | 7 |
| BA.2 | 277168 | male | 59 | Mild | Fully Vaccinated | 18.87 | 2 | 13 |
| BA.2 | 277169 | female | 1.94 | Mild | Unvaccinated | 18.82 | 2 | 13 |
| BA.2 | 277170 | female | 4.06 | Mild | Fully Vaccinated | 23.02 | 3 | 14 |
| BA.2 | 277171 | female | 30 | Moderate | Fully Vaccinated | 13.66 | 3 | 23 |
| BA.2 | 277172 | female | 36 | Mild | Unvaccinated | 14.71 | 2 | 26 |
| BA.2 | 277175 | female | 54 | Moderate | Fully Vaccinated | 13.84 | 6 | 20 |
| BA.2 | 277176 | male | 34 | Mild | Fully Vaccinated | 13.66 | 2 | 20 |
| BA.2 | 277177 | male | 45 | Mild | Unvaccinated | 15. 17 | 2 | 15 |
| BA.2 | 277179 | female | 32 | Mild | Fully Vaccinated | 18. 11 | 5 | 14 |
| BA.2 | 277181 | male | 57 | Mild | Boosted | 20.67 | 8 | 16 |
| BA.2 | 277182 | male | 51 | Mild | Boosted | 22.63 | 8 | 17 |
| BA.2 | 277184 | female | 6.08 | Mild | Unvaccinated | 22.37 | 6 | 20 |
| BA.2 | 277185 | female | 31 | Mild | Boosted | 18.77 | 5 | 11 |
| BA.2 | 277187 | male | 58 | Mild | Boosted | 16.73 | 9 | 19 |
| BA.2 | 277188 | male | 10.3 | Mild | Unvaccinated | 18.07 | 2 | 14 |

| BA.2 | 277193 | female | 48 | Asymptomatic | Boosted | 13.2 | 7 | 18 |
| --- | --- | --- | --- | --- | --- | --- | --- | --- |
| BA.2 | 277194 | female | 47 | Moderate | Boosted | 15.33 | 6 | 14 |
| BA.2 | 277195 | male | 22 | Mild | Fully Vaccinated | 13.92 | 3 | 17 |
| BA.2 | 277200 | male | 23 | Moderate | Fully Vaccinated | 18.53 | 8 | 15 |
| BA.2 | 277201 | female | 35 | Mild | Fully Vaccinated | 16.58 | 5 | 12 |
| BA.2 | 277202 | female | 68 | Mild | Fully Vaccinated | 15.48 | 5 | 12 |
| BA.2 | 277204 | male | 2.33 | Asymptomatic | Unvaccinated | 13.68 | 7 | 16 |
| BA.2 | 277205 | female | 26 | Asymptomatic | Boosted | 18.41 | 6 | 16 |
| BA.2 | 277209 | female | 32 | Asymptomatic | Fully Vaccinated | 18.03 | 3 | 15 |
| BA.2 | 277210 | female | 33 | Asymptomatic | Unvaccinated | 14.7 | 3 | 18 |
| BA.2 | 277212 | male | 15 | Asymptomatic | Unvaccinated | 19.59 | 5 | 14 |
| BA.2 | 277213 | female | 26 | Mild | Boosted | 12.35 | 4 | 12 |
| BA.2 | 277218 | female | 6.67 | Mild | Unvaccinated | 23.7 | 3 | 17 |
| BA.2 | 277219 | male | 66 | Moderate | Boosted | 14.81 | 7 | 19 |
| BA.2 | 277220 | female | 10.3 | Mild | Unvaccinated | 14.5 | 3 | 13 |
| BA.2 | 277230 | male | 46 | Asymptomatic | Boosted | 18.44 | 2 | 19 |
| BA.2 | 277231 | female | 42 | Asymptomatic | Boosted | 12.24 | 2 | 14 |
| BA.2 | 277233 | female | 69 | Moderate | Fully Vaccinated | 22.42 | 3 | 17 |
| BA.2 | 277248 | male | 5.58 | Asymptomatic | Unvaccinated | 14.43 | 14 | 21 |
| BA.2 | 277250 | male | 15 | Asymptomatic | Unvaccinated | 14.28 | 14 | 21 |
| BA.2 | 277252 | male | 53 | Mild | Fully Vaccinated | 17. 18 | 8 | 21 |
| BA.2 | 277281 | female | 29 | Mild | Boosted | 11.73 | 3 | 20 |
| BA.2 | 277282 | male | 1.59 | Mild | Unvaccinated | 17.66 | 9 | 20 |
| BA.2 | 277283 | male | 31 | Mild | Boosted | 22.3 | 2 | 9 |
| BA.2 | 277284 | female | 3.56 | Mild | Unvaccinated | 17. 17 | 6 | 12 |
| BA.2 | 277287 | female | 34 | Moderate | Fully Vaccinated | 14. 17 | 3 | 11 |
| BA.2 | 277288 | male | 5.83 | Mild | Unvaccinated | 18.46 | 3 | 12 |
| BA.2 | 277289 | female | 50 | Mild | Boosted | 19.84 | 5 | 16 |
| BA.2 | 277290 | male | 50 | Moderate | Boosted | 20. 11 | 7 | 16 |
| BA.2 | 277291 | male | 53 | Mild | Boosted | 17.51 | 9 | 18 |
| BA.2 | 277292 | male | 56 | Moderate | Boosted | 21.32 | 4 | 15 |
| BA.2 | 277293 | male | 52 | Mild | Boosted | 14.78 | 4 | 15 |

| BA.2 | 277296 | male | 55 | Mild | Fully Vaccinated | 16.26 | 7 | 15 |
| --- | --- | --- | --- | --- | --- | --- | --- | --- |
| BA.2 | 277297 | female | 23 | Mild | Unvaccinated | 19.54 | 7 | 26 |
| BA.2 | 277298 | male | 1.52 | Mild | Unvaccinated | 12.87 | 6 | 18 |
| BA.2 | 277299 | male | 13. 1 | Mild | Fully Vaccinated | 20.06 | 3 | 12 |
| BA.2 | 277300 | female | 40 | Mild | Boosted | 12.01 | 3 | 19 |
| BA.2 | 277301 | male | 24 | Mild | Boosted | 23.98 | 8 | 14 |
| BA.2 | 277302 | female | 26 | Mild | Unvaccinated | 14.45 | 3 | 17 |
| BA.2 | 277305 | male | 54 | Mild | Boosted | 14.54 | 3 | 21 |
| BA.2 | 277306 | female | 34 | Mild | Boosted | 18.75 | 3 | 19 |
| BA.2 | 277312 | male | 23 | Mild | Fully Vaccinated | 14.78 | 7 | 14 |
| BA.2 | 277316 | female | 57 | Mild | Boosted | 14.72 | 4 | 20 |
| BA.2 | 277318 | male | 67 | Asymptomatic | Boosted | 14.79 | 6 | 14 |
| BA.2 | 277319 | female | 29 | Mild | Fully Vaccinated | 15.23 | 4 | 18 |
| BA.2 | 277321 | female | 30 | Mild | Boosted | 17. 14 | 4 | 9 |
| BA.2 | 277329 | male | 60 | Moderate | Boosted | 16.99 | 7 | 17 |
| BA.2 | 277330 | male | 2.99 | Asymptomatic | Unvaccinated | 15. 15 | 3 | 13 |
| BA.2 | 277338 | female | 14.9 | Mild | Unvaccinated | 14.86 | 2 | 14 |
| BA.2 | 277339 | female | 19 | Mild | Unvaccinated | 23.03 | 2 | 15 |
| BA.2 | 277340 | male | 2.31 | Mild | Unvaccinated | 14.91 | 6 | 14 |
| BA.2 | 277342 | female | 29 | Moderate | Fully Vaccinated | 12.95 | 3 | 15 |
| BA.2 | 277343 | female | 34 | Mild | Boosted | 13.35 | 2 | 13 |
| BA.2 | 277344 | female | 9.75 | Moderate | Unvaccinated | 16.79 | 2 | 15 |
| BA.2 | 277345 | female | 26 | Mild | Fully Vaccinated | 11.46 | 3 | 22 |
| BA.2 | 277354 | male | 1.43 | Mild | Unvaccinated | 15.2 | 3 | 12 |
| BA.2 | 277355 | female | 40 | Mild | Boosted | 16.53 | 6 | 13 |
| BA.2 | 277357 | female | 26 | Mild | Fully Vaccinated | 16.49 | 7 | 16 |
| BA.2 | 277370 | male | 11.2 | Mild | Fully Vaccinated | 21.65 | 4 | 9 |
| BA.2 | 277373 | male | 56 | Mild | Boosted | 21.44 | 8 | 16 |
| BA.2 | 277374 | male | 34 | Mild | Boosted | 21.02 | 8 | 16 |
| BA.2 | 277387 | male | 3. 12 | Moderate | Unvaccinated | 14.89 | 3 | 13 |
| BA.2 | 277394 | female | 15 | Moderate | Fully Vaccinated | 19.81 | 5 | 17 |
| BA.2 | 277395 | male | 14.2 | Asymptomatic | Fully Vaccinated | 24.44 | 8 | 16 |

| BA.2 | 277403 | female | 52 | Mild | Boosted | 16. 17 | 6 | 13 |
| --- | --- | --- | --- | --- | --- | --- | --- | --- |
| BA.2 | 277406 | female | 32 | Mild | Fully Vaccinated | 12.88 | 3 | 18 |
| BA.2 | 277407 | female | 17 | Mild | Fully Vaccinated | 16.78 | 5 | 15 |
| BA.2 | 277410 | female | 1.1 | Mild | Unvaccinated | 19.84 | 3 | 12 |
| BA.2 | 277413 | female | 27 | Mild | Fully Vaccinated | 15.42 | 3 | 18 |
| BA.2 | 277414 | female | 15 | Mild | Fully Vaccinated | 16.6 | 6 | 11 |
| BA.2 | 277416 | female | 27 | Moderate | Unvaccinated | 16.02 | 3 | 17 |
| BA.2 | 277418 | male | 7.67 | Moderate | Unvaccinated | 12.33 | 4 | 10 |
| BA.2 | 277421 | female | 30 | Mild | Fully Vaccinated | 11.26 | 4 | 16 |
| BA.2 | 277422 | female | 3.22 | Mild | Unvaccinated | 12.72 | 3 | 5 |
| BA.2 | 277424 | female | 30 | Moderate | Boosted | 25.29 | 3 | 16 |
| BA.2 | 277425 | male | 5.5 | Mild | Unvaccinated | 20.05 | 6 | 13 |
| BA.2 | 277426 | male | 33 | Mild | Boosted | 16. 1 | 6 | 11 |
| BA.2 | 277427 | female | 6.33 | Mild | Unvaccinated | 23. 13 | 5 | 12 |
| BA.2 | 277428 | female | 27 | Mild | Fully Vaccinated | 14.41 | 6 | 14 |
| BA.2 | 277437 | male | 58 | Mild | Fully Vaccinated | 21.44 | 9 | 16 |
| BA.2 | 277439 | male | 15 | Asymptomatic | Fully Vaccinated | 18.08 | 3 | 14 |
| BA.2 | 277440 | female | 41 | Asymptomatic | Boosted | 19.93 | 3 | 14 |
| BA.2 | 277442 | female | 22 | Mild | Boosted | 20.2 | 7 | 13 |
| BA.2 | 277443 | female | 47 | Moderate | Boosted | 21.24 | 7 | 10 |
| BA.2 | 277445 | female | 73 | Mild | Fully Vaccinated | 13.43 | 4 | 15 |
| BA.2 | 277448 | male | 3. 19 | Mild | Unvaccinated | 19.87 | 6 | 10 |
| BA.2 | 277449 | female | 2.98 | Asymptomatic | Unvaccinated | 19.94 | 4 | 16 |
| BA.2 | 277450 | male | 7.5 | Asymptomatic | Unvaccinated | 20.67 | 8 | 11 |
| BA.2 | 277451 | female | 4.38 | Asymptomatic | Unvaccinated | 22.74 | 8 | 12 |
| BA.2 | 277452 | male | 31 | Asymptomatic | Fully Vaccinated | 18.34 | 6 | 12 |
| BA.2 | 277453 | male | 24 | Mild | Fully Vaccinated | 12.51 | 3 | 14 |
| BA.2 | 277454 | female | 28 | Mild | Boosted | 15.24 | 3 | 16 |
| BA.2 | 277455 | male | 7.75 | Mild | Unvaccinated | 15.84 | 2 | 17 |
| BA.2 | 277456 | male | 1.82 | Asymptomatic | Unvaccinated | 26.25 | 11 | 15 |
| BA.2 | 277457 | male | 28 | Asymptomatic | Boosted | 16.08 | 2 | 16 |
| BA.2 | 277458 | female | 10.8 | Mild | Unvaccinated | 21. 15 | 8 | 14 |

| BA.2 | 277459 | female | 28 | Mild | Fully Vaccinated | 14.57 | 6 | 23 |
| --- | --- | --- | --- | --- | --- | --- | --- | --- |
| BA.2 | 277460 | male | 55 | Asymptomatic | Fully Vaccinated | 16.04 | 6 | 19 |
| BA.2 | 277462 | female | 47 | Mild | Unvaccinated | 18.93 | 7 | 12 |
| BA.2 | 277463 | female | 49 | Moderate | Boosted | 16. 12 | 5 | 10 |
| BA.2 | 277464 | female | 52 | Mild | Boosted | 26.51 | 2 | 17 |
| BA.2 | 277465 | male | 35 | Mild | Boosted | 15.73 | 2 | 13 |
| BA.2 | 277466 | female | 52 | Mild | Fully Vaccinated | 16. 17 | 2 | 14 |
| BA.2 | 277467 | male | 61 | Mild | Boosted | 16. 18 | 3 | 15 |
| BA.2 | 277468 | female | 54 | Moderate | Boosted | 15.32 | 6 | 18 |
| BA.2 | 277469 | female | 48 | Moderate | Fully Vaccinated | 18.32 | 3 | 12 |
| BA.2 | 277470 | female | 10.9 | Mild | Unvaccinated | 17.42 | 3 | 12 |
| BA.2 | 277474 | male | 22 | Mild | Fully Vaccinated | 19.31 | 2 | 17 |
| BA.2 | 277477 | male | 6.58 | Moderate | Unvaccinated | 20.74 | 4 | 16 |
| BA.2 | 277484 | male | 45 | Mild | Boosted | 16.05 | 6 | 14 |
| BA.2 | 277485 | female | 40 | Asymptomatic | Boosted | 17.65 | 3 | 14 |
| BA.2 | 277487 | female | 16 | Moderate | Unvaccinated | 17.44 | 3 | 15 |
| BA.2 | 277488 | male | 27 | Mild | Boosted | 14.88 | 4 | 14 |
| BA.2 | 277489 | female | 42 | Moderate | Boosted | 15.67 | 3 | 14 |
| BA.2 | 277491 | male | 43 | Mild | Fully Vaccinated | 14.29 | 7 | 20 |
| BA.2 | 277496 | male | 24 | Mild | Fully Vaccinated | 20.62 | 6 | 12 |
| BA.2 | 277510 | male | 30 | Moderate | Fully Vaccinated | 20.6 | 7 | 12 |
| BA.2 | 277511 | female | 46 | Asymptomatic | Boosted | 17.91 | 3 | 11 |
| BA.2 | 277514 | female | 58 | Mild | Fully Vaccinated | 15.8 | 3 | 13 |
| BA.2 | 277518 | male | 56 | Asymptomatic | Boosted | 15.42 | 5 | 15 |
| BA.2 | 277524 | male | 55 | Mild | Boosted | 15.28 | 3 | 14 |
| BA.2 | 277536 | female | 52 | Mild | Boosted | 13.96 | 2 | 18 |
| BA.2 | 277538 | male | 4.71 | Mild | Unvaccinated | 25.61 | 9 | 17 |
| BA.2 | 277539 | female | 45 | Mild | Fully Vaccinated | 16.45 | 5 | 14 |
| BA.2 | 277541 | female | 36 | Asymptomatic | Boosted | 25.89 | 9 | 16 |
| BA.2 | 277544 | female | 53 | Asymptomatic | Fully Vaccinated | 13. 15 | 7 | 20 |
| BA.2 | 277545 | female | 30 | Asymptomatic | Boosted | 15.07 | 3 | 16 |
| BA.2 | 277549 | female | 11.8 | Asymptomatic | Fully Vaccinated | 23. 1 | 10 | 18 |

| BA.2 | 277550 | male | 68 | Mild | Boosted | 19.74 | 8 | 14 |
| --- | --- | --- | --- | --- | --- | --- | --- | --- |
| BA.2 | 277563 | male | 46 | Mild | Fully Vaccinated | 16.56 | 3 | 11 |
| BA.2 | 277565 | male | 39 | Moderate | Boosted | 25.35 | 3 | 13 |
| BA.2 | 277569 | female | 2.08 | Mild | Unvaccinated | 16.4 | 2 | 20 |
| BA.2 | 277584 | male | 17 | Mild | Fully Vaccinated | 17. 13 | 4 | 14 |
| BA.2 | 277585 | male | 47 | Moderate | Fully Vaccinated | 13.4 | 6 | 17 |
| BA.2 | 277589 | male | 65 | Asymptomatic | Fully Vaccinated | 16.05 | 6 | 15 |
| BA.2 | 277592 | male | 37 | Asymptomatic | Unvaccinated | 13.76 | 5 | 24 |
| BA.2 | 277593 | male | 63 | Asymptomatic | Boosted | 13. 18 | 6 | 14 |
| BA.2 | 277595 | male | 32 | Asymptomatic | Boosted | 17 | 3 | 14 |
| BA.2 | 277596 | male | 32 | Asymptomatic | Fully Vaccinated | 20.36 | 9 | 13 |
| BA.2 | 277599 | female | 30 | Mild | Unvaccinated | 16.72 | 6 | 18 |
| BA.2 | 277600 | female | 3.99 | Asymptomatic | Unvaccinated | 23.37 | 6 | 19 |
| BA.2 | 277601 | male | 8.92 | Mild | Unvaccinated | 21.29 | 3 | 15 |
| BA.2 | 277602 | male | 58 | Asymptomatic | Unvaccinated | 16.72 | 10 | 17 |
| BA.2 | 277604 | male | 67 | Asymptomatic | Fully Vaccinated | 18.02 | 3 | 14 |
| BA.2 | 277607 | female | 65 | Asymptomatic | Boosted | 16.23 | 3 | 16 |
| BA.2 | 277610 | male | 52 | Asymptomatic | Fully Vaccinated | 17.08 | 9 | 16 |
| BA.2 | 277611 | female | 29 | Asymptomatic | Fully Vaccinated | 17.25 | 2 | 14 |
| BA.2 | 277612 | male | 71 | Asymptomatic | Fully Vaccinated | 14.68 | 2 | 17 |
| BA.2 | 277613 | male | 32 | Asymptomatic | Fully Vaccinated | 19.29 | 2 | 9 |
| BA.2 | 277614 | female | 49 | Asymptomatic | Boosted | 23.56 | 13 | 16 |
| BA.2 | 277616 | male | 49 | Mild | Boosted | 16.27 | 3 | 13 |
| BA.2 | 277620 | male | 60 | Moderate | Boosted | 20. 14 | 6 | 15 |
| BA.2 | 277621 | male | 66 | Asymptomatic | Boosted | 22 | 2 | 14 |
| BA.2 | 277622 | female | 47 | Asymptomatic | Fully Vaccinated | 24.68 | 2 | 22 |
| BA.2 | 277625 | female | 37 | Asymptomatic | Boosted | 15.23 | 5 | 20 |
| BA.2 | 277627 | male | 22 | Asymptomatic | Fully Vaccinated | 19.28 | 2 | 16 |
| BA.2 | 277628 | male | 50 | Mild | Boosted | 15.98 | 4 | 12 |
| BA.2 | 277629 | female | 23 | Moderate | Fully Vaccinated | 19.29 | 4 | 19 |
| BA.2 | 277632 | female | 28 | Asymptomatic | Boosted | 25.27 | 3 | 14 |
| BA.2 | 277635 | female | 25 | Asymptomatic | Fully Vaccinated | 25.93 | 3 | 9 |

| BA.2 | 277637 | male | 2.89 | Asymptomatic | Unvaccinated | 24.37 | 3 | 9 |
| --- | --- | --- | --- | --- | --- | --- | --- | --- |
| BA.2 | 277638 | male | 28 | Asymptomatic | Unvaccinated | 20.86 | 3 | 14 |
| BA.2 | 277640 | male | 51 | Mild | Boosted | 15.09 | 3 | 11 |
| BA.2 | 277642 | male | 17 | Mild | Fully Vaccinated | 15.97 | 6 | 14 |
| BA.2 | 277643 | female | 30 | Mild | Boosted | 22.82 | 3 | 14 |
| BA.2 | 277648 | male | 56 | Mild | Boosted | 14.41 | 3 | 14 |
| BA.2 | 277650 | male | 41 | Asymptomatic | Unvaccinated | 14.74 | 3 | 20 |
| BA.2 | 277654 | male | 20 | Mild | Fully Vaccinated | 19.6 | 6 | 14 |
| BA.2 | 277655 | female | 25 | Mild | Fully Vaccinated | 16.54 | 4 | 11 |
| BA.2 | 277660 | female | 19 | Mild | Boosted | 19.86 | 3 | 15 |
| BA.2 | 277661 | male | 53 | Mild | Boosted | 16.06 | 4 | 23 |
| BA.2 | 277662 | male | 49 | Mild | Boosted | 16.01 | 3 | 15 |
| BA.2 | 277663 | male | 51 | Mild | Fully Vaccinated | 26.59 | 5 | 13 |
| BA.2 | 277668 | male | 44 | Asymptomatic | Boosted | 21.46 | 3 | 16 |
| BA.2 | 277671 | female | 54 | Asymptomatic | Boosted | 15.63 | 4 | 17 |
| BA.2 | 277677 | female | 60 | Mild | Fully Vaccinated | 14.63 | 3 | 14 |
| BA.2 | 277689 | male | 3.99 | Mild | Unvaccinated | 24.47 | 8 | 14 |
| BA.2 | 277690 | male | 3.99 | Mild | Unvaccinated | 22. 17 | 2 | 8 |
| BA.2 | 277700 | male | 49 | Mild | Boosted | 16.54 | 3 | 18 |
| BA.2 | 277702 | female | 26 | Mild | Boosted | 18.79 | 5 | 16 |
| BA.2 | 277703 | female | 28 | Mild | Fully Vaccinated | 15.2 | 3 | 11 |
| BA.2 | 277704 | female | 42 | Mild | Boosted | 20.36 | 4 | 20 |
| BA.2 | 277705 | male | 39 | Moderate | Fully Vaccinated | 15.47 | 4 | 21 |
| BA.2 | 277706 | female | 45 | Mild | Fully Vaccinated | 17.9 | 3 | 18 |
| BA.2 | 277709 | female | 8.25 | Mild | Fully Vaccinated | 16. 1 | 3 | 18 |
| BA.2 | 277710 | female | 0.66 | Moderate | Unvaccinated | 26.52 | 3 | 19 |
| BA.2 | 277711 | female | 39 | Moderate | Unvaccinated | 15.82 | 3 | 21 |
| BA.2 | 277712 | female | 31 | Mild | Boosted | 21.24 | 8 | 22 |
| BA.2 | 277714 | female | 41 | Asymptomatic | Fully Vaccinated | 11.24 | 4 | 14 |
| BA.2 | 277715 | male | 53 | Asymptomatic | Boosted | 16.89 | 8 | 15 |
| BA.2 | 277716 | female | 54 | Mild | Fully Vaccinated | 13. 14 | 3 | 18 |
| BA.2 | 277718 | female | 30 | Mild | Fully Vaccinated | 19.36 | 7 | 13 |

| BA.2 | 277720 | male | 65 | Mild | Boosted | 22. 16 | 5 | 13 |
| --- | --- | --- | --- | --- | --- | --- | --- | --- |
| BA.2 | 277722 | female | 58 | Moderate | Fully Vaccinated | 16.4 | 5 | 24 |
| BA.2 | 277723 | male | 36 | Mild | Fully Vaccinated | 19.04 | 4 | 14 |
| BA.2 | 277724 | male | 52 | Mild | Boosted | 16.32 | 5 | 13 |
| BA.2 | 277725 | male | 32 | Mild | Boosted | 15.02 | 5 | 13 |
| BA.2 | 277726 | female | 39 | Mild | Fully Vaccinated | 17.54 | 3 | 14 |
| BA.2 | 277727 | male | 63 | Mild | Fully Vaccinated | 16. 16 | 4 | 20 |
| BA.2 | 277728 | female | 64 | Mild | Unvaccinated | 15.31 | 5 | 22 |
| BA.2 | 277730 | female | 48 | Mild | Fully Vaccinated | 15.26 | 4 | 17 |
| BA.2 | 277731 | male | 46 | Asymptomatic | Boosted | 14.83 | 3 | 12 |
| BA.2 | 277733 | female | 47 | Asymptomatic | Boosted | 14.92 | 3 | 18 |
| BA.2 | 277734 | male | 50 | Moderate | Fully Vaccinated | 17.02 | 3 | 24 |
| BA.2 | 277736 | male | 42 | Moderate | Boosted | 19. 14 | 4 | 20 |
| BA.2 | 277738 | female | 57 | Mild | Boosted | 14.51 | 5 | 19 |
| BA.2 | 277739 | male | 15 | Moderate | Fully Vaccinated | 19.85 | 4 | 12 |
| BA.2 | 277740 | male | 54 | Mild | Fully Vaccinated | 15.81 | 3 | 15 |
| BA.2 | 277741 | male | 48 | Mild | Fully Vaccinated | 19.32 | 4 | 20 |
| BA.2 | 277742 | female | 42 | Mild | Boosted | 21.05 | 5 | 13 |
| BA.2 | 277743 | female | 50 | Moderate | Fully Vaccinated | 20.51 | 4 | 20 |
| BA.2 | 277744 | male | 55 | Asymptomatic | Fully Vaccinated | 25.64 | 2 | 6 |
| BA.2 | 277747 | male | 22 | Mild | Boosted | 16.88 | 4 | 13 |
| BA.2 | 277754 | female | 48 | Asymptomatic | Fully Vaccinated | 23.88 | 3 | 18 |
| BA.2 | 277756 | male | 84 | Mild | Fully Vaccinated | 17.04 | 2 | 13 |
| BA.2 | 277758 | female | 53 | Mild | Boosted | 17. 12 | 3 | 13 |
| BA.2 | 277759 | female | 29 | Mild | Fully Vaccinated | 15.95 | 4 | 19 |
| BA.2 | 277761 | male | 59 | Moderate | Boosted | 15.61 | 2 | 15 |
| BA.2 | 277762 | male | 51 | Mild | Fully Vaccinated | 16.28 | 4 | 11 |
| BA.2 | 277763 | male | 54 | Mild | Fully Vaccinated | 15. 19 | 2 | 11 |
| BA.2 | 277764 | male | 33 | Mild | Boosted | 14.69 | 3 | 19 |
| BA.2 | 277768 | female | 26 | Mild | Boosted | 16.24 | 3 | 12 |
| BA.2 | 277770 | male | 33 | Asymptomatic | Unvaccinated | 15.08 | 2 | 17 |
| BA.2 | 277774 | male | 30 | Moderate | Boosted | 14.59 | 3 | 15 |

| BA.2 | 277784 | female | 75 | Asymptomatic | Fully Vaccinated | 14.51 | 2 | 12 |
| --- | --- | --- | --- | --- | --- | --- | --- | --- |
| BA.2 | 277786 | male | 32 | Asymptomatic | Boosted | 21.75 | 2 | 19 |
| BA.2 | 277814 | female | 43 | Mild | Boosted | 12.07 | 4 | 20 |
| BA.2 | 277815 | male | 43 | Asymptomatic | Boosted | 25. 17 | 8 | 19 |
| BA.2 | 277822 | female | 1.99 | Mild | Unvaccinated | 16.09 | 3 | 14 |
| BA.2 | 277825 | male | 76 | Asymptomatic | Fully Vaccinated | 12.99 | 5 | 16 |
| BA.2 | 277826 | male | 45 | Mild | Boosted | 13.29 | 3 | 15 |
| BA.2 | 277828 | female | 59 | Moderate | Fully Vaccinated | 13.66 | 4 | 16 |
| BA.2 | 277829 | male | 43 | Mild | Boosted | 14.79 | 3 | 16 |
| BA.2 | 277833 | female | 65 | Mild | Boosted | 11. 19 | 5 | 17 |
| BA.2 | 277834 | male | 32 | Mild | Boosted | 23.32 | 8 | 19 |
| BA.2 | 277837 | male | 28 | Mild | Fully Vaccinated | 15.87 | 3 | 11 |
| BA.2 | 277851 | female | 51 | Mild | Boosted | 14.54 | 4 | 16 |
| BA.2 | 277852 | female | 27 | Mild | Boosted | 11.45 | 4 | 18 |
| BA.2 | 277853 | female | 35 | Moderate | Boosted | 11.05 | 3 | 14 |
| BA.2 | 277854 | female | 0.67 | Moderate | Unvaccinated | 25 | 5 | 19 |
| BA.2 | 277857 | male | 3.99 | Mild | Fully Vaccinated | 25.57 | 12 | 13 |
| BA.2 | 277858 | female | 5.92 | Mild | Unvaccinated | 14.46 | 3 | 8 |
| BA.2 | 277859 | female | 34 | Mild | Boosted | 18.29 | 3 | 15 |
| BA.2 | 277860 | male | 7.83 | Mild | Fully Vaccinated | 12.2 | 3 | 13 |
| BA.2 | 277861 | male | 35 | Mild | Boosted | 26.37 | 9 | 15 |
| BA.2 | 277863 | female | 48 | Mild | Boosted | 11.64 | 4 | 16 |
| BA.2 | 277864 | female | 14.8 | Mild | Fully Vaccinated | 11.73 | 3 | 17 |
| BA.2 | 277871 | male | 31 | Moderate | Fully Vaccinated | 21.99 | 3 | 15 |
| BA.2 | 277873 | male | 38 | Mild | Boosted | 15.77 | 4 | 17 |
| BA.2 | 277877 | male | 34 | Mild | Boosted | 19. 15 | 3 | 17 |
| BA.2 | 277879 | male | 9.58 | Mild | Fully Vaccinated | 11.86 | 3 | 11 |
| BA.2 | 277880 | female | 35 | Moderate | Fully Vaccinated | 21.39 | 3 | 17 |
| BA.2 | 277881 | male | 39 | Mild | Boosted | 24.01 | 5 | 11 |
| BA.2 | 277882 | male | 68 | Mild | Fully Vaccinated | 25.37 | 2 | 16 |
| BA.2 | 277883 | female | 23 | Mild | Boosted | 19.97 | 3 | 11 |
| BA.2 | 277884 | male | 88 | Mild | Fully Vaccinated | 12.05 | 3 | 21 |

| BA.2 | 277886 | female | 63 | Asymptomatic | Fully Vaccinated | 17 | 3 | 19 |
| --- | --- | --- | --- | --- | --- | --- | --- | --- |
| BA.2 | 277890 | female | 32 | Mild | Fully Vaccinated | 11.72 | 2 | 15 |
| BA.2 | 277891 | female | 31 | Mild | Boosted | 13.04 | 2 | 13 |
| BA.2 | 277892 | male | 10.4 | Moderate | Unvaccinated | 14.46 | 2 | 12 |
| BA.2 | 277893 | male | 1.95 | Mild | Unvaccinated | 13.01 | 3 | 16 |
| BA.2 | 277894 | male | 27 | Mild | Boosted | 25. 15 | 2 | 11 |
| BA.2 | 277895 | male | 20 | Mild | Unvaccinated | 18.6 | 5 | 18 |
| BA.2 | 277897 | male | 20 | Mild | Unvaccinated | 20.03 | 7 | 15 |
| BA.2 | 277898 | male | 20 | Mild | Unvaccinated | 20.61 | 5 | 19 |
| BA.2 | 277900 | female | 12.4 | Mild | Fully Vaccinated | 13.67 | 2 | 18 |
| BA.2 | 277901 | male | 42 | Mild | Fully Vaccinated | 17.9 | 3 | 18 |
| BA.2 | 277902 | male | 59 | Mild | Boosted | 13. 15 | 4 | 15 |
| BA.2 | 277904 | male | 20 | Mild | Unvaccinated | 17.91 | 6 | 12 |
| BA.2 | 277908 | male | 20 | Asymptomatic | Unvaccinated | 17.03 | 3 | 14 |
| BA.2 | 277911 | male | 36 | Mild | Boosted | 19. 15 | 11 | 19 |
| BA.2 | 277913 | male | 61 | Mild | Fully Vaccinated | 20.77 | 2 | 16 |
| BA.2 | 277914 | male | 49 | Mild | Fully Vaccinated | 20.76 | 3 | 12 |
| BA.2 | 277915 | male | 13.5 | Mild | Unvaccinated | 15.74 | 2 | 12 |
| BA.2 | 277916 | female | 53 | Mild | Fully Vaccinated | 14.72 | 2 | 17 |
| BA.2 | 277917 | male | 31 | Mild | Boosted | 18.68 | 8 | 14 |
| BA.2 | 277920 | male | 26 | Moderate | Unvaccinated | 16.5 | 8 | 18 |
| BA.2 | 277921 | male | 26 | Mild | Fully Vaccinated | 17.62 | 2 | 17 |
| BA.2 | 277922 | male | 35 | Moderate | Boosted | 25. 15 | 3 | 18 |
| BA.2 | 277923 | female | 57 | Asymptomatic | Boosted | 13.74 | 4 | 20 |
| BA.2 | 277924 | female | 83 | Asymptomatic | Unvaccinated | 24.27 | 5 | 23 |
| BA.2 | 277934 | male | 72 | Mild | Fully Vaccinated | 14.79 | 6 | 22 |
| BA.2 | 277947 | female | 47 | Mild | Fully Vaccinated | 14.93 | 8 | 16 |
| BA.2 | 277950 | female | 36 | Mild | Fully Vaccinated | 15.69 | 5 | 20 |
| BA.2 | 277951 | male | 2.82 | Mild | Unvaccinated | 26.57 | 4 | 19 |
| BA.2 | 277952 | female | 7.42 | Mild | Unvaccinated | 15.52 | 3 | 10 |
| BA.2 | 277954 | male | 41 | Mild | Boosted | 10.74 | 3 | 15 |
| BA.2 | 277961 | female | 49 | Moderate | Fully Vaccinated | 12.87 | 4 | 17 |

| BA.2 | 277962 | male | 23 | Mild | Boosted | 11.65 | 5 | 17 |
| --- | --- | --- | --- | --- | --- | --- | --- | --- |
| BA.2 | 277965 | female | 2 | Mild | Unvaccinated | 24.37 | 4 | 15 |
| BA.2 | 277966 | male | 41 | Mild | Boosted | 21.3 | 3 | 19 |
| BA.2 | 277968 | female | 14. 1 | Mild | Fully Vaccinated | 21. 12 | 9 | 15 |
| BA.2 | 277970 | male | 20 | Mild | Unvaccinated | 22. 19 | 7 | 10 |
| BA.2 | 277974 | male | 20 | Mild | Unvaccinated | 15.58 | 5 | 16 |
| BA.2 | 277977 | male | 20 | Mild | Unvaccinated | 20.8 | 8 | 19 |
| BA.2 | 277981 | female | 12.9 | Mild | Unvaccinated | 13.77 | 4 | 17 |
| BA.2 | 277982 | female | 10.9 | Mild | Fully Vaccinated | 14.66 | 4 | 18 |
| BA.2 | 277986 | male | 38 | Mild | Fully Vaccinated | 14.72 | 7 | 18 |
| BA.2 | 277987 | female | 15 | Moderate | Unvaccinated | 17.28 | 3 | 19 |
| BA.2 | 277988 | female | 34 | Mild | Fully Vaccinated | 23.97 | 3 | 18 |
| BA.2 | 277991 | male | 46 | Moderate | Boosted | 16.93 | 3 | 16 |
| BA.2 | 277993 | female | 29 | Moderate | Fully Vaccinated | 14.62 | 3 | 18 |
| BA.2 | 277996 | male | 24 | Mild | Boosted | 20.34 | 4 | 11 |
| BA.2 | 277997 | male | 60 | Mild | Fully Vaccinated | 17.49 | 3 | 16 |
| BA.2 | 278001 | male | 68 | Mild | Fully Vaccinated | 18.38 | 4 | 15 |
| BA.2 | 278003 | male | 4.98 | Mild | Unvaccinated | 14.41 | 3 | 16 |
| BA.2 | 278004 | male | 5.92 | Mild | Unvaccinated | 22.43 | 3 | 12 |
| BA.2 | 278008 | female | 42 | Moderate | Fully Vaccinated | 13.66 | 3 | 16 |
| BA.2 | 278009 | male | 3.1 | Mild | Unvaccinated | 16.91 | 3 | 14 |
| BA.2 | 278010 | female | 45 | Mild | Boosted | 16.98 | 4 | 18 |
| BA.2 | 278013 | male | 52 | Mild | Fully Vaccinated | 20.22 | 11 | 21 |
| BA.2 | 278014 | female | 38 | Mild | Fully Vaccinated | 26.52 | 3 | 18 |
| BA.2 | 278015 | male | 58 | Moderate | Boosted | 12.86 | 4 | 20 |
| BA.2 | 278017 | male | 16 | Mild | Fully Vaccinated | 22. 16 | 3 | 12 |
| BA.2 | 278019 | female | 15 | Mild | Fully Vaccinated | 18.25 | 5 | 11 |
| BA.2 | 278020 | male | 7.33 | Asymptomatic | Unvaccinated | 26 | 3 | 12 |
| BA.2 | 278021 | male | 4.41 | Mild | Unvaccinated | 13.92 | 4 | 12 |
| BA.2 | 278023 | male | 25 | Mild | Unvaccinated | 17.69 | 4 | 15 |
| BA.2 | 278024 | male | 27 | Mild | Unvaccinated | 16.73 | 3 | 14 |
| BA.2 | 278026 | female | 27 | Mild | Fully Vaccinated | 15. 14 | 3 | 17 |

| BA.2 | 278029 | female | 26 | Mild | Fully Vaccinated | 19.66 | 3 | 18 |
| --- | --- | --- | --- | --- | --- | --- | --- | --- |
| BA.2 | 278036 | female | 42 | Mild | Boosted | 24.98 | 9 | 15 |
| BA.2 | 278037 | male | 25 | Mild | Fully Vaccinated | 14. 12 | 5 | 22 |
| BA.2 | 278038 | female | 58 | Asymptomatic | Unvaccinated | 14.61 | 3 | 18 |
| BA.2 | 278039 | female | 12.9 | Mild | Unvaccinated | 14.58 | 4 | 14 |
| BA.2 | 278056 | female | 38 | Moderate | Fully Vaccinated | 13.04 | 4 | 18 |
| BA.2 | 278063 | male | 3.53 | Mild | Unvaccinated | 20. 1 | 5 | 18 |
| BA.2 | 278076 | female | 3.01 | Mild | Unvaccinated | 16.85 | 4 | 14 |
| BA.2 | 278078 | female | 25 | Mild | Fully Vaccinated | 17.79 | 3 | 13 |
| BA.2 | 278082 | female | 41 | Mild | Boosted | 11.46 | 4 | 16 |
| BA.2 | 278089 | male | 20 | Mild | Unvaccinated | 21.76 | 6 | 18 |
| BA.2 | 278093 | male | 62 | Moderate | Fully Vaccinated | 16.53 | 5 | 17 |
| BA.2 | 278097 | female | 47 | Mild | Boosted | 10.82 | 3 | 13 |
| BA.2 | 278101 | female | 0.55 | Mild | Unvaccinated | 12.44 | 3 | 15 |
| BA.2 | 278102 | male | 28 | Moderate | Boosted | 12.35 | 5 | 17 |
| BA.2 | 278115 | male | 6.42 | Mild | Unvaccinated | 17.59 | 4 | 14 |
| BA.2 | 278116 | female | 32 | Mild | Fully Vaccinated | 16.35 | 5 | 19 |
| BA.2 | 278117 | male | 21 | Moderate | Boosted | 11.46 | 3 | 18 |
| BA.2 | 278118 | female | 39 | Mild | Boosted | 19.01 | 5 | 15 |
| BA.2 | 278119 | male | 30 | Mild | Fully Vaccinated | 13.21 | 3 | 16 |
| BA.2 | 278120 | male | 30 | Mild | Boosted | 16.31 | 3 | 13 |
| BA.2 | 278121 | male | 35 | Mild | Boosted | 12.93 | 3 | 18 |
| BA.2 | 278122 | female | 42 | Asymptomatic | Fully Vaccinated | 20.24 | 6 | 17 |
| BA.2 | 278126 | male | 30 | Mild | Fully Vaccinated | 14.24 | 6 | 17 |
| BA.2 | 278128 | female | 54 | Mild | Boosted | 12.77 | 4 | 19 |
| BA.2 | 278129 | male | 54 | Mild | Boosted | 17.64 | 4 | 19 |
| BA.2 | 278131 | male | 24 | Mild | Unvaccinated | 16.99 | 2 | 18 |
| BA.2 | 278132 | male | 24 | Mild | Unvaccinated | 18.36 | 5 | 19 |
| BA.2 | 278133 | male | 26 | Asymptomatic | Unvaccinated | 20.38 | 2 | 5 |
| BA.2 | 278135 | male | 21 | Asymptomatic | Unvaccinated | 19. 16 | 2 | 11 |
| BA.2 | 278165 | male | 31 | Mild | Fully Vaccinated | 18.27 | 4 | 14 |
| BA.2 | 278166 | male | 11.2 | Mild | Unvaccinated | 17.23 | 3 | 12 |

| BA.2 | 278170 | female | 34 | Mild | Boosted | 13.31 | 3 | 16 |
| --- | --- | --- | --- | --- | --- | --- | --- | --- |
| BA.2 | 278179 | male | 53 | Mild | Boosted | 14.03 | 5 | 18 |
| BA.2 | 278183 | female | 5.5 | Mild | Fully Vaccinated | 20. 19 | 4 | 10 |
| BA.2 | 278196 | female | 43 | Asymptomatic | Boosted | 19. 14 | 3 | 9 |
| BA.2 | 278198 | female | 28 | Moderate | Boosted | 26.54 | 4 | 9 |
| BA.2 | 278200 | male | 46 | Asymptomatic | Boosted | 12.86 | 3 | 15 |
| BA.2 | 278201 | female | 47 | Asymptomatic | Boosted | 17.86 | 6 | 15 |
| BA.2 | 278202 | male | 11.6 | Asymptomatic | Fully Vaccinated | 16.4 | 3 | 15 |
| BA.2 | 278203 | female | 9.33 | Mild | Unvaccinated | 12.65 | 3 | 10 |
| BA.2 | 278210 | female | 43 | Mild | Boosted | 16.34 | 3 | 14 |
| BA.2 | 278233 | female | 50 | Mild | Fully Vaccinated | 18.65 | 3 | 15 |
| BA.2 | 278235 | female | 32 | Asymptomatic | Boosted | 14.32 | 4 | 15 |
| BA.2 | 278246 | male | 46 | Mild | Boosted | 24.26 | 7 | 23 |
| BA.2 | 278249 | female | 30 | Mild | Fully Vaccinated | 13.99 | 5 | 12 |
| BA.2 | 278254 | female | 49 | Mild | Boosted | 18.31 | 3 | 14 |
| BA.2 | 278255 | male | 24 | Mild | Fully Vaccinated | 13.09 | 4 | 17 |
| BA.2 | 278256 | male | 22 | Mild | Fully Vaccinated | 13.61 | 3 | 16 |
| BA.2 | 278257 | male | 45 | Mild | Fully Vaccinated | 14. 18 | 6 | 15 |
| BA.2 | 278258 | male | 48 | Mild | Fully Vaccinated | 14.48 | 3 | 16 |
| BA.2 | 278259 | male | 32 | Mild | Fully Vaccinated | 13.66 | 3 | 16 |
| BA.2 | 278260 | female | 23 | Mild | Fully Vaccinated | 17.38 | 5 | 20 |
| BA.2 | 278261 | male | 37 | Mild | Fully Vaccinated | 13.71 | 4 | 14 |
| BA.2 | 278263 | female | 27 | Mild | Fully Vaccinated | 26.07 | 7 | 12 |
| BA.2 | 278264 | female | 62 | Mild | Fully Vaccinated | 15.7 | 8 | 18 |
| BA.2 | 278266 | male | 32 | Asymptomatic | Boosted | 19.5 | 4 | 16 |
| BA.2 | 278269 | female | 40 | Mild | Boosted | 13.32 | 3 | 18 |
| BA.2 | 278270 | female | 36 | Mild | Boosted | 12 | 3 | 13 |
| BA.2 | 278280 | male | 44 | Moderate | Fully Vaccinated | 17.78 | 6 | 14 |
| BA.2 | 278281 | male | 23 | Mild | Fully Vaccinated | 14. 11 | 3 | 16 |
| BA.2 | 278283 | male | 81 | Mild | Unvaccinated | 20.23 | 9 | 18 |
| BA.2 | 278286 | male | 45 | Mild | Boosted | 16.76 | 4 | 15 |
| BA.2 | 278293 | female | 12.9 | Moderate | Unvaccinated | 17.67 | 4 | 14 |

| BA.2 | 278294 | female | 47 | Moderate | Boosted | 16.7 | 6 | 13 |
| --- | --- | --- | --- | --- | --- | --- | --- | --- |
| BA.2 | 278297 | male | 21 | Mild | Unvaccinated | 15.87 | 6 | 18 |
| BA.2 | 278298 | male | 32 | Mild | Unvaccinated | 18.28 | 3 | 15 |
| BA.2 | 278303 | male | 31 | Mild | Unvaccinated | 19.29 | 8 | 15 |
| BA.2 | 278304 | male | 23 | Mild | Unvaccinated | 16.25 | 2 | 15 |
| BA.2 | 278307 | male | 21 | Mild | Unvaccinated | 16.91 | 4 | 16 |
| BA.2 | 278308 | male | 25 | Mild | Unvaccinated | 22.29 | 5 | 11 |
| BA.2 | 278312 | male | 20 | Mild | Unvaccinated | 18.49 | 3 | 14 |
| BA.2 | 278315 | male | 25 | Mild | Unvaccinated | 19. 15 | 3 | 15 |
| BA.2 | 278317 | female | 29 | Mild | Unvaccinated | 16.67 | 3 | 16 |
| BA.2 | 278318 | male | 8.92 | Moderate | Unvaccinated | 24.37 | 3 | 14 |
| BA.2 | 278319 | male | 5.92 | Mild | Unvaccinated | 19.51 | 3 | 14 |
| BA.2 | 278320 | female | 11.9 | Mild | Fully Vaccinated | 16.4 | 2 | 10 |
| BA.2 | 278328 | female | 52 | Mild | Fully Vaccinated | 13.58 | 2 | 17 |
| BA.2 | 278333 | female | 47 | Mild | Boosted | 20.46 | 5 | 18 |
| BA.2 | 278337 | male | 47 | Mild | Boosted | 16.82 | 3 | 18 |
| BA.2 | 278339 | female | 26 | Mild | Boosted | 21.31 | 2 | 12 |
| BA.2 | 278343 | male | 9.58 | Mild | Unvaccinated | 22. 15 | 2 | 13 |
| BA.2 | 278347 | male | 79 | Mild | Fully Vaccinated | 18.22 | 4 | 19 |
| BA.2 | 278349 | female | 41 | Mild | Fully Vaccinated | 13.65 | 3 | 13 |
| BA.2 | 278352 | female | 37 | Moderate | Boosted | 13.26 | 2 | 15 |
| BA.2 | 278353 | male | 10.5 | Moderate | Boosted | 21.62 | 1 | 10 |
| BA.2 | 278360 | female | 51 | Mild | Boosted | 20.38 | 6 | 13 |
| BA.2 | 278361 | female | 57 | Mild | Boosted | 14.69 | 2 | 16 |
| BA.2 | 278363 | female | 47 | Mild | Boosted | 16.25 | 2 | 14 |
| BA.2 | 278367 | female | 48 | Moderate | Boosted | 15 | 4 | 18 |
| BA.2 | 278368 | male | 13 | Mild | Fully Vaccinated | 15. 16 | 4 | 13 |
| BA.2 | 278369 | male | 11 | Mild | Fully Vaccinated | 24.27 | 7 | 12 |
| BA.2 | 278371 | male | 49 | Mild | Boosted | 19.55 | 4 | 10 |
| BA.2 | 278372 | female | 14.7 | Mild | Fully Vaccinated | 14.62 | 4 | 10 |
| BA.2 | 278373 | female | 29 | Mild | Fully Vaccinated | 12.85 | 13 | 18 |
| BA.2 | 278375 | female | 1 | Mild | Unvaccinated | 25. 16 | 3 | 16 |

| BA.2 | 278378 | male | 10.7 | Mild | Unvaccinated | 18.78 | 2 | 18 |
| --- | --- | --- | --- | --- | --- | --- | --- | --- |
| BA.2 | 278381 | female | 11.9 | Mild | Unvaccinated | 22.36 | 6 | 13 |
| BA.2 | 278393 | male | 2.99 | Mild | Unvaccinated | 21 | 3 | 12 |
| BA.2 | 278394 | female | 46 | Mild | Boosted | 14.41 | 3 | 15 |
| BA.2 | 278396 | female | 57 | Moderate | Boosted | 15.78 | 3 | 14 |
| BA.2 | 278397 | female | 32 | Mild | Fully Vaccinated | 14.87 | 3 | 16 |
| BA.2 | 278399 | female | 48 | Mild | Fully Vaccinated | 13.22 | 3 | 18 |
| BA.2 | 278400 | female | 42 | Mild | Boosted | 21.02 | 6 | 15 |
| BA.2 | 278425 | male | 17 | Mild | Fully Vaccinated | 16.08 | 6 | 13 |
| BA.2 | 278426 | male | 15 | Mild | Fully Vaccinated | 18.84 | 3 | 15 |
| BA.2 | 278432 | female | 51 | Mild | Fully Vaccinated | 16. 18 | 3 | 18 |
| BA.2 | 278434 | female | 10.9 | Mild | Unvaccinated | 17.54 | 2 | 15 |
| BA.2 | 278437 | male | 53 | Mild | Boosted | 15.52 | 3 | 17 |
| BA.2 | 278438 | female | 53 | Moderate | Fully Vaccinated | 16.27 | 3 | 19 |
| BA.2 | 278440 | male | 25 | Moderate | Fully Vaccinated | 16. 13 | 3 | 13 |
| BA.2 | 278441 | male | 58 | Mild | Unvaccinated | 19.08 | 3 | 18 |
| BA.2 | 278442 | female | 12.5 | Mild | Fully Vaccinated | 19. 16 | 3 | 10 |
| BA.2 | 278443 | male | 7.75 | Mild | Fully Vaccinated | 26.92 | 4 | 14 |
| BA.2 | 278444 | female | 10 | Mild | Unvaccinated | 16.05 | 3 | 19 |
| BA.2 | 278445 | male | 9.92 | Mild | Unvaccinated | 16.81 | 3 | 12 |
| BA.2 | 278446 | male | 49 | Mild | Boosted | 19.3 | 6 | 20 |
| BA.2 | 278454 | male | 11.9 | Mild | Unvaccinated | 19. 1 | 2 | 12 |
| BA.2 | 278455 | female | 15 | Mild | Fully Vaccinated | 17. 15 | 3 | 13 |
| BA.2 | 278456 | female | 16 | Mild | Fully Vaccinated | 16. 16 | 6 | 20 |
| BA.2 | 278457 | male | 46 | Mild | Boosted | 16.03 | 3 | 12 |
| BA.2 | 278458 | male | 52 | Mild | Boosted | 17.94 | 3 | 15 |
| BA.2 | 278459 | male | 64 | Mild | Fully Vaccinated | 16.75 | 3 | 13 |
| BA.2 | 278461 | male | 11 | Mild | Unvaccinated | 20.03 | 3 | 16 |
| BA.2 | 278462 | female | 21 | Mild | Boosted | 21.65 | 5 | 13 |
| BA.2 | 278463 | female | 45 | Mild | Boosted | 15. 11 | 2 | 12 |
| BA.2 | 278464 | male | 56 | Mild | Boosted | 15.03 | 2 | 10 |
| BA.2 | 278465 | female | 42 | Mild | Unvaccinated | 19.95 | 3 | 12 |

| BA.2 | 278467 | female | 33 | Mild | Boosted | 21.84 | 10 | 13 |
| --- | --- | --- | --- | --- | --- | --- | --- | --- |
| BA.2 | 278468 | female | 47 | Mild | Boosted | 13.76 | 3 | 31 |
| BA.2 | 278475 | male | 5.5 | Mild | Unvaccinated | 23.25 | 7 | 16 |
| BA.2 | 278478 | female | 4.08 | Asymptomatic | Fully Vaccinated | 16. 14 | 3 | 12 |
| BA.2 | 278482 | female | 7.83 | Mild | Unvaccinated | 15.55 | 3 | 10 |
| BA.2 | 278489 | male | 40 | Mild | Boosted | 15.44 | 2 | 12 |
| BA.2 | 278506 | female | 5.25 | Mild | Unvaccinated | 16.81 | 3 | 11 |
| BA.2 | 278514 | female | 13.8 | Mild | Fully Vaccinated | 16.9 | 6 | 18 |
| BA.2 | 278523 | female | 12.5 | Mild | Fully Vaccinated | 15. 15 | 3 | 11 |
| BA.2 | 280497 | female | 50 | Asymptomatic | Unvaccinated | 18.06 | 3 | 13 |
| BA.2 | 280502 | female | 24 | Asymptomatic | Boosted | 16. 1 | 4 | 17 |
| BA.2 | 280512 | male | 36 | Asymptomatic | Fully Vaccinated | 18.95 | 3 | 8 |
| BA.2 | 280561 | male | 58 | Asymptomatic | Boosted | 14.27 | 5 | 11 |
| BA.2 | 280678 | female | 63 | Asymptomatic | Boosted | 18.25 | 7 | 10 |
| BA.2 | 280694 | male | 34 | Asymptomatic | Unvaccinated | 17.82 | 5 | 13 |
| BA.2 | 280710 | female | 62 | Asymptomatic | Unvaccinated | 28.36 | 2 | 5 |
| BA.2 | 280755 | male | 48 | Asymptomatic | Boosted | 17.77 | 3 | 11 |
| BA.2 | 280795 | male | 34 | Moderate | Boosted | 23. 1 | 4 | 12 |
| BA.2 | 280800 | male | 59 | Asymptomatic | Boosted | 23. 14 | 8 | 14 |
| BA.2 | 280809 | male | 33 | Mild | Fully Vaccinated | 19. 1 | 3 | 8 |
| BA.2 | 280825 | female | 31 | Mild | Unvaccinated | 12.71 | 4 | 12 |
| BA.2 | 280835 | female | 12. 1 | Asymptomatic | Fully Vaccinated | 20.39 | 2 | 9 |
| BA.2 | 280836 | female | 39 | Asymptomatic | Boosted | 14.5 | 2 | 17 |
| BA.2 | 280850 | male | 24 | Mild | Boosted | 14.78 | 3 | 12 |
| BA.2 | 280851 | female | 42 | Asymptomatic | Boosted | 21.96 | 3 | 11 |
| BA.2 | 280935 | female | 28 | Asymptomatic | Unvaccinated | 24 | 7 | 12 |
| BA.2 | 280961 | male | 24 | Asymptomatic | Boosted | 16. 14 | 4 | 13 |
| BA.2 | 281022 | male | 27 | Mild | Boosted | 18.7 | 8 | 13 |
| BA.2 | 281023 | male | 21 | Asymptomatic | Boosted | 17.78 | 6 | 10 |
| BA.2 | 281024 | male | 27 | Mild | Boosted | 20.71 | 6 | 14 |
| BA.2 | 281025 | male | 38 | Asymptomatic | Boosted | 15.85 | 6 | 12 |
| BA.2 | 281028 | male | 34 | Asymptomatic | Boosted | 14.71 | 3 | 10 |

| BA.2 | 281034 | male | 25 | Mild | Boosted | 13.67 | 3 | 10 |
| --- | --- | --- | --- | --- | --- | --- | --- | --- |
| BA.2 | 281035 | male | 45 | Asymptomatic | Boosted | 15.06 | 5 | 14 |
| BA.2 | 281048 | female | 27 | Mild | Unvaccinated | 16.88 | 3 | 13 |
| BA.2 | 281063 | male | 47 | Asymptomatic | Fully Vaccinated | 18.84 | 6 | 10 |
| BA.2 | 281064 | male | 59 | Mild | Boosted | 16.36 | 9 | 15 |
| BA.2 | 281079 | male | 30 | Asymptomatic | Boosted | 18.91 | 4 | 8 |
| BA.2 | 281085 | male | 26 | Mild | Boosted | 20.26 | 10 | 17 |
| BA.2 | 281095 | male | 28 | Asymptomatic | Boosted | 19.36 | 8 | 14 |
| BA.2 | 281096 | male | 36 | Asymptomatic | Fully Vaccinated | 17.79 | 9 | 14 |
| BA.2 | 281100 | female | 57 | Mild | Boosted | 15.76 | 4 | 14 |
| BA.2 | 281101 | male | 38 | Mild | Boosted | 22.75 | 3 | 10 |
| BA.2 | 281103 | male | 33 | Mild | Boosted | 18.46 | 3 | 10 |
| BA.2 | 281104 | male | 33 | Asymptomatic | Boosted | 14.32 | 2 | 9 |
| BA.2 | 281105 | male | 31 | Asymptomatic | Fully Vaccinated | 15.2 | 5 | 13 |
| BA.2 | 281106 | male | 33 | Mild | Boosted | 18.56 | 3 | 10 |
| BA.2 | 281108 | male | 25 | Asymptomatic | Boosted | 18.5 | 5 | 10 |
| BA.2 | 281109 | male | 38 | Asymptomatic | Fully Vaccinated | 17.88 | 5 | 10 |
| BA.2 | 281110 | male | 29 | Moderate | Boosted | 12. 17 | 3 | 12 |
| BA.2 | 281111 | male | 32 | Asymptomatic | Boosted | 19.04 | 5 | 12 |
| BA.2 | 281113 | male | 34 | Asymptomatic | Boosted | 17.73 | 5 | 10 |
| BA.2 | 281114 | male | 47 | Asymptomatic | Boosted | 15.48 | 7 | 12 |
| BA.2 | 281117 | male | 56 | Asymptomatic | Boosted | 17.09 | 5 | 18 |
| BA.2 | 281119 | male | 37 | Asymptomatic | Boosted | 20. 18 | 8 | 16 |
| BA.2 | 281128 | male | 23 | Mild | Boosted | 16.08 | 4 | 8 |
| BA.2 | 281129 | male | 47 | Asymptomatic | Boosted | 16.94 | 6 | 9 |
| BA.2 | 281142 | male | 42 | Asymptomatic | Boosted | 16. 12 | 4 | 9 |
| BA.2 | 281144 | male | 26 | Asymptomatic | Fully Vaccinated | 18.82 | 4 | 12 |
| BA.2 | 281146 | male | 32 | Asymptomatic | Fully Vaccinated | 17.87 | 5 | 11 |
| BA.2 | 281186 | male | 22 | Asymptomatic | Boosted | 21.75 | 4 | 11 |
| BA.2 | 281187 | male | 24 | Asymptomatic | Fully Vaccinated | 17.47 | 2 | 6 |
| BA.2 | 281188 | male | 27 | Moderate | Boosted | 20. 11 | 4 | 9 |
| BA.2 | 281189 | male | 22 | Asymptomatic | Boosted | 18.89 | 4 | 12 |

| BA.2 | 281205 | male | 43 | Moderate | Boosted | 15.69 | 5 | 8 |
| --- | --- | --- | --- | --- | --- | --- | --- | --- |
| BA 2 | 281249 | male | 49 | Mild | Boosted | 17 41 | 4 | 13 |
| BA 2 | 281250 | male | 34 | Asymptomatic | Boosted | 15 13 | 3 | 8 |
| BA.2 | 281251 | male | 35 | Mild | Boosted | 22.27 | 7 | 9 |
| BA 2 | 281252 | male | 32 | Asymptomatic | Boosted | 18 49 | 3 | 16 |
| BA 2 | 281257 | male | 35 | Moderate | Boosted | 17 96 | 3 | 18 |
| BA.2 | 281258 | male | 27 | Mild | Boosted | 14. 13 | 5 | 11 |
| BA.2 | 281280 | male | 49 | Mild | Boosted | 20.52 | 5 | 11 |
| BA 2 | 281299 | male | 38 | Mild | Boosted | 12 38 | 3 | 14 |
| BA.2 | 281300 | male | 21 | Mild | Fully Vaccinated | 14.03 | 3 | 9 |
| BA.2 | 281312 | male | 38 | Asymptomatic | Boosted | 15.95 | 3 | 9 |
| BA 2 | 281313 | male | 32 | Asymptomatic | Fully Vaccinated | 14 86 | 3 | 15 |
| BA 2 | 281314 | male | 40 | Mild | Fully Vaccinated | 22 46 | 3 | 9 |
| BA.2 | 281371 | male | 26 | Mild | Boosted | 18.5 | 2 | 9 |
| BA 2 | 281469 | male | 33 | Moderate | Fully Vaccinated | 12 11 | 3 | 13 |
| BA 2 | 281737 | female | 25 | Mild | Boosted | 14 27 | 3 | 12 |
| BA.2 | 281772 | female | 35 | Mild | Boosted | 15.9 | 4 | 17 |
| BA.2 | 282057 | male | 26 | Mild | Unvaccinated | 13.97 | 3 | 11 |

| **variants** | **ID** | **Gender** | **Age** | **Severity** | **Vaccination** | **Peak** **Ct** **(N** **gene)** | **Days** **for** **peak** **viral** **load** | **Days** **for** **viral** **clearance** |
| --- | --- | --- | --- | --- | --- | --- | --- | --- |
| BA.5 | 295729 | female | 3 | Mild | Unvaccinated | 29.95 | 4 | 6 |
| BA.5 | 295109 | female | 10 | Mild | Fully Vaccinated | 28.48 | 6 | 7 |
| BA.5 | 294841 | male | 1 | Asymptomatic | Unvaccinated | 28.2 | 5 | 7 |
| BA.5 | 295482 | male | 34 | Mild | Fully Vaccinated | 27.83 | 2 | 13 |
| BA.5 | 295603 | male | 11 | Asymptomatic | Boosted | 27.78 | 4 | 7 |
| BA.5 | 295702 | female | 3 | Mild | Unvaccinated | 27.67 | 6 | 9 |
| BA.5 | 294846 | male | 27 | Mild | Boosted | 27.6 | 4 | 7 |
| BA.5 | 291398 | male | 27 | Mild | Fully Vaccinated | 27.01 | 4 | 6 |
| BA.5 | 294942 | female | 1 | Mild | Unknown | 26.81 | 3 | 6 |
| BA.5 | 295508 | female | 3 | Mild | Unknown | 26.23 | 3 | 8 |
| BA.5 | 294970 | male | 54 | Asymptomatic | Boosted | 25.9 | 2 | 12 |
| BA.5 | 295397 | male | 3 | Mild | Unvaccinated | 25.56 | 10 | 8 |
| BA.5 | 294842 | male | 5 | Mild | Fully Vaccinated | 25.51 | 4 | 9 |
| BA.5 | 295588 | female | 5 | Asymptomatic | Fully Vaccinated | 25.41 | 2 | 5 |
| BA.5 | 295487 | female | 4 | Mild | Unvaccinated | 24.87 | 6 | 8 |
| BA.5 | 294579 | male | 10 | Asymptomatic | Fully Vaccinated | 24.42 | 2 | 10 |
| BA.5 | 294895 | female | 1 | Mild | Unvaccinated | 24.23 | 4 | 10 |
| BA.5 | 294540 | male | 12 | Asymptomatic | Fully Vaccinated | 24.09 | 2 | 11 |
| BA.5 | 294299 | female | 36 | Mild | Boosted | 24.07 | 5 | 11 |
| BA.5 | 295372 | female | 8 | Asymptomatic | Unknown | 23.88 | 3 | 9 |
| BA.5 | 294474 | male | 34 | Mild | Boosted | 23.73 | 5 | 8 |
| BA.5 | 295248 | female | 11 | Asymptomatic | Fully Vaccinated | 23.73 | 11 | 16 |
| BA.5 | 295589 | female | 8 | Asymptomatic | Fully Vaccinated | 23.69 | 2 | 12 |
| BA.5 | 295739 | male | 1 | Asymptomatic | Unvaccinated | 23.49 | 2 | 15 |
| BA.5 | 294886 | female | 19 | Mild | Boosted | 23.41 | 5 | 15 |
| BA.5 | 294922 | female | 47 | Mild | Boosted | 23.38 | 2 | 13 |
| BA.5 | 294664 | female | 30 | Mild | Boosted | 23.31 | 2 | 8 |
| BA.5 | 295371 | male | 35 | Mild | Unknown | 23.3 | 5 | 10 |
| BA.5 | 295036 | female | 40 | Mild | Boosted | 22.94 | 2 | 10 |
| BA.5 | 290452 | male | 28 | Mild | Boosted | 22.89 | 4 | 7 |
| BA.5 | 294880 | male | 55 | Asymptomatic | Boosted | 22.88 | 6 | 13 |

| BA.5 | 295268 | male | 56 | Asymptomatic | Unknown | 22.82 | 8 | 16 |
| --- | --- | --- | --- | --- | --- | --- | --- | --- |
| BA.5 | 295267 | male | 28 | Mild | Boosted | 22.76 | 3 | 9 |
| BA.5 | 294563 | male | 57 | Asymptomatic | Unknown | 22.72 | 5 | 12 |
| BA.5 | 291289 | male | 49 | Asymptomatic | Boosted | 22.5 | 7 | 11 |
| BA.5 | 295214 | female | 36 | Mild | Boosted | 22.45 | 6 | 10 |
| BA.5 | 287655 | male | 28 | Mild | Boosted | 22.42 | 4 | 8 |
| BA.5 | 295736 | female | 43 | Moderate | Boosted | 22.41 | 7 | 12 |
| BA.5 | 289545 | male | 53 | Mild | Boosted | 22.26 | 4 | 7 |
| BA.5 | 295216 | female | 29 | Moderate | Boosted | 22. 14 | 3 | 12 |
| BA.5 | 294935 | female | 54 | Asymptomatic | Boosted | 22. 12 | 6 | 9 |
| BA.5 | 295570 | female | 8 | Asymptomatic | Fully Vaccinated | 22. 12 | 3 | 11 |
| BA.5 | 295266 | male | 29 | Mild | Boosted | 22.05 | 6 | 11 |
| BA.5 | 292066 | female | 38 | Mild | Boosted | 22.03 | 3 | 15 |
| BA.5 | 294954 | male | 42 | Mild | Boosted | 22.03 | 2 | 10 |
| BA.5 | 290449 | male | 21 | Mild | Boosted | 21.98 | 3 | 5 |
| BA.5 | 294334 | male | 13 | Mild | Fully Vaccinated | 21.87 | 3 | 12 |
| BA.5 | 294578 | female | 37 | Mild | Boosted | 21.69 | 1 | 10 |
| BA.5 | 295735 | female | 60 | Asymptomatic | Boosted | 21.59 | 7 | 15 |
| BA.5 | 294926 | female | 5 | Asymptomatic | Fully Vaccinated | 21.55 | 4 | 11 |
| BA.5 | 295362 | female | 49 | Mild | Boosted | 21.43 | 5 | 12 |
| BA.5 | 295622 | male | 50 | Asymptomatic | Boosted | 21.36 | 5 | 13 |
| BA.5 | 294621 | male | 34 | Asymptomatic | Unvaccinated | 21.34 | 3 | 7 |
| BA.5 | 289771 | female | 38 | Mild | Boosted | 21.31 | 8 | 13 |
| BA.5 | 294420 | male | 19 | Asymptomatic | Boosted | 21.29 | 3 | 7 |
| BA.5 | 294892 | female | 83 | Mild | Unvaccinated | 21.27 | 5 | 16 |
| BA.5 | 294167 | male | 27 | Mild | Boosted | 21.22 | 5 | 11 |
| BA.5 | 293920 | male | 46 | Mild | Boosted | 21. 14 | 5 | 11 |
| BA.5 | 295710 | female | 6 | Mild | Fully Vaccinated | 21.07 | 3 | 6 |
| BA.5 | 294605 | male | 12 | Mild | Unknown | 21.05 | 3 | 15 |
| BA.5 | 294948 | female | 31 | Mild | Fully Vaccinated | 21.04 | 9 | 12 |
| BA.5 | 295790 | female | 8 | Asymptomatic | Fully Vaccinated | 21.04 | 10 | 15 |
| BA.5 | 295797 | male | 41 | Mild | Boosted | 20.98 | 5 | 11 |

| BA.5 | 294115 | male | 49 | Mild | Boosted | 20.9 | 5 | 10 |
| --- | --- | --- | --- | --- | --- | --- | --- | --- |
| BA.5 | 295223 | male | 28 | Moderate | Fully Vaccinated | 20.84 | 6 | 11 |
| BA.5 | 295782 | male | 8 | Mild | Unvaccinated | 20.78 | 2 | 3 |
| BA.5 | 295655 | male | 18 | Mild | Fully Vaccinated | 20.74 | 5 | 15 |
| BA.5 | 295743 | female | 37 | Mild | Boosted | 20.74 | 2 | 14 |
| BA.5 | 295590 | male | 72 | Mild | Unvaccinated | 20.73 | 5 | 12 |
| BA.5 | 294907 | male | 39 | Asymptomatic | Fully Vaccinated | 20.69 | 3 | 16 |
| BA.5 | 294447 | male | 18 | Mild | Boosted | 20.67 | 4 | 10 |
| BA.5 | 295367 | male | 38 | Asymptomatic | Boosted | 20.56 | 12 | 18 |
| BA.5 | 295612 | female | 5 | Mild | Fully Vaccinated | 20.46 | 3 | 6 |
| BA.5 | 295125 | male | 31 | Asymptomatic | Boosted | 20.42 | 5 | 10 |
| BA.5 | 294876 | male | 23 | Mild | Boosted | 20.36 | 2 | 12 |
| BA.5 | 295030 | male | 55 | Asymptomatic | Unvaccinated | 20.36 | 9 | 13 |
| BA.5 | 295488 | male | 10 | Mild | Fully Vaccinated | 20.29 | 3 | 6 |
| BA.5 | 294619 | male | 35 | Asymptomatic | Fully Vaccinated | 20.28 | 3 | 11 |
| BA.5 | 292069 | female | 49 | Mild | Unvaccinated | 20.25 | 6 | 10 |
| BA.5 | 294849 | female | 25 | Mild | Unknown | 20.24 | 3 | 13 |
| BA.5 | 295205 | male | 38 | Mild | Boosted | 20. 17 | 3 | 11 |
| BA.5 | 294901 | male | 34 | Asymptomatic | Boosted | 20. 14 | 5 | 14 |
| BA.5 | 294845 | female | 29 | Asymptomatic | Boosted | 20. 1 | 7 | 11 |
| BA.5 | 295051 | female | 30 | Mild | Boosted | 20.09 | 8 | 11 |
| BA.5 | 295511 | female | 53 | Mild | Boosted | 20.09 | 3 | 12 |
| BA.5 | 295561 | female | 26 | Asymptomatic | Fully Vaccinated | 20.09 | 5 | 12 |
| BA.5 | 295818 | female | 5 | Mild | Fully Vaccinated | 20 | 2 | 9 |
| BA.5 | 295260 | male | 73 | Moderate | Unvaccinated | 19.98 | 2 | 11 |
| BA.5 | 294847 | male | 44 | Mild | Boosted | 19.89 | 4 | 11 |
| BA.5 | 295514 | male | 29 | Moderate | Boosted | 19.88 | 3 | 12 |
| BA.5 | 290447 | male | 22 | Mild | Boosted | 19.86 | 3 | 8 |
| BA.5 | 294305 | male | 43 | Mild | Boosted | 19.85 | 4 | 10 |
| BA.5 | 295245 | female | 67 | Asymptomatic | Unknown | 19.83 | 8 | 14 |
| BA.5 | 294758 | male | 35 | Mild | Unknown | 19.81 | 4 | 10 |
| BA.5 | 294582 | male | 22 | Asymptomatic | Boosted | 19.8 | 5 | 12 |

| BA.5 | 295092 | female | 18 | Mild | Unknown | 19.79 | 4 | 10 |
| --- | --- | --- | --- | --- | --- | --- | --- | --- |
| BA.5 | 295446 | male | 19 | Mild | Boosted | 19.68 | 5 | 12 |
| BA.5 | 291867 | male | 31 | Mild | Boosted | 19.67 | 2 | 10 |
| BA.5 | 293961 | male | 9 | Mild | Fully Vaccinated | 19.67 | 3 | 11 |
| BA.5 | 294566 | female | 46 | Mild | Boosted | 19.65 | 7 | 12 |
| BA.5 | 294622 | male | 51 | Asymptomatic | Boosted | 19.51 | 6 | 11 |
| BA.5 | 295395 | male | 25 | Mild | Boosted | 19.5 | 4 | 9 |
| BA.5 | 295261 | female | 1 | Mild | Unknown | 19.49 | 3 | 10 |
| BA.5 | 294297 | female | 1 | Mild | Unvaccinated | 19.48 | 3 | 8 |
| BA.5 | 295479 | female | 53 | Mild | Boosted | 19.48 | 5 | 11 |
| BA.5 | 295286 | female | 45 | Asymptomatic | Boosted | 19.46 | 3 | 9 |
| BA.5 | 294421 | male | 51 | Mild | Boosted | 19.44 | 6 | 12 |
| BA.5 | 295329 | female | 40 | Asymptomatic | Unvaccinated | 19.44 | 2 | 13 |
| BA.5 | 294322 | male | 36 | Mild | Boosted | 19.41 | 5 | 8 |
| BA.5 | 295445 | male | 25 | Mild | Unknown | 19.39 | 3 | 13 |
| BA.5 | 295584 | female | 41 | Asymptomatic | Boosted | 19.38 | 6 | 12 |
| BA.5 | 289949 | male | 58 | Moderate | Boosted | 19.36 | 6 | 14 |
| BA.5 | 294303 | male | 14 | Mild | Fully Vaccinated | 19.36 | 6 | 10 |
| BA.5 | 295052 | male | 51 | Mild | Boosted | 19.35 | 6 | 11 |
| BA.5 | 294527 | male | 50 | Asymptomatic | Boosted | 19.32 | 5 | 16 |
| BA.5 | 295730 | male | 35 | Asymptomatic | Boosted | 19.32 | 8 | 13 |
| BA.5 | 294967 | female | 41 | Asymptomatic | Unknown | 19.29 | 1 | 7 |
| BA.5 | 295236 | male | 30 | Mild | Unknown | 19.23 | 3 | 11 |
| BA.5 | 294398 | female | 41 | Mild | Boosted | 19.21 | 2 | 12 |
| BA.5 | 295071 | female | 45 | Mild | Fully Vaccinated | 19.2 | 2 | 9 |
| BA.5 | 293988 | female | 30 | Mild | Boosted | 19. 19 | 3 | 11 |
| BA.5 | 294641 | male | 27 | Mild | Boosted | 19. 18 | 4 | 8 |
| BA.5 | 294921 | female | 25 | Mild | Fully Vaccinated | 19. 16 | 4 | 16 |
| BA.5 | 295106 | female | 46 | Mild | Boosted | 19. 15 | 4 | 12 |
| BA.5 | 294497 | male | 33 | Asymptomatic | Boosted | 19. 12 | 2 | 8 |
| BA.5 | 294919 | female | 32 | Mild | Boosted | 19. 1 | 2 | 10 |
| BA.5 | 295677 | male | 20 | Asymptomatic | Fully Vaccinated | 19.09 | 2 | 14 |

| BA.5 | 293996 | male | 34 | Asymptomatic | Boosted | 19.08 | 7 | 15 |
| --- | --- | --- | --- | --- | --- | --- | --- | --- |
| BA.5 | 295599 | male | 54 | Mild | Boosted | 19.08 | 8 | 13 |
| BA.5 | 294589 | female | 47 | Moderate | Unknown | 19.07 | 3 | 15 |
| BA.5 | 294743 | female | 47 | Asymptomatic | Unknown | 19.07 | 2 | 13 |
| BA.5 | 294958 | female | 7 | Mild | Fully Vaccinated | 19.07 | 6 | 11 |
| BA.5 | 295770 | male | 49 | Asymptomatic | Boosted | 19.04 | 3 | 13 |
| BA.5 | 295207 | female | 39 | Asymptomatic | Boosted | 19.02 | 7 | 13 |
| BA.5 | 295270 | male | 51 | Mild | Unknown | 19.01 | 6 | 14 |
| BA.5 | 295786 | female | 56 | Asymptomatic | Fully Vaccinated | 19.01 | 2 | 11 |
| BA.5 | 295115 | female | 9 | Mild | Fully Vaccinated | 18.98 | 2 | 12 |
| BA.5 | 295262 | male | 50 | Asymptomatic | Boosted | 18.98 | 6 | 12 |
| BA.5 | 295778 | female | 32 | Mild | Unvaccinated | 18.98 | 3 | 11 |
| BA.5 | 295512 | female | 24 | Asymptomatic | Boosted | 18.95 | 3 | 13 |
| BA.5 | 295595 | male | 57 | Asymptomatic | Unknown | 18.93 | 3 | 11 |
| BA.5 | 295619 | male | 28 | Mild | Fully Vaccinated | 18.92 | 2 | 11 |
| BA.5 | 294929 | male | 44 | Mild | Boosted | 18.87 | 6 | 14 |
| BA.5 | 295144 | female | 22 | Mild | Boosted | 18.87 | 3 | 9 |
| BA.5 | 292064 | male | 24 | Mild | Fully Vaccinated | 18.86 | 4 | 8 |
| BA.5 | 294908 | male | 33 | Asymptomatic | Boosted | 18.86 | 5 | 17 |
| BA.5 | 294653 | male | 51 | Mild | Unknown | 18.85 | 4 | 13 |
| BA.5 | 295501 | male | 36 | Mild | Boosted | 18.85 | 4 | 13 |
| BA.5 | 295717 | male | 33 | Mild | Boosted | 18.85 | 4 | 10 |
| BA.5 | 292184 | male | 30 | Mild | Fully Vaccinated | 18.84 | 2 | 7 |
| BA.5 | 295252 | male | 30 | Mild | Boosted | 18.83 | 3 | 10 |
| BA.5 | 291207 | male | 61 | Mild | Boosted | 18.82 | 5 | 10 |
| BA.5 | 295102 | male | 54 | Asymptomatic | Unknown | 18.82 | 6 | 13 |
| BA.5 | 294890 | male | 37 | Mild | Boosted | 18.81 | 2 | 8 |
| BA.5 | 295333 | female | 22 | Mild | Fully Vaccinated | 18.81 | 3 | 10 |
| BA.5 | 294968 | male | 34 | Mild | Boosted | 18.77 | 3 | 10 |
| BA.5 | 295202 | male | 48 | Asymptomatic | Fully Vaccinated | 18.77 | 7 | 14 |
| BA.5 | 295386 | female | 33 | Asymptomatic | Boosted | 18.76 | 3 | 10 |
| BA.5 | 294392 | male | 40 | Mild | Fully Vaccinated | 18.75 | 5 | 14 |

| BA.5 | 294703 | female | 46 | Mild | Boosted | 18.74 | 4 | 11 |
| --- | --- | --- | --- | --- | --- | --- | --- | --- |
| BA.5 | 294728 | female | 40 | Asymptomatic | Boosted | 18.7 | 3 | 17 |
| BA.5 | 291285 | female | 50 | Mild | Fully Vaccinated | 18.68 | 3 | 9 |
| BA.5 | 294965 | female | 4 | Asymptomatic | Fully Vaccinated | 18.68 | 3 | 9 |
| BA.5 | 294796 | male | 41 | Mild | Boosted | 18.63 | 4 | 9 |
| BA.5 | 294137 | male | 20 | Mild | Fully Vaccinated | 18.57 | 3 | 11 |
| BA.5 | 295278 | male | 30 | Asymptomatic | Boosted | 18.51 | 3 | 11 |
| BA.5 | 295095 | male | 31 | Asymptomatic | Boosted | 18.49 | 5 | 11 |
| BA.5 | 288461 | male | 21 | Asymptomatic | Fully Vaccinated | 18.46 | 2 | 14 |
| BA.5 | 294393 | male | 35 | Mild | Boosted | 18.46 | 2 | 14 |
| BA.5 | 294767 | male | 60 | Asymptomatic | Boosted | 18.45 | 6 | 17 |
| BA.5 | 294445 | male | 3 | Mild | Unvaccinated | 18.4 | 2 | 7 |
| BA.5 | 294727 | male | 37 | Mild | Boosted | 18.39 | 2 | 8 |
| BA.5 | 294964 | female | 1 | Mild | Unvaccinated | 18.39 | 6 | 9 |
| BA.5 | 295117 | male | 21 | Mild | Boosted | 18.39 | 5 | 12 |
| BA.5 | 294577 | female | 30 | Mild | Boosted | 18.38 | 2 | 11 |
| BA.5 | 295676 | female | 69 | Asymptomatic | Fully Vaccinated | 18.38 | 3 | 18 |
| BA.5 | 295199 | female | 13 | Mild | Fully Vaccinated | 18.37 | 4 | 15 |
| BA.5 | 295746 | female | 38 | Asymptomatic | Unvaccinated | 18.37 | 3 | 10 |
| BA.5 | 291446 | male | 25 | Mild | Boosted | 18.36 | 5 | 8 |
| BA.5 | 295174 | female | 4 | Mild | Fully Vaccinated | 18.36 | 6 | 8 |
| BA.5 | 295210 | male | 34 | Mild | Boosted | 18.34 | 3 | 10 |
| BA.5 | 294390 | male | 30 | Mild | Boosted | 18.33 | 3 | 10 |
| BA.5 | 295041 | female | 24 | Mild | Boosted | 18.33 | 3 | 14 |
| BA.5 | 295708 | female | 27 | Mild | Boosted | 18.32 | 3 | 9 |
| BA.5 | 295122 | male | 36 | Mild | Boosted | 18.31 | 6 | 13 |
| BA.5 | 295583 | male | 52 | Asymptomatic | Boosted | 18. 19 | 5 | 12 |
| BA.5 | 294424 | female | 57 | Mild | Boosted | 18. 17 | 3 | 15 |
| BA.5 | 295277 | male | 28 | Mild | Boosted | 18. 17 | 6 | 10 |
| BA.5 | 295080 | female | 21 | Moderate | Boosted | 18. 16 | 4 | 11 |
| BA.5 | 295133 | male | 26 | Mild | Boosted | 18. 16 | 3 | 8 |
| BA.5 | 294575 | female | 50 | Mild | Boosted | 18. 15 | 5 | 10 |

| BA.5 | 295056 | female | 3 | Mild | Unvaccinated | 18. 14 | 9 | 12 |
| --- | --- | --- | --- | --- | --- | --- | --- | --- |
| BA.5 | 294825 | male | 31 | Mild | Unvaccinated | 18. 11 | 6 | 10 |
| BA.5 | 295055 | male | 43 | Asymptomatic | Boosted | 18. 1 | 2 | 10 |
| BA.5 | 295124 | female | 26 | Mild | Unknown | 18. 1 | 3 | 12 |
| BA.5 | 295201 | male | 33 | Moderate | Boosted | 18.09 | 3 | 9 |
| BA.5 | 295572 | female | 26 | Mild | Unknown | 18.08 | 2 | 10 |
| BA.5 | 295749 | female | 37 | Asymptomatic | Unvaccinated | 18.08 | 3 | 13 |
| BA.5 | 294397 | male | 40 | Asymptomatic | Unvaccinated | 18.06 | 6 | 16 |
| BA.5 | 294425 | female | 15 | Mild | Fully Vaccinated | 18.04 | 3 | 11 |
| BA.5 | 295160 | male | 64 | Asymptomatic | Unvaccinated | 18.03 | 3 | 10 |
| BA.5 | 295596 | male | 31 | Mild | Boosted | 18.03 | 4 | 12 |
| BA.5 | 295376 | male | 26 | Mild | Boosted | 18.02 | 4 | 9 |
| BA.5 | 295681 | female | 16 | Asymptomatic | Unvaccinated | 18.02 | 4 | 15 |
| BA.5 | 294852 | male | 25 | Mild | Fully Vaccinated | 18.01 | 5 | 11 |
| BA.5 | 294900 | male | 40 | Asymptomatic | Fully Vaccinated | 18 | 2 | 11 |
| BA.5 | 295398 | female | 39 | Mild | Unknown | 18 | 1 | 11 |
| BA.5 | 295725 | male | 34 | Mild | Boosted | 18 | 2 | 7 |
| BA.5 | 293780 | female | 25 | Asymptomatic | Boosted | 17.99 | 5 | 13 |
| BA.5 | 294005 | female | 35 | Asymptomatic | Fully Vaccinated | 17.98 | 2 | 19 |
| BA.5 | 294884 | female | 53 | Moderate | Boosted | 17.96 | 5 | 12 |
| BA.5 | 294186 | male | 31 | Mild | Boosted | 17.95 | 4 | 12 |
| BA.5 | 293795 | male | 26 | Mild | Boosted | 17.93 | 4 | 12 |
| BA.5 | 295126 | male | 5 | Mild | Fully Vaccinated | 17.93 | 3 | 11 |
| BA.5 | 295636 | male | 60 | Asymptomatic | Boosted | 17.93 | 4 | 13 |
| BA.5 | 295392 | male | 57 | Asymptomatic | Boosted | 17.89 | 3 | 13 |
| BA.5 | 293703 | male | 28 | Mild | Boosted | 17.87 | 6 | 12 |
| BA.5 | 295403 | female | 35 | Asymptomatic | Boosted | 17.87 | 3 | 12 |
| BA.5 | 294896 | male | 41 | Asymptomatic | Boosted | 17.82 | 5 | 12 |
| BA.5 | 292073 | male | 22 | Mild | Fully Vaccinated | 17.81 | 4 | 10 |
| BA.5 | 294990 | female | 53 | Asymptomatic | Fully Vaccinated | 17.79 | 6 | 12 |
| BA.5 | 295714 | female | 44 | Asymptomatic | Boosted | 17.77 | 2 | 13 |
| BA.5 | 294301 | female | 32 | Asymptomatic | Unvaccinated | 17.76 | 5 | 15 |

| BA.5 | 294449 | male | 46 | Asymptomatic | Boosted | 17.74 | 2 | 11 |
| --- | --- | --- | --- | --- | --- | --- | --- | --- |
| BA.5 | 294510 | male | 20 | Mild | Boosted | 17.74 | 3 | 13 |
| BA.5 | 295129 | male | 43 | Mild | Boosted | 17.74 | 5 | 14 |
| BA.5 | 294535 | male | 24 | Asymptomatic | Boosted | 17.73 | 2 | 12 |
| BA.5 | 294099 | male | 60 | Mild | Fully Vaccinated | 17.71 | 5 | 14 |
| BA.5 | 294141 | male | 27 | Asymptomatic | Fully Vaccinated | 17.71 | 5 | 11 |
| BA.5 | 294888 | female | 24 | Mild | Boosted | 17.66 | 6 | 15 |
| BA.5 | 295070 | female | 13 | Mild | Fully Vaccinated | 17.66 | 2 | 9 |
| BA.5 | 295712 | male | 35 | Asymptomatic | Boosted | 17.66 | 2 | 13 |
| BA.5 | 294972 | male | 26 | Mild | Boosted | 17.65 | 3 | 9 |
| BA.5 | 294925 | female | 49 | Mild | Boosted | 17.64 | 6 | 12 |
| BA.5 | 295388 | male | 33 | Mild | Fully Vaccinated | 17.64 | 7 | 13 |
| BA.5 | 295607 | male | 52 | Asymptomatic | Boosted | 17.62 | 2 | 12 |
| BA.5 | 295256 | female | 36 | Mild | Fully Vaccinated | 17.61 | 6 | 13 |
| BA.5 | 295183 | male | 25 | Mild | Boosted | 17.58 | 5 | 11 |
| BA.5 | 295390 | female | 37 | Mild | Boosted | 17.58 | 5 | 11 |
| BA.5 | 294492 | female | 32 | Mild | Boosted | 17.57 | 3 | 12 |
| BA.5 | 295035 | female | 12 | Mild | Fully Vaccinated | 17.57 | 2 | 10 |
| BA.5 | 295247 | male | 37 | Mild | Boosted | 17.56 | 6 | 12 |
| BA.5 | 294332 | female | 26 | Mild | Boosted | 17.55 | 2 | 15 |
| BA.5 | 294684 | male | 58 | Mild | Boosted | 17.45 | 4 | 12 |
| BA.5 | 294917 | male | 11 | Mild | Fully Vaccinated | 17.43 | 3 | 11 |
| BA.5 | 295748 | male | 53 | Asymptomatic | Boosted | 17.43 | 5 | 10 |
| BA.5 | 293347 | female | 21 | Moderate | Boosted | 17.42 | 5 | 10 |
| BA.5 | 294677 | male | 19 | Asymptomatic | Boosted | 17.37 | 4 | 8 |
| BA.5 | 294584 | male | 27 | Mild | Fully Vaccinated | 17.35 | 3 | 15 |
| BA.5 | 295130 | female | 44 | Mild | Boosted | 17.35 | 3 | 12 |
| BA.5 | 288572 | female | 20 | Asymptomatic | Boosted | 17.33 | 3 | 10 |
| BA.5 | 289528 | male | 54 | Mild | Fully Vaccinated | 17.33 | 4 | 13 |
| BA.5 | 294528 | female | 41 | Mild | Fully Vaccinated | 17.32 | 5 | 14 |
| BA.5 | 295193 | female | 30 | Mild | Boosted | 17.31 | 3 | 12 |
| BA.5 | 295090 | female | 67 | Asymptomatic | Unknown | 17.3 | 8 | 19 |

| BA.5 | 292406 | female | 25 | Mild | Boosted | 17.29 | 3 | 10 |
| --- | --- | --- | --- | --- | --- | --- | --- | --- |
| BA.5 | 294893 | female | 21 | Mild | Boosted | 17.26 | 2 | 13 |
| BA.5 | 294385 | female | 50 | Asymptomatic | Boosted | 17.25 | 2 | 11 |
| BA.5 | 288532 | male | 24 | Asymptomatic | Boosted | 17.24 | 2 | 13 |
| BA.5 | 294534 | female | 24 | Mild | Boosted | 17. 19 | 3 | 10 |
| BA.5 | 294485 | female | 78 | Asymptomatic | Boosted | 17. 17 | 5 | 14 |
| BA.5 | 295368 | male | 32 | Asymptomatic | Unknown | 17. 17 | 6 | 13 |
| BA.5 | 295721 | female | 58 | Mild | Boosted | 17. 16 | 4 | 16 |
| BA.5 | 290451 | male | 27 | Mild | Boosted | 17. 15 | 3 | 12 |
| BA.5 | 291753 | female | 34 | Mild | Boosted | 17. 15 | 5 | 12 |
| BA.5 | 294554 | male | 60 | Mild | Boosted | 17. 15 | 5 | 12 |
| BA.5 | 295048 | male | 58 | Mild | Boosted | 17. 13 | 4 | 9 |
| BA.5 | 291091 | female | 32 | Mild | Boosted | 17. 11 | 4 | 11 |
| BA.5 | 291502 | male | 44 | Asymptomatic | Boosted | 17. 11 | 5 | 13 |
| BA.5 | 294898 | male | 60 | Asymptomatic | Boosted | 17.08 | 3 | 11 |
| BA.5 | 295709 | male | 32 | Asymptomatic | Fully Vaccinated | 17.05 | 6 | 14 |
| BA.5 | 295379 | male | 22 | Asymptomatic | Fully Vaccinated | 17.04 | 4 | 11 |
| BA.5 | 294902 | male | 16 | Asymptomatic | Unvaccinated | 17.03 | 6 | 18 |
| BA.5 | 295546 | male | 31 | Asymptomatic | Fully Vaccinated | 17.03 | 2 | 13 |
| BA.5 | 294951 | male | 34 | Mild | Boosted | 17.02 | 3 | 9 |
| BA.5 | 293868 | male | 29 | Asymptomatic | Boosted | 17.01 | 6 | 12 |
| BA.5 | 294623 | male | 46 | Asymptomatic | Boosted | 17.01 | 3 | 12 |
| BA.5 | 294652 | male | 39 | Mild | Boosted | 16.99 | 4 | 10 |
| BA.5 | 294744 | female | 41 | Asymptomatic | Boosted | 16.96 | 2 | 8 |
| BA.5 | 294885 | male | 51 | Asymptomatic | Boosted | 16.96 | 3 | 10 |
| BA.5 | 295791 | male | 50 | Mild | Boosted | 16.96 | 3 | 14 |
| BA.5 | 291444 | female | 24 | Mild | Boosted | 16.94 | 5 | 10 |
| BA.5 | 294975 | male | 62 | Mild | Unvaccinated | 16.93 | 3 | 12 |
| BA.5 | 295664 | male | 10 | Mild | Fully Vaccinated | 16.93 | 3 | 10 |
| BA.5 | 294882 | female | 70 | Mild | Boosted | 16.92 | 4 | 13 |
| BA.5 | 295396 | female | 30 | Mild | Boosted | 16.91 | 3 | 9 |
| BA.5 | 294558 | female | 42 | Asymptomatic | Boosted | 16.9 | 3 | 10 |

| BA.5 | 295408 | male | 25 | Mild | Fully Vaccinated | 16.87 | 2 | 8 |
| --- | --- | --- | --- | --- | --- | --- | --- | --- |
| BA.5 | 295194 | female | 45 | Mild | Boosted | 16.85 | 3 | 12 |
| BA.5 | 294624 | male | 54 | Mild | Boosted | 16.83 | 3 | 14 |
| BA.5 | 295510 | female | 16 | Moderate | Fully Vaccinated | 16.83 | 5 | 12 |
| BA.5 | 295733 | female | 34 | Asymptomatic | Boosted | 16.83 | 5 | 14 |
| BA.5 | 294903 | male | 22 | Asymptomatic | Boosted | 16.82 | 4 | 13 |
| BA.5 | 294944 | female | 56 | Asymptomatic | Boosted | 16.81 | 6 | 12 |
| BA.5 | 292421 | female | 21 | Mild | Fully Vaccinated | 16.78 | 3 | 12 |
| BA.5 | 295072 | female | 54 | Asymptomatic | Boosted | 16.75 | 2 | 9 |
| BA.5 | 294443 | male | 51 | Asymptomatic | Unknown | 16.74 | 2 | 12 |
| BA.5 | 294962 | male | 62 | Mild | Fully Vaccinated | 16.74 | 5 | 19 |
| BA.5 | 295346 | male | 45 | Moderate | Boosted | 16.7 | 2 | 13 |
| BA.5 | 294368 | female | 28 | Asymptomatic | Boosted | 16.69 | 4 | 12 |
| BA.5 | 294386 | male | 24 | Asymptomatic | Boosted | 16.69 | 5 | 12 |
| BA.5 | 294347 | female | 31 | Asymptomatic | Boosted | 16.68 | 3 | 14 |
| BA.5 | 294685 | female | 36 | Asymptomatic | Unvaccinated | 16.68 | 3 | 14 |
| BA.5 | 294783 | male | 44 | Mild | Fully Vaccinated | 16.68 | 2 | 13 |
| BA.5 | 295373 | female | 38 | Asymptomatic | Unknown | 16.68 | 3 | 10 |
| BA.5 | 294089 | male | 29 | Mild | Boosted | 16.67 | 6 | 12 |
| BA.5 | 292048 | male | 50 | Mild | Boosted | 16.64 | 2 | 9 |
| BA.5 | 294100 | male | 29 | Mild | Boosted | 16.64 | 1 | 11 |
| BA.5 | 295377 | male | 34 | Moderate | Unvaccinated | 16.64 | 4 | 14 |
| BA.5 | 294966 | female | 47 | Asymptomatic | Boosted | 16.63 | 5 | 11 |
| BA.5 | 295057 | male | 37 | Mild | Unknown | 16.63 | 6 | 15 |
| BA.5 | 295364 | female | 46 | Mild | Boosted | 16.63 | 3 | 10 |
| BA.5 | 294627 | male | 71 | Moderate | Boosted | 16.62 | 3 | 11 |
| BA.5 | 295249 | female | 34 | Mild | Boosted | 16.62 | 2 | 8 |
| BA.5 | 295114 | female | 37 | Mild | Fully Vaccinated | 16.59 | 3 | 14 |
| BA.5 | 295704 | male | 33 | Asymptomatic | Boosted | 16.57 | 5 | 13 |
| BA.5 | 295081 | female | 54 | Mild | Boosted | 16.56 | 4 | 11 |
| BA.5 | 295378 | female | 53 | Mild | Boosted | 16.55 | 3 | 13 |
| BA.5 | 294838 | male | 68 | Moderate | Unknown | 16.54 | 5 | 21 |

| BA.5 | 294526 | male | 63 | Asymptomatic | Boosted | 16.53 | 5 | 14 |
| --- | --- | --- | --- | --- | --- | --- | --- | --- |
| BA.5 | 292181 | male | 23 | Mild | Fully Vaccinated | 16.52 | 4 | 16 |
| BA.5 | 295094 | female | 31 | Mild | Unknown | 16.49 | 4 | 17 |
| BA.5 | 295112 | female | 24 | Mild | Unknown | 16.47 | 5 | 15 |
| BA.5 | 295185 | female | 52 | Mild | Boosted | 16.45 | 7 | 15 |
| BA.5 | 295222 | female | 14 | Mild | Fully Vaccinated | 16.44 | 2 | 10 |
| BA.5 | 294431 | female | 55 | Mild | Boosted | 16.42 | 3 | 12 |
| BA.5 | 294868 | female | 34 | Asymptomatic | Boosted | 16.4 | 2 | 13 |
| BA.5 | 290761 | male | 10 | Mild | Fully Vaccinated | 16.38 | 2 | 7 |
| BA.5 | 294406 | female | 23 | Mild | Boosted | 16.38 | 2 | 7 |
| BA.5 | 294408 | female | 15 | Mild | Fully Vaccinated | 16.36 | 6 | 14 |
| BA.5 | 295610 | female | 53 | Mild | Boosted | 16.35 | 2 | 12 |
| BA.5 | 295711 | male | 44 | Mild | Boosted | 16.35 | 4 | 13 |
| BA.5 | 294982 | male | 57 | Mild | Boosted | 16.34 | 5 | 14 |
| BA.5 | 295347 | male | 30 | Mild | Boosted | 16.34 | 2 | 11 |
| BA.5 | 294730 | female | 53 | Asymptomatic | Boosted | 16.33 | 5 | 11 |
| BA.5 | 294957 | male | 46 | Mild | Boosted | 16.32 | 3 | 16 |
| BA.5 | 295728 | male | 22 | Mild | Boosted | 16.32 | 4 | 14 |
| BA.5 | 293777 | female | 26 | Mild | Boosted | 16.29 | 4 | 13 |
| BA.5 | 293986 | female | 42 | Mild | Boosted | 16.29 | 3 | 14 |
| BA.5 | 294444 | female | 24 | Mild | Fully Vaccinated | 16.28 | 3 | 12 |
| BA.5 | 295029 | female | 46 | Asymptomatic | Boosted | 16.27 | 3 | 13 |
| BA.5 | 295031 | male | 46 | Mild | Unvaccinated | 16.27 | 2 | 14 |
| BA.5 | 295061 | female | 50 | Mild | Boosted | 16.27 | 3 | 10 |
| BA.5 | 294204 | male | 49 | Mild | Boosted | 16.26 | 3 | 14 |
| BA.5 | 294597 | female | 58 | Asymptomatic | Boosted | 16.26 | 4 | 11 |
| BA.5 | 295074 | female | 43 | Mild | Boosted | 16.26 | 4 | 11 |
| BA.5 | 290970 | male | 33 | Mild | Unvaccinated | 16.24 | 3 | 14 |
| BA.5 | 294741 | female | 59 | Asymptomatic | Boosted | 16.24 | 3 | 11 |
| BA.5 | 295177 | female | 30 | Asymptomatic | Boosted | 16.24 | 2 | 14 |
| BA.5 | 292419 | female | 53 | Asymptomatic | Fully Vaccinated | 16.23 | 4 | 12 |
| BA.5 | 294559 | female | 59 | Mild | Unknown | 16.23 | 3 | 15 |

| BA.5 | 295696 | male | 59 | Mild | Boosted | 16.22 | 2 | 10 |
| --- | --- | --- | --- | --- | --- | --- | --- | --- |
| BA.5 | 294506 | male | 46 | Moderate | Boosted | 16.21 | 4 | 11 |
| BA.5 | 295389 | female | 25 | Mild | Boosted | 16.21 | 3 | 14 |
| BA.5 | 295375 | female | 67 | Mild | Unknown | 16.2 | 5 | 13 |
| BA.5 | 291918 | male | 28 | Mild | Boosted | 16. 18 | 5 | 13 |
| BA.5 | 295079 | male | 48 | Mild | Boosted | 16. 18 | 3 | 13 |
| BA.5 | 294794 | female | 44 | Moderate | Boosted | 16. 16 | 2 | 10 |
| BA.5 | 294904 | female | 47 | Asymptomatic | Unknown | 16. 16 | 7 | 16 |
| BA.5 | 295500 | male | 39 | Mild | Boosted | 16. 16 | 5 | 9 |
| BA.5 | 295015 | female | 36 | Mild | Boosted | 16. 15 | 5 | 11 |
| BA.5 | 295614 | male | 31 | Mild | Boosted | 16. 15 | 4 | 13 |
| BA.5 | 294442 | male | 43 | Mild | Boosted | 16. 14 | 5 | 16 |
| BA.5 | 294585 | male | 13 | Mild | Boosted | 16. 12 | 3 | 10 |
| BA.5 | 291495 | male | 25 | Mild | Boosted | 16. 11 | 3 | 9 |
| BA.5 | 295148 | female | 58 | Mild | Boosted | 16. 11 | 3 | 10 |
| BA.5 | 295706 | male | 53 | Mild | Fully Vaccinated | 16. 1 | 5 | 13 |
| BA.5 | 294250 | female | 54 | Mild | Boosted | 16.09 | 5 | 15 |
| BA.5 | 295817 | male | 30 | Mild | Boosted | 16.09 | 2 | 9 |
| BA.5 | 294323 | female | 36 | Asymptomatic | Unknown | 16.08 | 5 | 10 |
| BA.5 | 295063 | male | 85 | Asymptomatic | Boosted | 16.06 | 3 | 17 |
| BA.5 | 295753 | male | 21 | Mild | Boosted | 16.06 | 4 | 9 |
| BA.5 | 289508 | male | 39 | Mild | Boosted | 16.05 | 3 | 11 |
| BA.5 | 294674 | female | 9 | Mild | Fully Vaccinated | 16.05 | 3 | 11 |
| BA.5 | 295528 | male | 37 | Mild | Boosted | 16.05 | 3 | 10 |
| BA.5 | 295592 | female | 53 | Mild | Unknown | 16.05 | 3 | 12 |
| BA.5 | 295601 | female | 21 | Moderate | Boosted | 16.05 | 2 | 12 |
| BA.5 | 295713 | male | 54 | Mild | Boosted | 16.05 | 4 | 12 |
| BA.5 | 294251 | male | 13 | Mild | Fully Vaccinated | 16.04 | 5 | 13 |
| BA.5 | 295308 | male | 41 | Mild | Unvaccinated | 16.03 | 2 | 21 |
| BA.5 | 295787 | female | 33 | Asymptomatic | Boosted | 16.03 | 3 | 11 |
| BA.5 | 294593 | male | 45 | Asymptomatic | Boosted | 16.02 | 5 | 16 |
| BA.5 | 294594 | male | 52 | Asymptomatic | Boosted | 16.02 | 2 | 12 |

| BA.5 | 295271 | female | 40 | Mild | Boosted | 16.02 | 4 | 12 |
| --- | --- | --- | --- | --- | --- | --- | --- | --- |
| BA.5 | 294562 | male | 59 | Asymptomatic | Boosted | 16.01 | 6 | 15 |
| BA.5 | 294961 | female | 44 | Mild | Boosted | 15.99 | 3 | 12 |
| BA.5 | 295705 | female | 27 | Mild | Fully Vaccinated | 15.98 | 2 | 10 |
| BA.5 | 294759 | female | 24 | Mild | Boosted | 15.96 | 3 | 13 |
| BA.5 | 295586 | male | 56 | Mild | Boosted | 15.96 | 4 | 12 |
| BA.5 | 69911 | female | 59 | Mild | Boosted | 15.92 | 3 | 12 |
| BA.5 | 294665 | female | 38 | Mild | Fully Vaccinated | 15.91 | 2 | 14 |
| BA.5 | 294335 | female | 55 | Mild | Boosted | 15.89 | 5 | 13 |
| BA.5 | 294848 | female | 45 | Asymptomatic | Unvaccinated | 15.87 | 4 | 20 |
| BA.5 | 295779 | male | 3 | Mild | Fully Vaccinated | 15.86 | 3 | 10 |
| BA.5 | 294719 | female | 31 | Asymptomatic | Boosted | 15.84 | 4 | 17 |
| BA.5 | 294803 | female | 41 | Mild | Boosted | 15.84 | 2 | 9 |
| BA.5 | 294806 | male | 6 | Asymptomatic | Unvaccinated | 15.84 | 4 | 12 |
| BA.5 | 294940 | female | 71 | Asymptomatic | Boosted | 15.84 | 5 | 15 |
| BA.5 | 295038 | male | 12 | Mild | Fully Vaccinated | 15.84 | 2 | 9 |
| BA.5 | 292633 | male | 37 | Mild | Boosted | 15.83 | 4 | 12 |
| BA.5 | 295499 | male | 72 | Asymptomatic | Unknown | 15.83 | 4 | 16 |
| BA.5 | 295719 | female | 38 | Asymptomatic | Boosted | 15.83 | 5 | 12 |
| BA.5 | 294771 | female | 55 | Mild | Boosted | 15.82 | 3 | 12 |
| BA.5 | 295503 | female | 30 | Moderate | Boosted | 15.81 | 3 | 15 |
| BA.5 | 294826 | female | 50 | Asymptomatic | Unvaccinated | 15.8 | 8 | 14 |
| BA.5 | 295188 | female | 50 | Mild | Boosted | 15.78 | 6 | 18 |
| BA.5 | 294537 | female | 52 | Asymptomatic | Boosted | 15.74 | 2 | 11 |
| BA.5 | 291064 | female | 33 | Mild | Fully Vaccinated | 15.73 | 5 | 11 |
| BA.5 | 294740 | female | 23 | Mild | Boosted | 15.71 | 1 | 8 |
| BA.5 | 291501 | male | 39 | Asymptomatic | Boosted | 15.69 | 6 | 14 |
| BA.5 | 294953 | female | 51 | Asymptomatic | Fully Vaccinated | 15.69 | 3 | 11 |
| BA.5 | 294906 | male | 29 | Asymptomatic | Boosted | 15.67 | 2 | 11 |
| BA.5 | 295738 | male | 7 | Mild | Unvaccinated | 15.67 | 2 | 10 |
| BA.5 | 295076 | male | 11 | Mild | Unknown | 15.66 | 3 | 10 |
| BA.5 | 294298 | male | 43 | Moderate | Boosted | 15.65 | 3 | 13 |

| BA.5 | 294747 | female | 67 | Asymptomatic | Boosted | 15.64 | 4 | 11 |
| --- | --- | --- | --- | --- | --- | --- | --- | --- |
| BA.5 | 294963 | female | 29 | Mild | Boosted | 15.64 | 3 | 11 |
| BA.5 | 294340 | male | 51 | Asymptomatic | Boosted | 15.63 | 4 | 9 |
| BA.5 | 295180 | female | 46 | Mild | Fully Vaccinated | 15.63 | 3 | 7 |
| BA.5 | 294587 | male | 28 | Asymptomatic | Boosted | 15.6 | 3 | 10 |
| BA.5 | 294682 | male | 48 | Mild | Boosted | 15.58 | 2 | 9 |
| BA.5 | 295037 | female | 10 | Mild | Unknown | 15.58 | 3 | 10 |
| BA.5 | 295054 | female | 50 | Mild | Boosted | 15.58 | 2 | 15 |
| BA.5 | 294441 | female | 29 | Asymptomatic | Unvaccinated | 15.52 | 5 | 13 |
| BA.5 | 295593 | male | 30 | Moderate | Boosted | 15.52 | 4 | 12 |
| BA.5 | 294312 | male | 40 | Mild | Boosted | 15.5 | 3 | 10 |
| BA.5 | 294319 | male | 45 | Mild | Boosted | 15.5 | 5 | 15 |
| BA.5 | 295598 | male | 57 | Mild | Boosted | 15.5 | 4 | 13 |
| BA.5 | 295203 | female | 54 | Mild | Fully Vaccinated | 15.49 | 4 | 18 |
| BA.5 | 295393 | female | 52 | Mild | Fully Vaccinated | 15.47 | 5 | 14 |
| BA.5 | 295244 | male | 16 | Mild | Fully Vaccinated | 15.45 | 5 | 16 |
| BA.5 | 294200 | male | 41 | Mild | Boosted | 15.44 | 3 | 13 |
| BA.5 | 294244 | male | 50 | Asymptomatic | Boosted | 15.43 | 6 | 13 |
| BA.5 | 294476 | female | 28 | Mild | Boosted | 15.42 | 3 | 9 |
| BA.5 | 295077 | male | 58 | Asymptomatic | Unknown | 15.39 | 3 | 18 |
| BA.5 | 294999 | male | 63 | Asymptomatic | Unknown | 15.36 | 3 | 10 |
| BA.5 | 294409 | female | 32 | Asymptomatic | Unknown | 15.31 | 5 | 16 |
| BA.5 | 295402 | male | 38 | Mild | Boosted | 15.29 | 5 | 14 |
| BA.5 | 295731 | male | 10 | Mild | Fully Vaccinated | 15.29 | 2 | 10 |
| BA.5 | 295058 | female | 39 | Asymptomatic | Fully Vaccinated | 15.27 | 2 | 9 |
| BA.5 | 294960 | female | 30 | Mild | Boosted | 15.26 | 2 | 12 |
| BA.5 | 295118 | female | 39 | Mild | Boosted | 15.26 | 4 | 9 |
| BA.5 | 294700 | male | 15 | Asymptomatic | Fully Vaccinated | 15.25 | 4 | 15 |
| BA.5 | 295405 | female | 22 | Asymptomatic | Boosted | 15.25 | 6 | 11 |
| BA.5 | 294383 | female | 23 | Mild | Boosted | 15.24 | 3 | 11 |
| BA.5 | 295656 | male | 38 | Mild | Unvaccinated | 15.23 | 3 | 11 |
| BA.5 | 294778 | female | 56 | Mild | Boosted | 15.22 | 2 | 13 |

| BA.5 | 294938 | female | 23 | Mild | Boosted | 15.22 | 2 | 11 |
| --- | --- | --- | --- | --- | --- | --- | --- | --- |
| BA.5 | 295034 | male | 11 | Mild | Fully Vaccinated | 15.22 | 2 | 9 |
| BA.5 | 291696 | male | 28 | Mild | Fully Vaccinated | 15.21 | 4 | 16 |
| BA.5 | 293760 | female | 22 | Mild | Boosted | 15.21 | 2 | 17 |
| BA.5 | 294761 | male | 12 | Mild | Fully Vaccinated | 15.21 | 2 | 13 |
| BA.5 | 295832 | female | 3 | Mild | Unvaccinated | 15.21 | 2 | 9 |
| BA.5 | 294546 | male | 4 | Mild | Unvaccinated | 15.2 | 3 | 9 |
| BA.5 | 294339 | female | 56 | Mild | Boosted | 15. 19 | 3 | 12 |
| BA.5 | 295600 | male | 36 | Mild | Boosted | 15. 19 | 4 | 12 |
| BA.5 | 288530 | male | 53 | Mild | Boosted | 15. 17 | 2 | 13 |
| BA.5 | 294302 | female | 63 | Asymptomatic | Boosted | 15. 15 | 3 | 15 |
| BA.5 | 294304 | female | 32 | Mild | Boosted | 15. 15 | 5 | 15 |
| BA.5 | 294519 | male | 70 | Asymptomatic | Boosted | 15. 15 | 2 | 10 |
| BA.5 | 295448 | male | 48 | Mild | Boosted | 15. 14 | 6 | 17 |
| BA.5 | 295127 | female | 57 | Mild | Fully Vaccinated | 15. 13 | 3 | 11 |
| BA.5 | 294580 | female | 49 | Mild | Boosted | 15. 12 | 3 | 15 |
| BA.5 | 294581 | male | 33 | Asymptomatic | Boosted | 15. 12 | 3 | 16 |
| BA.5 | 294626 | female | 29 | Asymptomatic | Fully Vaccinated | 15. 12 | 5 | 12 |
| BA.5 | 294930 | female | 54 | Mild | Boosted | 15. 12 | 5 | 18 |
| BA.5 | 295087 | female | 33 | Asymptomatic | Boosted | 15. 12 | 3 | 12 |
| BA.5 | 294669 | male | 31 | Mild | Unknown | 15. 11 | 4 | 9 |
| BA.5 | 294977 | female | 32 | Mild | Boosted | 15. 11 | 3 | 12 |
| BA.5 | 294746 | female | 36 | Asymptomatic | Unvaccinated | 15. 1 | 3 | 15 |
| BA.5 | 295505 | male | 58 | Asymptomatic | Unvaccinated | 15. 1 | 3 | 20 |
| BA.5 | 291662 | male | 47 | Mild | Boosted | 15.09 | 5 | 10 |
| BA.5 | 291500 | male | 54 | Mild | Boosted | 15.07 | 5 | 16 |
| BA.5 | 294777 | male | 64 | Mild | Fully Vaccinated | 15.05 | 3 | 17 |
| BA.5 | 294734 | male | 47 | Mild | Boosted | 15.03 | 3 | 12 |
| BA.5 | 294752 | female | 54 | Asymptomatic | Unknown | 15.03 | 3 | 17 |
| BA.5 | 295394 | male | 51 | Mild | Boosted | 15.03 | 5 | 14 |
| BA.5 | 295195 | female | 46 | Mild | Boosted | 15.02 | 3 | 11 |
| BA.5 | 294450 | male | 27 | Mild | Unvaccinated | 14.99 | 2 | 14 |

| BA.5 | 295585 | male | 52 | Asymptomatic | Boosted | 14.97 | 4 | 12 |
| --- | --- | --- | --- | --- | --- | --- | --- | --- |
| BA.5 | 294604 | male | 42 | Asymptomatic | Boosted | 14.96 | 6 | 17 |
| BA.5 | 294795 | female | 57 | Asymptomatic | Boosted | 14.95 | 2 | 11 |
| BA.5 | 294875 | male | 34 | Asymptomatic | Boosted | 14.95 | 6 | 12 |
| BA.5 | 295181 | female | 49 | Moderate | Boosted | 14.9 | 2 | 12 |
| BA.5 | 295344 | male | 22 | Mild | Boosted | 14.88 | 2 | 12 |
| BA.5 | 294891 | female | 50 | Mild | Boosted | 14.87 | 3 | 16 |
| BA.5 | 294369 | female | 39 | Mild | Boosted | 14.83 | 3 | 15 |
| BA.5 | 295597 | female | 51 | Mild | Unvaccinated | 14.83 | 4 | 17 |
| BA.5 | 295184 | female | 43 | Mild | Boosted | 14.82 | 4 | 11 |
| BA.5 | 294850 | female | 57 | Mild | Boosted | 14.8 | 3 | 12 |
| BA.5 | 294937 | female | 51 | Mild | Fully Vaccinated | 14.79 | 1 | 16 |
| BA.5 | 294739 | female | 57 | Mild | Boosted | 14.78 | 3 | 15 |
| BA.5 | 295113 | female | 30 | Mild | Unknown | 14.76 | 2 | 13 |
| BA.5 | 294690 | male | 33 | Moderate | Boosted | 14.75 | 4 | 14 |
| BA.5 | 294831 | male | 80 | Asymptomatic | Boosted | 14.74 | 2 | 12 |
| BA.5 | 295549 | male | 52 | Mild | Boosted | 14.72 | 4 | 9 |
| BA.5 | 295356 | female | 20 | Asymptomatic | Unknown | 14.67 | 2 | 12 |
| BA.5 | 294495 | female | 53 | Asymptomatic | Boosted | 14.65 | 3 | 14 |
| BA.5 | 294792 | female | 44 | Mild | Boosted | 14.65 | 2 | 13 |
| BA.5 | 295128 | female | 48 | Mild | Boosted | 14.65 | 3 | 12 |
| BA.5 | 295737 | female | 52 | Mild | Boosted | 14.64 | 2 | 18 |
| BA.5 | 294681 | female | 56 | Mild | Boosted | 14.62 | 5 | 13 |
| BA.5 | 294905 | male | 48 | Asymptomatic | Boosted | 14.62 | 7 | 15 |
| BA.5 | 295357 | female | 27 | Mild | Boosted | 14.62 | 3 | 15 |
| BA.5 | 295224 | male | 33 | Asymptomatic | Unknown | 14.59 | 3 | 11 |
| BA.5 | 294909 | male | 34 | Mild | Boosted | 14.53 | 5 | 12 |
| BA.5 | 291811 | male | 33 | Mild | Boosted | 14.52 | 3 | 11 |
| BA.5 | 294678 | male | 46 | Mild | Boosted | 14.48 | 4 | 11 |
| BA.5 | 294894 | male | 50 | Mild | Boosted | 14.45 | 6 | 19 |
| BA.5 | 294548 | female | 45 | Mild | Boosted | 14.41 | 4 | 14 |
| BA.5 | 294974 | male | 39 | Mild | Boosted | 14.41 | 3 | 15 |

| BA.5 | 295232 | female | 47 | Mild | Boosted | 14.4 | 2 | 16 |
| --- | --- | --- | --- | --- | --- | --- | --- | --- |
| BA.5 | 294101 | female | 25 | Moderate | Boosted | 14.39 | 2 | 13 |
| BA.5 | 295230 | female | 87 | Asymptomatic | Unvaccinated | 14.38 | 4 | 6 |
| BA.5 | 295745 | female | 36 | Mild | Boosted | 14.38 | 6 | 14 |
| BA.5 | 294916 | male | 9 | Mild | Fully Vaccinated | 14.36 | 3 | 17 |
| BA.5 | 294378 | female | 40 | Mild | Boosted | 14.35 | 4 | 11 |
| BA.5 | 295175 | female | 24 | Mild | Boosted | 14.34 | 3 | 17 |
| BA.5 | 291494 | male | 35 | Mild | Boosted | 14.31 | 5 | 16 |
| BA.5 | 294959 | female | 55 | Mild | Boosted | 14.29 | 5 | 16 |
| BA.5 | 295197 | male | 37 | Moderate | Unvaccinated | 14.29 | 4 | 13 |
| BA.5 | 294889 | female | 22 | Mild | Boosted | 14.27 | 3 | 12 |
| BA.5 | 294411 | female | 60 | Asymptomatic | Unknown | 14.26 | 5 | 14 |
| BA.5 | 292124 | male | 40 | Asymptomatic | Boosted | 14.24 | 4 | 14 |
| BA.5 | 294203 | female | 25 | Mild | Fully Vaccinated | 14.24 | 5 | 16 |
| BA.5 | 294410 | male | 65 | Asymptomatic | Boosted | 14.24 | 5 | 16 |
| BA.5 | 294657 | female | 36 | Mild | Boosted | 14.22 | 3 | 10 |
| BA.5 | 288822 | male | 20 | Mild | Fully Vaccinated | 14.21 | 2 | 10 |
| BA.5 | 294249 | female | 9 | Mild | Fully Vaccinated | 14.2 | 3 | 12 |
| BA.5 | 295040 | male | 20 | Asymptomatic | Boosted | 14. 13 | 2 | 15 |
| BA.5 | 294950 | male | 33 | Mild | Boosted | 14. 1 | 3 | 20 |
| BA.5 | 295477 | female | 48 | Mild | Unknown | 14. 1 | 2 | 14 |
| BA.5 | 294726 | female | 50 | Mild | Fully Vaccinated | 14.09 | 2 | 12 |
| BA.5 | 295064 | female | 81 | Asymptomatic | Fully Vaccinated | 14.09 | 3 | 17 |
| BA.5 | 292078 | female | 25 | Mild | Unvaccinated | 14.06 | 3 | 11 |
| BA.5 | 294296 | male | 36 | Asymptomatic | Boosted | 14.06 | 5 | 18 |
| BA.5 | 294427 | female | 30 | Asymptomatic | Boosted | 14.06 | 4 | 13 |
| BA.5 | 294955 | male | 35 | Mild | Boosted | 14.06 | 2 | 10 |
| BA.5 | 295490 | male | 47 | Mild | Boosted | 14.04 | 3 | 13 |
| BA.5 | 294822 | female | 56 | Mild | Unknown | 14.03 | 2 | 16 |
| BA.5 | 292653 | male | 30 | Mild | Fully Vaccinated | 14.02 | 6 | 11 |
| BA.5 | 294199 | male | 50 | Mild | Boosted | 14 | 5 | 14 |
| BA.5 | 295009 | male | 49 | Mild | Boosted | 14 | 6 | 14 |

| BA.5 | 295187 | female | 47 | Mild | Boosted | 13.99 | 2 | 14 |
| --- | --- | --- | --- | --- | --- | --- | --- | --- |
| BA.5 | 294923 | female | 63 | Mild | Fully Vaccinated | 13.97 | 6 | 16 |
| BA.5 | 295047 | male | 30 | Mild | Boosted | 13.96 | 3 | 9 |
| BA.5 | 288707 | female | 18 | Moderate | Boosted | 13.95 | 2 | 12 |
| BA.5 | 294384 | male | 64 | Asymptomatic | Fully Vaccinated | 13.88 | 4 | 11 |
| BA.5 | 295231 | female | 27 | Mild | Boosted | 13.86 | 4 | 13 |
| BA.5 | 295288 | female | 57 | Moderate | Boosted | 13.84 | 3 | 17 |
| BA.5 | 295608 | male | 14 | Mild | Fully Vaccinated | 13.83 | 2 | 13 |
| BA.5 | 291860 | female | 19 | Mild | Boosted | 13.82 | 5 | 13 |
| BA.5 | 292222 | male | 59 | Mild | Boosted | 13.8 | 2 | 8 |
| BA.5 | 294596 | female | 43 | Mild | Boosted | 13.69 | 3 | 17 |
| BA.5 | 294295 | female | 64 | Moderate | Fully Vaccinated | 13.67 | 2 | 16 |
| BA.5 | 295182 | female | 46 | Moderate | Boosted | 13.62 | 3 | 12 |
| BA.5 | 294736 | male | 40 | Asymptomatic | Boosted | 13.58 | 3 | 12 |
| BA.5 | 295406 | female | 25 | Asymptomatic | Boosted | 13.56 | 5 | 13 |
| BA.5 | 294076 | male | 31 | Mild | Boosted | 13.52 | 2 | 9 |
| BA.5 | 294565 | female | 49 | Asymptomatic | Unknown | 13.51 | 3 | 14 |
| BA.5 | 294590 | female | 31 | Moderate | Boosted | 13.5 | 5 | 12 |
| BA.5 | 295100 | female | 52 | Moderate | Boosted | 13.42 | 3 | 9 |
| BA.5 | 295198 | female | 27 | Mild | Boosted | 13.38 | 3 | 11 |
| BA.5 | 295069 | female | 29 | Mild | Boosted | 13.3 | 2 | 9 |
| BA.5 | 294651 | male | 56 | Asymptomatic | Boosted | 13.24 | 5 | 17 |
| BA.5 | 290955 | male | 52 | Mild | Boosted | 13. 19 | 4 | 11 |
| BA.5 | 294749 | male | 46 | Mild | Unknown | 13. 18 | 4 | 17 |
| BA.5 | 293746 | female | 28 | Mild | Boosted | 13. 17 | 4 | 12 |
| BA.5 | 294691 | female | 41 | Asymptomatic | Boosted | 13. 17 | 5 | 16 |
| BA.5 | 294019 | female | 39 | Mild | Boosted | 13. 15 | 2 | 14 |
| BA.5 | 294742 | male | 52 | Mild | Boosted | 13. 15 | 4 | 17 |
| BA.5 | 294090 | female | 29 | Mild | Boosted | 13. 12 | 2 | 9 |
| BA.5 | 294943 | female | 34 | Mild | Unvaccinated | 13. 12 | 4 | 11 |
| BA.5 | 295767 | female | 57 | Asymptomatic | Boosted | 13.09 | 3 | 12 |
| BA.5 | 294696 | female | 46 | Mild | Boosted | 13.02 | 2 | 13 |

| BA.5 | 294091 | male | 29 | Asymptomatic | Boosted | 12.96 | 2 | 9 |
| --- | --- | --- | --- | --- | --- | --- | --- | --- |
| BA 5 | 292615 | male | 36 | Mild | Boosted | 12 9 | 4 | 12 |
| BA 5 | 294956 | female | 57 | Asymptomatic | Unknown | 12 8 | 6 | 15 |
| BA.5 | 295768 | female | 9 | Asymptomatic | Fully Vaccinated | 12.8 | 3 | 7 |
| BA 5 | 295131 | female | 10 | Mild | Fully Vaccinated | 12 72 | 3 | 12 |
| BA 5 | 294738 | male | 17 | Mild | Fully Vaccinated | 12 56 | 3 | 12 |
| BA.5 | 288700 | male | 24 | Mild | Unvaccinated | 12.44 | 2 | 9 |
| BA.5 | 292212 | female | 46 | Mild | Fully Vaccinated | 12.3 | 3 | 9 |
| BA 5 | 294625 | male | 48 | Mild | Boosted | 12 29 | 3 | 13 |
| BA.5 | 291075 | male | 38 | Mild | Boosted | 12. 18 | 3 | 12 |
| BA.5 | 295101 | female | 14 | Mild | Fully Vaccinated | 11.97 | 2 | 9 |
| BA 5 | 295059 | female | 40 | Mild | Fully Vaccinated | 11 88 | 2 | 10 |
| BA 5 | 294052 | female | 58 | Mild | Boosted | 11 7 | 3 | 13 |
| BA.5 | 294214 | female | 77 | Mild | Boosted | 11.69 | 4 | 17 |
| BA 5 | 294941 | female | 27 | Mild | Boosted | 11 09 | 3 | 10 |

**Table S6.** **2019_nCoV-IgG** **of** **BA.2** **and** **BA.5**

| **Variants** | **ID** | **Vaccine** | **The** **months** **since** **the** **onset** **of** **the** **last** **dose** **of** **vaccine** | **Days** **to** **onset** | **IgG** **value** |
| --- | --- | --- | --- | --- | --- |
| BA.2 | 282057 | Unvaccinated | NA | 3 | 0.22 |
| BA.2 | 282057 | Unvaccinated | NA | 6 | 0. 14 |
| BA.2 | 281772 | Boosted | ≤ 6 months | 4 | 243.84 |
| BA.2 | 281737 | Boosted | ≤ 6 months | 2 | 317. 16 |
| BA.2 | 281469 | Fully Vaccinated | 7- 12 months | 3 | 3.48 |
| BA.2 | 281371 | Boosted | ≤ 6 months | 4 | 312.86 |
| BA.2 | 281314 | Fully Vaccinated | 7- 12 months | 3 | 2.06 |
| BA.2 | 281313 | Fully Vaccinated | 7- 12 months | 17 | 341.35 |
| BA.2 | 281313 | Fully Vaccinated | 7- 12 months | 3 | 11.86 |
| BA.2 | 281312 | Boosted | ≤ 6 months | 3 | 112.28 |
| BA.2 | 281300 | Fully Vaccinated | 7- 12 months | 2 | 3.6 |
| BA.2 | 281299 | Boosted | ≤ 6 months | 2 | 10.79 |
| BA.2 | 281280 | Boosted | ≤ 6 months | 3 | 38.48 |
| BA.2 | 281258 | Boosted | 7- 12 months | 2 | 47.24 |
| BA.2 | 281257 | Boosted | ≤ 6 months | 2 | 0.75 |
| BA.2 | 281252 | Boosted | ≤ 6 months | 18 | 262.09 |
| BA.2 | 281252 | Boosted | ≤ 6 months | 2 | 100.63 |
| BA.2 | 281251 | Boosted | ≤ 6 months | 5 | 48.69 |
| BA.2 | 281250 | Boosted | ≤ 6 months | 3 | 165.87 |
| BA.2 | 281249 | Boosted | ≤ 6 months | 14 | 431.08 |
| BA.2 | 281249 | Boosted | ≤ 6 months | 4 | 106. 16 |
| BA.2 | 281205 | Boosted | ≤ 6 months | 3 | 254. 16 |
| BA.2 | 281189 | Boosted | ≤ 6 months | 2 | 128.24 |
| BA.2 | 281188 | Boosted | ≤ 6 months | 4 | 333.81 |
| BA.2 | 281187 | Fully Vaccinated | 7- 12 months | 2 | 2.2 |
| BA.2 | 281186 | Boosted | ≤ 6 months | 2 | 158. 18 |

| BA.2 | 281146 | Fully Vaccinated | 7- 12 months | 12 | 418. 15 |
| --- | --- | --- | --- | --- | --- |
| BA.2 | 281146 | Fully Vaccinated | 7- 12 months | 3 | 6.75 |
| BA.2 | 281144 | Fully Vaccinated | 7- 12 months | 11 | 385.52 |
| BA.2 | 281144 | Fully Vaccinated | 7- 12 months | 2 | 3. 13 |
| BA.2 | 281142 | Boosted | Unknown | 11 | 402.44 |
| BA.2 | 281142 | Boosted | Unknown | 2 | 40.27 |
| BA.2 | 281129 | Boosted | ≤ 6 months | 15 | 412.28 |
| BA.2 | 281129 | Boosted | ≤ 6 months | 2 | 43.07 |
| BA.2 | 281128 | Boosted | ≤ 6 months | 2 | 330.68 |
| BA.2 | 281119 | Boosted | ≤ 6 months | 3 | 51.58 |
| BA.2 | 281117 | Boosted | Unknown | 20 | 445. 12 |
| BA.2 | 281117 | Boosted | Unknown | 2 | 12.94 |
| BA.2 | 281114 | Boosted | 7- 12 months | 2 | 17.85 |
| BA.2 | 281113 | Boosted | ≤ 6 months | 2 | 22.27 |
| BA.2 | 281111 | Boosted | 7- 12 months | 2 | 4.07 |
| BA.2 | 281110 | Boosted | ≤ 6 months | 2 | 23.93 |
| BA.2 | 281109 | Fully Vaccinated | 7- 12 months | 2 | 0.5 |
| BA.2 | 281108 | Boosted | ≤ 6 months | 2 | 45.35 |
| BA.2 | 281106 | Boosted | ≤ 6 months | 3 | 158.82 |
| BA.2 | 281105 | Fully Vaccinated | 7- 12 months | 2 | 0.83 |
| BA.2 | 281104 | Boosted | Unknown | 2 | 391.59 |
| BA.2 | 281103 | Boosted | ≤ 6 months | 3 | 111.77 |
| BA.2 | 281101 | Boosted | ≤ 6 months | 3 | 70.45 |
| BA.2 | 281100 | Boosted | ≤ 6 months | 21 | 400.53 |
| BA.2 | 281100 | Boosted | ≤ 6 months | 8 | 91.67 |
| BA.2 | 281100 | Boosted | ≤ 6 months | 4 | 8.79 |
| BA.2 | 281096 | Fully Vaccinated | ≤ 6 months | 2 | 2.28 |
| BA.2 | 281095 | Boosted | ≤ 6 months | 2 | 110.39 |
| BA.2 | 281085 | Boosted | ≤ 6 months | 6 | 101.62 |

| BA.2 | 281079 | Boosted | ≤ 6 months | 2 | 26.48 |
| --- | --- | --- | --- | --- | --- |
| BA.2 | 281064 | Boosted | ≤ 6 months | 9 | 25.37 |
| BA.2 | 281063 | Fully Vaccinated | 7- 12 months | 2 | 45.64 |
| BA.2 | 281048 | Unvaccinated | NA | 3 | 0.71 |
| BA.2 | 281035 | Boosted | Unknown | 16 | 281.08 |
| BA.2 | 281035 | Boosted | Unknown | 3 | 1.29 |
| BA.2 | 281034 | Boosted | Unknown | 3 | 108.83 |
| BA.2 | 281028 | Boosted | 7- 12 months | 2 | 79.58 |
| BA.2 | 281025 | Boosted | ≤ 6 months | 2 | 238.28 |
| BA.2 | 281024 | Boosted | Unknown | 3 | 104. 19 |
| BA.2 | 281023 | Boosted | ≤ 6 months | 10 | 425.46 |
| BA.2 | 281023 | Boosted | ≤ 6 months | 2 | 78.81 |
| BA.2 | 281022 | Boosted | ≤ 6 months | 10 | 381. 13 |
| BA.2 | 281022 | Boosted | ≤ 6 months | 2 | 151.7 |
| BA.2 | 280961 | Boosted | ≤ 6 months | 17 | 398.79 |
| BA.2 | 280961 | Boosted | ≤ 6 months | 3 | 90.66 |
| BA.2 | 280935 | Unvaccinated | NA | 2 | 41.58 |
| BA.2 | 280851 | Boosted | ≤ 6 months | 3 | 49. 15 |
| BA.2 | 280850 | Boosted | ≤ 6 months | 3 | 125.84 |
| BA.2 | 280836 | Boosted | ≤ 6 months | 19 | 302.75 |
| BA.2 | 280836 | Boosted | ≤ 6 months | 3 | 18.4 |
| BA.2 | 280835 | Fully Vaccinated | ≤ 6 months | 3 | 17.91 |
| BA.2 | 280825 | Unvaccinated | NA | 3 | 128.98 |
| BA.2 | 280809 | Fully Vaccinated | Unknown | 3 | 2.69 |
| BA.2 | 280800 | Boosted | 7- 12 months | 3 | 37.85 |
| BA.2 | 280795 | Boosted | ≤ 6 months | 3 | 36.89 |
| BA.2 | 280755 | Boosted | ≤ 6 months | 3 | 49.08 |
| BA.2 | 280710 | Unvaccinated | NA | 2 | 414.81 |
| BA.2 | 280694 | Unvaccinated | NA | 16 | 366.92 |

| BA.2 | 280694 | Unvaccinated | NA | 8 | 188.45 |
| --- | --- | --- | --- | --- | --- |
| BA.2 | 280694 | Unvaccinated | NA | 2 | 64.56 |
| BA.2 | 280678 | Boosted | ≤ 6 months | 3 | 397.23 |
| BA.2 | 280561 | Boosted | ≤ 6 months | 3 | 23.66 |
| BA.2 | 280512 | Fully Vaccinated | 7- 12 months | 3 | 16.08 |
| BA.2 | 280502 | Boosted | ≤ 6 months | 2 | 104.89 |
| BA.2 | 280497 | Unvaccinated | NA | 6 | 0.4 |
| BA.2 | 280497 | Unvaccinated | NA | 3 | 0.35 |
| BA.2 | 278523 | Fully Vaccinated | ≤ 6 months | 3 | 37.08 |
| BA.2 | 278514 | Fully Vaccinated | 7- 12 months | 4 | 240.98 |
| BA.2 | 278506 | Unvaccinated | NA | 3 | 15.4 |
| BA.2 | 278489 | Boosted | ≤ 6 months | 2 | 98 |
| BA.2 | 278482 | Unvaccinated | NA | 4 | 7.85 |
| BA.2 | 278478 | Fully Vaccinated | ≤ 6 months | 3 | 62.85 |
| BA.2 | 278475 | Unvaccinated | NA | 4 | 0.2 |
| BA.2 | 278468 | Boosted | ≤ 6 months | 4 | 21.36 |
| BA.2 | 278467 | Boosted | ≤ 6 months | 3 | 53.24 |
| BA.2 | 278465 | Unvaccinated | NA | 2 | 15.68 |
| BA.2 | 278464 | Boosted | ≤ 6 months | 10 | 402.23 |
| BA.2 | 278464 | Boosted | ≤ 6 months | 2 | 297.23 |
| BA.2 | 278463 | Boosted | ≤ 6 months | 9 | 218.3 |
| BA.2 | 278463 | Boosted | ≤ 6 months | 2 | 33.79 |
| BA.2 | 278462 | Boosted | ≤ 6 months | 9 | 295.83 |
| BA.2 | 278462 | Boosted | ≤ 6 months | 2 | 248. 17 |
| BA.2 | 278461 | Unvaccinated | NA | 27 | 109.59 |
| BA.2 | 278461 | Unvaccinated | NA | 3 | 0.38 |
| BA.2 | 278459 | Fully Vaccinated | ≤ 6 months | 3 | 1. 14 |
| BA.2 | 278458 | Boosted | ≤ 6 months | 3 | 42.44 |
| BA.2 | 278457 | Boosted | ≤ 6 months | 3 | 12.33 |

| BA.2 | 278456 | Fully Vaccinated | 7- 12 months | 10 | 439.35 |
| --- | --- | --- | --- | --- | --- |
| BA.2 | 278456 | Fully Vaccinated | 7- 12 months | 3 | 131.74 |
| BA.2 | 278455 | Fully Vaccinated | 7- 12 months | 3 | 112.87 |
| BA.2 | 278454 | Unvaccinated | NA | 2 | 17.94 |
| BA.2 | 278446 | Boosted | ≤ 6 months | 3 | 336.91 |
| BA.2 | 278445 | Unvaccinated | NA | 3 | 0. 13 |
| BA.2 | 278444 | Unvaccinated | NA | 3 | 0. 18 |
| BA.2 | 278443 | Fully Vaccinated | ≤ 6 months | 6 | 46.73 |
| BA.2 | 278442 | Fully Vaccinated | ≤ 6 months | 3 | 4.46 |
| BA.2 | 278441 | Unvaccinated | NA | 3 | 0.24 |
| BA.2 | 278440 | Fully Vaccinated | 7- 12 months | 3 | 1.57 |
| BA.2 | 278438 | Fully Vaccinated | ≤ 6 months | 30 | 395.52 |
| BA.2 | 278438 | Fully Vaccinated | ≤ 6 months | 3 | 4.72 |
| BA.2 | 278437 | Boosted | 7- 12 months | 3 | 55.05 |
[truncated: 103,220 more chars]
